# Stereochemical Control in the Still-Wittig Rearrangement Synthesis of Cyclohexyl (*Z*)-Alkene Inhibitors of Pin1

**DOI:** 10.1371/journal.pone.0139543

**Published:** 2015-10-07

**Authors:** Xingguo R. Chen, Shuang A. Fan, Rachel I. Ware, Felicia A. Etzkorn

**Affiliations:** Department of Chemistry, Virginia Tech, Blacksburg, Virginia, 24061, United States of America; National Cancer Institute at Frederick, UNITED STATES

## Abstract

Three stereoisomeric inhibitors of Pin1: (2*R*,5*S*)-, (2*S*,5*R*)- and (2*S*,5*S*)-Ac–pSer–Ψ[(*Z*)CH = C]–pipecolyl(Pip)–2-(2-naphthyl)ethylamine **1**, that mimic L-pSer–D-Pro, D-pSer–L-Pro, and D-pSer–D-Pro amides respectively, were synthesized by a 13-step route. The newly formed stereogenic centers in the pipecolyl ring were introduced by Luche reduction, followed by stereospecific [2,3]-Still-Wittig rearrangement. The (*Z*)- to (*E*)-alkene ratio in the rearrangements were consistently 5.5 to 1. The stereochemistry at the original Ser α-carbon controlled the stereochemistry of the Luche reduction, but it did not affect the stereochemical outcome of the rearrangement, which consistently gave the (*Z*)-alkene. The epimerized by-product, **(2*S*,5*S*)-10**, resulting from the work-up after Na/NH_3_ debenzylation of **(2*S*,5*R*)-9**, was carried on to the **(2*S*,5*S*)-1** isomer. Compound **(2*S*,5*S*)-10** was resynthesized from the Luche reduction by-product, **(2*R*,3*R*)-3**, and the stereochemistry was confirmed by comparison of the optical rotations. The IC_50_ values for **(2*R*,5*S*)-1**, **(2*S*,5*R*)-1** and **(2*S*,5*S*)-1** Pin1 inhibition were: 52, 85, and 140 μM, respectively.

## Introduction

Pin1 (Peptidyl-prolyl isomerase (PPIase) interacting with never-in-mitosis A kinase 1) catalyzes the isomerization of phospho-Ser/Thr–Pro (pSer/Thr-Pro) amides, and negatively regulates the G2 to M transition in the cell cycle.[1,2] Pin1 plays an important role in cancer,[3] Alzheimer’s disease,[4,5] and asthma,[6] and regulates the uncoating and replication processes of human immunodeficiency virus type 1 (HIV–1).[7,8] Specific inhibitors for Pin1 are valuable for understanding its role in these diseases.[3,9] Inhibitors of Pin1 designed and synthesized by several groups were recently reviewed. [9]

In our own work, we have synthesized competitive inhibitors of Pin1 that incorporated phospho-Ser–Ψ[(*Z*)CH = C]–Pro and phospho-Ser–Ψ[(*E*)CH = C]–Pro into pentapeptides.[10,11] Our peptidomimetics were used to elucidate the inhibition specificity,[11,12] structure,[13] and dynamics[14,15] of the Pin1 catalytic and WW domains with these cis and trans amide isosteres.

**Fig 1:**
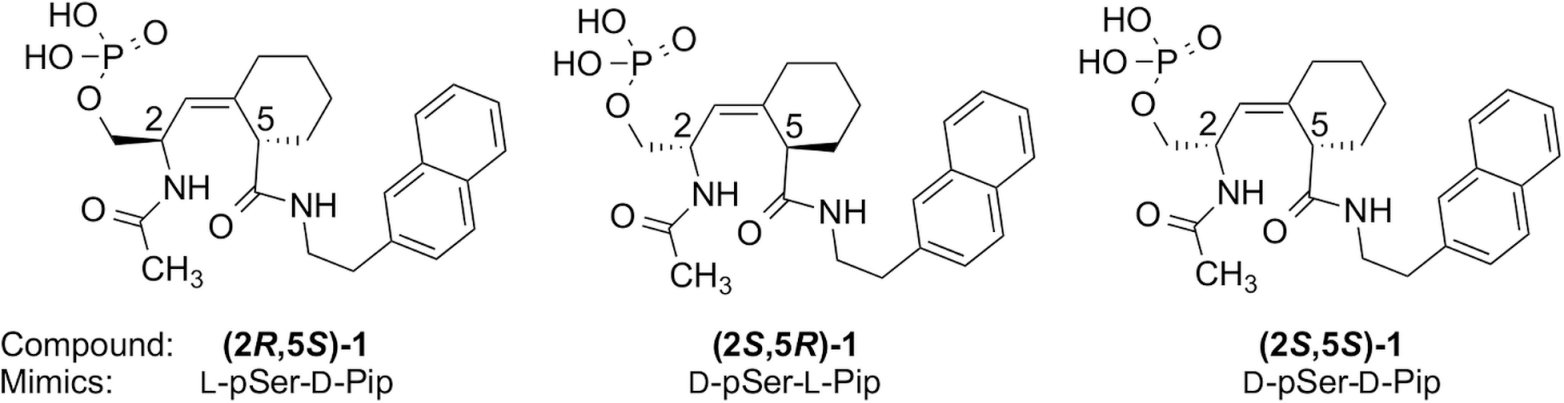
Designed enantiomeric inhibitors synthesized.

By screening combinatorial peptide libraries containing unnatural amino acid residues, the Fischer group identified several potent peptide inhibitors of Pin1.[16] Replacement of Pro with pipecolate (Pip) in an octapeptide improved the inhibition by 100-fold. [16] Replacement of L-Thr with D-Thr in the octapeptide improved the inhibition by 150-fold.[16] The combination of D-Thr and Pip at the appropriate positions of the octapeptide gave the best inhibitor for Pin1 to date, with a *K*_i_ value of 1.2 nM.[16] Zhang et al. reported the crystal structures of Pin1 in complex with Fisher’s pentapeptides, Ac–Phe–D/L-pThr–Pip–Nal–Gln–NH_2_ (Pip = piperidyl, Nal = 2-naphthylalanine).[17] Electrostatic contacts and hydrogen bonds between the phosphate group and Pin1, and hydrophobic interactions between the Pip and Nal residues and Pin1, accounted for the potent inhibitory activity.[17] These results were the starting point for the design of the cyclohexyl alkene inhibitors that we now report (Fig 1).

In the present study, we wanted to see if we could control all aspects of the stereochemistry in the Still-Wittig rearrangement. The key step in the synthesis of the Ser-*cis*-Pro (*Z*)-alkene isostere was the Still-Wittig [2,3]-sigmatropic rearrangement.[10,18,19] In that native-like synthesis, Felkin-Ahn reduction[20] of an L-Ser-based intermediate ketone set up the (*S*)-allylic alcohol stereochemistry to introduce the *cis*-L-Pro mimic stereochemistry in the Still-Wittig rearrangement to give the (*Z*)-alkene.[10] The *Z*/*E* selectivity was solvent dependent; THF favored (*Z*)-selectivity, while toluene favored (*E*)-selectivity.[21] To produce the Ser-*trans*-L-Pro mimic stereochemistry in the (*E*)-alkene,[10] Luche reduction[22] to the opposite (*R*)-allylic alcohol preceeded an Ireland-Claisen rearrangement.[23]

In this work, strategic combinations of these stereospecific reactions were used to synthesize three stereoisomeric inhibitors of Pin1. The Luche reduction[22] could be used to install the anti-stereochemistry of the allylic alcohol. In addition to using such precursors in an Ireland-Claisen rearrangement to make (*E*)-alkene isosteres from the Luche precursor,[10] we reasoned that the Still-Wittig rearrangement would transfer the alcohol stereochemistry to the desired stereogenic center in the ring. Thus, Ser-*cis*-Pro mimics could be made with opposite stereogenic centers at the Ser and Pro mimic alpha-carbons, i. e. L-Ser-D-Pro or D-Ser-L-Pro, which had not been made before.

## Materials and Methods

### Synthesis

#### General

Unless otherwise indicated, all reactions were carried out under dry N_2_ in flame-dried glassware. THF was distilled from Na-benzophenone, and CH_2_Cl_2_ was dried by passage through dry alumina. Anhydrous DMF (99.8%), MeOH, and DIEA were used directly from sealed bottles. Brine (NaCl), Na_2_S_2_O_3_, NaHCO_3_, and NH_4_Cl refer to saturated aqueous solutions, and HCl refers to a 1 N aqueous solution, unless otherwise noted. Flash chromatography was performed on 230–400 mesh silica gel with reagent grade solvents. Analytical HPLC were obtained on a 4.6 × 50 mm C18 column with 10% CH_3_CN/H_2_O for 3 min followed by a 10% to 90% CH_3_CN/H_2_O gradient over 6 min unless otherwise noted. HPLC results are reported as retention time, integrated % purity. ^1^H-, ^13^C-, and ^31^P-NMR spectra were obtained at ambient temperature in CDCl_3_, at 500, 125, and 162 MHz, respectively, unless otherwise noted. Chemical shifts are reported in parts per million (ppm) downfield from tetramethylsilane (TMS). NMR data are reported as follows: ∂ chemical shift, multiplicity: singlet (s), doublet (d), triplet (t), multiplet (m), broad singlet (br s), coupling constants *J* in Hz, and integration. Spectra and HPLC chromatograms are given in Fig C in S1 Dataset.

#### L- and D-Serine Weinreb amides

Synthesized by the method of Niel.[24]

#### 1-Iodocyclohexene

Synthesized by the method of Barton.[25]

#### Ketone, (*S*)-2

A solution of 1-iodocyclohexene (9.8 g, 47 mmol) in THF (285 mL) was cooled to –78°C and *s*-BuLi (1.4 M in cyclohexane, 67 mL, 94 mmol) was added dropwise over 15 min to generate 1-cyclohexenyl lithium. The resulting solution was stirred at −78°C for 3 h. In another flask, a solution of Boc–L-Ser–*N-*(Me)-*O-*Me[10] Weinreb amide (9.9 g, 29 mmol) in THF (82 mL) was cooled to −60°C, and *i*-PrMgCl (2.0 M in THF, 14 mL, 28 mmol) was added dropwise and stirred for 55 min. The 1-cyclohexenyl lithium solution was added to the solution of the deprotonated Weinreb amide via cannula. The mixture was stirred at −78°C for 1 h and warmed slowly to rt. The mixture was stirred overnight, quenched with NH_4_Cl (50 mL) at –30° to –40°C, diluted with EtOAc (300 mL), washed with NH_4_Cl (2 × 200 mL), NaHCO_3_ (300 mL) and brine (300 mL). The organic solution was then dried with Na_2_SO_4_ and evaporated at reduced pressure. The crude product was purified by flash chromatography with EtOAc:hexanes (1:18), followed by EtOAc:hexanes (1:15) to give a colorless oil (6.4 g, 61%). ^1^H NMR (400 MHz): δ 7.34–7.22 (m, 5H), 6.91 (m, 1H), 5.59 (d, *J* = 7.5, 1H), 5.13 (dt, *J* = 4.3, 8.4, 1H), 4.54 (d, *J* = 12.4, 1H), 4.42 (d, *J* = 12.4, 1H), 3.67 (d, *J* = 3.8, 2H), 2.39–2.10 (m, 4H), 1.65–1.58 (m, 4H), 1.44 (s, 9H); ^13^C NMR (100 MHz): δ 197.8, 155.5, 141.8, 137.8, 137.4, 128.4, 127.8, 127.6, 79.8, 73.1, 71.3, 54.3, 28.4, 26.2, 23.4, 21.8, 21.5.

#### Ketone (*R*)-2

Yield 81%. The ^1^H and ^13^C NMR spectra were identical to **(*S*)-2.** FTIR 3448 cm^–1^ (NH), 1711 cm^–1^ (C = O).

#### Allyl alcohols, (2*S*,3*R*)-3 and (2*S*,3*S*)-3

A solution of ketone **(*S*)-2** (6.2 g, 17 mmol) in THF:CH_3_OH (2.5:1, 500 mL) was cooled in an ice bath. CeCl_3_ 7H_2_O (9.6 g, 26 mmol) was added and stirred for 15 min. NaBH_4_ (3.9 g, 0.10 mol) was added in three portions. The reaction was stirred for 4.5 h, quenched with NH_4_Cl (50 mL), diluted with EtOAc (250 mL), and washed with NH_4_Cl (2 × 250 mL) and brine (250 mL). The organic solution was dried with Na_2_SO_4_ and evaporated under reduced pressure to give a colorless oil as an inseparable mixture of two diastereomers, **(2*S*,3*R*)-3** and **(2*S*,3*S*)-3** (6:1 by ^1^H NMR, 6.2 g, 100%). The crude product was used in the next step without further purification. ^1^H NMR: δ 7.37–7.29 (m, 5H), 5.72 (br, 1H), 5.26 (d, *J* = 8.6, 0.85H), 5.12 (d, *J* = 9.2, 0.15H), 4.55 (d, *J* = 12.4, 0.15H), 4.53 (d, *J* = 12.0, 0.85H), 4.49 (d, *J* = 12.4, 0.15H), 4.44 (d, *J* = 12.0, 0.85H), 4.18 (br, 0.15H), 4.06 (t, *J* = 6.3, 0.85H), 3.82 (m, 0.85H), 3.76 (dd, *J* = 2.4, 9.4, 1H), 3.64 (m, 0.3H), 3.56 (dd, *J* = 2.6, 9.2, 0.85H), 3.02 (d, *J* = 7.4, 0.85H), 2.02–1.93 (m, 4H), 1.63–1.48 (m, 4H), 1.44 (s, 7.65H), 1.43 (s, 1.35H).

#### Allyl alcohols, (2*R*,3*S*)-3 and (2*R*,3*R*)-3

Yield 98%. The ^1^H NMR spectrum was identical to the **(2*S*,3*R*)-3** and **(2*S*,3*S*)-3** mixture. HRMS (ESI^+^, *m/z*): calcd for C_21_H_31_NO_4_Na [M+Na]^+^ 384.2145, found 384.2126.

#### Dibenzyl amine, (2*S*,3*R*)-4

A mixture of **(2*S*,3*R*)-3** and **(2*S*,3*S*)-3**, (0.50 g, 1.4 mmol) was dissolved in CH_2_Cl_2_ (9 mL) and TFA (4.5 mL, 58 mmol) was added and stirred for 1.5 h. The mixture was concentrated, and the residue was dissolved in CHCl_3_ (13 mL). DIEA (1.4 g, 11 mmol) and BnBr (0.59 g, 3.4 mmol) were added, and the solution was stirred for 52 h. The solution was diluted with EtOAc (25 mL), washed with NH_4_Cl (2 × 25 mL) and brine (25 mL). The organic solution was dried with Na_2_SO_4_ and evaporated at reduced pressure. The crude product was purified by flash chromatography with EtOAc:hexanes (1:25), followed by EtOAc:hexanes (1:12) to give a colorless oil as a single diastereomer (0.40 g, 65%). HPLC: 18.8 min, 90%, λ = 210 nm; ^1^H NMR: δ 7.40–7.19 (m, 15H), 5.64 (m, 1H), 4.60 (d, *J* = 11.8, 1H), 4.54 (d, *J* = 11.8, 1H), 4.32 (d, *J* = 8.1, 1H), 3.94 (dd, *J* = 4.6, 9.7, 1H), 3.86 (dd, *J* = 5.2, 9.7, 1H), 3.83 (d, *J* = 13.8, 2H), 3.56 (d, *J* = 13.8, 2H), 2.93 (dt, *J* = 4.9, 8.1, 1H), 2.75 (br s, 1H), 2.09–1.99 (m, 2H), 1.80–1.75 (m, 1H), 1.65–1.47 (m, 4H), 1.42–1.37 (m, 1H); ^13^C NMR (100 MHz): δ 140.2, 138.7, 138.1, 129.2, 128.6, 128.2, 127.9, 127.8, 127.0, 125.0, 77.7, 73.6, 68.6, 58.0, 55.0, 25.3, 22.74, 22.72, 22.67; HRMS (ESI^+^, *m/z*): calcd for C_30_H_36_NO_2_ [M+H]^+^ 442.2741, found 442.2727.

#### Dibenzyl amine, (2*R*,3*S*)-4

Yield 64%. HPLC: 18.7 min, 94%, 254 nm. The ^1^H NMR spectrum was identical to **(2*S*,3*R*)-4**. FTIR (neat): 3676 cm^–1^ (OH), 2988 cm^–1^ (CH), 2901 cm^–1^ (CH), 1265 cm^–1^ (CO); HRMS (ESI^+^, *m/z*): calcd for C_30_H_36_NO_2_ [M+H]^+^ 442.2741, found 442.2750; [α]^25^_D_ +6.6°(*c* 0.51, CH_3_OH).

#### Dibenzyl amine, (2*R*,3*R*)-4

Isolated yield 8% after dibenzylation of mixture **(2*R*,3*S*)-3** and **(2*R*,3*R*)-3** and chromatographic separation. ^1^H NMR (400 MHz): δ 7.39–7.22 (m, 15H), 5.59 (s, 1H), 4.56 (d, *J* = 12.0, 1H), 4.48 (d, *J* = 12.0, 1H), 4.36 (br, 1H), 3.94 (d, *J* = 13.0, 2H), 3.82 (d, *J* = 10.0, 1H), 3.68 (m, 3H), 3.52 (dd, *J* = 3.2, 10.5, 1H), 3.01 (ddd, *J* = 3.1, 8.1, 10.6, 1H), 1.96 (m, 2H), 1.82 (d, *J* = 16.5, 1H), 1.43 (m, 5H); ^13^C NMR (100 MHz): δ 139.3, 138.5, 137.2, 129.4, 128.6, 128.5, 127.8, 127.6, 127.3, 126.8, 73.5, 72.7, 67.8, 59.1, 54.5, 25.3, 22.8, 22.7, 22.4; HRMS (ESI^+^, *m/z*): calcd. for C_30_H_36_NO_2_ [M+H]^+^ 442.2741, found 442.2712; [α]^22^_D_ −68°(*c* 0.36, CH_3_OH).

#### Stannane, (2*S*,3*R*)-5

To a solution of dibenzyl amine, **(2*S*,3*R*)-4**, (3.5 g, 7.9 mmol) in THF (115 mL), 18-crown–6 (2.7 g, 10 mmol) in THF (5 mL) was added, followed by the addition of KH (0.48 g, 12 mmol). A solution of *n*-Bu_3_SnCH_2_I (5.1 g, 12 mmol), prepared as previously reported,[26] was added and the mixture was stirred for 2.5 h. The reaction was quenched with CH_3_OH (15 mL), and the resulting yellow solution was diluted with EtOAc (350 mL) and washed with NH_4_Cl (2 × 200 mL). The organic solution was dried with Na_2_SO_4_ and evaporated under reduced pressure. The crude product was purified by flash chromatography with hexanes, followed by EtOAc:hexanes (1:150) to give a colorless oil (4.9 g, 83%). ^1^H NMR: δ 7.42–7.17 (m, 15H), 5.54 (br, 1H), 4.56 (d, *J* = 12.1, 1H), 4.51 (d, *J* = 12.1, 1H), 3.87 (dd, *J* = 2.7, 10.3, 1H), 3.82 (dd, *J* = 6.7, 10.3, 1H), 3.76 (d, *J* = 13.6, 2H), 3.70 (d, *J* = 13.6, 2H), 3.61 (d, *J* = 9.8, 1H), 3.54 (d, *J* = 8.0, 1H), 3.24 (d, *J* = 9.8, 1H), 2.90 (ddd, *J* = 2.6, 6.7, 8.0, 1H), 2.07 (m, 2H), 1.63–1.29 (m, 13H), 1.24 (app. sext., *J* = 7.4, 6H), 0.93–0.74 (m, 16H); ^13^C NMR (100 MHz): δ 140.9, 139.3, 136.0, 129.4, 128.4, 128.0, 127.5, 127.4, 126.7, 126.5, 88.3, 73.4, 68.4, 58.4, 58.1, 55.0, 29.3, 27.5, 25.4, 23.0, 22.7, 22.4, 13.9, 9.0; HRMS (ESI^+^, *m/z*): calcd for C_43_H_64_NO_2_Sn [M+H]^+^ 746.3954, found 746.3956; [α]^22^_D_ –2.3°(*c* 2.9, CHCl_3_).

#### Stannane, (2*R*,3*S*)-5

Yield 58%. The ^1^H NMR spectrum was identical to **(2*S*,3*R*)-5**. FTIR (neat) 1265 cm^–1^ (C-O); HRMS (ESI^+^, *m/z*): calcd for C_43_H_64_NO_2_Sn [M+H]^+^ 746.3954, found 746.3933.

#### Stannane, (2*R*,3*R*)-5

Yield 92%. ^1^H NMR: δ 7.40–7.16 (m, 15H), 5.59 (s, 1H), 4.42 (d, *J* = 12.0, 1H), 4.32 (d, *J* = 12.0, 1H), 3.94 (d, *J* = 13.6, 2H), 3.86 (d, *J* = 13.7, 2H), 3.70 (d, *J* = 7.6, 1H), 3.67 (d, *J* = 9.9, 1H), 3.52 (dd, *J* = 5.8, 9.8, 1H), 3.45 (dd, *J* = 4.0, 9.8, 1H), 3.35 (d, *J* = 9.9, 1H), 2.96 (ddd, *J* = 4.2, 5.6, 8.6, 1H), 2.06–1.98 (m, 2H), 1.76 (m, 1H), 1.61–1.42 (m, 11H), 1.32 (sextet, *J* = 7.3, 6H), 0.95 (t, *J* = 8.2, 6H), 0.89 (t, *J* = 7.3, 9H); ^13^C NMR (100 MHz): δ 141.7, 139.0, 135.6, 129.1, 128.3, 128.0, 127.6, 127.4, 126.5, 126.1, 90.5, 73.2, 71.1, 58.3, 58.0, 55.7, 29.4, 27.6, 25.3, 23.6, 22.9, 22.8, 13.9, 9.0; HRMS (ESI^+^, *m/z*): calcd. for C_43_H_64_NO_2_Sn [M+H]^+^ 746.3959, found 746.3937; [α]^22^_D_ −27°(*c* 0.52, CH_2_Cl_2_).

#### (*Z*)-Alkene, (2*R*,3*Z*,5*S*)-6

The intermediate **(2*S*,3*R*)-5** (2.45 g, 3.29 mmol) was dissolved in THF (35 mL) and dried with 4 Å molecular sieves for 2 h. The solution was transferred to another flask via cannula and cooled to –78°C. *n*-BuLi (2.5 M in hexanes, 1.7 mL, 4.3 mmol) was added slowly and stirred for 2.5 h. (The reaction time was critical. If the reaction was quenched before completion, the remaining starting material was converted into the corresponding methyl ether, and could not be recovered. Prolonged reaction time resulted in removal of benzyl protecting groups. The color change from pale yellow to red was a reasonably good indicator for the completion of the reaction.) The reaction was quenched with CH_3_OH (8 mL), diluted with CH_2_Cl_2_, washed with NH_4_Cl (150 mL) and brine (150 mL). The organic solution was dried with Na_2_SO_4_ and evaporated under reduced pressure. The ratio of (*Z*)- to (*E*)-alkene was 5.5:1 as calculated by the NMR of the crude product. The crude product was purified by flash chromatography with EtOAc:hexanes (1:25), followed by EtOAc:hexanes (1:12) to give a colorless oil (0.94 g, 62%). HPLC: 100% H_2_O for 3 min, then 0% to 100% CH_3_CN/H_2_O gradient over 15 min, 100% CH_3_CN for 15 min, flow rate 1.0 mL/min, λ = 254 nm, 21.0 min, 98%. ^1^H NMR: δ 7.34–7.18 (m, 15H), 5.40 (dd, *J* = 1.4, 10.4, 1H), 4.49 (d, *J* = 12.6, 1H), 4.44 (d, *J* = 12.6, 1H), 3.76–3.67 (m, 5H), 3.48 (t, *J* = 8.8, 1H), 3.44 (d, *J* = 14.2, 2H), 3.33 (ddd, *J* = 4.8, 8.2, 10.5, 1H), 2.58 (dd, *J* = 3.4, 8.2, 1H), 2.52 (m, 1H), 2.32 (m, 1H), 2.18 (d, *J* = 13.7, 1H), 1.89 (m, 1H), 1.68(d, *J* = 13.4, 1H), 1.61–1.52 (m, 2H), 1.50–1.38 (m, 2H); ^13^C NMR (100 MHz): δ 144.9, 140.5, 137.8, 128.5, 128.4, 128.3, 128.0, 127.8, 127.0, 122.0, 73.2, 72.3, 63.6, 54.8, 54.5, 39.1, 33.4, 29.9, 28.9, 22.2; HRMS (ESI^+^, *m/z*): calcd. for C_31_H_38_NO_2_ [M+H]^+^ 456.2897, found 456.2916; [α]^25^_D_ +49°(c 0.30, CH_3_OH).

#### (*Z*)-Alkene, (2*S*,3*Z*,5*R*)-6

The ratio of (*Z*)- to (*E*)-alkene was 5.5:1 as calculated by the NMR of the crude product. Yield 58%. The ^1^H NMR spectrum was identical to **(2*R*,3*Z*,5*S*)-6**. FTIR (neat): 3488 cm^–1^ (OH), 3057 cm^–1^ (sp^2^ CH), 2933–2851 cm^–1^ (sp^3^ CH), 1265 cm^–1^ (CO); HRMS (ESI^+^, *m/z*): calcd for C_31_H_38_NO_2_ [M+H]^+^ 456.2897, found 456.2874; [α]^25^_D_ –49°(*c* 0.33, CH_3_OH).

#### (*Z*)-Alkene, (2*S*,3*Z*,5*S*)-6

The ratio of (*Z*)- to (*E*)-alkene was 5.5:1 as calculated by the NMR of the crude product. Yield 59%. ^1^H NMR: δ 7.39–7.20 (m, 15H), 5.42 (dd, *J* = 10, 1, 1H), 4.57 (d, *J* = 12, 1H), 4.52 (d, *J* = 12, 1H), 3.83 (m, 2H), 3.73 (m, 1H), 3.61 (m, 4H), 3.43 (m, 2H), 2.21 (m, 2H), 2.06 (m, 1H), 1.75 (m, 1H), 1.46 (m, 3H), 1.24 (m, 2H); ^13^C NMR: δ 144.8, 139.1, 138.6, 129.9, 128.5, 128.3, 127.8, 127.7, 127.1, 123.0, 73.4, 70.1, 63.1, 54.7, 53.3, 39.1, 33.0, 28.02, 27.99, 21.7; 1D nOe H_f_−H_m_; HRMS (ESI^+^, *m/z*): calcd for C_31_H_37_NO_2_ [M+H]^+^ 456.2897, found 456.2878; cald for C_31_H_37_NO_2_Na [M+Na]^+^ 478.2717 found 478.2678; [α]^22^_D_ +36°(*c* 1.3 CH_3_OH).

#### Benzylamino alcohol, (2*R*,5*S*)-7

To a flask containing (*Z*)-alkene**, (2*R*,5*S*)-6** (303 mg, 0.665 mmol) was added 20% Pd(OH)_2_/C (25.9 mg). CH_3_OH (21 mL) was added, followed by the addition of HCOOH (4.59 g, 99.8 mmol). The reaction mixture was stirred and monitored by TLC with EtOAc:hexanes (1:4). When all starting material was consumed, approximately 15 min, the mixture was filtered through Celite immediately because the other benzyl protecting groups could be removed with prolonged reaction time. The Celite was washed with CH_3_OH (250 mL), and the combined filtrate was concentrated under reduced pressure. The residue was neutralized with NaHCO_3_ and extracted with CH_2_Cl_2_ (7 × 80 mL). The combined organic solution was dried with Na_2_SO_4_ and evaporated to give a colorless oil (237 mg, 97%). It was critical to remove formic acid completely to prevent the formation of formamide by-product in the subsequent acylation step. The resulting secondary amine was not stable on silica gel, and no further purification was performed. ^1^H NMR: δ 7.35–7.22 (m, 10H), 5.13 (dd, *J* = 1.9, 9.6, 1H), 4.49 (s, 2H), 3.85 (d, *J* = 13.4, 1H), 3.75–3.70 (m, 2H), 3.65 (d, *J* = 13.4, 1H), 3.50 (dd, *J* = 5.5, 10.6, 1H), 3.47 (dd, *J* = 6.2, 8.7, 1H), 3.31 (dd, *J* = 7.2, 8.7, 1H), 2.78 (m, 1H), 2.25 (ddt, *J* = 1.7, 4.4, 13.5, 1H), 2.12 (m, 3H), 1.82 (m, 1H), 1.72 (m, 1H), 1.56 (m, 1H), 1.52–1.41 (m, 2H), 1.40–1.29 (m, 1H); ^13^C NMR: δ 143.3, 140.5, 137.9, 128.6, 128.5, 128.2, 128.1, 127.9, 127.1, 126.5, 73.33, 73.31, 63.7, 53.6, 51.1, 39.9, 33.3, 29.3, 28.5, 22.1.

#### Benzylamino alcohol, (2*S*,5*R*)-7

Yield 94%. The ^1^H and ^13^C NMR spectra were identical to **(2*R*,5*S*)-7**. FTIR (neat): 3054 cm^–1^ (sp^2^ CH), 1265 cm^–1^ (CO); MS (ESI^+^, *m/z*): calcd for C_24_H_32_NO_2_ [M+H]^+^ 366.2, found 366.5.

#### Benzylamino alcohol, (2*S*,5*S*)-7

Yield 86%. ^1^H NMR: δ7.37–7.20 (m, 10H), 5.39 (dd, *J* = 1.8, 7.3, 1H), 4.56 (d, *J* = 12.1, 1H), 4.53 (d, *J* = 12.0, 1H), 3.77–3.66 (m, 3H), 3.61 (m, 2H), 3.56 (dd, *J* = 5.1, 10.0, 1H), 3.50 (dd, *J* = 6.9, 10.6, 1H), 2.80 (m, 1H), 2.23 (m, 1H), 2.06 (d, *J* = 13.8, 1H), 1.77–1.66 (m, 2H), 1.50–1.25 (m, 4H).

#### Acetylbenzylamino alcohol, (2*R*,5*S*)-8

Benzylamino alcohol, **(2*R*,5*S*)-7** (237 mg, 0.648 mmol) was dissolved in CH_2_Cl_2_ (7 mL), and Et_3_N (197 mg, 1.94 mmol) and Ac_2_O (132 mg, 1.30 mmol) were added and stirred for 30 min. The mixture was washed with NH_4_Cl (30 mL), NaHCO_3_ (30 mL), and water (30 mL). The organic solution was dried with Na_2_SO_4_ and evaporated at reduced pressure. The crude product was purified by flash chromatography with EtOAc:hexanes (1:2), followed by EtOAc:hexanes (1:1) to give a colorless oil (215 mg, 81%). HPLC: 15.7 min, 94%, λ = 210 nm; **(2*R*,5*S*)-8** exists as a pair of rotamers, in CDCl_3_, the ratio was ca. 7:3, in DMSO-*d*_6_, the ratio was ca. 1:1; ^1^H NMR (CDCl_3_): δ 7.34–7.17 (m, 10H), 5.58 (dd, *J* = 1.3, 9.9, 0.7H), 5.25 (dt, *J* = 6.3, 9.9, 0.7H), 5.18 (d, *J* = 8.9, 0.3H), 4.89 (t, *J* = 7.7, 0.3H), 4.59 (d, *J* = 15.6, 0.3H), 4.56 (d, *J* = 15.6, 0.3H), 4.51 (s, 1.3H), 4.48 (d, *J* = 12.2, 0.7H), 4.45 (d, *J* = 12.2, 0.7H), 4.37 (d, *J* = 12.0, 0.3H), 4.33 (d, *J* = 12.0, 0.3H), 3.75 (t, *J* = 9.8, 0.3H), 3.71 (t, *J* = 10.6, 0.7H), 3.62–3.49 (m, 2.3H), 3.34 (dd, *J* = 7.2, 9.5, 0.3H), 3.31 (dd, *J* = 7.0, 9.0, 0.3H), 2.91–2.88 (m, 0.7H), 2.80–2.78 (m, 0.3H), 2.30 (br, 0.3H), 2.28 (s, 0.9H), 2.20–2.14 (m, 1H), 2.03 (s, 2H), 1.98–1.94 (m, 1H), 1.71–1.69 (m, 2.7H), 1.50–1.48 (m, 1H), 1.42–1.29 (m, 2H), 1.13–1.05 (m, 1H); ^1^H NMR (DMSO-d_6_): δ 7.37–7.14 (m, 10H), 5.46 (m, 0.5H), 5.22 (d, *J* = 8.7, 0.5H), 5.03 (d, *J* = 7.3, 0.5H), 4.89 (dt, *J* = 4.3, 8.8, 0.5H), 4.63 (d, *J* = 15.8, 0.5H), 4.60 (t, *J* = 5.6, 0.5H), 4.57 (s, 1H), 4.53 (t, *J* = 5.5, 0.5H), 4.45 (d, *J* = 12.4, 0.5H), 4.43 (d, *J* = 12.4, 0.5H), 4.37 (d, *J* = 12.0, 0.5H), 4.34 (d, *J* = 12.0, 0.5H), 4.28 (d, *J* = 15.8, 0.5H), 3.56 (dd, *J* = 7.6, 10.0, 0.5H), 3.49–3.34 (m, 3.5H), 2.81 (m, 0.5H), 2.65 (m, 0.5H), 2.19 (s, 1.5H), 2.06–1.99 (m, 1H), 1.86 (s, 1.5H), 1.84–1.76 (m, 2H), 1.63–1.55 (m, 1H), 1.40–1.31 (m, 2H), 1.21–1.13 (m, 1H), 0.98 (m, 0.5H), 0.85 (m, 0.5H); ^13^C NMR: δ 171.6, 144.9 (m), 144.7, 139.6 (m), 138.0, 137.9, 128.8, 128.6 (m), 128.5, 128.4 (m), 128.01 (m), 127.98, 127.94 (m), 127.8, 127.5, 127.4, 126.8 (m), 126.3, 121.5, 121.2 (m), 73.3 (m), 73.2, 71.3, 63.7, 54.7 (m), 52.1, 50.4, 45.1 (m), 40.1 (m), 39.7, 33.2, 33.1 (m), 28.8, 27.8, 27.2 (m), 22.7, 21.9, 21.8 (m); MS (ESI^+^, *m/z*): calcd for C_26_H_34_NO_3_ [M+H]^+^ 408.25, found 408.65.

#### Acetylbenzylamino alcohol, (2*S*,5*R*)-8

Yield 86%. The ^1^H and ^13^C NMR spectra were identical to **(2*R*,5*S*)-8**. HPLC: 15.9 min, 96%, λ = 210 nm; FTIR (neat): 3422 cm^–1^ (OH), 1630 cm^–1^ (C = O), 1266 cm^–1^ (CO); HRMS (ESI^+^, *m/z*): calcd for C_26_H_34_NO_3_ [M+H]^+^ 408.2533, found 408.2542; [α]^25^_D_ –37°(*c* 0.99, CH_3_OH).

#### Acetylbenzylamino alcohol, (2*S*,5*S*)-8

Yield 78%. ^1^H NMR (400 MHz): δ 7.34–7.21 (m, 10H), 5.73 (dt, *J* = 4.8, 9.1, 0.8H), 5.18 (d, *J* = 9.5, 1H), 4.94 (dt, *J* = 5.4, 8.6, 0.2H), 4.62 (d, *J* = 17.7, 1H), 4.53 (d, *J* = 17.6, 1H), 4.39 (d, *J* = 11.9, 0.8H), 4.32 (d, *J* = 11.9, 0.8H), 4.28 (d, *J* = 11.1, 0.2H), 4.23 (d, *J* = 11.9, 0.2H), 3.69 (m, 1.6H), 3.53 (dd, *J* = 8.9, 10.1, 1H), 3.39 (dd, *J* = 4.8, 10.6, 1H), 3.31 (m, 0.4H), 3.11 (m, 1H), 3.00 (m, 0.8H), 2.78 (m, 0.2H), 2.33 (s, 0.6H), 2.28 (m, 0.2H), 2.21 (dt, *J* = 3.2, 13.6, 1H), 2.01 (s, 2.4H), 1.93 (d, *J* = 13.7, 1H), 1.78 (d, *J* = 11.7, 2H), 1.49–1.34 (m, 3H), 1.28–1.17 (m, 1H). ^13^C NMR: δ 172.8, 172.0 (m), 145.1, 145.0 (m), 139.7 (m), 138.1, 138.0 (m), 128.7, 128.4, 127.8, 127.7, 127.2, 126.9 (m), 126.2, 119.9 (m), 119.5, 73.0 (m), 72.8, 71.3 (m), 70.7, 63.36, 62.73 (m), 55.3 (m), 50.8, 48.6, 44.8 (m), 40.3 (m), 39.8, 33.3 (m), 33.0, 29.1, 28.3, 28.0 (m), 22.5, 21.7, 21.4 (m). HRMS (ESI^+^, *m/z*): calcd for C_26_H_34_NO_3_ [M+H]^+^ 408.2533, found 408.2516; calcd for C_52_H_66_N_2_O_6_Na [2M+Na]^+^ 837.4813, found 837.4767; [α]^25^_D_ +36°(*c* 0.82, CH_3_OH).

#### Acetylbenzylamino acid, (2*R*,5*S*)-9

Acetylbenzylamino alcohol **(2*R*,5*S*)-8** (266 mg, 0.653 mmol) was dissolved in acetone (36 mL) and cooled with an ice bath. A solution of 24% CrO_3_ in aq. H_2_SO_4_ (0.73 mL, 2.7 M) was added dropwise, and the mixture was stirred for 30 min. *i*-PrOH (8 mL) was added and the mixture was stirred for 30 min. Water (50 mL) was added to the solution and the mixture was extracted with CH_2_Cl_2_ (12 × 25 mL). The combined organic solution was dried with Na_2_SO_4_ and evaporated at reduced pressure. The crude product was purified by flash chromatography with 1% AcOH in EtOAc:hexanes (1:3) to give a colorless oil (246 mg, 89%). Compound **(2*R*,5*S*)-9** exists as a pair of rotamers, with a ratio of ca. 4:1 in CDCl_3._^1^H NMR: δ 7.34–7.15 (m, 10H), 5.50 (d, *J* = 9.8, 0.8H) 5.22 (m, 0.8H), 5.15 (d, *J* = 8.5, 0.2H), 4.81 (q, *J* = 7.3, 0.2H), 4.58–4.42 (m, 3.5H), 4.36 (d, *J* = 12.0, 0.2H), 4.30 (d, *J* = 12.0, 0.2H), 3.68–3.58 (m, 2.2H), 3.46 (br, 0.2H), 3.40–3.32 (m, 0.5H), 2.28 (s, 0.8H), 2.22–2.02 (m, 5.25H), 1.72–1.55 (m, 3H), 1.25–1.04 (m, 2H); HRMS (ESI^+^, *m/z*): calcd for C_26_H_32_NO_4_ [M+H]^+^ 422.2326, found 422.2332.

#### Acetylbenzylamino acid, (2*S*,5*R*)-9

Yield 77%. HPLC: 10.7 min, 98%, λ = 210 nm. The ^1^H NMR spectrum was identical to **(2*R*,5*S*)-9**. FTIR (neat): 3422 cm^–1^ (OH), 1630 cm^–1^ (C = O), 1266 cm^–1^ (CO); MS (ESI^+^, *m/z*): calcd for C_26_H_32_NO_4_ [M+H]^+^ 422.2, found 422.3; MS (ESI^–^, *m/z*): calcd for C_26_H_30_NO_4_ [M–H]^–^ 420.2, found 420.4; HRMS (ESI^+^, *m/z*): calcd for C_26_H_31_NO_4_ [M+H]^+^ 422.2326, found 422.2305; calcd for C_26_H_31_NO_4_Na^+^ [M+Na]^+^ 447.2315, found 447.2235; [α]^25^_D_ –86°(*c* 0.37, CH_3_OH).

#### Acetylbenzylamino acid, (2*S*,5*S*)-9

Yield 68%. ^1^H NMR (400 MHz): δ 7.35–7.15 (m, 10H), 5.60 (dt, *J* = 5.2, 8.3, 0.8H), 5.23 (d, *J* = 8.1, 0.8H), 5.14 (d, *J* = 7.4, 0.2H), 4.91 (d, *J* = 15.9, 0.2H), 4.85 (dt, *J* = 5.6, 8.1, 0.2H), 4.59 (d, *J* = 17.9, 0.8H), 4.51 (d, *J* = 17.9, 0.8H), 4.39 (d, *J* = 11.9, 0.8H), 4.34 (d, *J* = 12.0, 0.8H), 4.22 (d, *J* = 15.8, 0.2H), 4.18 (s, 0.4H), 3.80 (d, *J* = 3.9, 0.8H), 3.51 (dd, *J* = 7.8, 10.2, 0.8H), 3.48 (m, 0.2H), 3.41 (dd, *J* = 5.0, 10.3, 0.8H), 3.26 (dd, *J* = 5.0, 9.7, 0.2H), 3.19 (t, *J* = 9.2, 0.2H), 2.37 (s, 0.6H), 2.32–2.13 (m, 2H), 2.09–2.14 (m, 1H), 2.03 (s, 2.4H), 1.80–1.57 (m, 3H), 1.41–1.19 (m, 2H); ^13^C NMR: δ 175.2, 173.3, 173.0 (m), 143.6 (m), 143.0, 139.2 (m), 137.9, 128.8, 128.5, 127.9, 127.8 (m), 126.2, 120.9, 119.8 (m), 73.0, 71.4 (m), 70.7, 55.6 (m), 51.4, 49.0, 45.1 (m), 43.1, 42.9 (m), 34.5, 34.3 (m), 29.3, 29.2 (m), 28.0, 27.3 (m), 22.6, 22.4 (m), 22.2 (m), 22.1; HRMS (ESI^+^, *m/z*): calcd for C_26_H_32_NO_4_ [M+H]^+^ 422.2326, found 422.2329; calcd for C_52_H_62_N_2_O_8_Na [2M+Na]^+^ 865.4398, found 865.4369; [α]^22^_D_ +67°(c 0.84, CH_3_OH).

#### Acetylamino acid, (2*R*,5*S*)-10

NH_3_ (19 mL) was distilled into a flask and Na (139 mg, 6.04 mmol) was added at –78°C. A solution of acetyl-benzylamino acid, **(2*R*,5*S*)-9** (212 mg, 0.503 mmol) in dry THF (9.5 mL) was added dropwise to the Na/NH_3_ solution. The reaction was warmed to reflux for 3 h, and quenched with solid NH_4_Cl (ca. 4 g). AcOH (40 mL) was added slowly to the solution at –78°C, followed by the addition of CH_3_OH (30 mL). The mixture was filtered, and the filtrate was concentrated. The residue was purified by flash chromatography with 1% AcOH in CH_3_OH:CH_2_Cl_2_ (1:12) to give a colorless oil (84 mg, 69%). HPLC: λ = 210 nm, 9.48 min, 100%. ^1^H NMR (CD_3_OD): δ 5.21 (d, *J* = 9.0, 1H), 4.70 (dt, *J* = 5.9, 9.0, 1H), 3.76 (br, 1H), 3.54 (dd, *J* = 5.1, 11.0, 1H), 3.49 (dd, *J* = 6.6, 11.0, 1H), 2.28–2.23 (m, 2H), 2.13 (m, 1H), 1.95 (s, 3H), 1.82–1.77 (m, 1H), 1.64–1.59 (m, 2H), 1.53–1.43 (m, 1H), 1.36–1.27 (m, 1H); ^13^C NMR (CD_3_OD): δ 172.6, 142.5, 123.6, 65.2, 50.5, 35.5, 30.4, 28.9, 24.0, 22.7; MS (ESI^+^, *m/z*): calcd for C_12_H_19_NNaO_4_ [M + Na]^+^ 264.12, found 264.51.

#### Acetylamino acid, (2*S*,5*R*)-10

Yield 81%. The ^1^H NMR contained a small amount of **(2*S*,5*S*)-10.** MS (ESI^–^, *m/z*): calcd for C_12_H_18_NO_4_ [M–H]^–^ 240.1, found 240.4; [α]^23^_D_ –0.27°(*c* 0.84, CH_3_OH).

#### Acetylamino acid, (2*S*,5*S*)-10

Yield 75%. ^1^H NMR (CD_3_OD): δ 5.38 (d, *J* = 7.9, 1H), 4.74 (q, *J* = 6.6, 1H), 3.74 (br, 1H), 3.64 (dd, *J* = 5.8, 11.0, 1H), 3.58 (dd, *J* = 6.0, 11.0, 1H), 2.43–2.35 (m, 2H), 2.25 (m, 1H), 2.01 (s, 3H), 1.89 (m, 1H), 1.70–1.57 (m, 3H), 1.47–1.41 (m, 1H). ^13^C NMR: δ 172.7, 143.8, 123.3, 65.5, 50.1, 35.5, 30.7, 29.0, 24.0, 22.6. [α]^22^_D_ +180°(c 0.59, CH_3_OH).

#### NEA amide, (2*R*,5*S*)-11

Acetyl amino acid, **(2*R*,5*S*)-10** (42.0 mg, 0.174 mmol) was dissolved in DMF:CH_2_Cl_2_ (1:2, 18 mL). 2-(2-naphthyl)ethylamine (89.4 mg, 0.522 mmol), DIEA (67.5 mg, 0.522 mmol), DMAP (ca. 3 mg), HOBt (79.9 mg, 0.522 mmol) and DCC (108 mg, 0.522 mmol) were added, and the mixture was stirred for 24 h. The reaction was diluted with EtOAc (75 mL), washed with water (30 mL), HCl (1M, 30 mL), NaHCO_3_ (30 mL) and brine (30 mL), dried with Na_2_SO_4_ and concentrated. The crude product was purified by flash chromatography with CH_3_OH:CHCl_3_ (1:8) to give a colorless oil (67 mg, 98%). HPLC: λ = 254 nm, 16.0 min, 95%. ^1^H NMR: δ 7.79 (d, *J* = 7.4, 1H), 7.77 (d, *J* = 8.0, 1H), 7.76 (d, *J* = 5.7, 1H), 7.60 (s, 1H), 7.45 (dt, *J* = 1.4, 7.0, 1H), 7.42 (dt, *J* = 1.4, 7.0, 1H), 7.31 (dd, *J* = 1.6, 8.7, 1H), 6.75 (t, *J* = 5.7, 1H), 5.89 (d, *J* = 7.3, 1H), 5.13 (d, *J* = 9.5, 1H), 4.56 (m, 1H), 3.57 (quintet, *J* = 6.8, 1H), 3.55 (t, *J* = 6.6, 1H), 3.49 (quintet, *J* = 6.6, 1H), 3.43 (dd, *J* = 4.6, 10.0, 1H), 3.21 (br, 1H), 3.16 (dd, *J* = 7.6, 10.0, 1H), 2.94 (dt, *J* = 2.0, 7.0, 2H), 2.33 (d, *J* = 13.2, 1H), 1.99 (m, 2H), 1.93 (s, 3H), 1.66 (d, *J* = 12.0, 1H), 1.56–1.45 (m, 2H), 1.36–1.28 (m, 1H), 1.23 (m, 1H); ^13^C NMR: δ 172.1, 170.1, 143.3, 136.8, 133.6, 132.2, 128.2, 127.7, 127.5, 127.40, 127.38, 126.2, 125.6, 123.6, 64.9, 49.1, 43.6, 40.8, 35.7, 34.7, 28.4, 27.7, 23.4, 22.8; [α]^25^_D_ +100°(*c* 1.6, CH_3_OH).

#### NEA amide, (2*S*,5*R*)-11

Yield 61%. HPLC: λ = 210 nm, 16.2 min, 99%. The ^1^H and ^13^C NMR spectra were identical to **(2*R*,5*S*)-11**. MS (ESI^+^, *m/z*): calcd for C_24_H_31_N_2_O_3_ [M+H]^+^ 422.2, found 422.3; HRMS (ESI^+^, *m/z*): calcd for C_19_H_30_N_4_O_5_ [M+H]^+^ 395.2289, found 395.2315; calcd for C_19_H_30_N_4_O_5_Na^+^ [M+Na]^+^ 417.2108, found 417.2129; [α]^25^_D_ –100°(*c* 1.8, CH_3_OH).

#### NEA amide, (2*S*,5*S*)-11

Yield 66%. HPLC: λ = 210 nm, 16.0 min, 91%; ^1^H NMR: δ 7.79–7.74 (m, 4H), 7.62 (s, 1H), 7.42 (m, 2H), 7.36 (dd, *J* = 1.4, 8.5, 1H), 6.24 (m, 1H), 5.05 (dd, *J* = 1.5, 9.0, 1H), 4.34 (tt, *J* = 4.8, 9.2, 1H), 3.68–3.56 (m, 2H), 3.52–3.45 (m, 2H), 3.42 (d, *J* = 3.8, 1H), 3.27–3.15 (m, 1H), 3.05–2.96 (m, 2H), 2.40 (d, J = 13.6, 1H), 2.00–1.97 (m, 1H), 1.90–1.85 (m, 2H), 1.77 (s, 3H), 1.70–1.53 (m, 2H), 1.26–1.13 (m, 2H); ^13^C NMR: δ 171.8, 171.1, 142.4, 137.3, 133.7, 132.2, 127.9, 127.8, 127.7, 127.5, 127.4, 126.0, 125.3, 123.4, 64.7, 50.7, 42.7, 41.1, 35.8, 34.3, 27.4, 27.9, 23.1, 22.8; [α]^22^_D_ +97°(c 0.59, CH_3_OH); As synthesized from the epimerized **(2*S*,5*S*)-10** [α]^22^_D_ +99°(*c* 0.32, CH_3_OH).

#### Dibenzylphosphate, (2*R*,5*S*)-12

To a flask containing amide, **(2*R*,5*S*)-11**, (24.0 mg, 0.0610 mmol) and 5-ethylthio-1*H*-tetrazole (31.8 mg, 0.244 mmol) under N_2_, THF (8 mL) was added. The mixture was stirred for 5 min, and P(OBn)_2_N(*i*-Pr)_2_ (63.2 mg, 0.183 mmol) was added via syringe. The mixture was stirred for 18 h, cooled to –40°C with a CH_3_CN/dry ice bath, and *t*-BuOOH (49 μL, 5.0–6.0 M in decane) was added. The mixture was warmed slowly to rt, and the stirring was continued for 30 min. Na_2_S_2_O_5_ (15 mL) was added. The mixture was stirred for 20 min, extracted with CH_2_Cl_2_ (4 × 20 mL), dried with Na_2_SO_4_ and evaporated at reduced pressure. The crude product was purified by flash chromatography with EtOAc to give **(2*R*,5*S*)-12** (24.5 mg, 62%) as a colorless oil. ^1^H NMR: δ 7.75 (dd, *J* = 1.2, 7.1, 1H), 7.74 (d, *J* = 8.2, 2H), 7.59 (s, 1H), 7.42–7.29 (m, 13H), 6.10 (t, *J* = 5.6, 1H), 5.87 (d, *J* = 7.9, 1H), 5.09 (dd, *J* = 1.4, 9.0, 1H), 5.02 (dd, *J* = 5.2, 11.6, 1H), 5.00 (dd, *J* = 5.4, 11.7, 1H), 4.98 (dd, *J* = 5.4, 11.6, 1H), 4.97 (dd, *J* = 5.1, 11.7, 1H), 4.66 (app. septet, *J* = 4.2 Hz, 1H), 3.70 (ddd, *J* = 4.2, 8.5, 10.7, 1H), 3.67 (ddd, *J* = 4.6, 9.8, 10.4, 1H), 3.55 (tt, *J* = 6.8, 13.4, 1H), 3.51 (tt, *J* = 6.5, 12.8, 1H), 6.92 (d, *J* = 3.8, 1H), 2.96 (t, *J* = 7.0, 2H), 2.33 (d, *J* = 13.1, 1H), 2.03–1.90 (m, 2H), 1.87 (s, 3H), 1.65 (d, *J* = 12.1, 1H), 1.54–1.51 (m, 1H), 1.47 (app. tq, *J* = 3.6, 13.1, 1H), 1.31 (app. tt, *J* = 4.8, 13.1, 1H), 1.20 (app. tq, *J* = 4.1, 12.7, 1H); ^13^C NMR: δ 171.6, 169.4, 143.8, 136.8, 132.8, 131.9, 131.1, 129.0, 128.8, 128.3, 128.19, 128.18, 127.7, 127.6, 127.43, 127.40, 126.2, 125.5, 121.9, 77.4, 69.81, 69.77, 46.9, 44.0, 40.7, 35.7, 34.5, 28.9, 27.7, 23.4, 22.7; ^31^P NMR: δ 0.63; MS (ESI^+^, *m/z*): calcd for C_38_H_44_N_2_O_6_P [M+H]^+^ 655.29, found 655.98; [α]^25^_D_ +84°(*c* 0.57, CH_3_OH).

#### Dibenzylphosphate, (2*S*,5*R*)-12

Yield 58%. The ^1^H and ^13^C NMR spectra were identical to **(2*R*,5*S*)-12**. MS (ESI^+^, *m/z*): calcd for C_38_H_44_N_2_O_6_P [M+H]^+^ 655.3, found 655.4; [α]^25^_D_ –79°(*c* 0.82, CH_3_OH).

#### Dibenzylphosphate, (2*S*,5*S*)-12

Yield 47%. HPLC: λ = 254 nm, 20.8 min, 96%; ^1^H NMR δ 7.78–7.72 (m, 3H), 7.65 (t, *J* = 5.6, 1H), 7.61 (s, 1H), 7.43–7.33 (m, 13H), 6.70 (d, *J* = 5.3, 1H), 5.10–5.00 (m, 4H), 4.92 (d, *J* = 9.2, 1H), 4.53–4.48 (m, 1H), 3.88–3.78 (m, 2H), 3.67 (dq, *J* = 6.9, 13.6, 1H), 3.47 (ddt, *J* = 5.4, 7.2, 13.2, 1H), 3.34 (d, *J* = 3.5, 1H), 3.00 (t, *J* = 7.4, 2H), 2.42 (d, *J* = 12.7, 1H), 1.94–1.84 (m, 2H), 1.74 (s, 3H), 1.71–1.68 (m, 2H), 1.55–1.53 (m, 1H), 1.16–1.09 (m, 2H); ^13^C NMR: δ 171.1, 170.7, 137.4, 144.1, 135.5, 135.3, 133.6, 132.2, 129.08, 129.06, 128.9, 128.24, 128.22, 127.86, 127.82, 127.69, 127.56, 127.3, 125.9, 125.2, 121.2, 77.4, 70.1, 70.0, 68.56, 68.51, 48.74, 48.71, 42.68, 41.01, 35.9, 34.4, 28.6, 28.1, 23.0, 22.7; [α]^25^_D_ +98°(*c* 0.61, CH_3_OH).

#### Phosphate, (2*R*,5*S*)-1

Dibenzylphosphate, **(2*R*,5*S*)-12**, (22 mg, 0.034 mmol) was dissolved in *i*Pr_3_SiH:H_2_O:TFA (2.5:2.5:95, 5 mL), and the mixture was stirred for 3 h. The reaction solution was concentrated, and the residue was purified by HPLC on a Waters XBridge C18 5 μm 19 × 100 mm column, gradient 0% to 100% CH_3_CN/H_2_O over 12 min, 100% CH_3_CN for 4 min, at 12 mL/min, λ = 254 nm, to give a white solid (5.3 mg, 33%). (Note: Higher yields may be obtained by neutralizing the TFA of the reaction, and eliminating the use of TFA in the mobile phase.) HPLC with 0.1% TFA in the mobile phase: 14.5 min, 99%; ^1^H NMR (CD_3_OD): δ 7.68 (m, 3H), 7.59 (s, 1H), 7.31 (m, 3H), 5.21 (br, 1H), 4.66 (br, 1H), 3.71 (br, 2H), 3.52 (br, 2H), 3.44 (br, 1H), 2.91 (br, 2H), 2.14 (d, *J* = 9.2, 1H), 1.83 (m, 4H), 1.48–1.08 (m, 6H); ^13^C NMR (CD_3_OD): δ 174.7, 172.3, 142.2, 138.3, 135.1, 133.7, 129.0, 128.9, 128.6, 128.45, 128.38, 126.8, 126.2, 125.6, 67.6, 44.7, 44.6, 41.8, 36.6, 35.3, 29.8, 28.7, 23.8, 22.7; ^31^P NMR (CD_3_OD): δ 0.2 (br, overlapped with external H_3_PO_4_ standard); MS (ESI^+^, *m/z*): calcd for C_24_H_32_N_2_O_6_P [M+H]^+^ 475.2, found 475.2; HRMS (ESI^–^, *m/z*): calcd for C_24_H_30_N_2_O_6_P [M–H]^–^ 473.1842, found 473.1837; [α]^25^_D_ +62°(*c* 0.12, CH_3_OH).

#### Phosphate, (2*S*,5*R*)-1

Yield 38%. HPLC with 0.1% TFA in the mobile phase: 14.5 min, 95%; The ^1^H, ^13^C, and ^31^P NMR spectra were identical to **(2*R*,5*S*)-1**; MS (ESI^+^, *m/z*): calcd for C_24_H_32_N_2_O_6_P [M+H]^+^ 475.2, found 475.2; [α]^25^_D_ –61°(*c* 0.22, CH_3_OH).

#### Phosphate, (2*S*,5*S*)-1

Yield 54%. HPLC: λ = 254 nm, 20.8 min, 96%. ^1^H NMR (CD_3_OD): δ 7.80 (d, *J* = 8.1, 1H), 7.76 (d, *J* = 8.1, 2H), 7.62 (s, 1H), 7.44 (t, *J* = 6.8, 1H), 7.40 (t, *J* = 6.8, 1H), 7.36 (d, *J* = 8.4, 1H), 5.09 (d, *J* = 8.3, 1H), 4.62 (br, 1H), 3.84 (br, 2H), 3.66 (dt, *J* = 7.2, 13.1, 1H), 3.56 (br, 1H), 3.42 (app. dt, *J* = 6.8, 13.3, 1H), 2.97 (t, *J* = 6.8, 2H), 2.31 (d, *J* = 13.1, 1H), 1.94–1.91 (m, 1H), 1.80 (m, 1H), 1.78 (s, 3H), 1.63–1.51 (m, 3H), 1.36–1.31 (m, 1H), 1.24–1.16 (m, 1H); ^13^C NMR: 171.1, 169.8, 140.7, 137.3, 133.1, 131.6, 127.6, 127.5, 127.4, 127.3, 126.7, 125.9, 125.2, 123.8, 48.3, 41.8, 35.2, 33.6, 30.7, 27.9, 27.4, 22.5, 22.3; ^31^P NMR (DMSO-*d*_6_): δ 1 (br, overlapped with external H_3_PO_4_ standard); [α]^25^_D_ +180°(*c* 0.14, CH_3_OH).

#### Oxazolidinones, (2*R*,3*S*)-13 and (2*R*,3*R*)-13

A mixture of allyl alcohol **(2*R*,3*S*)-3** and **(2*R*,3*R*)-3** (22 mg, 0.059 mmol) was dissolved in THF (3 mL). KH (3.5 mg, 0.088 mmol) was added, and the mixture was stirred for 1 h. The reaction was quenched with CH_3_OH, diluted with NH_4_Cl (15 mL) and extracted with EtOAc (15 mL). The organic solution was washed with brine, dried with Na_2_SO_4_, and evaporated under reduced pressure. The crude product was purified by flash chromatography with EtOAc:hexanes (1:4), followed by EtOAc:hexanes (1:3) to give a colorless oil as a mixture of two diastereomers, (13 mg, 81%). ^1^H NMR: δ 7.38–7.26 (m, 5H), 5.84 (m, 0.87H), 5.76 (m, 0.13H), 5.30 (s, 0.87H), 5.24 (s, 0.13H), 4.96 (d, *J* = 7.7, 0.87H), 4.55 (s, 0.26H), 4.51 (d, *J* = 5.5, 0.13H), 4.49 (s, 1.74H), 4.02 (m, 0.87H), 3.78 (m, 0.13H), 3.51 (dd, *J* = 4.0, 9.3, 0.13H), 3.45 (dd, *J* = 7.7, 9.3, 0.13H), 3.39 (dd, *J* = 3.7, 9.3, 0.87H), 3.34 (app t, *J* = 9.3, 0.87H), 2.06 (m, 2H), 1.95–1.86 (m, 2H), 1.69–1.50 (m, 4H).

#### Oxazolidinones, (2*S*,3*R*)-13 and (2*S*,3*S*)-13

The ^1^H spectrum was identical to **(2*R*,3*S*)-13** and **(2*R*,3*R*)-13**.

#### Bicyclic alkene, (2*S*,5*R*)-14

The (*Z*)-alkene, **(2*S*,5*R*)-6**, (34 mg, 0.075 mmol) was dissolved in CH_2_Cl_2_, and the solution was cooled with an ice bath. Pyridine (88 mg, 1.1 mmol) was added, followed by the addition of CH_3_SO_2_Cl (100 mg, 0.90 mmol). The mixture was stirred and warmed slowly to rt. The stirring was continued for 16 h. The reaction was washed with H_2_O, dried with Na_2_SO_4_ and evaporated to give (*S*,*R*)-*Z*-alkene methylsulfonate as a colorless oil (29 mg, 73%). The crude product was used for next step without further purification. Pd(OH)_2_/C (2.5 mg, 20%) was added to a flask containing (*S*,*R*)-*Z*-alkene methylsulfonate (18 mg, 0.034 mmol). CH_3_OH (2 mL) was added, followed by the addition of HCOOH (235 mg, 5.10 mmol). The mixture was stirred and monitored by TLC. When the reaction was complete, the mixture was filtered through Celite immediately and washed with CH_3_OH (25 mL). The filtrate was concentrated at reduced pressure, and the residue was neutralized with NaHCO_3_ and extracted with CH_2_Cl_2_ (2 × 25 mL). The combined organic solution was dried with Na_2_SO_4_ and evaporated. The crude product was purified by flash chromatography with EtOAc:hexanes (1:12) to give **(2*S*,5*R*)-14** (9.3 mg, 79%). ^1^H NMR: δ 7.35–7.21 (m, 10H), 5.36 (m, 1H), 4.57 (d, *J* = 12.1, 1H), 4.53 (d, *J* = 12.1, 1H), 4.18 (d, *J* = 13.8, 1H), 3.72 (dd, *J* = 4.6, 9.6, 1H), 3.46 (dd, *J* = 5.8, 9.6, 1H), 3.24 (d, *J* = 13.8, 1H), 3.15 (br, 1H), 2.83 (dd, *J* = 5.6, 11.1, 1H), 2.36–2.23 (m, 1H), 2.13 (br, 1H), 2.04–1.96 (m, 1H), 1.87 (dd, *J* = 9.5, 11.2, 1H), 1.76–1.67 (m, 2H), 1.64–1.59 (m, 2H), 1.34–1.13 (m, 2H), 0.87 (dq, *J* = 3.5, 12.5, 1H); ^13^C NMR: δ 140.3, 139.6, 138.5, 129.1, 128.5, 128.3, 127.8, 127.7, 126.9, 120.2, 73.8, 73.4, 60.8, 59.4, 56.4, 37.0, 34.0, 31.8, 27.5, 25.5; HRMS (ESI^+^, *m/z*): calcd. for C_24_H_30_NO [M+H]^+^ 348.2322, found 348.2329.

#### Bicyclic alkene, (2*R*,5*S*)-14

The ^1^H and ^13^C NMR spectra were identical to **(2*S*,5*R*)-14**.

#### Bicyclic alkene, (2*S*,5*S*)-14

Yield 6.2 mg, 79%. ^1^H NMR: δ 7.39–7.21 (m, 10H), 5.36 (d, *J* = 1.8, 1H), 4.53 (d, *J* = 12.2, 1H), 4.49 (d, *J* = 12.2, 1H), 3.77 (s, 2H), 3.66 (dd, *J* = 6.8, 10.0, 1H), 3.42 (dd, *J* = 5.1, 10.0, 1H), 3.31 (br, 1H), 2.70 (dd, *J* = 5.6, 12.8, 1H), 2.54 (dd, *J* = 9.2, 12.8, 1H), 2.22 (d, *J* = 13.5, 1H), 2.14 (br, 1H), 2.02 (t, *J* = 12.3, 1H), 1.76 (t, *J* = 12.2, 2H), 1.64 (d, *J* = 12.3, 1H), 1.36 (tq, *J* = 3.2, 13.2, 1H), 1.24 (tq, *J* = 3.7, 13.1, 1H), 0.93 (dq, *J* = 3.3, 12.5, 1H); ^13^C NMR: δ 141.1, 140.2, 138.8, 128.8, 128.4, 128.3, 127.6, 127.5, 126.8, 118.6, 73.1, 72.1, 58.6, 58.3, 51.8, 34.8, 33.8, 32.2, 27.8, 26.0; HRMS (ESI^+^, *m/z*): calcd. for C_24_H_30_NO [M+H]^+^ 348.2322, found 348.2330.

### Bioassay

#### IC_50_ Determination of Inhibitors (2*R*,5*S*)-1, (2*S*,5*R*)-1, and (2*S*,5*S*)-1

The concentrations of the inhibitor stock solutions in DMSO:H_2_O (2:1) were determined by UV at 286 nm (log ξ = 3.59 for the naphthyl group). The assays were performed as published.[11] The stock solution was diluted to prepare final concentrations of: 0.45, 1.8, 7.3, 29, 58, 116, 232 and 465 μM for **(2*R*,5*S*)-1**; 6.0, 12, 24, 48, 96, 191 and 382 μM for **(2*S*,5*R*)-1**; and 6.0, 12, 24, 47, 94, 113, 189 and 378 μM for **(2*S*,5*S*)-1**. The assay was performed in duplicate for each concentration. The inhibitors were pre-equilibrated in a cuvette at 4°C for 12 min. The concentration of released *p*NA was recorded by UV at 390 nm for 90 s. The % inhibitions were plotted against log [I] with Table Curve, Version 3. The IC_50_ value of an inhibitor was calculated as the concentration of the inhibitor at 50% inhibition derived from the fitted equation (S2 Dataset).

## Results and Discussion

Ac–D-pSer–Ψ[(*Z*)CH = C]–L-Pip–2-(2-naphthyl)ethylamine (NEA), **(2*S*,5*R*)-1**, with a D-pSer, a cis-locked alkene, a 6-membered ring, and a naphthyl side chain, was designed as an inhibitor for Pin1 (Fig 1). The D-pSer–Ψ[(*Z*)CH = C]–L-Pip core mimics the D-pSer–L-Pip, and the NEA group mimics the Nal in Fischer’s peptides.[16] An acetyl group was attached to the Ser analogue to avoid a charged terminus; no further *N*-terminal residues were included because the *N*-terminal residues were disordered in electron density maps of several Pin1-inhibitor complexes.[13,17] As a test case, the enantiomeric **(2*R*,5*S*)-1** was synthesized first as a model compound (Fig 1). The desired enantiomer **(2*S*,5*R*)-1**, with the more expensive Boc–D-Ser(Bn)–OH as the starting material, was then synthesized (Fig A in S1 Dataset). The **(2*S*,5*S*)-1** diastereomer arose from an accidental epimerization during work-up after ammonia debenzylation.

**Fig 2:**
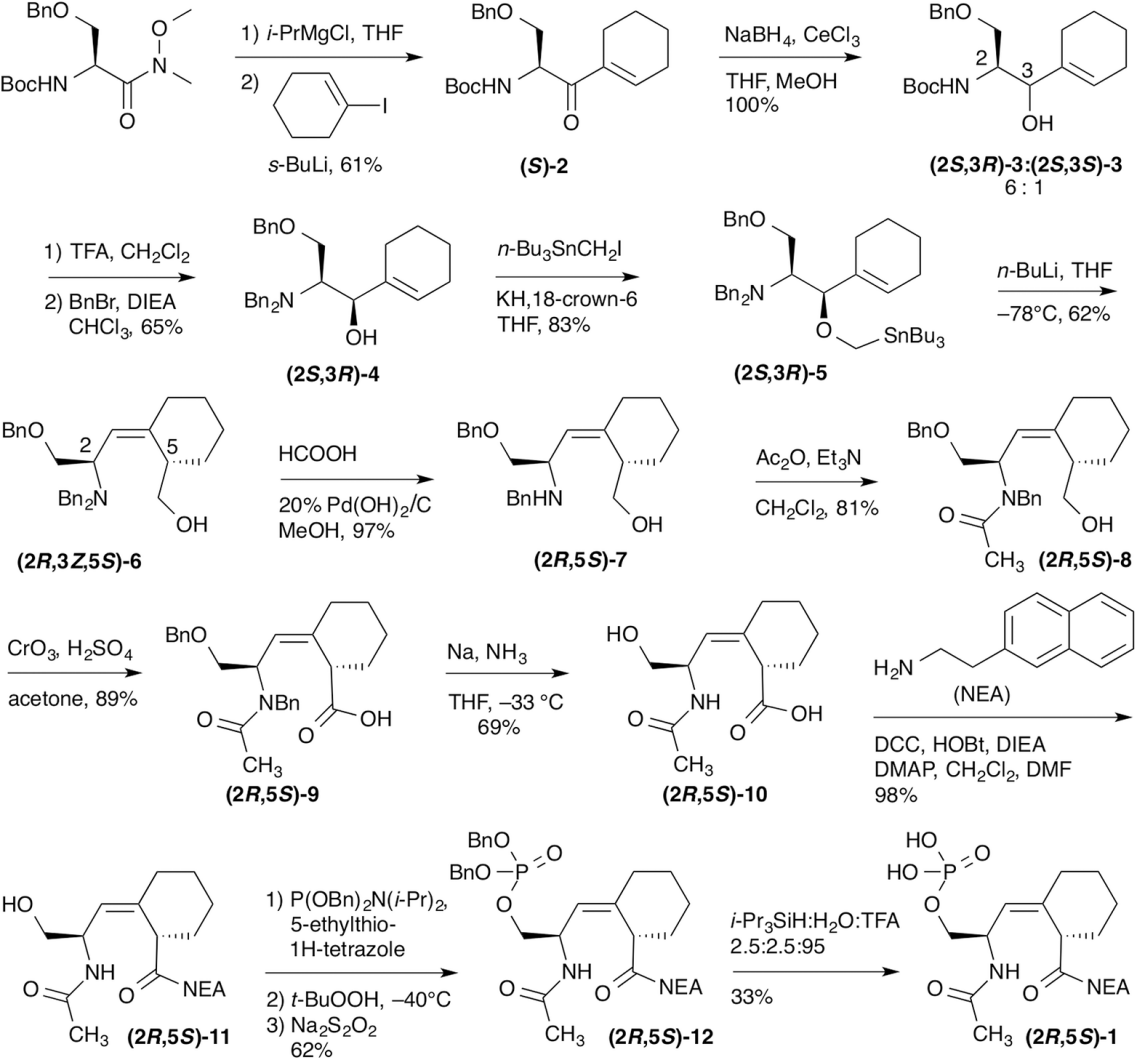
Synthesis of the Ac–L-pSer–Ψ[(*Z*)CH = C]-D-Pip–NEA inhibitor (2*R*,5*S*)-1.

**Fig 3:**
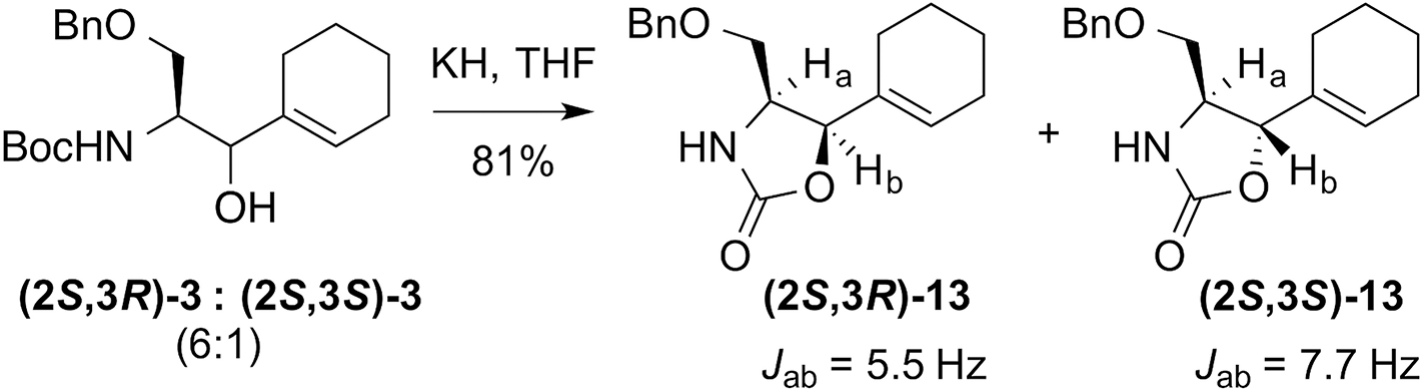
Synthesis and determination of the stereochemistry of derivative (2*S*,3*R*)-13. Cyclic compounds **13** were synthesized. ^1^H NMR coupling constants (*J*) were used to determine the relative stereochemistry at the carbons to which H_a_ and H_b_ are attached.

In the synthesis of **(2*R*,5*S*)-1**, Luche reduction was used to set up for the synthesis of the D-Pro mimic in the key Still-Wittig rearrangement (Fig 2). We were not certain if the initial Ser stereochemistry would affect the outcome of the Still-Wittig rearrangement. It did not––the stereochemistry depended only upon the stereochemistry of the allylic alcohol resulting from the Luche reduction.[10,22] The Weinreb amide of Boc–L-Ser(OBn)–OH was synthesized as reported.[24,27] 1-Iodocyclohexene was prepared from cyclohexanone by the method of Barton.[25] Nucleophilic addition of cyclohexenyl lithium, prepared from 1-iodocyclohexene *in situ*, to the Weinreb amide afforded the new cyclohexenyl ketone **(*S*)-2** (Fig 2). Luche reduction of **(*S*)-2** gave two inseparable diastereomers **(2*S*,3*R*)-3** and **(2*S*,3*S*)-3** in a ratio of 6-to–1. The stereochemistry and the ratio of the intermediate alcohols **(2*S*,3*R*)-3** and **(2*S*,3*S*)-3** from the Luche reduction[22] was determined by 1D coupling constants of cyclic oxazolidinone derivatives **(2*S*,3*R*)-13** and **(2*S*,3*S*)-13** (Fig 3). The yield and diastereoselectivity were comparable to the Luche reduction step in the synthesis of the (*E*)-alkene 5-membered ring analogue.[10]

**Fig 4:**
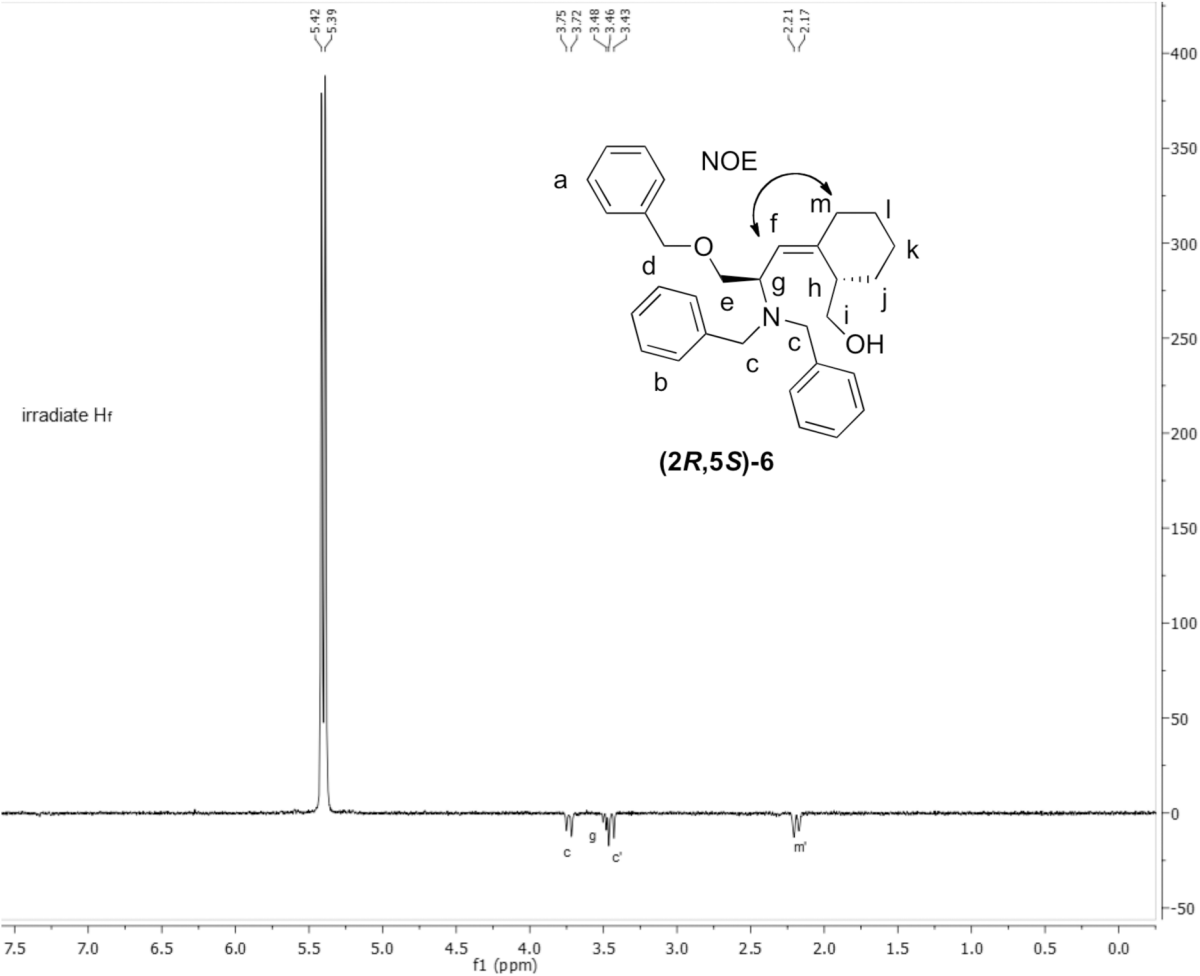
Determination of the (*Z*)-alkene stereochemistry of intermediate (2*R*,5*S*)-6. The 1D nOe ^1^H NMR. Irradiation of ^1^H_f_ shows an nOe at ^1^H_m_ and not at ^1^H_h_.

Without separation, the mixture was converted to the dibenzyl protected amine, and the desired single diastereomer, **(2*S*,3*R*)-4**, was isolated by chromatography. The bulky dibenzyl amine was necessary to obtain high stereoselectivity in the Still-Wittig rearrangement. The precursor for the Still-Wittig rearrangement, **(2*S*,3*R*)-5**, was synthesized by treating **(2*S*,3*R*)-4** with *n*-Bu_3_SnCH_2_I, prepared as previously reported (Fig 2).[26] In the presence of *n*-BuLi at –78°C, Still-Wittig rearrangement of **(2*S*,3*R*)-5** afforded the (*Z*)-alkene **(2*R*,3*Z*,5*S*)-6** as the major product, with a (*Z*):(*E*) ratio of 5.5:1. The alkene geometries were determined by 1D nuclear Overhauser effect (nOe) spectra (Fig 4). The desired stereoselectivity was higher than that obtained with the Ser−Pro (3:1), or the Ala−Pro (2:1), alkene isosteres, probably due to the greater bulk of the 6-membered ring.[10,19] The (3*R*)-alcohol stereocenter from the Luche reduction was successfully transferred to the (5*S*)-cyclohexyl stereocenter. The stereoselectivity in the rearrangements was not affected by the stereocenter at the original Ser α-carbon.

**Fig 5:**
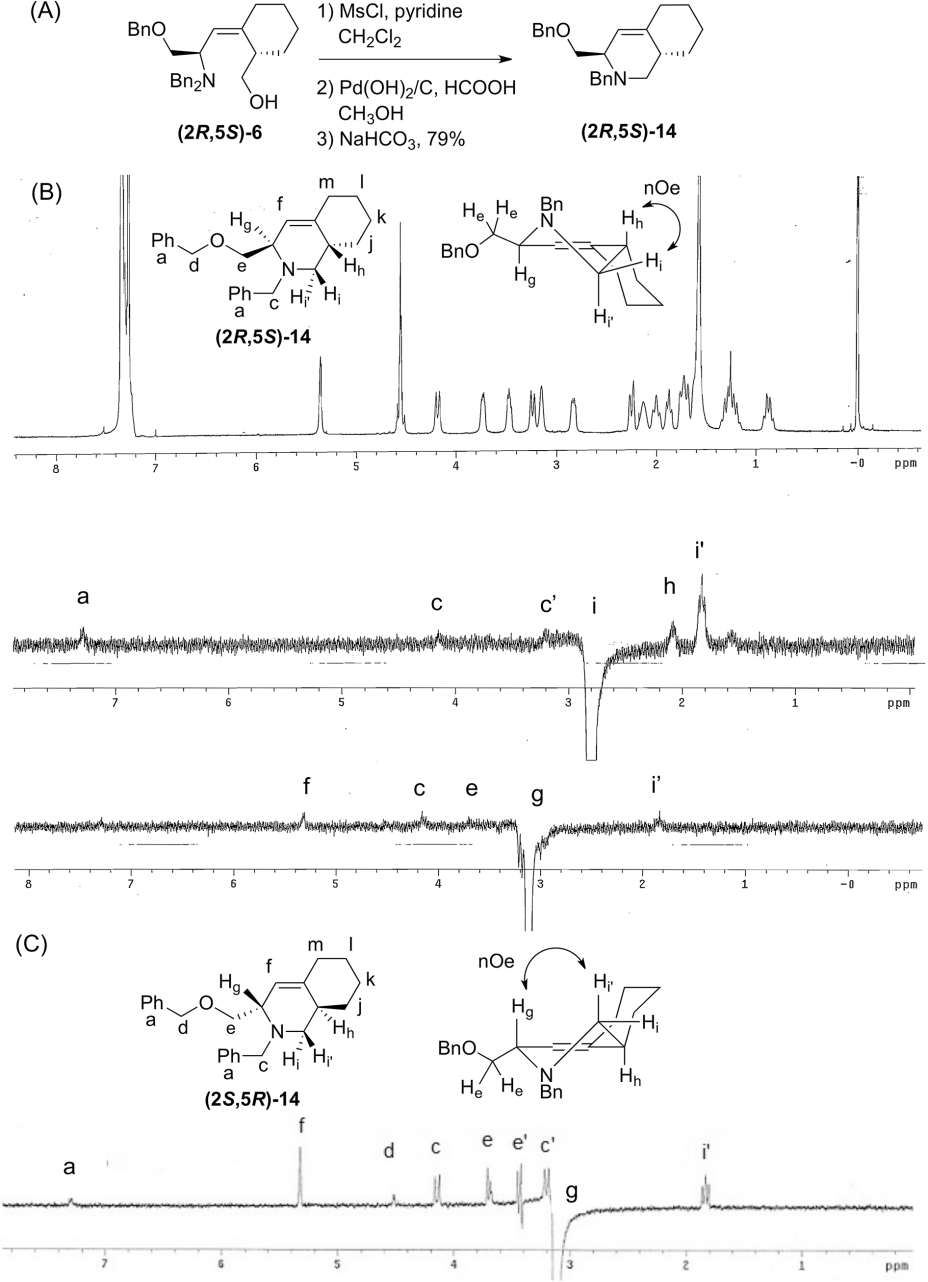
Determination of the stereochemistry of Still-Wittig intermediates 6. (A) Compound **(2*S*,5*R*)-14** was synthesized to rigidify intermediate **(2*R*,5*S*)-6** for nOe determination. (B) Structure of **(2*S*,5*R*)-14** with lettering of the protons, structure of the major conformation showing nOe interactions, the ^1^H NMR and 1D nOe spectra in CDCl_3_ (400 MHz) are shown. Irradiation of H_i_ shows an nOe at H_h_. Irradiation of H_g_ shows an nOe at H_i’_. (C) The stereochemistry of enantiomer **(2*R*,5*S*)-14** was determined. Structure with lettering of the protons, structure of the major conformation showing nOe interactions, and a 1D nOe spectrum in CDCl_3_ (400 MHz) are shown. Irradiation of H_g_ shows an nOe at H_i’_.

The relative stereochemistry in the cyclohexyl rings of **(2*R*,5*S*)-6** formed in the Still-Wittig rearrangement, and the stereochemistry of the enantiomer **(2*S*,5*R*)-6**, were determined by 1D nOe in bicyclic derivatives **(2*R*,5*S*)-14** and **(2*S*,5*R*)-14** (Fig 5). Bicyclic derivative **(2*R*,5*S*)-14** was synthesized in three steps without purification of intermediates. The primary alcohol was converted to the mesylate, one benzyl of the amine was deprotected, and NaHCO_3_ was used as base to cyclize the amine (Fig 5A). The enantiomer **(2*S*,5*R*)-14** was prepared the same way. For both derivatives, the ^1^H NMR coupling constants between H_i_-H_h_ and H_i’_-H_h_ were 5.6 Hz and 9.5 Hz, respectively, which showed H_i_ was syn to H_h_, and H_i’_ was anti to H_h_. The nOe correlations H_i—_H_h_ and H_g—_H_i’_ of **(2*R*,5*S*)-14** demonstrated that H_h_ is anti to H_g_ (Fig 5B). For **(2*S*,5*R*)-14,** the nOe H_g_—H_i’_ also showed that H_h_ is anti to H_g_ (Fig 5C). Thus, the relative relationship between H_g_ and H_h_ was shown to be anti in both enantiomers, confirming the assigned relative stereochemistry.

One *N*-benzyl protecting group of **(2*R*,5*S*)-6** was selectively removed with formic acid with 20% Pd(OH)_2_/C catalyst (Fig 2). Selective acylation of the amino group of **(2*R*,5*S*)-7** with acetic anhydride gave **(2*R*,5*S*)-8**, without affecting the primary hydroxyl group. The primary hydroxyl was converted to the carboxylic acid with Jones reagent to afford **(2*R*,5*S*)-9**. The remaining *N-* and *O-*benzyl protecting groups were removed in one step with Na/NH_3_ to give **(2*R*,5*S*)-10** (Fig 2).

**Fig 6:**
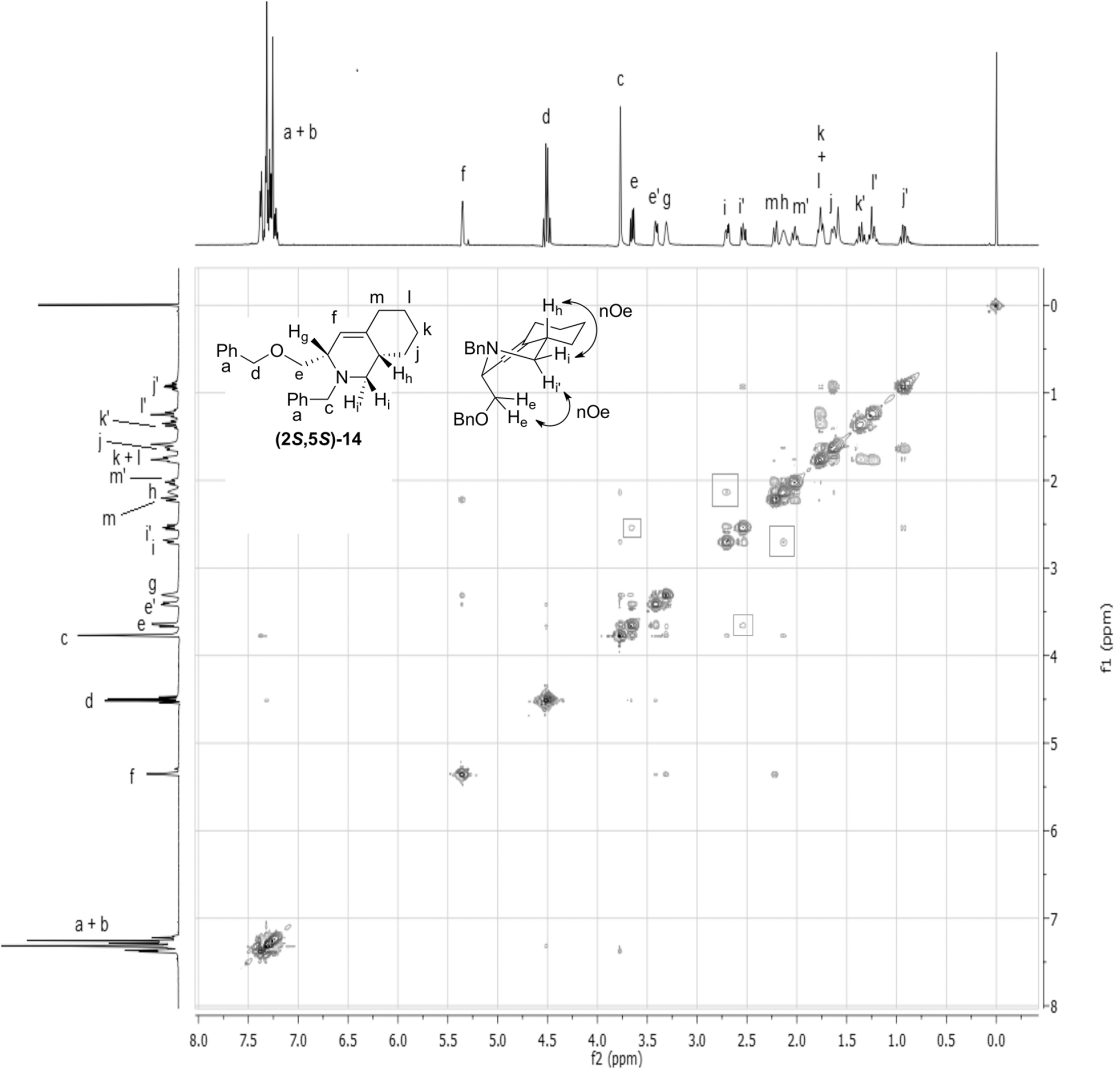
Determination of the stereochemistry of of (2*S*,5*S*)-14. The 2D NOESY spectrum is shown with lettering of the major conformation showing key nOe interactions. Crosspeaks between H_i_ and H_h_, and between H_e_ and H_i’_ show the stereochemistry given.

Partial epimerization occurred during the Na/NH_3_ deprotection of **(2*R*,5*S*)-9** to produce **(2*S*,5*S*)-10**, which was used to synthesize **(2*S*,5*S*)-1**. To determine which stereocenter was epimerized, **(2*R*,3*R*)-4** was used to resynthesize **(2*S*,5*S*)-11** (Fig B in S1 Dataset), and the optical rotations were compared (Materials and Methods). The 2D nuclear Overhauser effect spectroscopy (NOESY) of derivative **(2*S*,5*S*)-14** was used to determine the relative stereochemistry of the Still-Wittig rearrangement product **(2*S*,5*S*)-6** (Fig 6). The ^1^H NMR coupling constants between H_i—_H_h_ and H_i’—_H_h_ were 5.6 Hz and 9.2 Hz, respectively, which indicated that on this 6-membered ring, H_i_ and H_h_ were syn to each other, while H_i’_ and H_h_ were anti to each other. In the NOESY spectrum of **(2*S*,5*S*)-14**, the nOe correlation H_e_—H_i’_ indicated that the CH_2_OBn group and H_i’_ were syn to each other (Fig 6). The NOESY correlation H_i_—H_h_ indicated that H_i_ and H_h_ were syn to each other. Therefore the relative position of H_h_ and the CH_2_OBn group was confirmed to be anti, and the configuration of the stereogenic center in the 6-membered ring was determined to be (*S*).

Enzymatic assays to evaluate inhibition of Pin1 by the three stereoisomeric target compounds were performed by the protease-coupled method as previously reported.[11] The results of bioassay showed that all three stereoisomers were poor inhibitors of Pin1. The IC_50_ value of **(2*R*,5*S*)-1** was 52 ± 4 μM (Fig A in S2 Dataset), the IC_50_ value of **(2*S*,5*R*)-1** was 85 ± 10 μM (Fig B in S2 Dataset), and the IC_50_ value of **(2*S*,5*S*)-1** was 140 ± 20 μM (Fig C in S2 Dataset). We note that these alkene isosteres are ground-state analogues of the Pin1 substrate. Compound **(2*R*,5*S*)-1**, which mimics the L-pSer–D-Pip peptide, initially synthesized as a model compound, was the best inhibitor among them. This was surprising since the most potent peptidic Pin1 inhibitor has the D-pThr–L-Pip stereochemistry.[16] Compound **(2*S*,5*R*)-1**, which mimics D-pSer–L-Pip, gave slightly weaker inhibition, and **(2*S*,5*S*)-1**, which mimics D-pSer–D-Pip, was the weakest inhibitor. The relatively small differences in the inhibition implied that the stereochemistry of the Ser or Pro of these (*Z*)-alkene isosteres affects Pin1 inhibition only slightly––Pin1 can accommodate a variety of stereoisomers in its active site.

In the Zhang crystal structures, the two pentapeptide inhibitors, D-peptide and L-peptide, bound to Pin1 in approximately trans (ω angle = 183°) and cis (ω angle = −19°) conformations, respectively.[17] In our crystal structure of the Pin1 complex with the (*Z*)-alkene pentapeptide, the phosphate and the 5-membered ring of the inhibitor were found to bind to the same sites of Pin1 as the Zhang L-peptide.[13,17] Our (*Z*)-alkene pentapeptide inhibitor with both natural L-stereocenters, was 65-fold less potent than the Zhang L-peptide.[17] Upon changing Pro to Pip in Fischer’s peptide series, the IC_50_ value improved by 100-fold.[16] So, we thought that the 6-membered ring analogues of Pip could significantly improve the inhibitory activity. However, this was not the case; inhibition was worse than any of our previous (*Z*)-alkene isosteres,[11,12] probably because the D-peptide binds to Pin1 in the trans conformation.[17]

## Conclusions

The Still-Wittig [2,3]-sigmatropic rearrangement has proven to be a reliable method to synthesize Xaa–Pro and Ser–Pip alkene isosteres, predictably achieving the desired (*Z*) geometry of the double bond and the stereogenic center in the 6-membered ring. We developed practical methods to determine the configurations of the newly formed stereogenic centers in the Luche reduction, and in the [2,3]-Still-Wittig rearrangement. None of our final compounds were potent Pin1 inhibitors. We conclude that the (*S*)-configuration, at either D-Ser or D-Pro mimic site, were not optimal in any combination in the cyclohexyl (*Z*)-alkene inhibitors. Our analysis leads us to suggest that the inhibitory activity could be improved by using either D-pSer–[(*E*)CH = C]–L-Pip or L-pSer–[(*Z*)CH = C]–L-Pip as core structures.

## Supporting Information

S1 Dataset Fig A. Synthesis of (2*S*,5*R*)-1. Fig B. Synthesis of (2*S*,5*S*)-1. Fig C. NMR and IR spectra, HPLC chromatograms for compounds 1–14. (PDF)

S2 Dataset Pin1 inhibition plots for (2*R*,5*S*)-1, (2*S*,5*R*)-1, and (2*S*,5*S*)-1. Fig A. Inhibition of Pin1 by (2*R*,5*S*)-1, IC_50_ = 52 ± 4 μM. Fig B. Inhibition of Pin1 by (2*S*,5*R*)-1, IC_50_ = 85 ± 10 μM. Fig C. Inhibition of Pin1 by (2*S*,5*S*)-1, IC_50_ = 140 ± 20 μM.
(PDF)
