# Supplementary material for: Stereochemical Control in the Still-Wittig Rearrangement Synthesis of Cyclohexyl (Z)-Alkene Inhibitors of Pin1
Source: PLoS One. 2015 Oct 7;10(10):e0139543. doi: 10.1371/journal.pone.0139543 (PMC4596862; doi:10.1371/journal.pone.0139543)
Supplement: S1 Dataset — (PDF) [file pone.0139543.s001.pdf]

Electronic Supporting Information: S1 Dataset

Stereochemical control in the Still-Wittig rearrangement synthesis of cyclohexyl (*Z*)-alkene inhibitors of Pin1

Xingguo R. Chen, Shuang A. Fan, Rachel I. Ware, and Felicia A. Etzkorn*

Department of Chemistry, Virginia Tech, Blacksburg, Virginia 24061

*Corresponding author
E-mail: fetzkorn@vt.edu

Table of Contents **Page**

Fig A. Synthesis of (2*S*,5*R*)-1 1
Fig B. Synthesis of (2*S*,5*S*)-1 2
Fig C. NMR and IR spectra, HPLC chromatograms for compounds 1 – 14 3

Fig A in S1 Dataset. Synthesis of (2*S*,5*R*)-1.

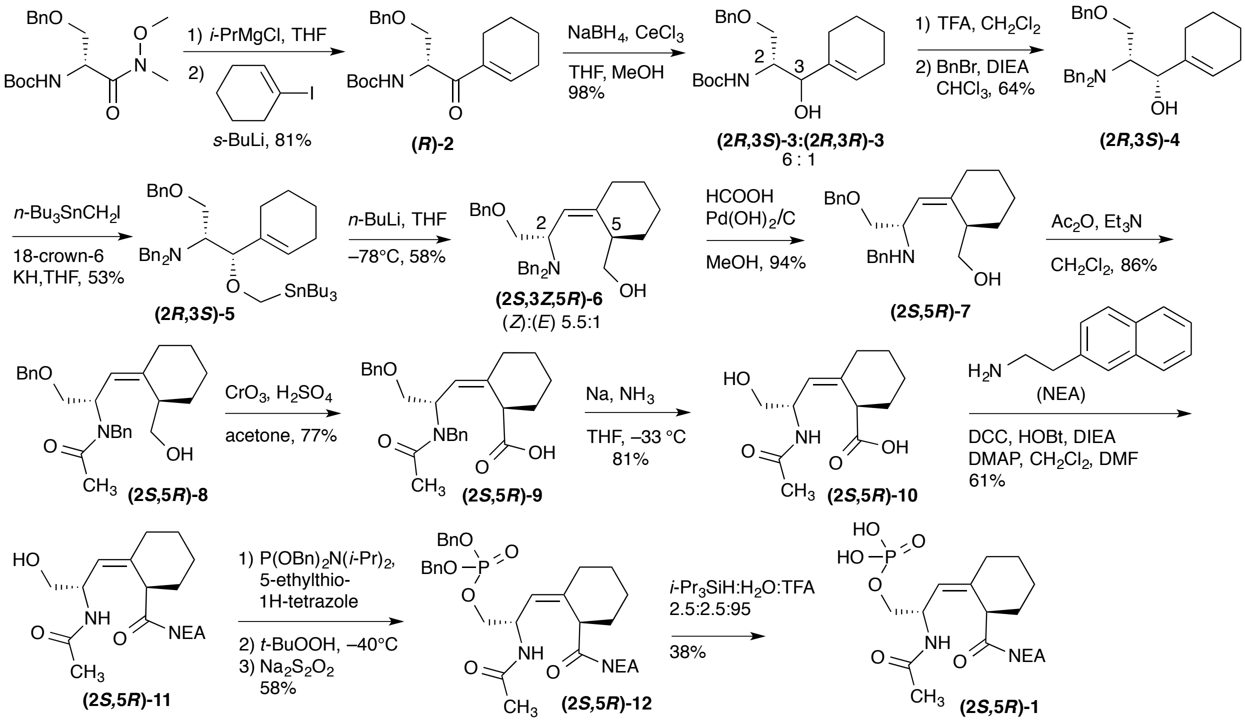

Fig A in S1 Dataset. Synthesis of (2*S*,5*S*)-1.

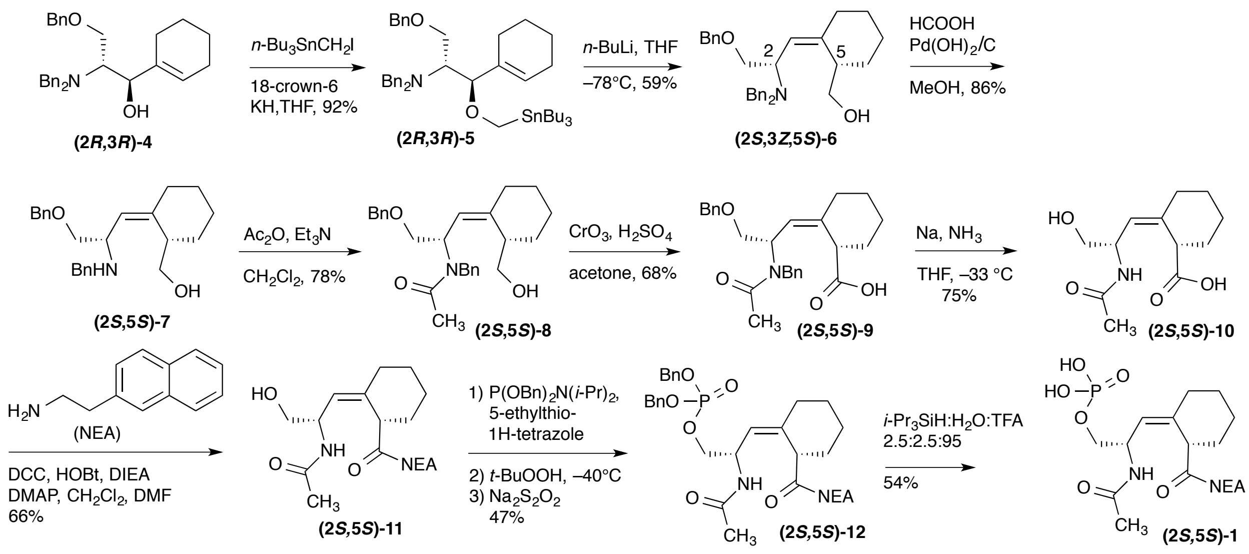

Fig C in S1 Dataset. NMR and IR spectra, HPLC chromatograms for compounds 1 – 14.

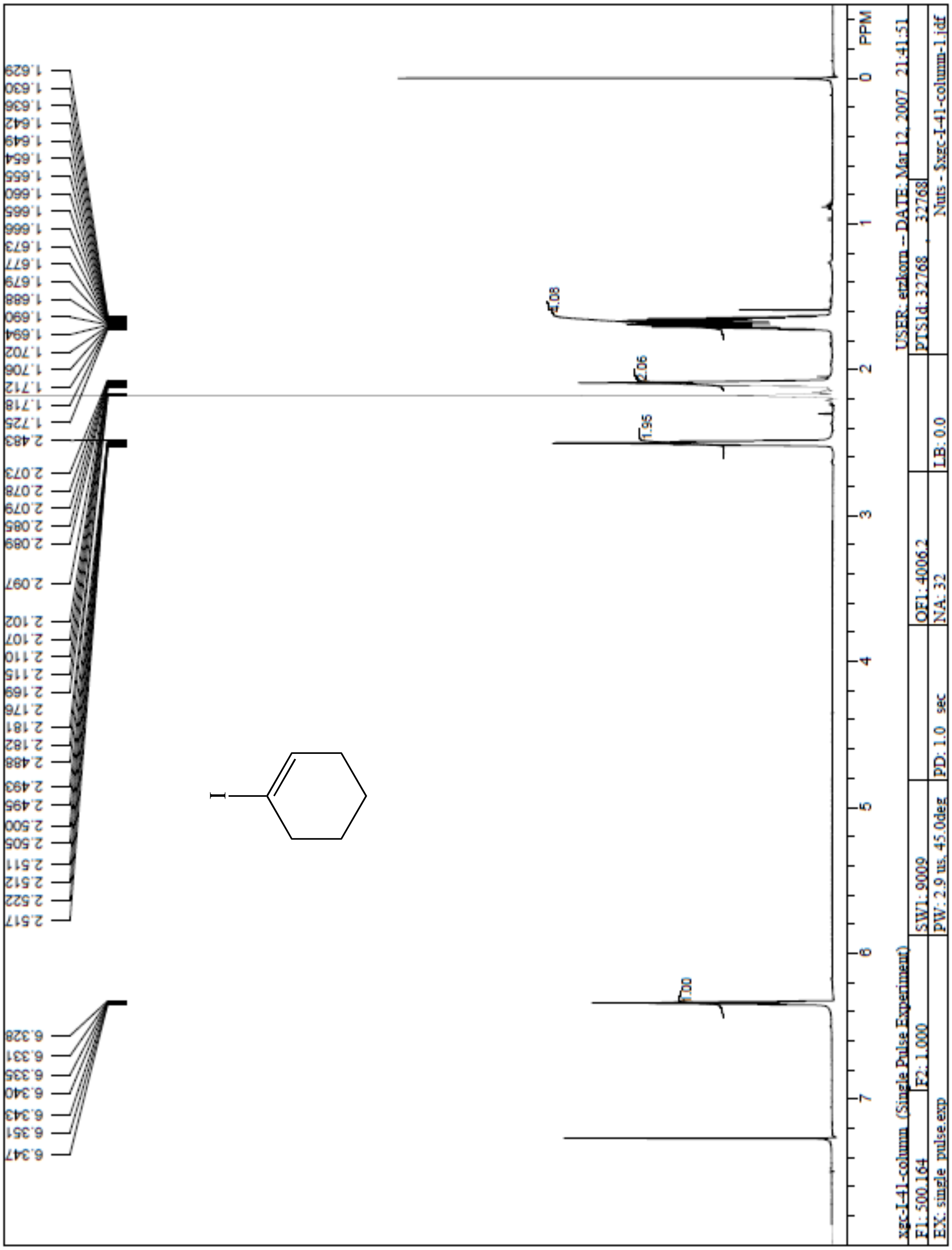

^{1}H NMR of 1-iodocyclohexene in $CDCl_3$ (500 MHz)

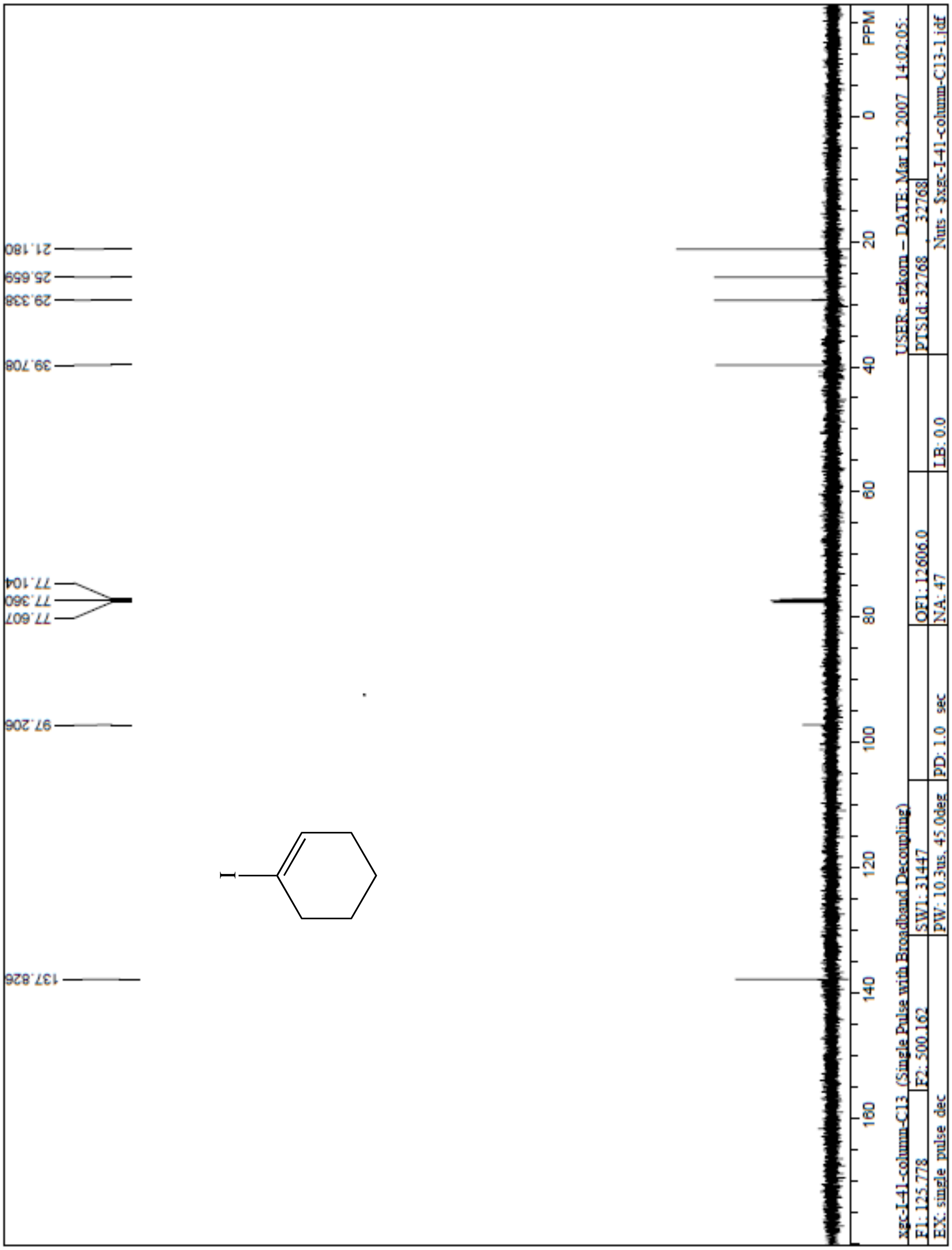

^{13}C NMR of 1-iodocyclohexene in $CDCl_3$ (125 MHz)

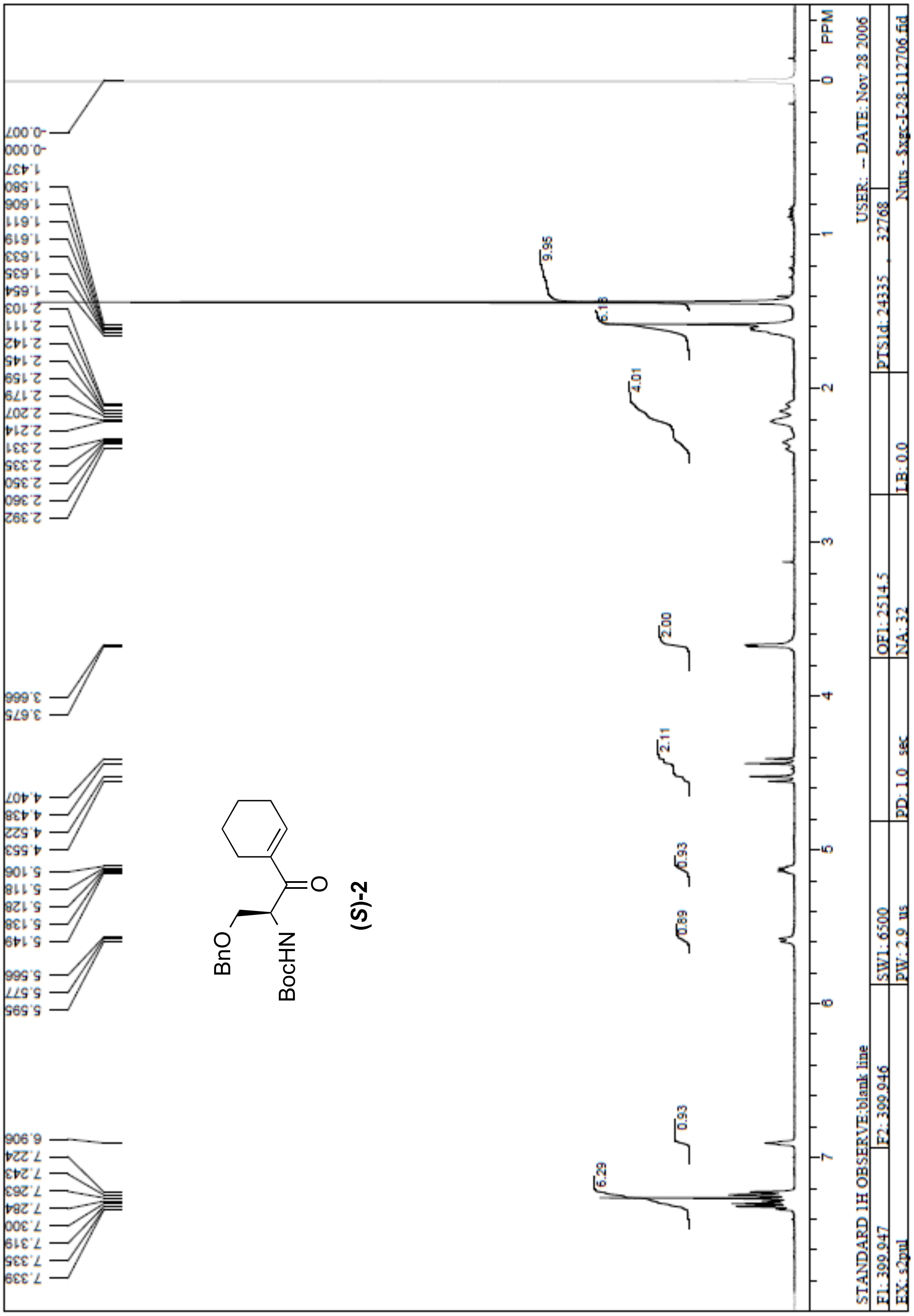

^{1}H NMR of ***(S)*-2** in $CDCl_3$ (400 MHz)

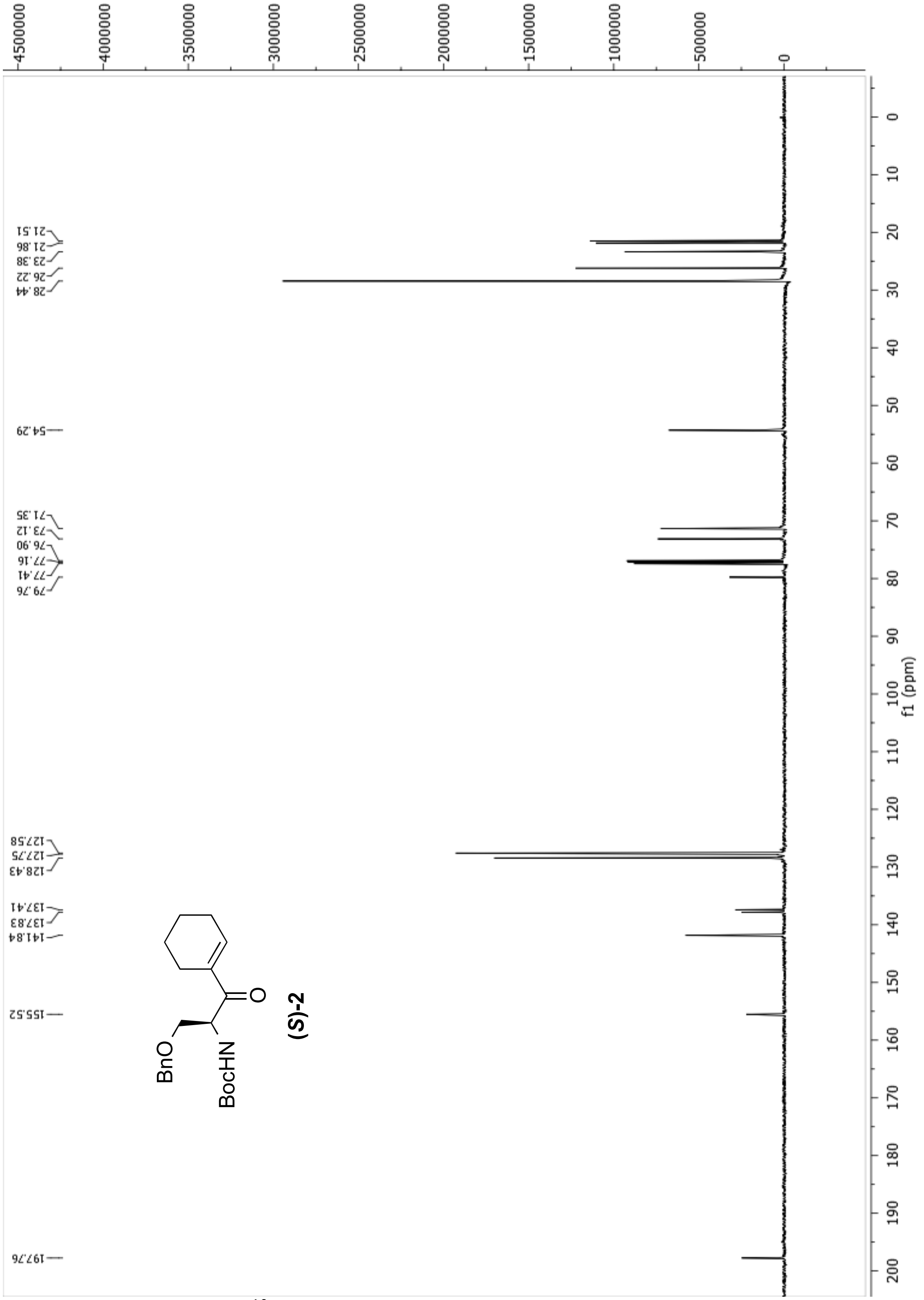

^{13}C NMR of ***(S)*-2** in $CDCl_3$ (100 MHz)

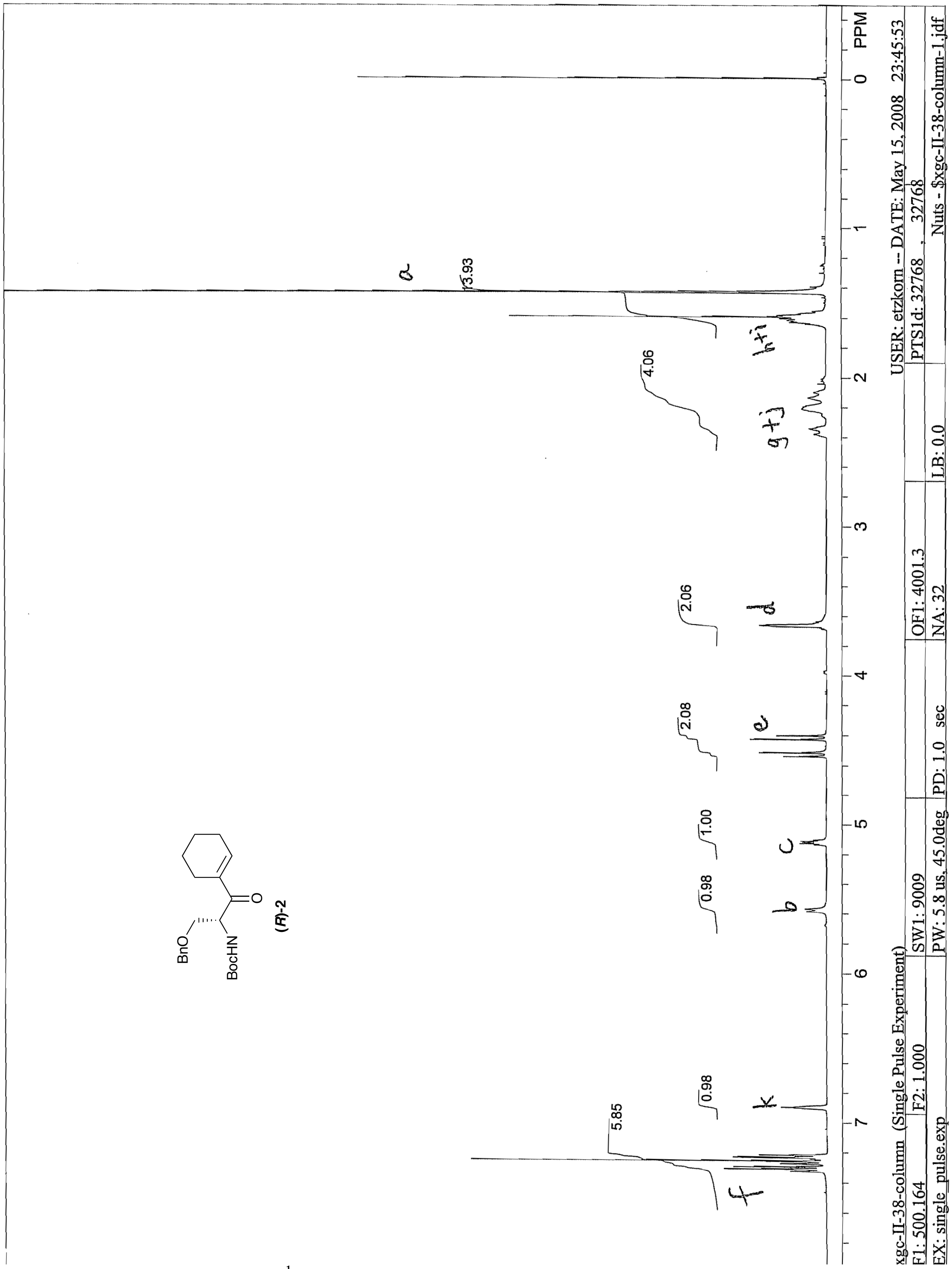

^{1}H NMR of ***(R)*-2** in $CDCl_3$ (400 MHz)

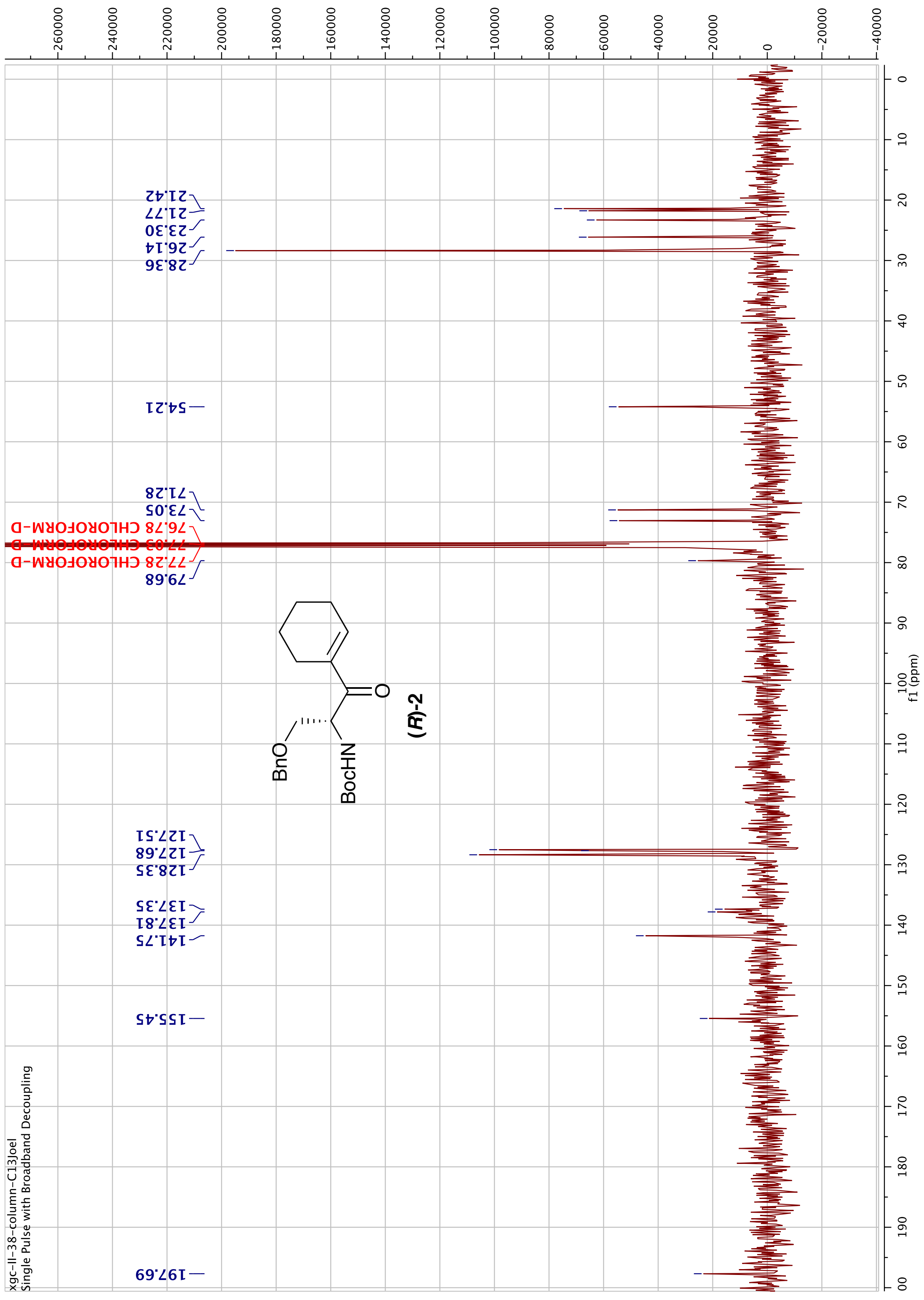

^{13}C NMR of ***(R)*-2** in $CDCl_3$ (100 MHz)

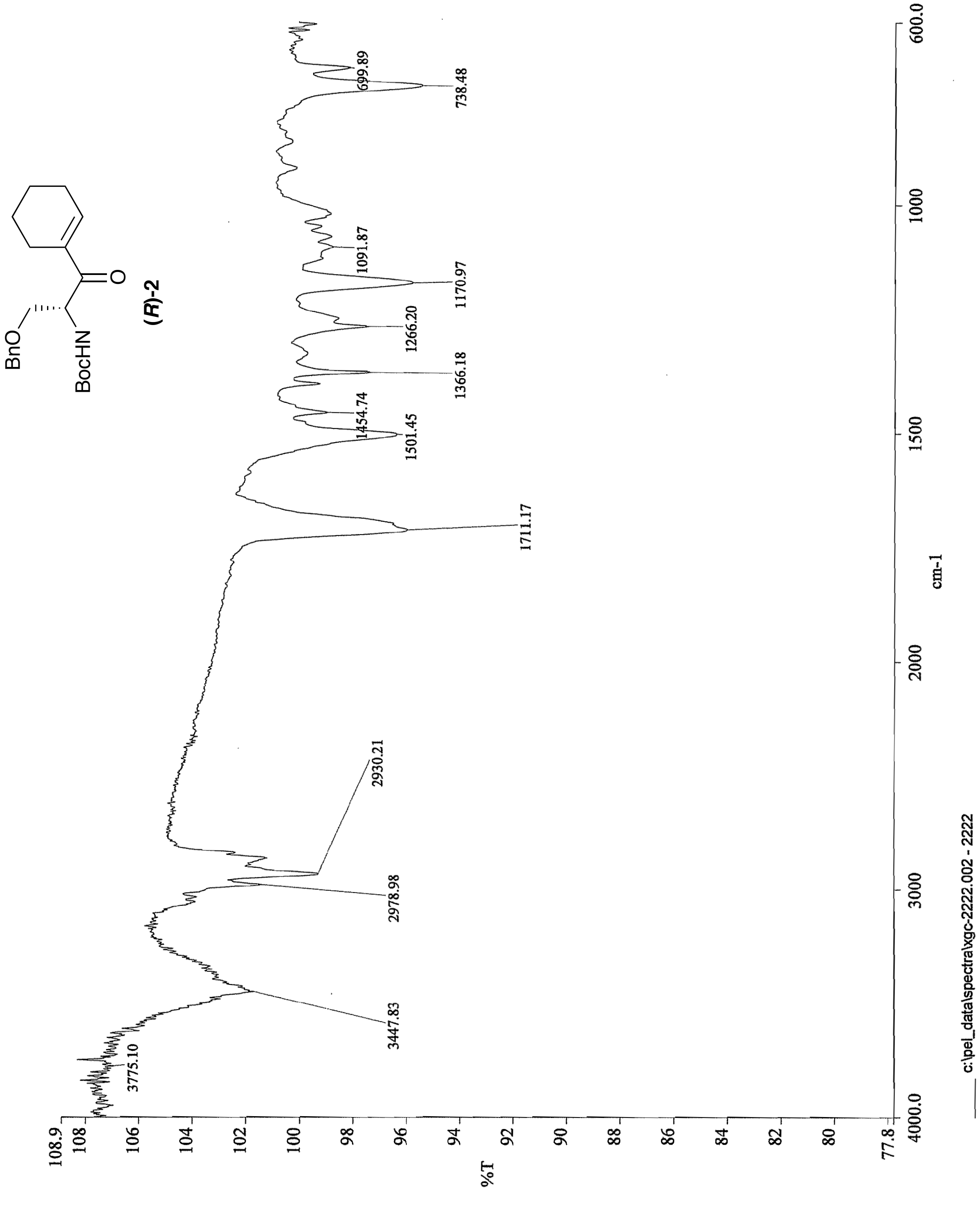
BnO
BocHN
O
(R)-2
%T
cm-1
108.9
108
106
104
102
100
98
96
94
92
90
88
86
84
82
80
77.8
4000.0
3000
2000
1500
1000
600.0
3775.10
3447.83
2978.98
2930.21
1711.17
1501.45
1454.74
1366.18
1266.20
1170.97
1091.87
738.48
699.89
c:\pel_data\spectra\xgc-2222.002 - 2222

IR of ***(R)*-2** (neat)

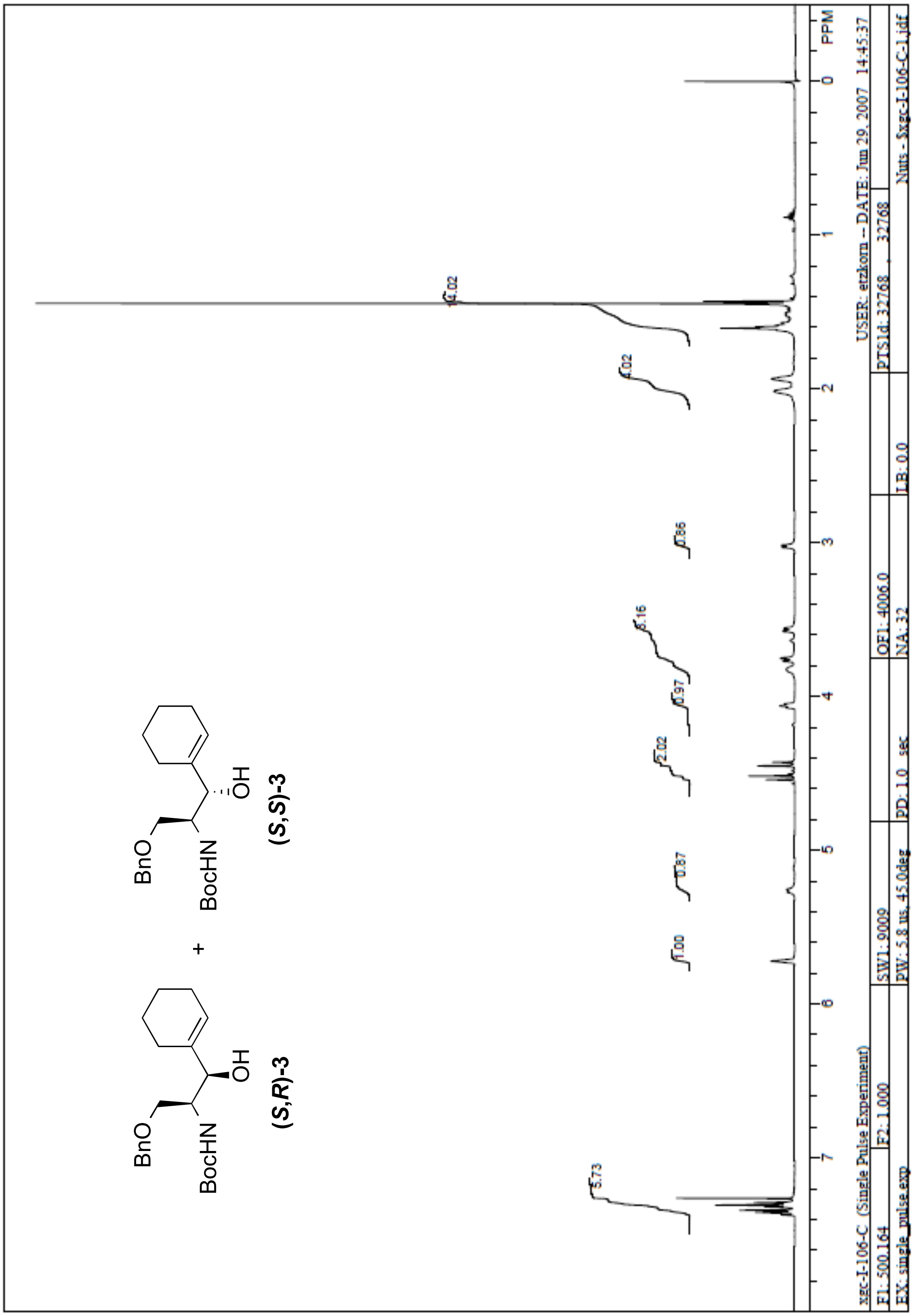

^{1}H NMR of the mixture of **(2*S*,3*R*)-3** and **(2*S*,3*S*)-3** in $CDCl_3$ (500 MHz)

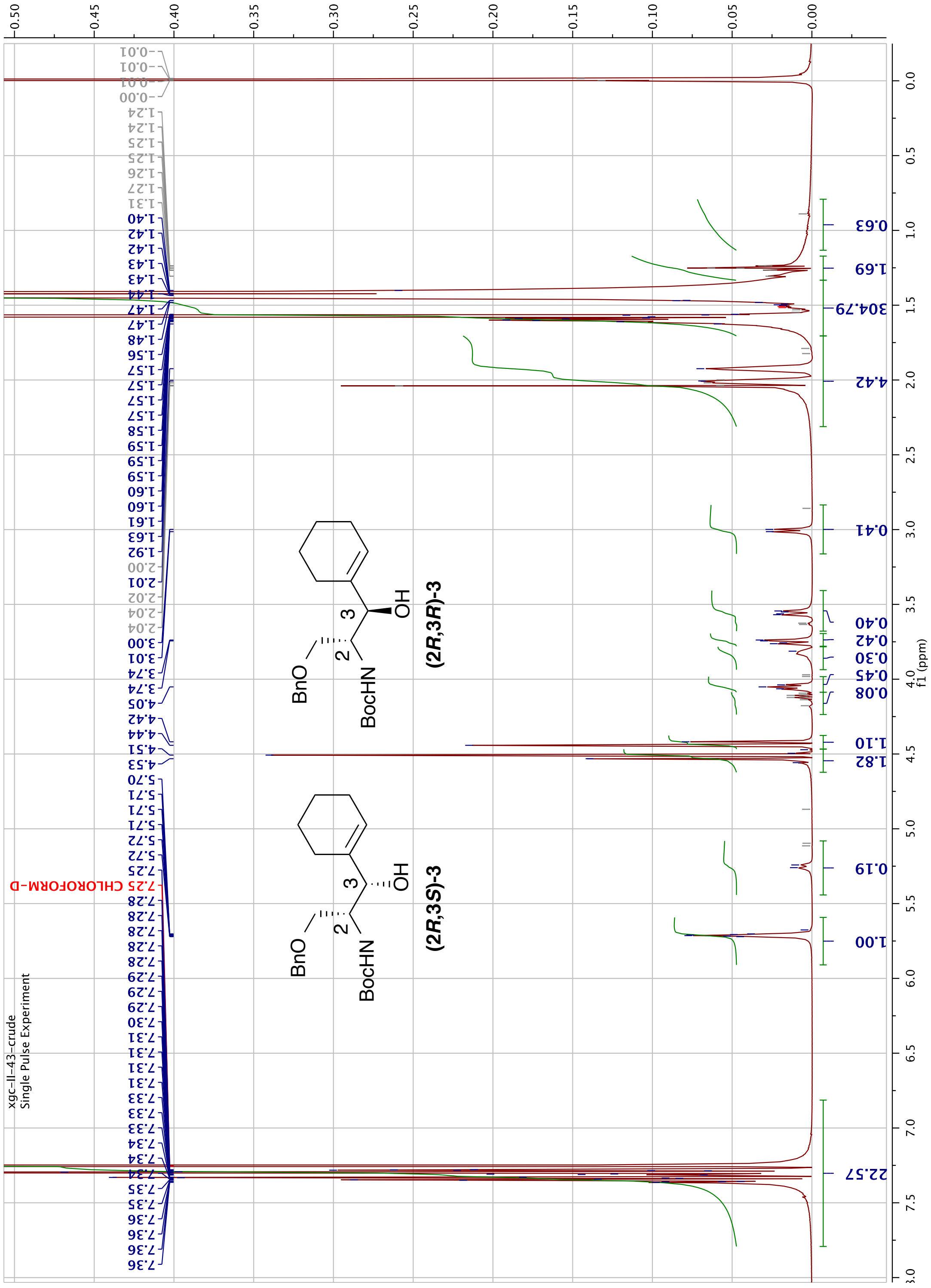

^{1}H NMR of the mixture of **(2*R*,3*S*)-3** and **(2*R*,3*R*)-3** in $CDCl_3$ (500 MHz)

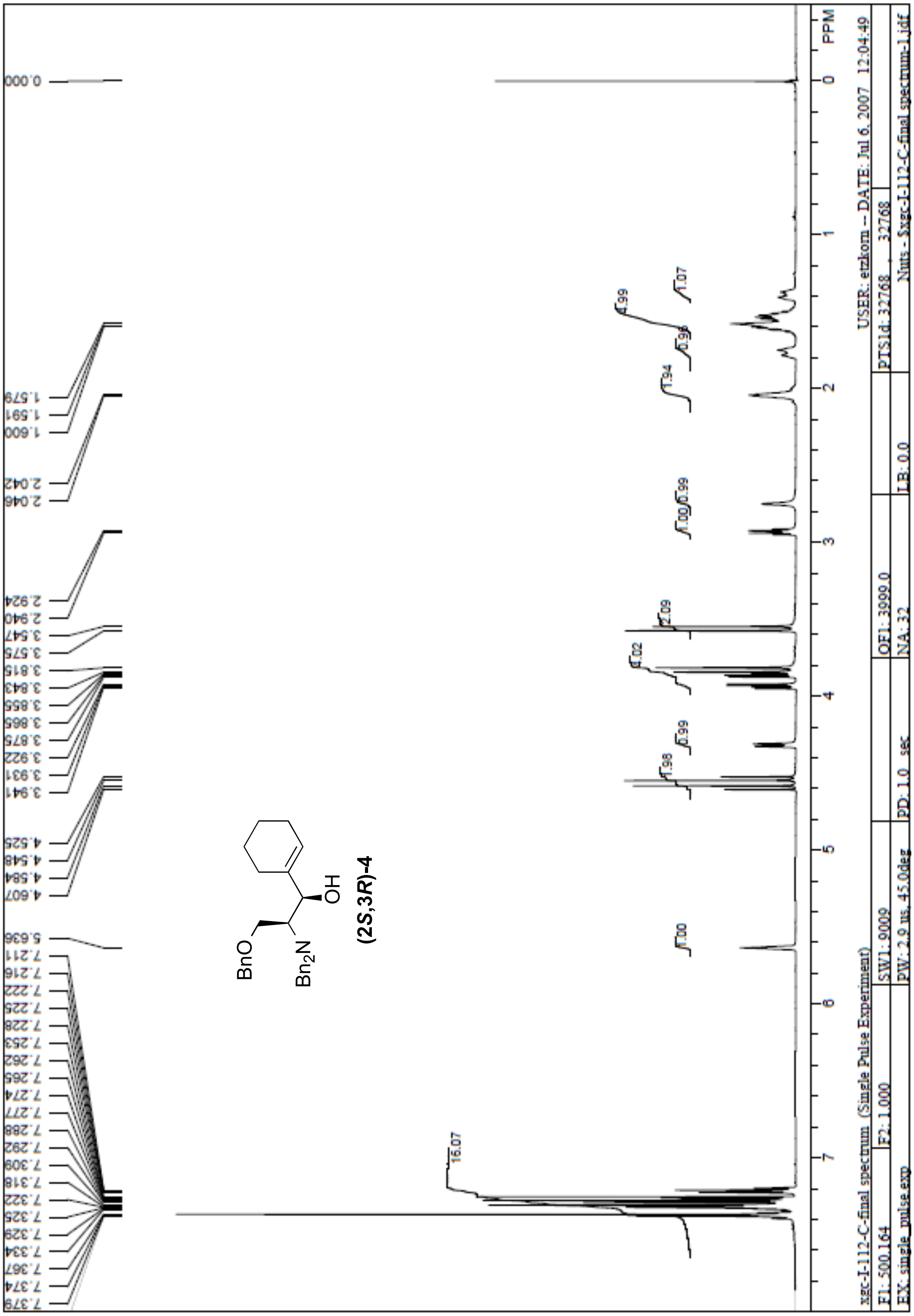

^{1}H NMR of **(2*S*,3*R*)-4** in $CDCl_3$ (500 MHz)

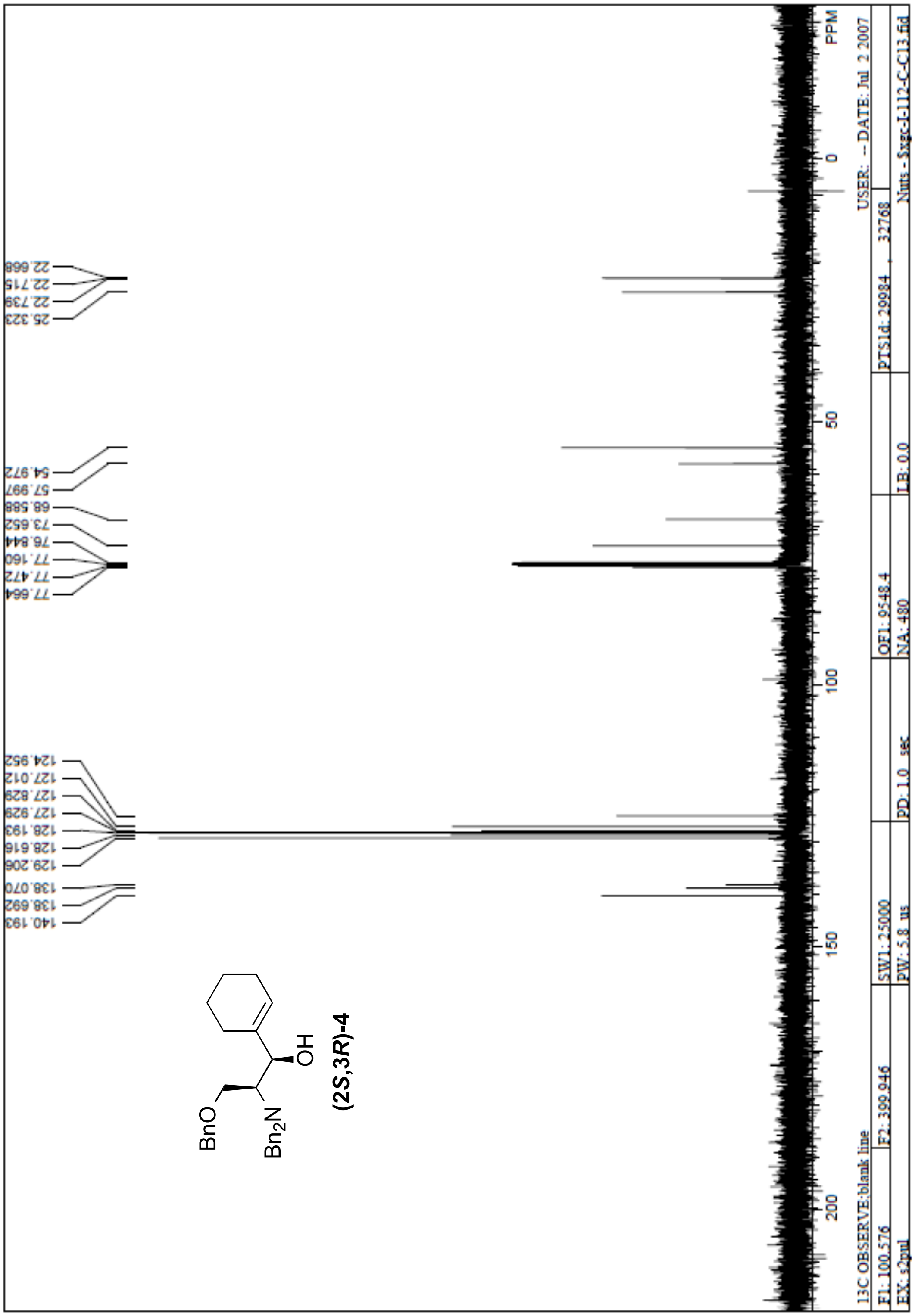

^{13}C NMR of **(2*S*,3*R*)-4** in $CDCl_3$ (100 MHz)

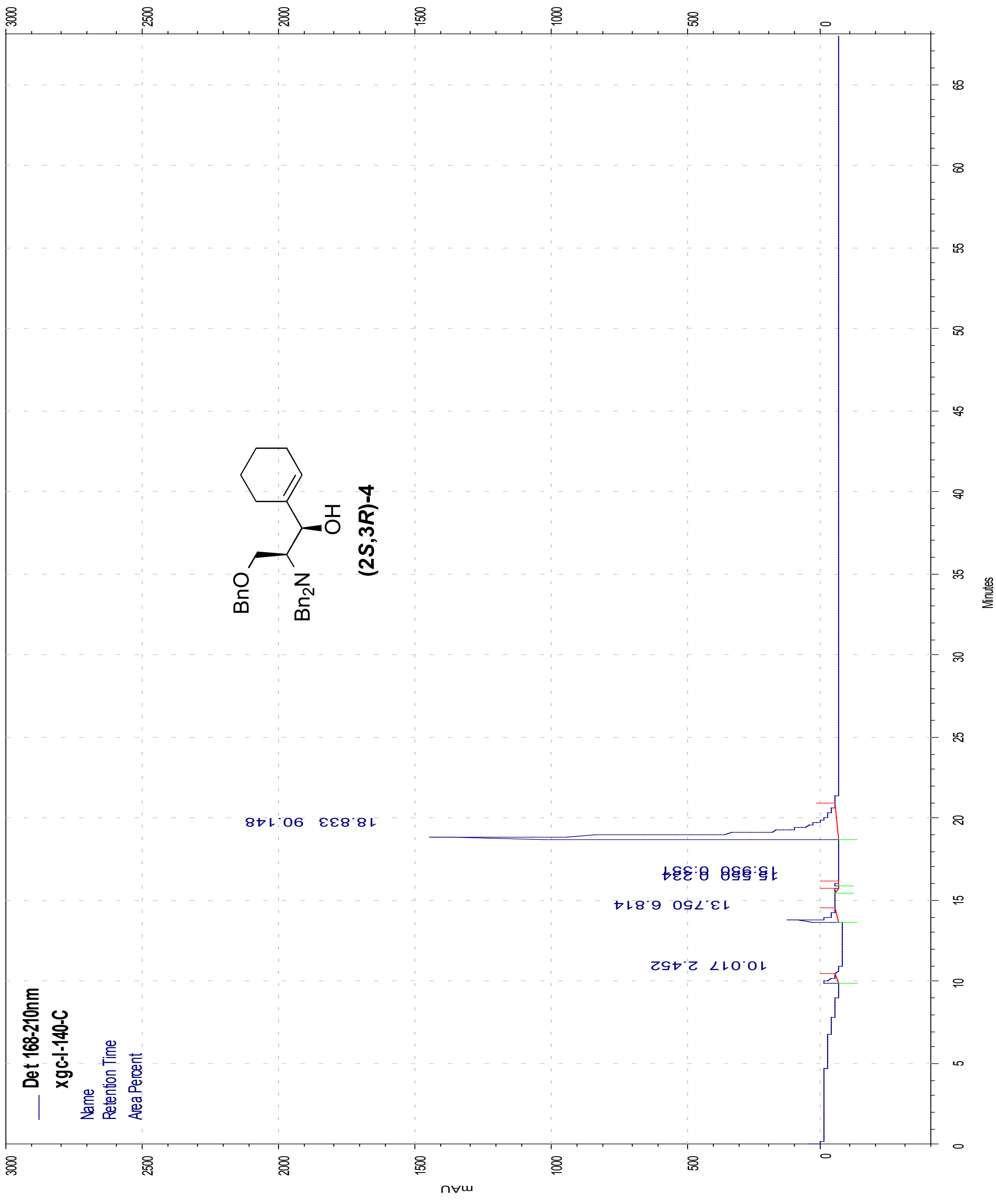

Analytical HPLC of **(2*S*,3*R*)-4**

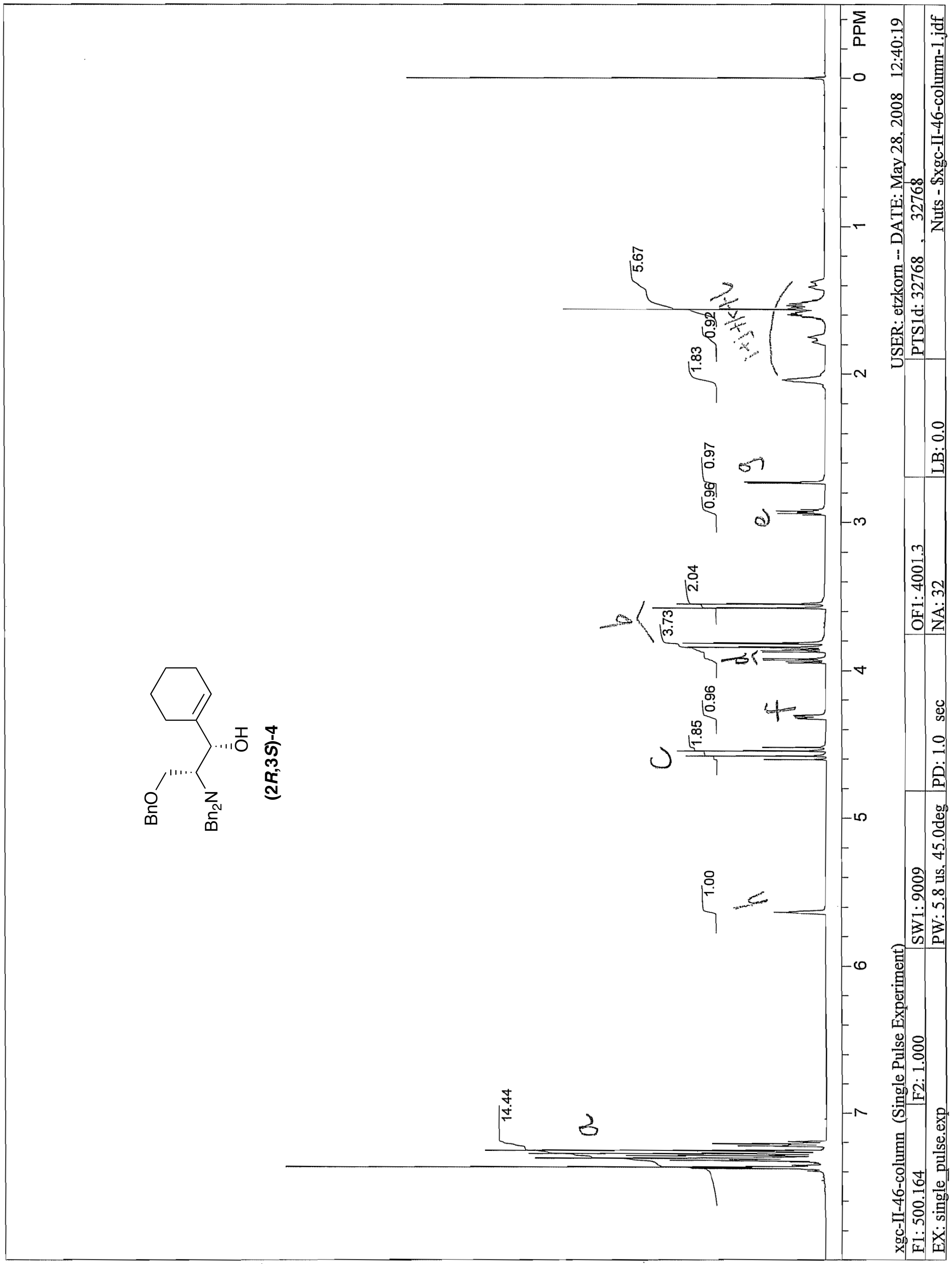

^{1}H NMR of **(2*R*,3*S*)-4** in $CDCl_3$ (500 MHz)

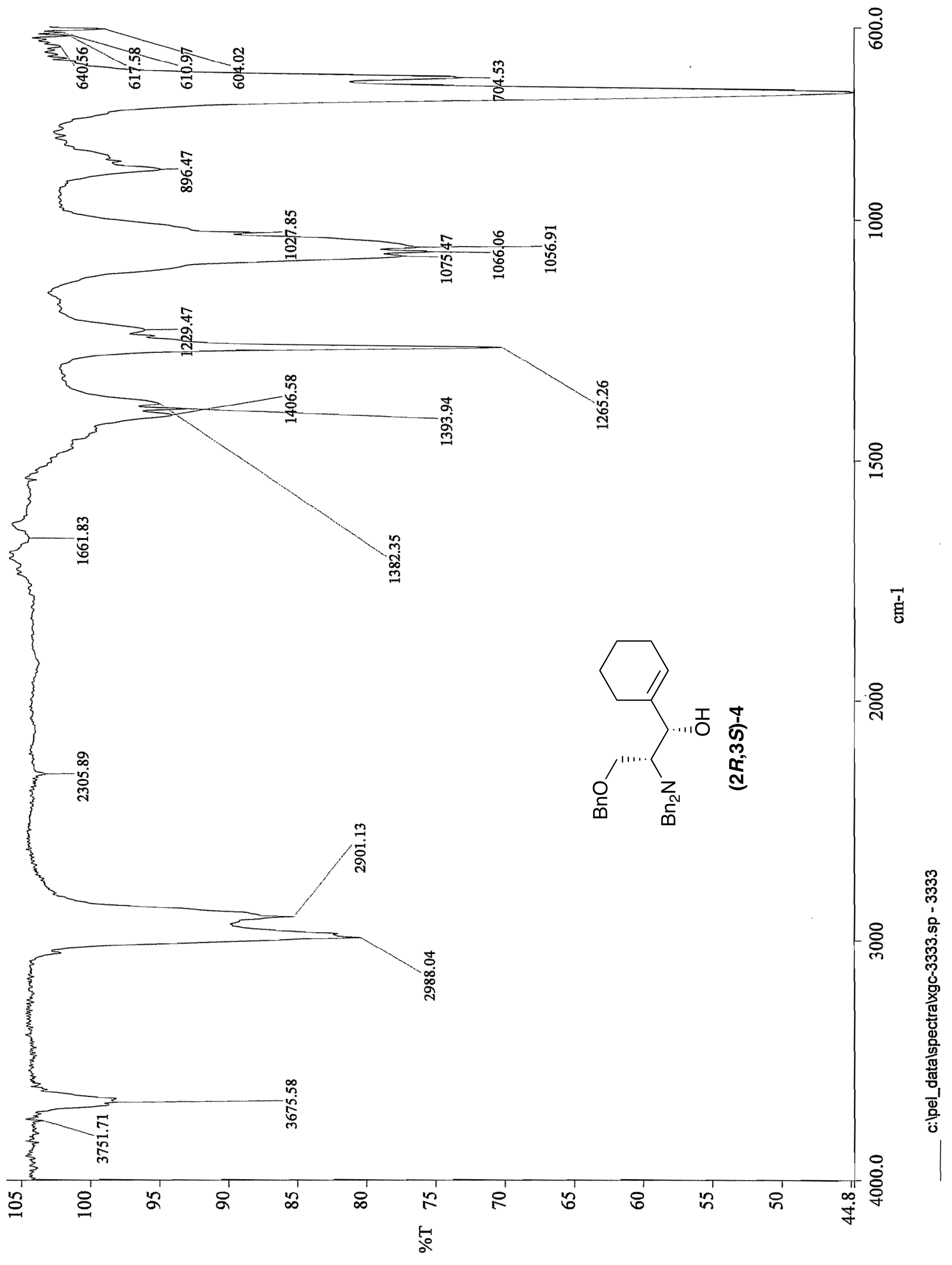

FTIR of **(2*R*,3*S*)-4** (neat)

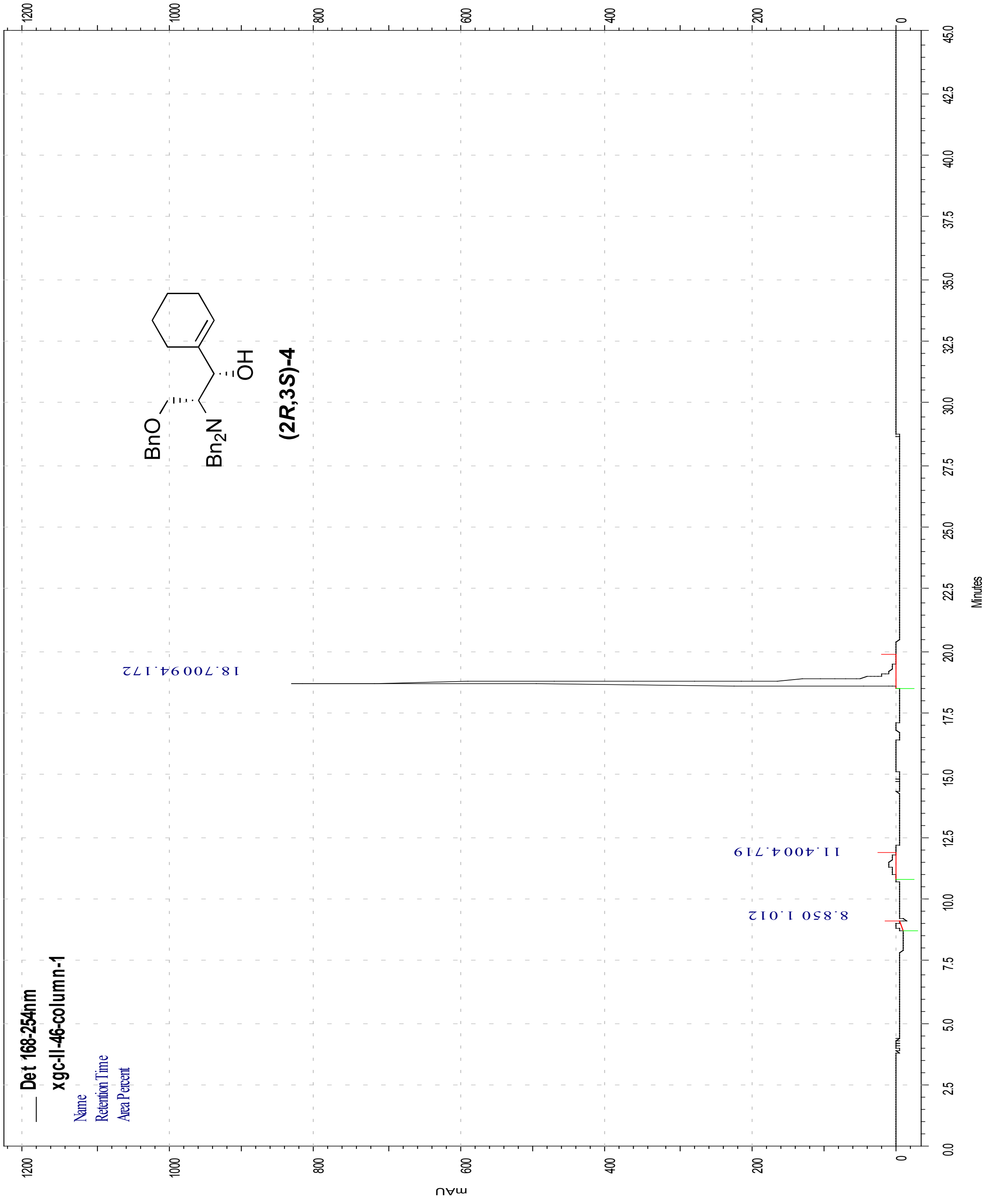

Analytical HPLC of **(2*R*,3*S*)-4**

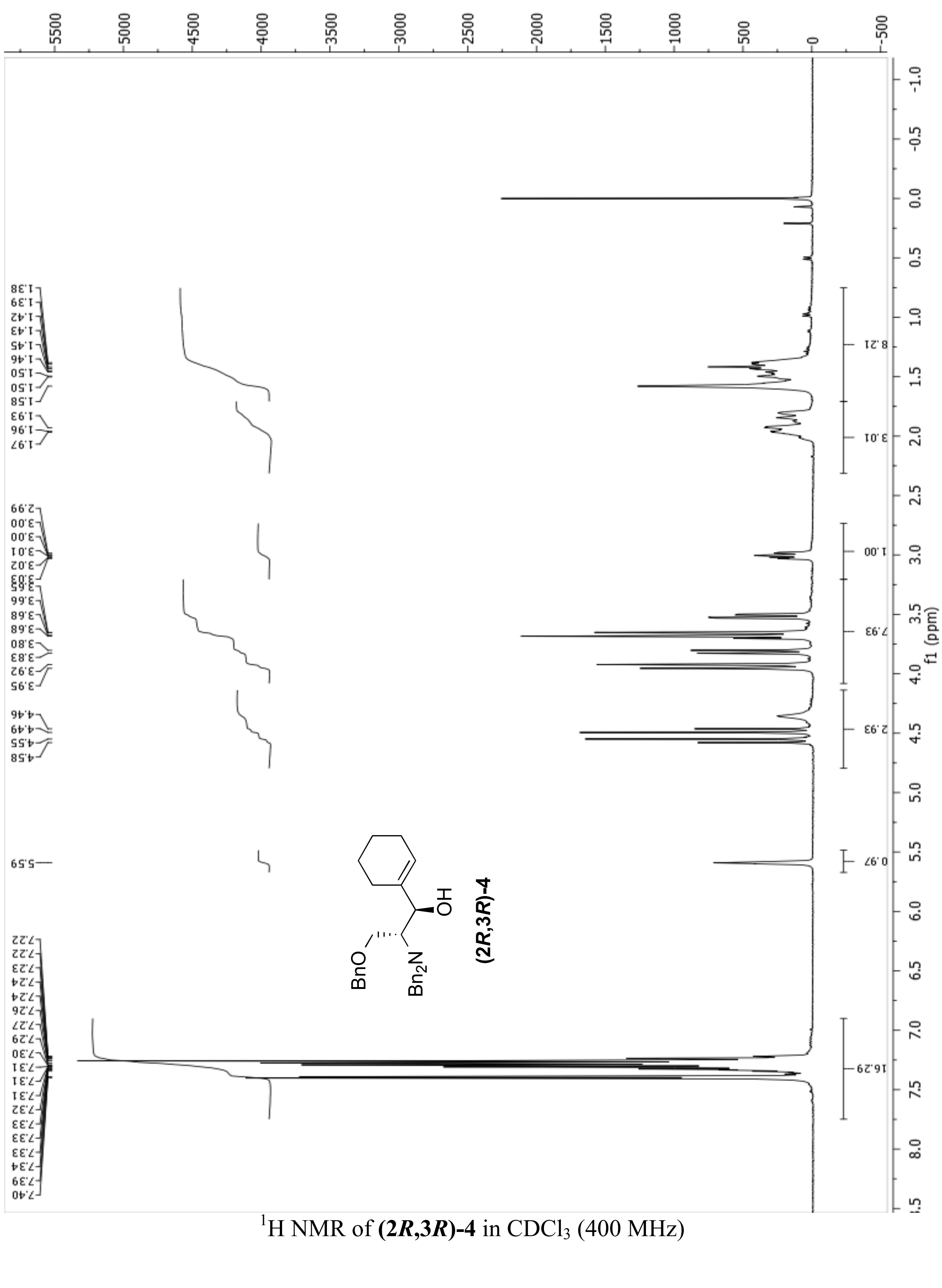

^{1}H NMR of **(2*R*,3*R*)-4** in $CDCl_3$ (400 MHz)

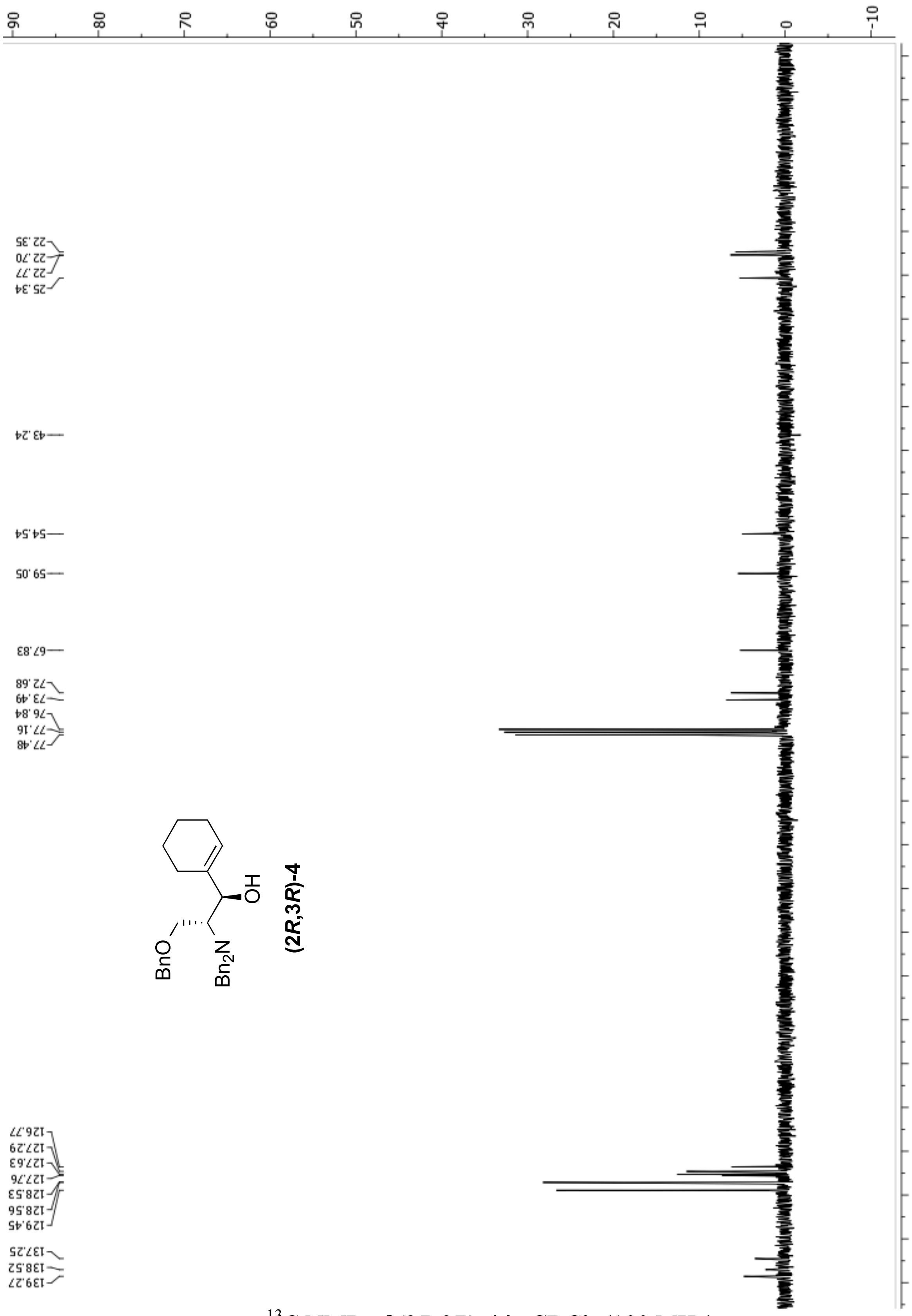

^{13}C NMR of **(2*R*,3*R*)-4** in $CDCl_3$ (100 MHz)

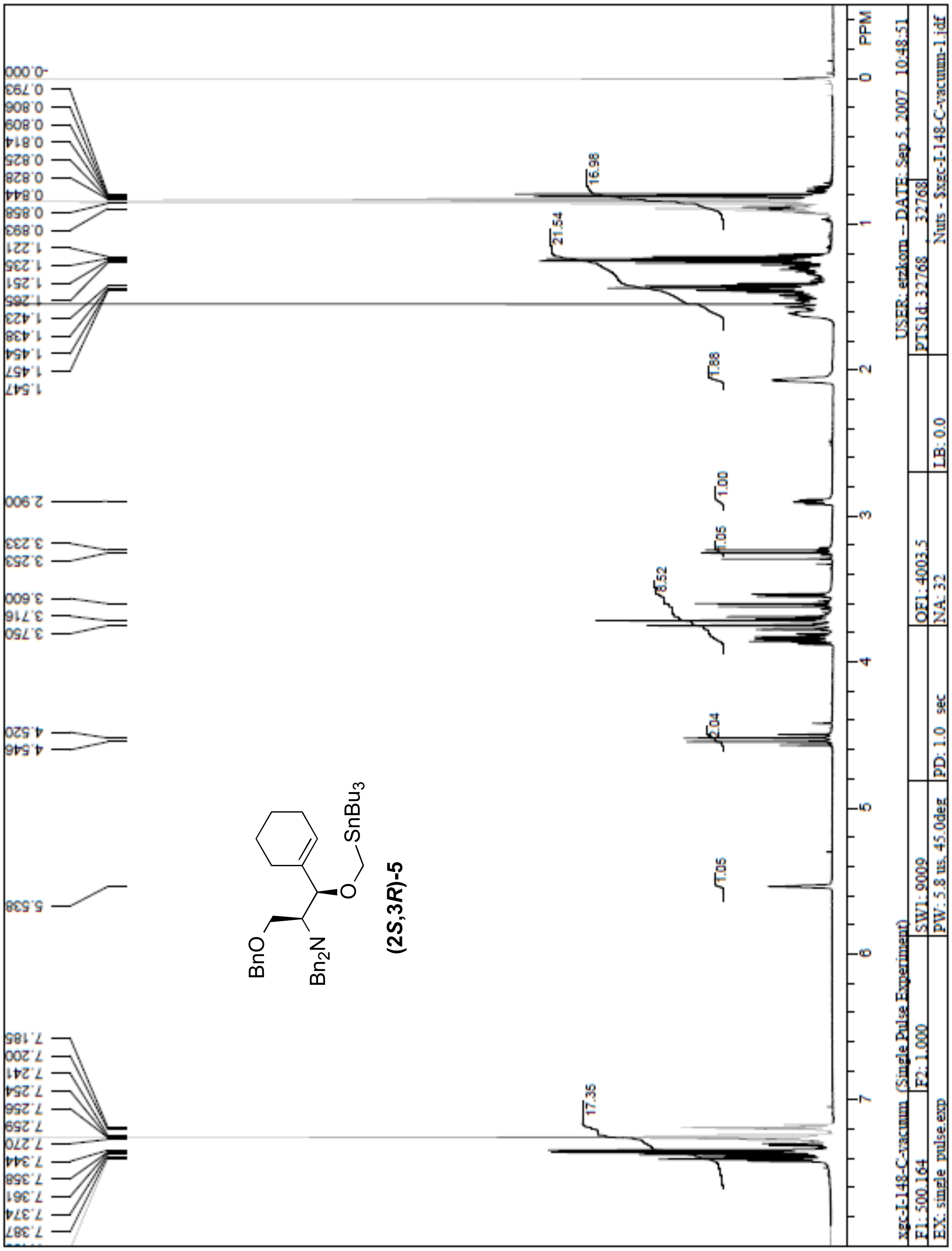

^{1}H NMR of **(2*S*,3*R*)-5** in $CDCl_3$ (500 MHz)

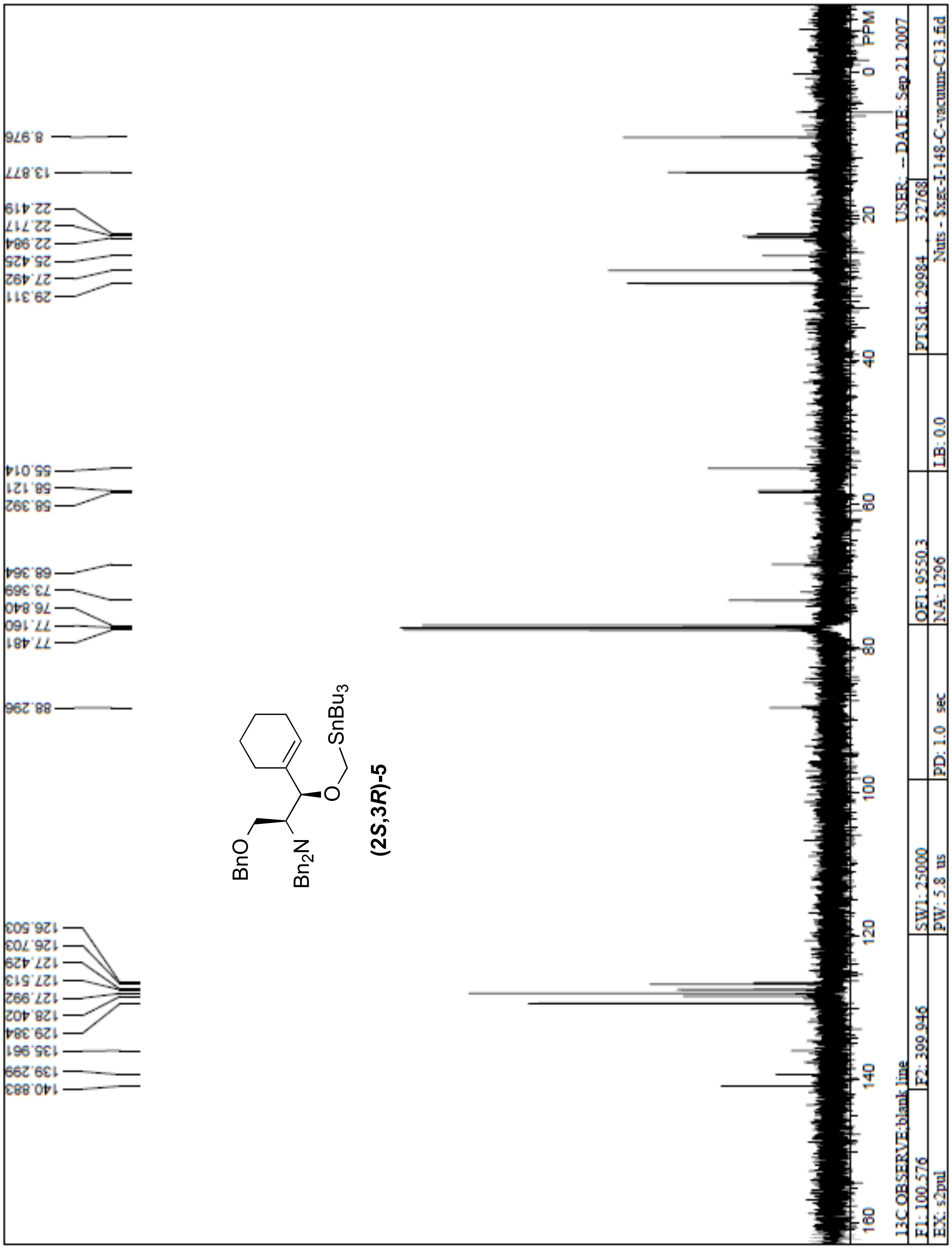

^{13}C NMR of **(2*S*,3*R*)-5** in $CDCl_3$ (100 MHz)

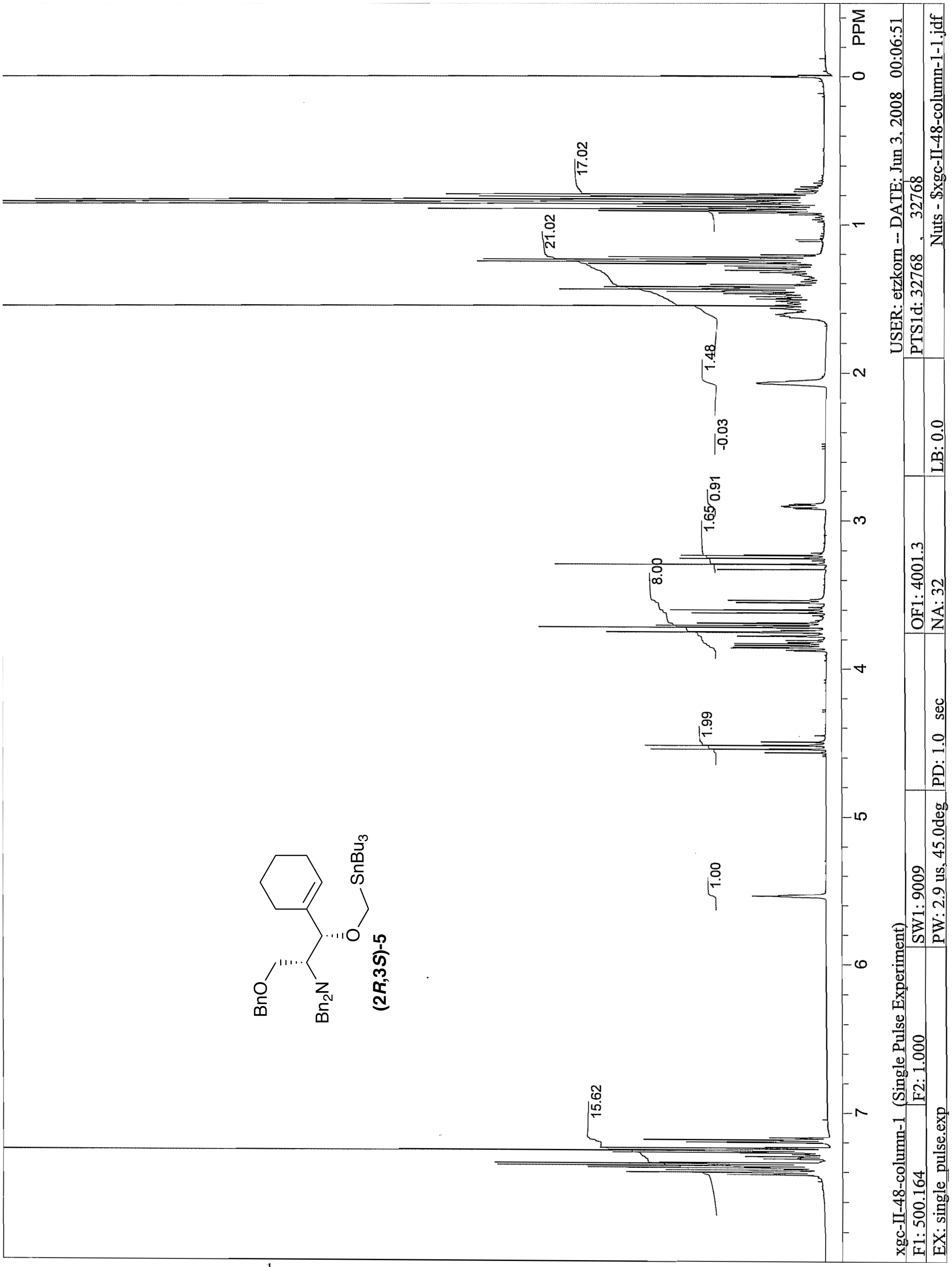

^{1}H NMR of **(2*R*,3*S*)-5** in $CDCl_3$ (500 MHz)

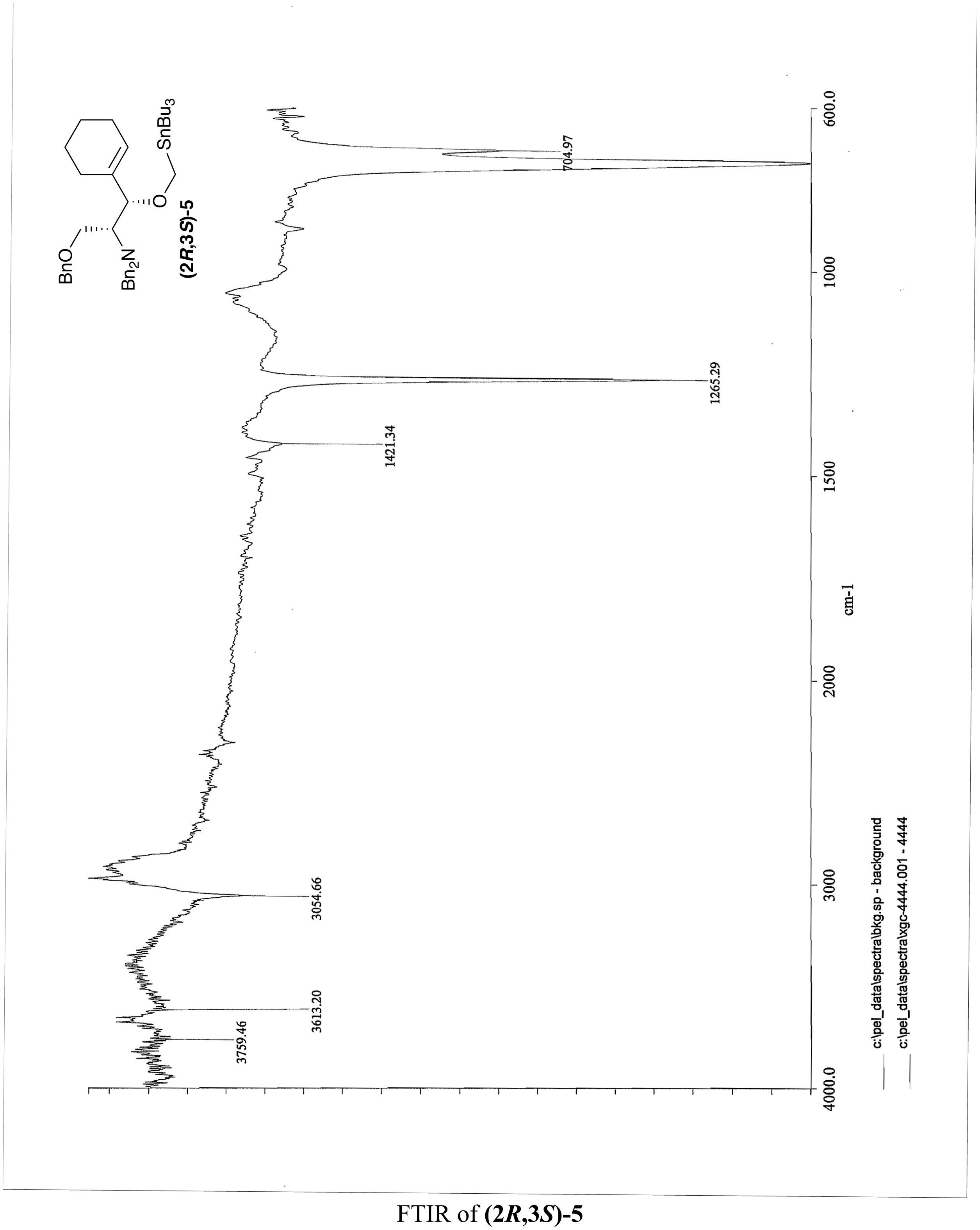

FTIR of **(2*R*,3*S*)-5**

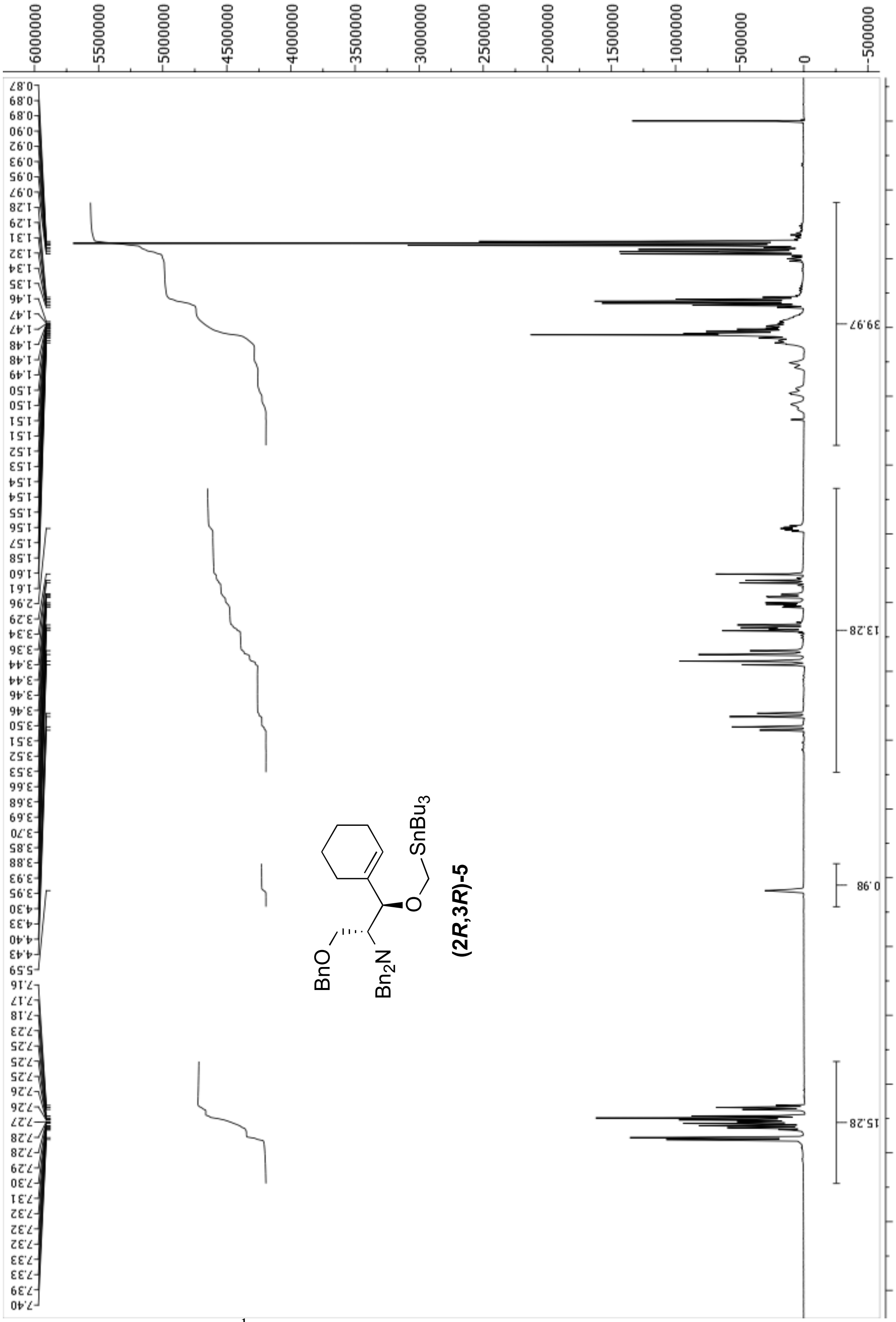

^{1}H NMR of **(2*R*,3*R*)-5** in $CDCl_3$ (500 MHz)

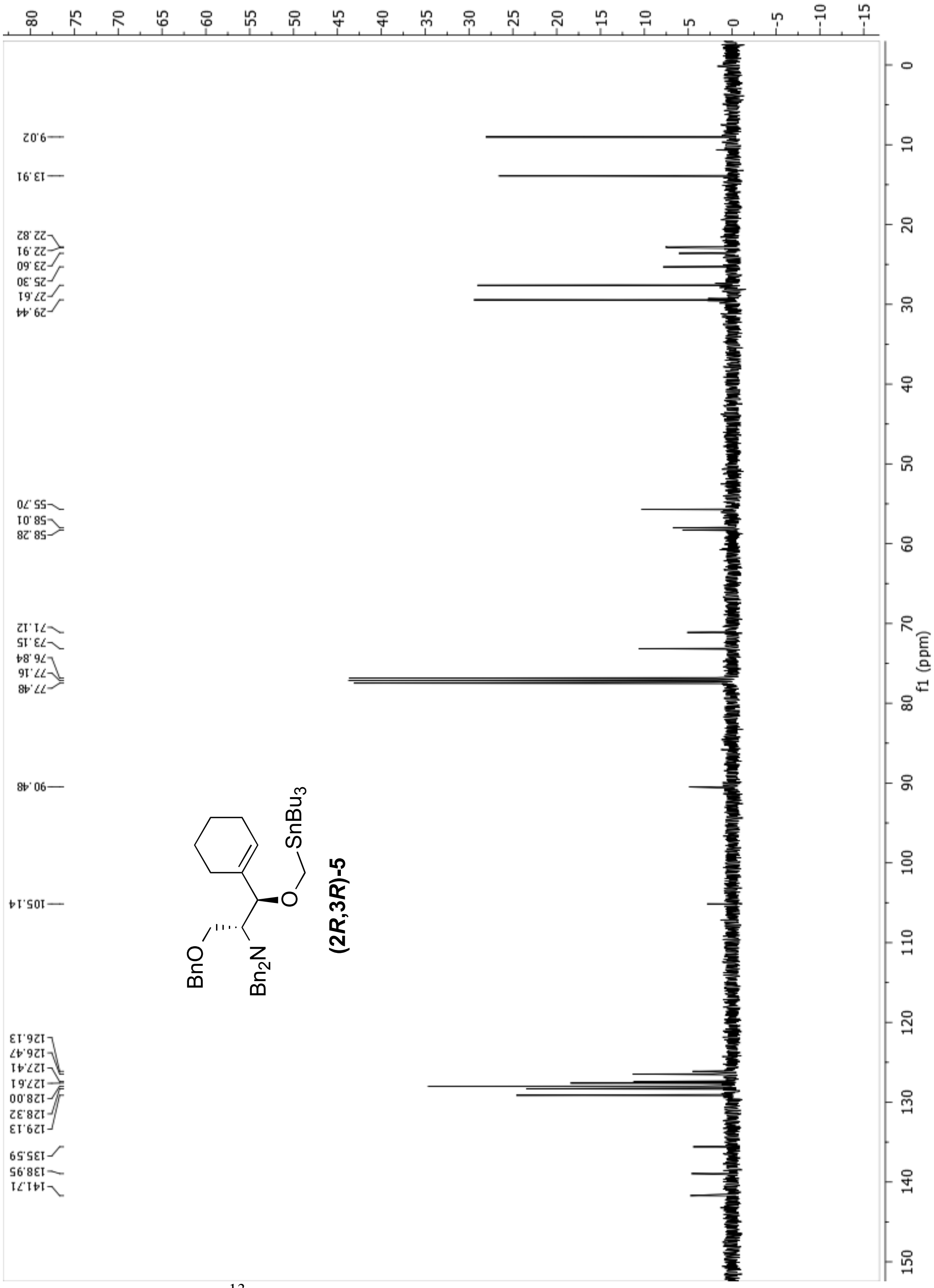

^{13}C NMR of **(2*R*,3*R*)-5** in $CDCl_3$ (100 MHz)

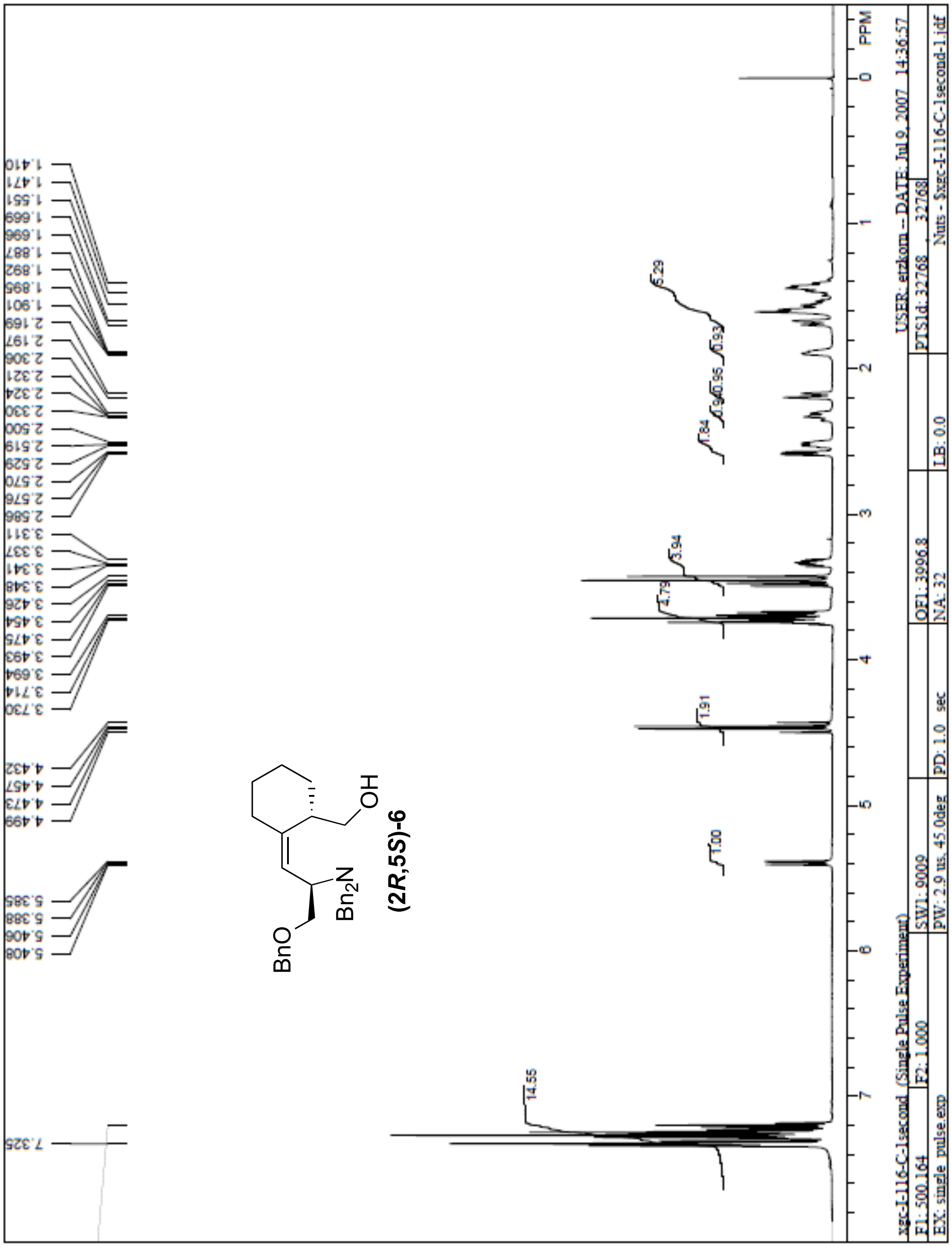

^{1}H NMR of **(2*R*,5*S*)-6** in $CDCl_3$ (500 MHz)

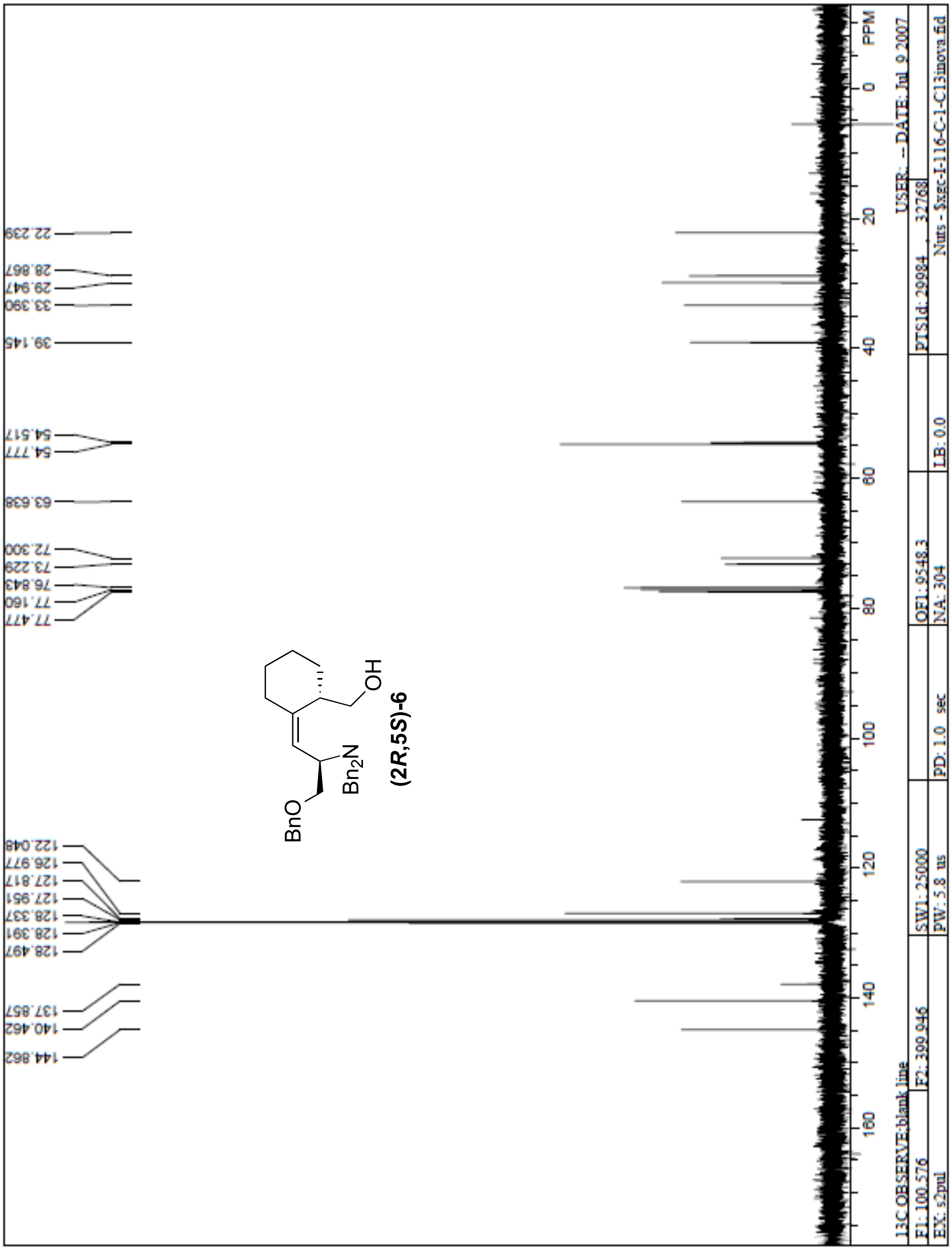

^{13}C NMR of **(2*R*,5*S*)-6** in $CDCl_3$ (100 MHz)

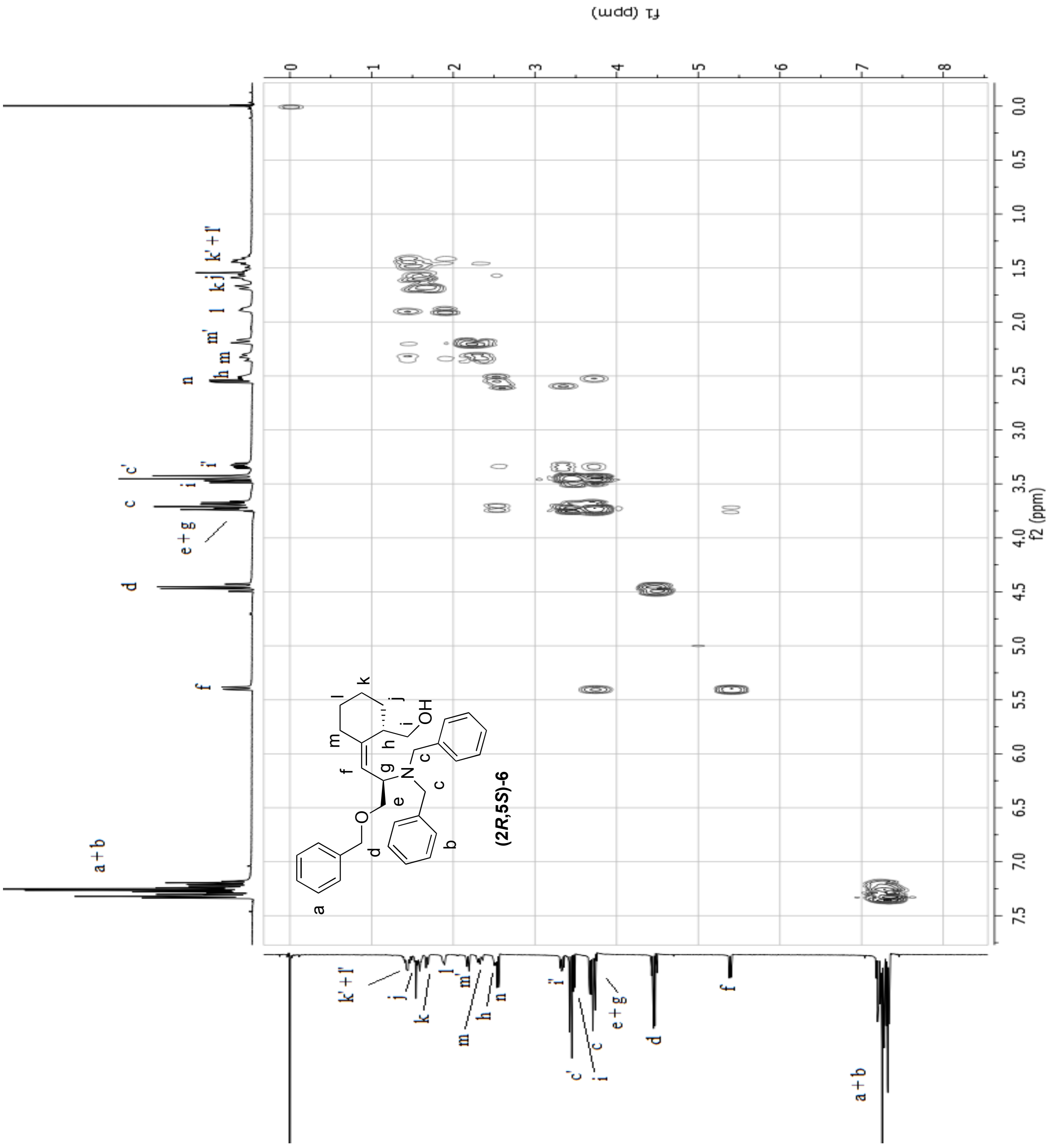

COSY of **(2*R*,3*Z*,5*S*)-6** in $CDCl_3$ (500 MHz) with assignments

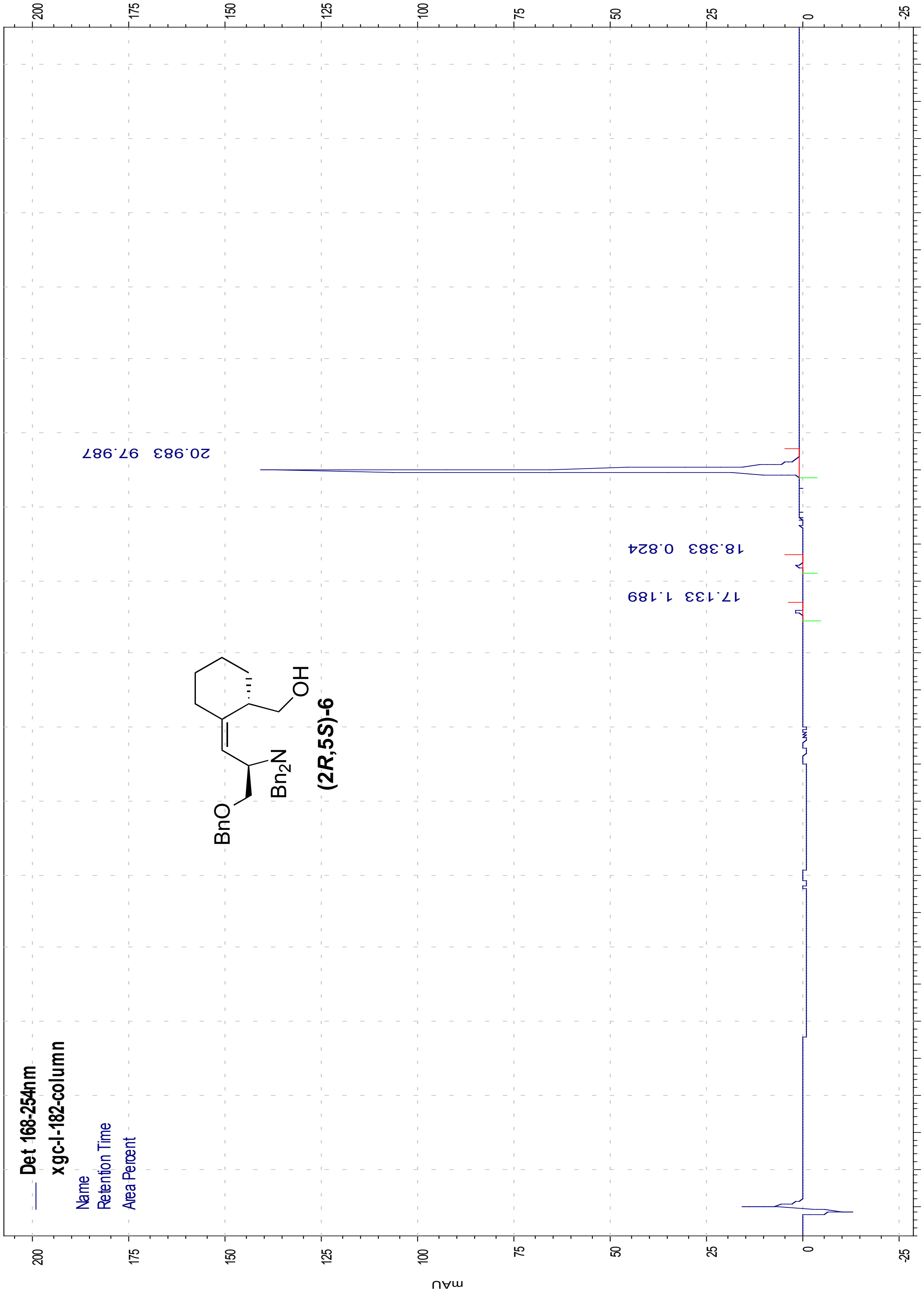

Analytical HPLC of **(2*R*,3*Z*,5*S*)- 6**

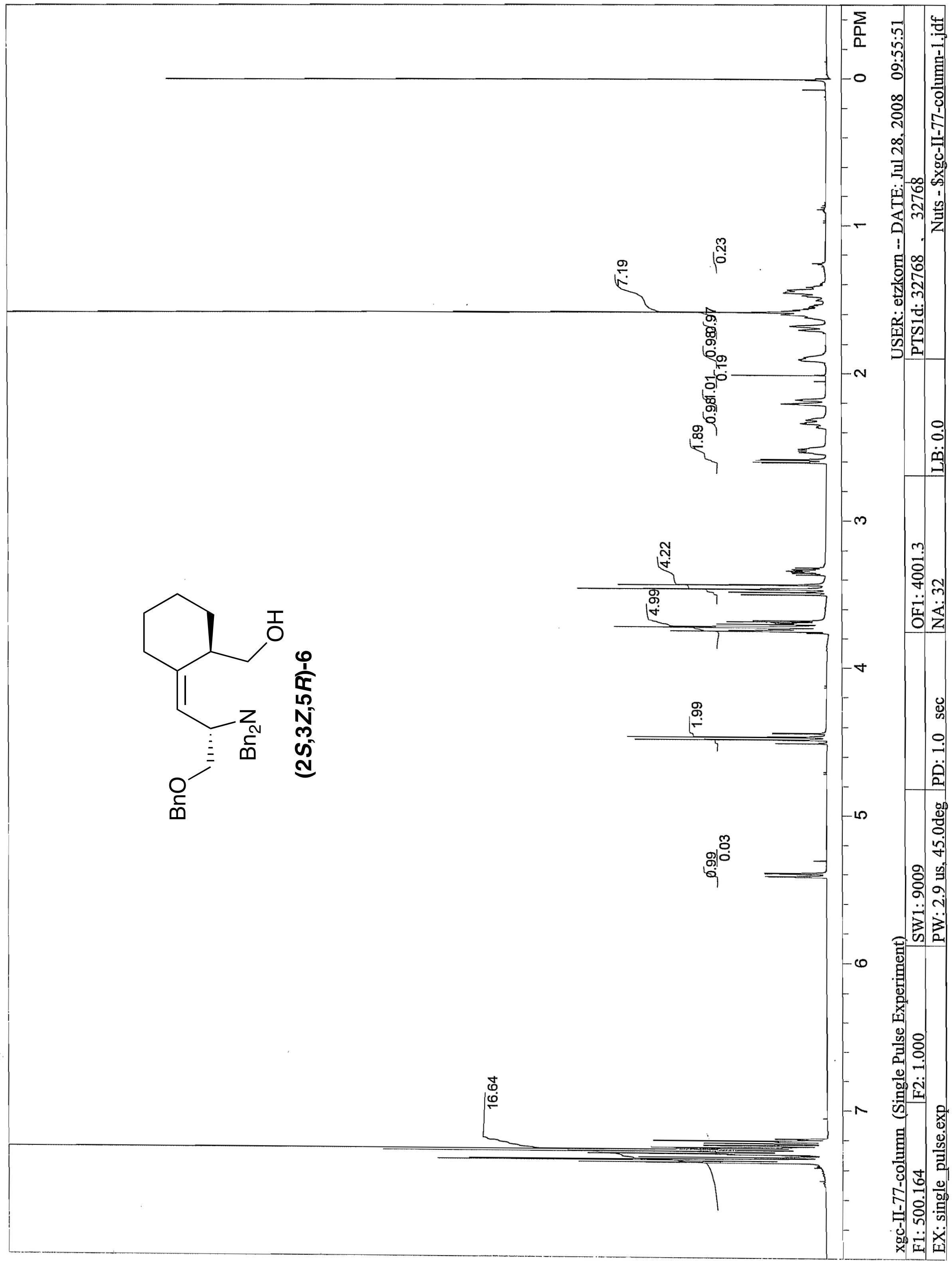

^{1}H NMR of **(2*S*,3*Z*,5*R*)-6** in $CDCl_3$ (500 MHz)

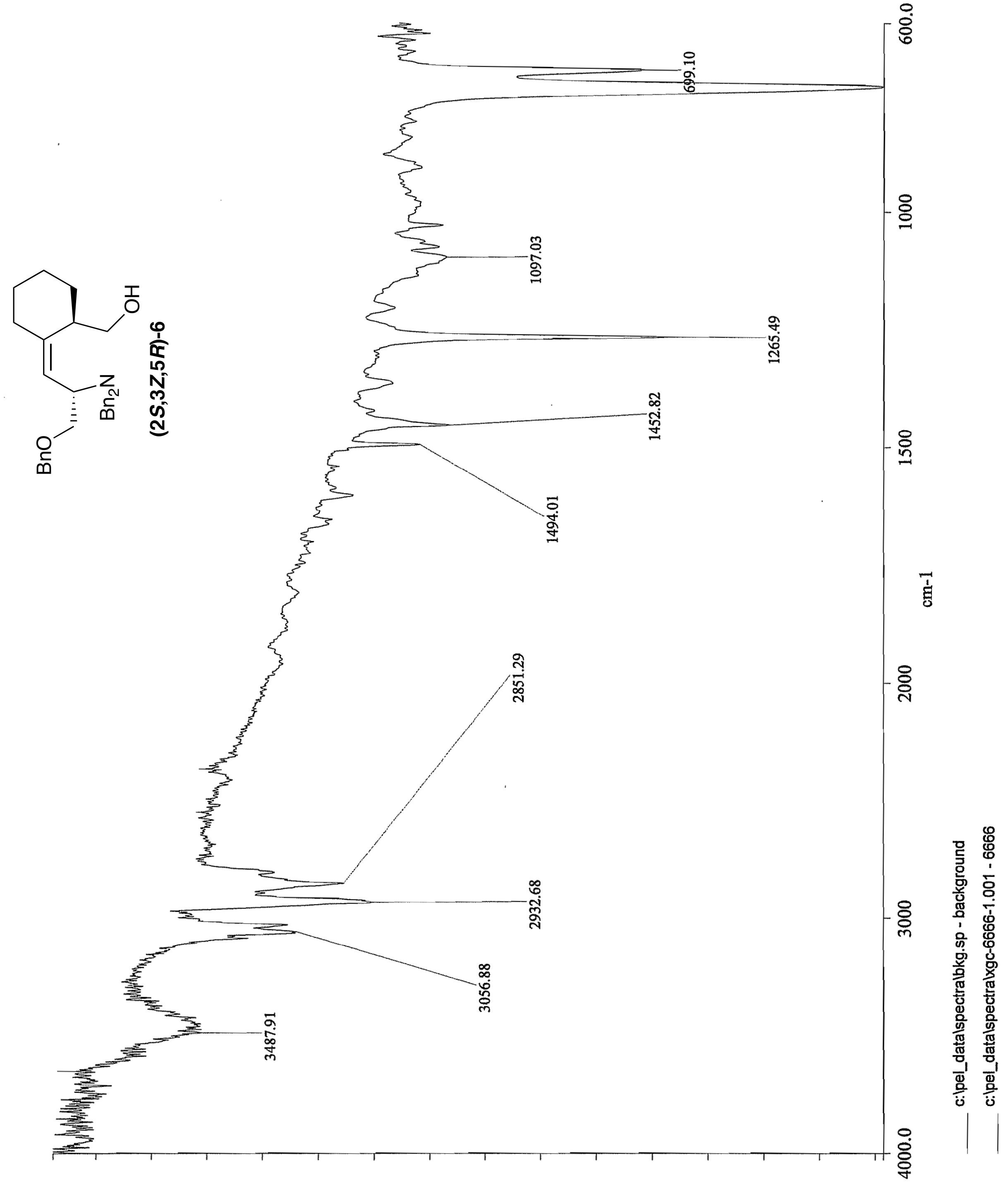

FTIR of **(2*S*,3*Z*,5R)-6** (neat)

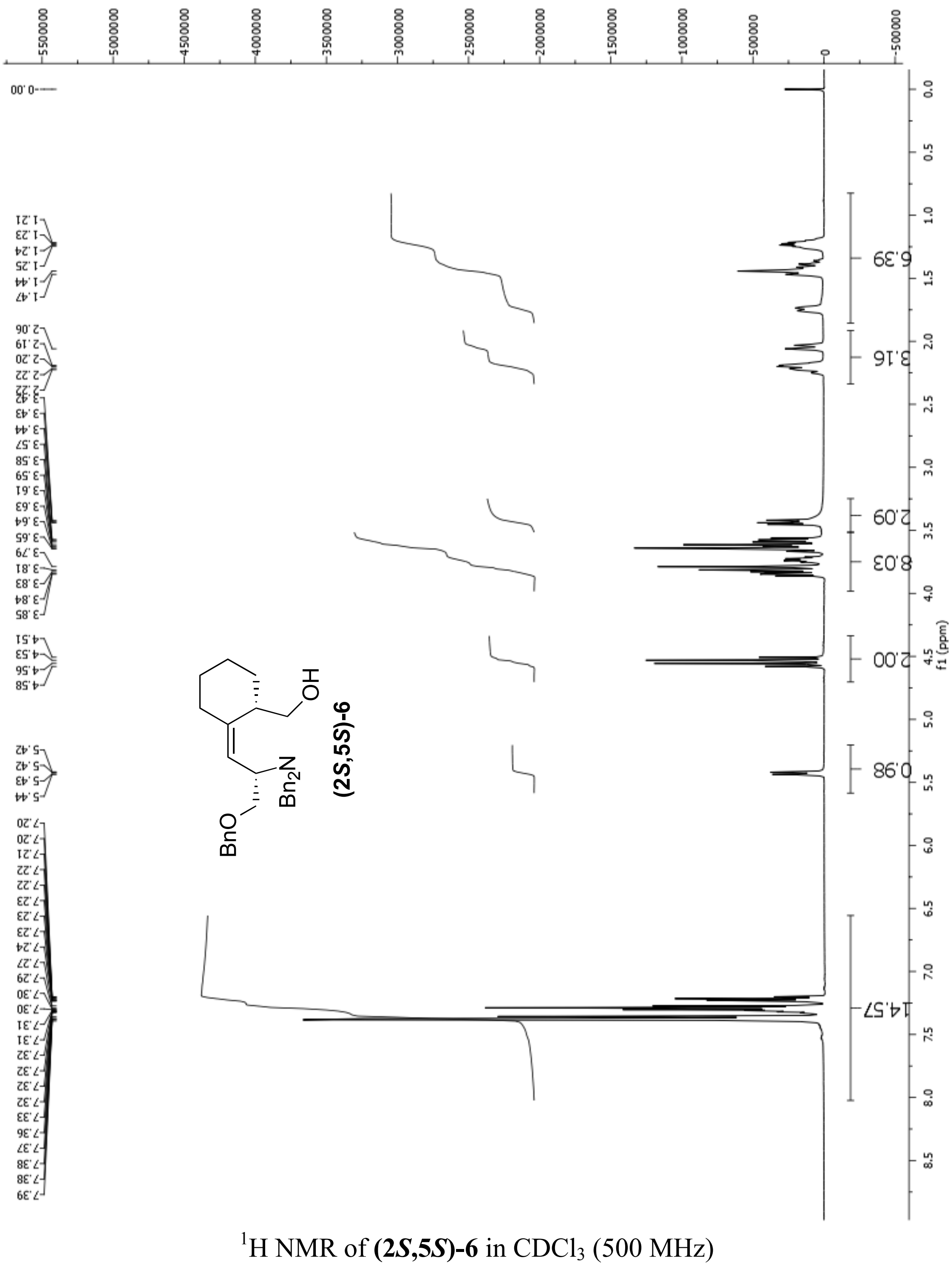

^{1}H NMR of **(2*S*,5*S*)-6** in $CDCl_3$ (500 MHz)

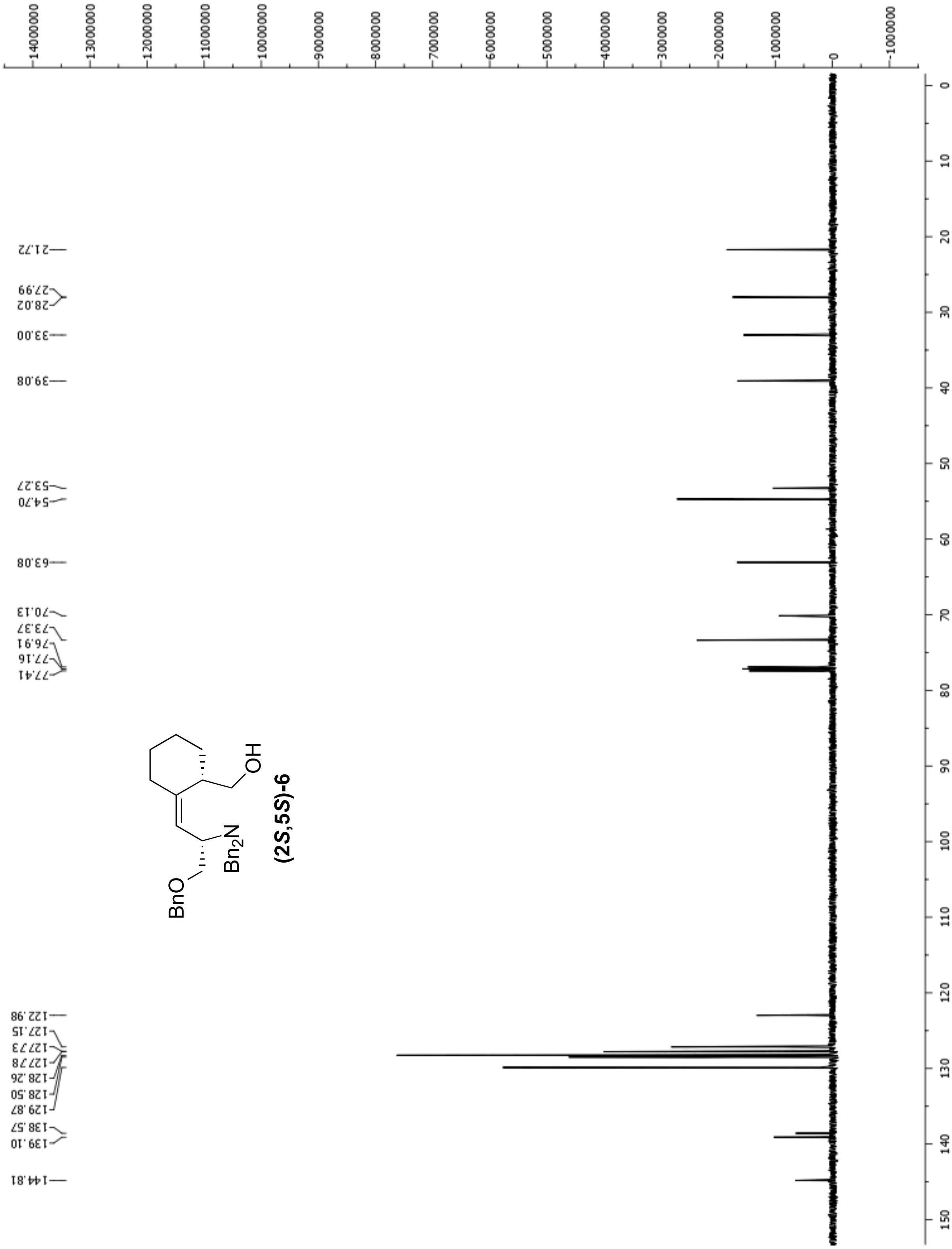

^{13}C NMR of **(2*S*,5*S*)-6** in $CDCl_3$ (125 MHz)

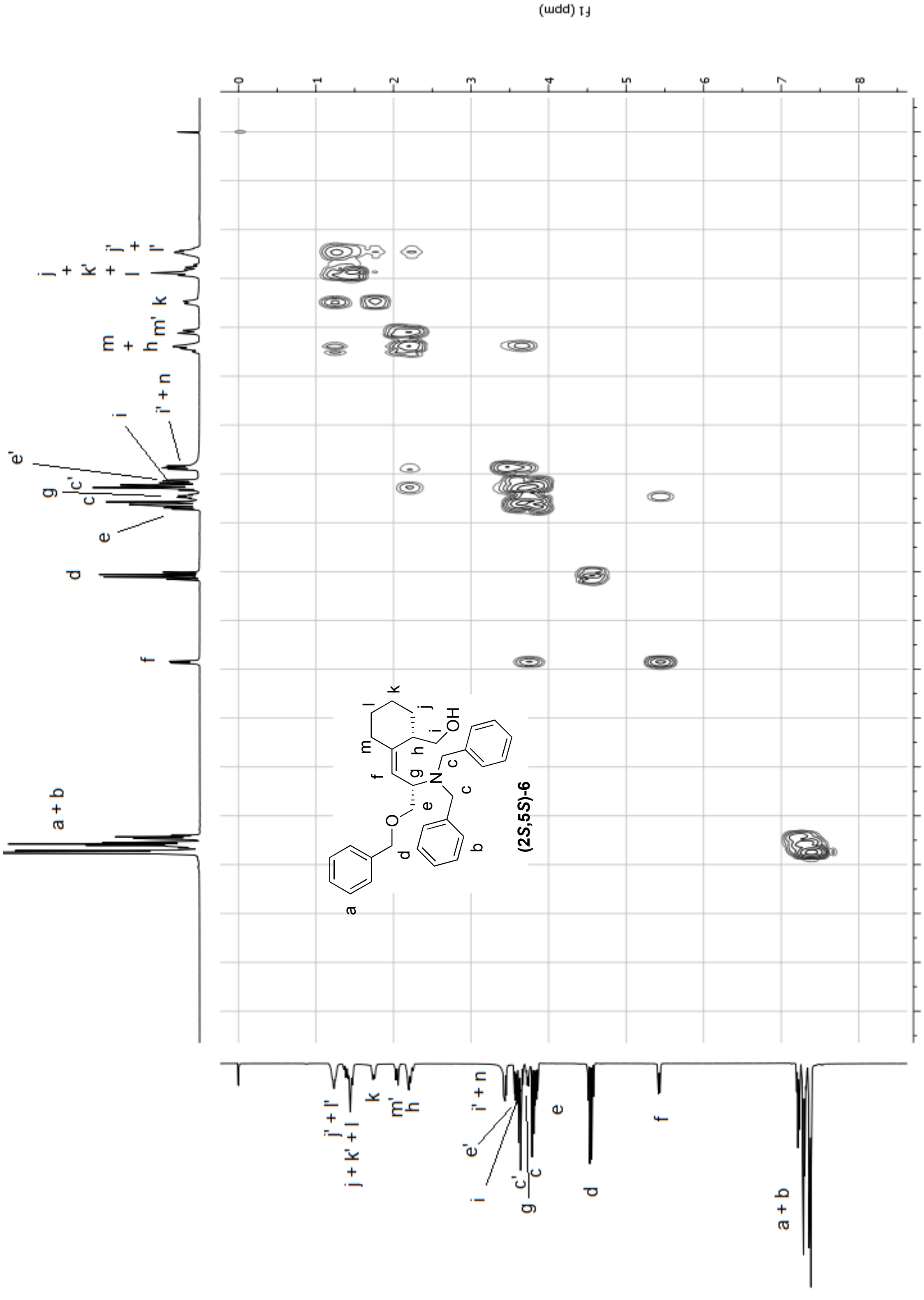

COSY of **(2*S*,5*S*)-6** in $CDCl_3$ (500 MHz)

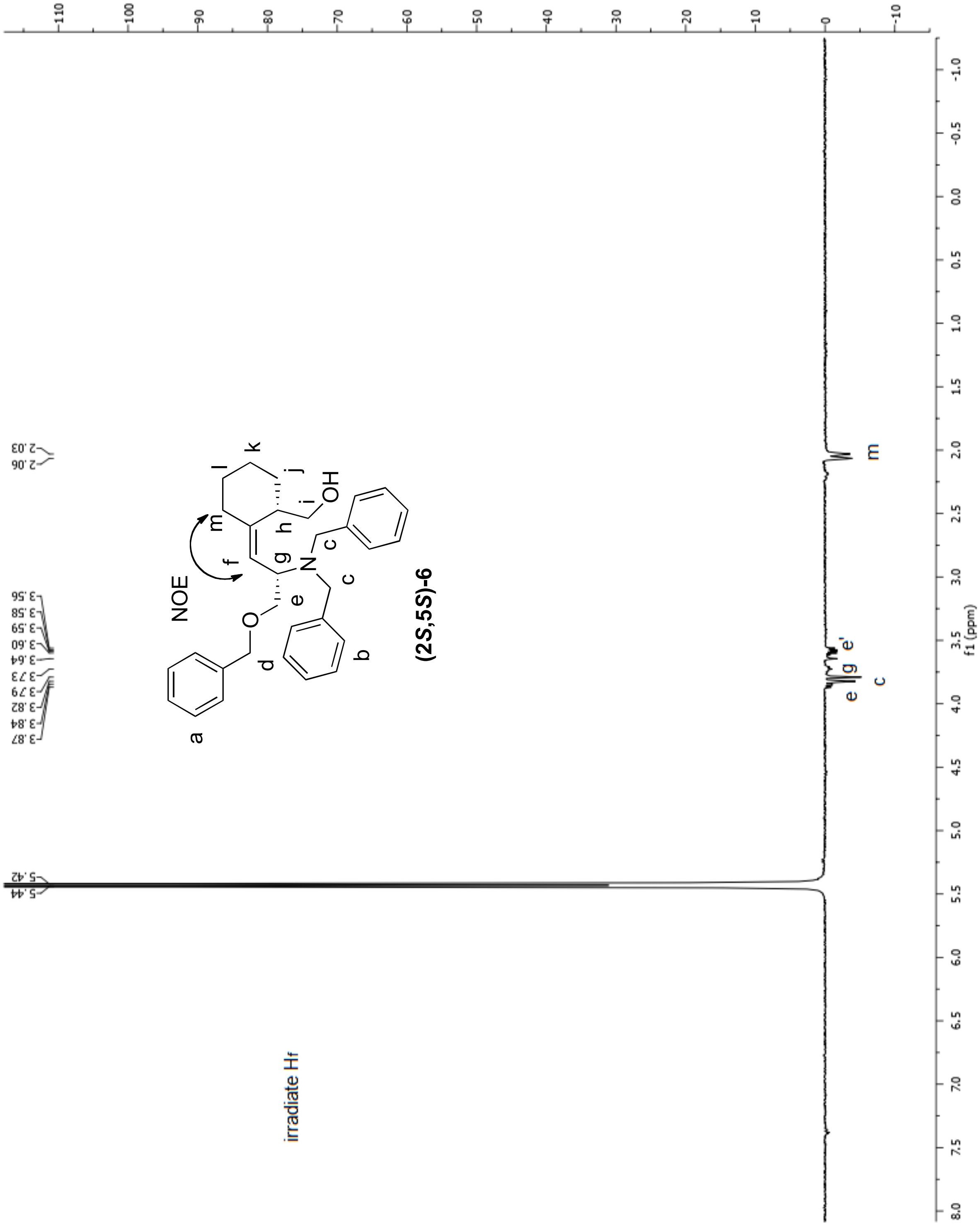

1D nOe of **(2*S*,5*S*)-6** in $CDCl_3$ (400 MHz)

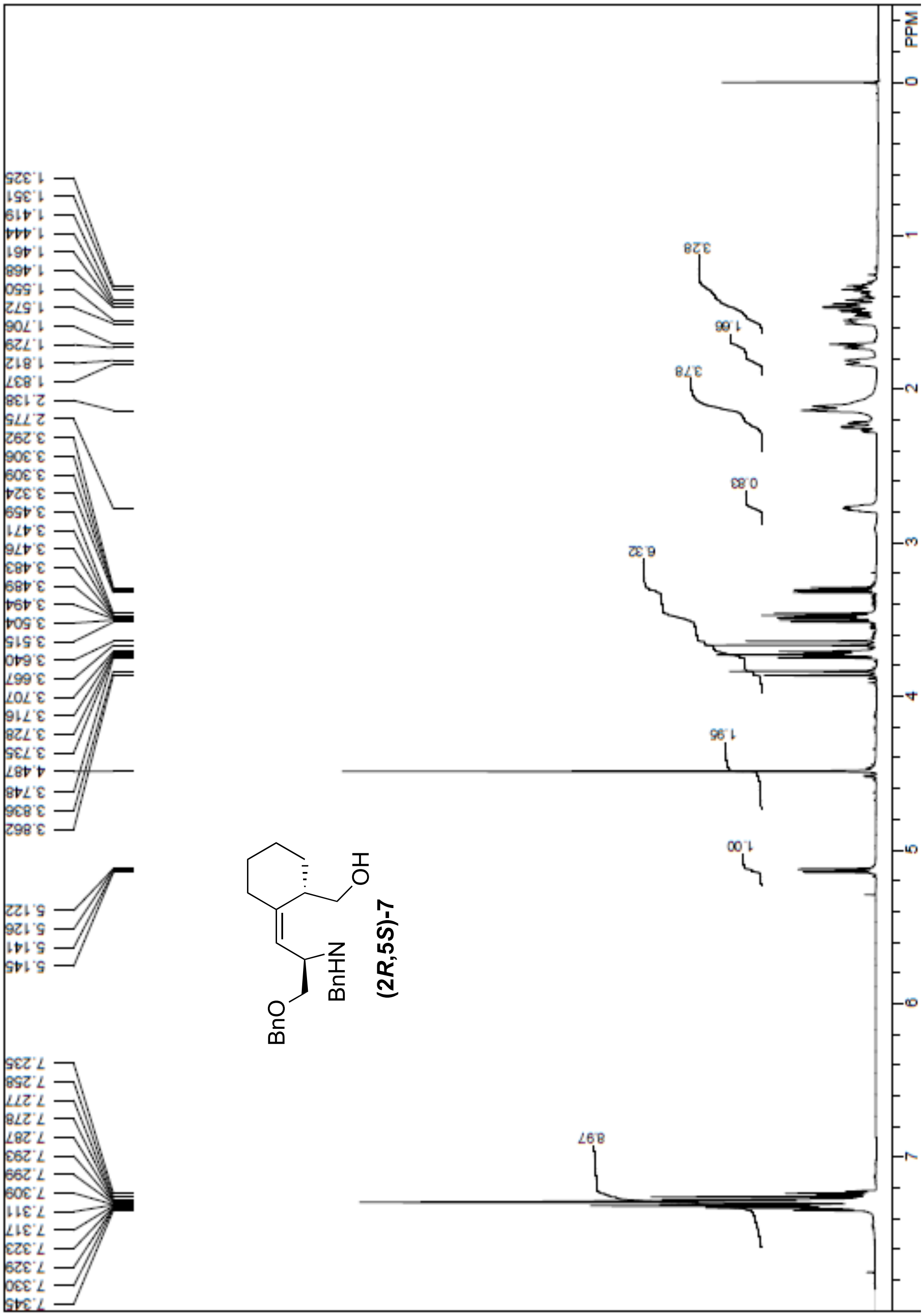

^{1}H NMR of **(2*R*,5*S*)-7** in $CDCl_3$ (500 MHz)

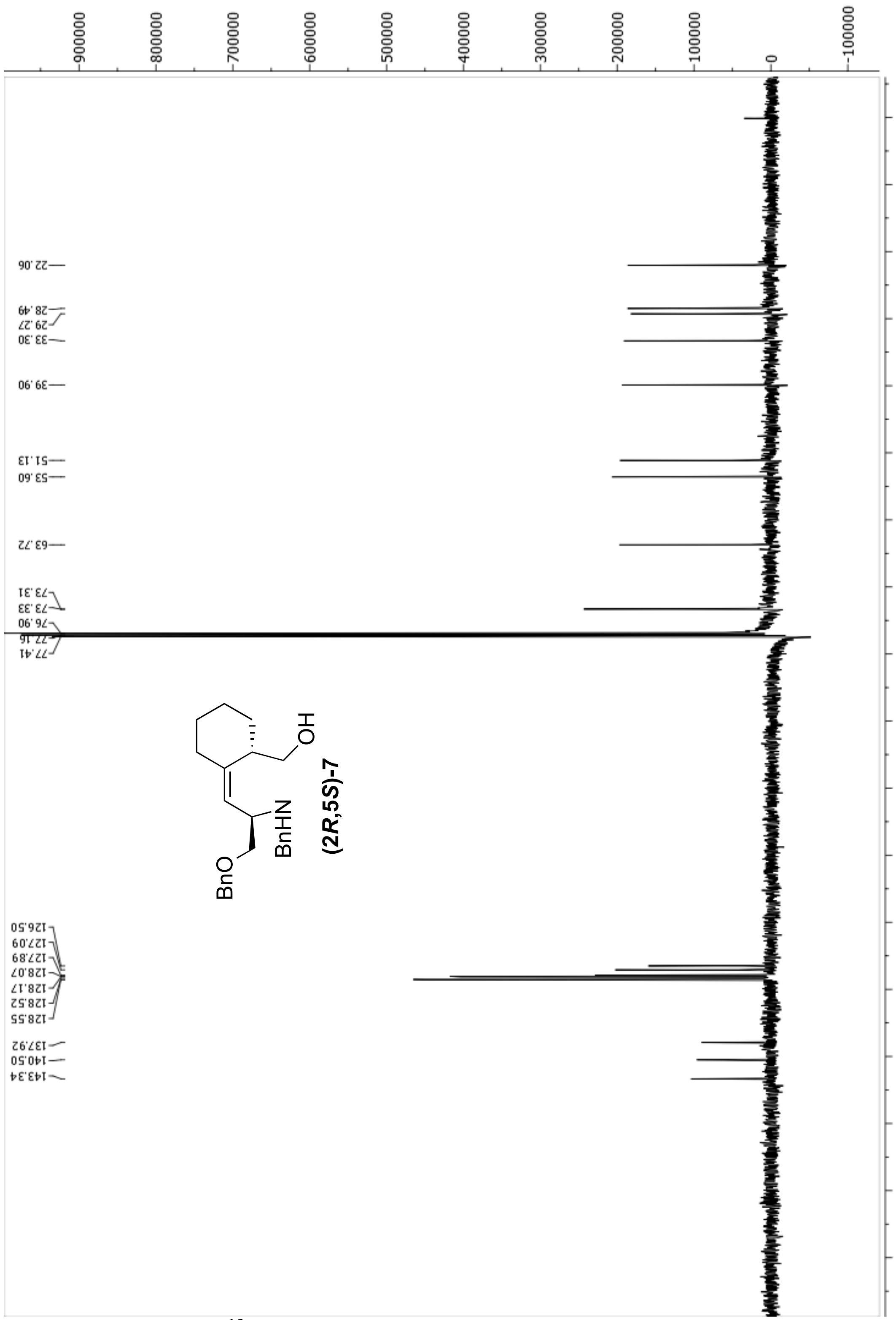

^{13}C NMR of **(2*R*,5*S*)-7** in $CDCl_3$ (125 MHz)

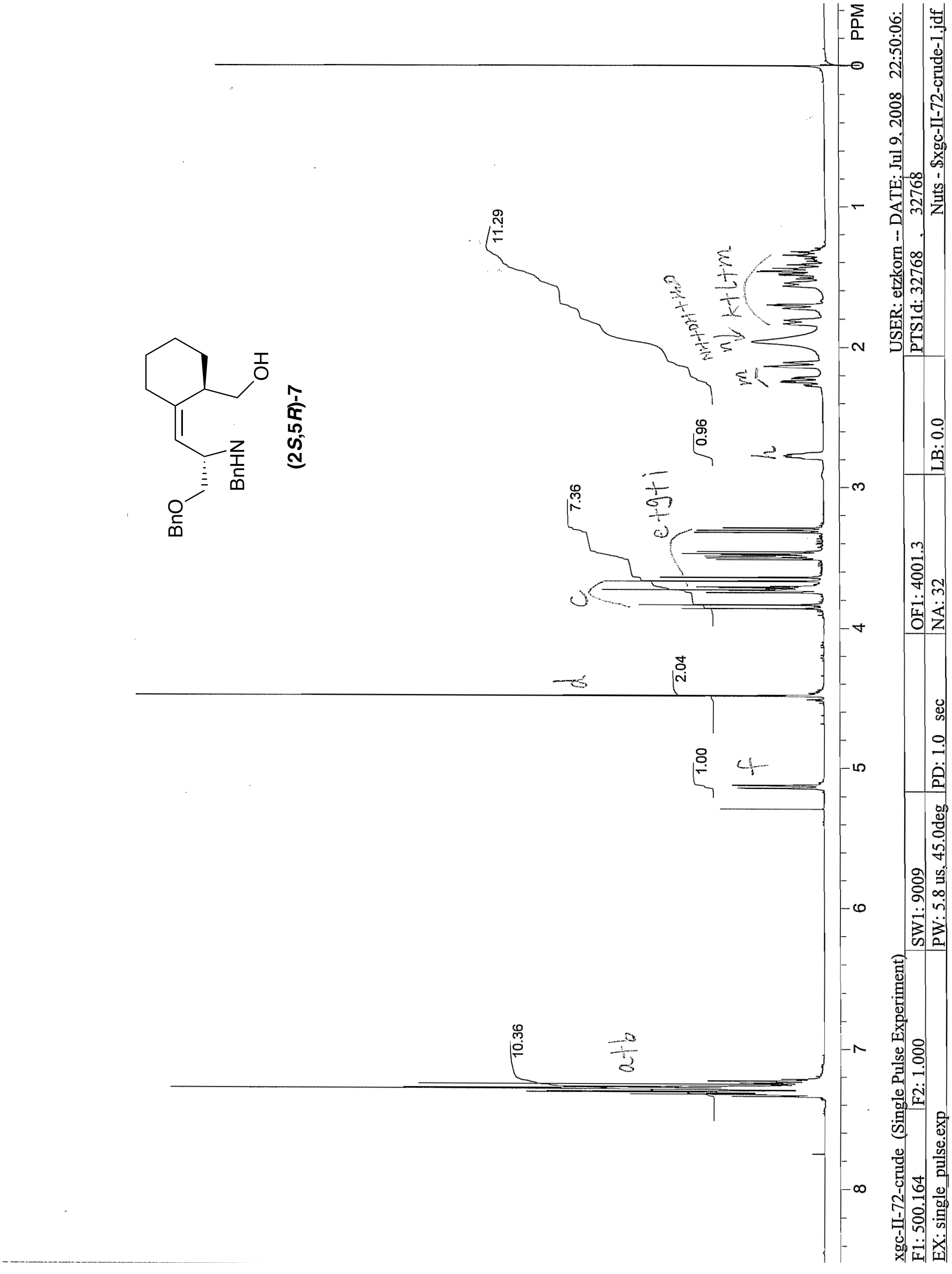

^{1}H NMR of **(2*S*,5*R*)-7** in $CDCl_3$ (500 MHz)

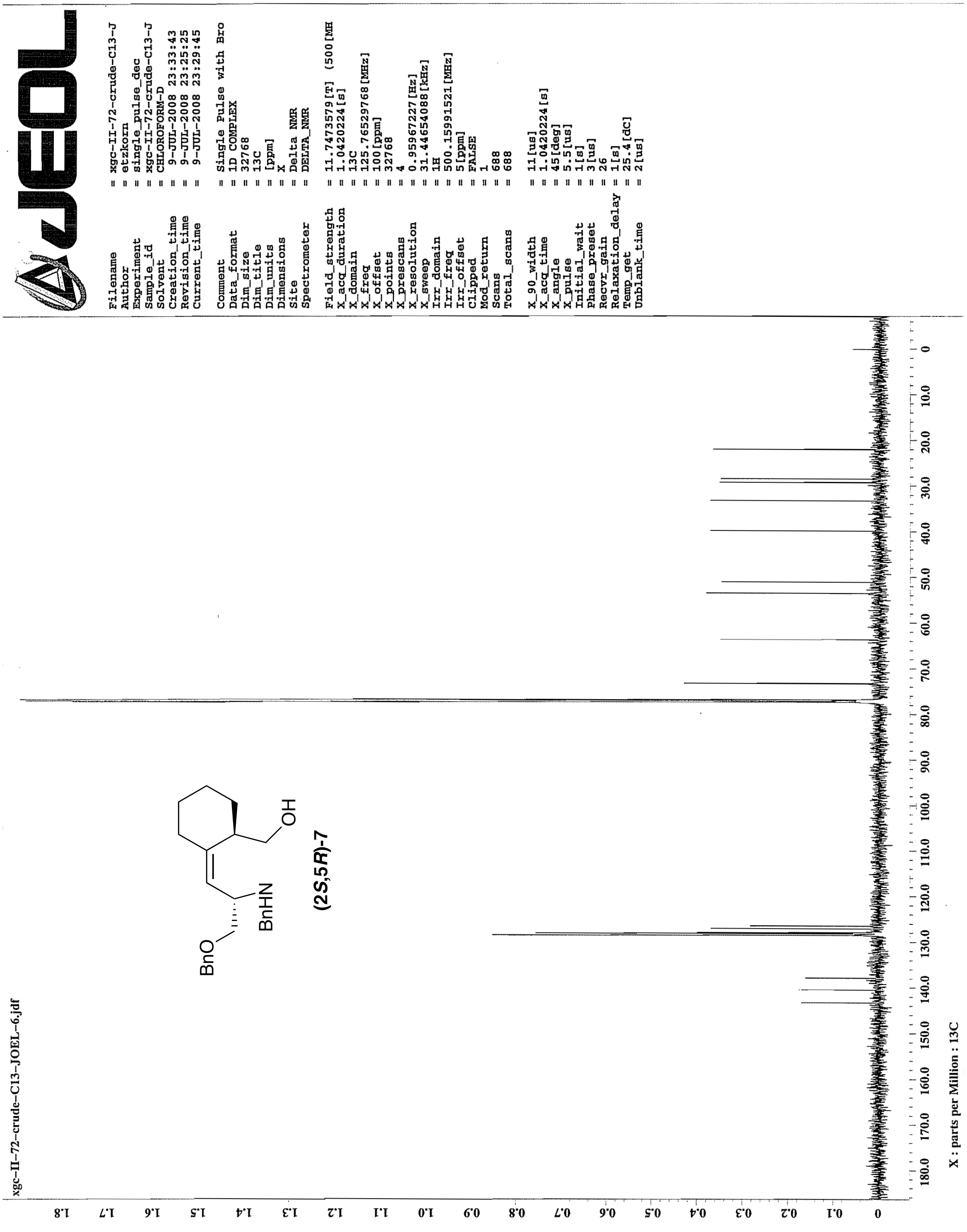

^{13}C NMR of **(2*S*,5*R*)-7** in $CDCl_3$ (125 MHz)

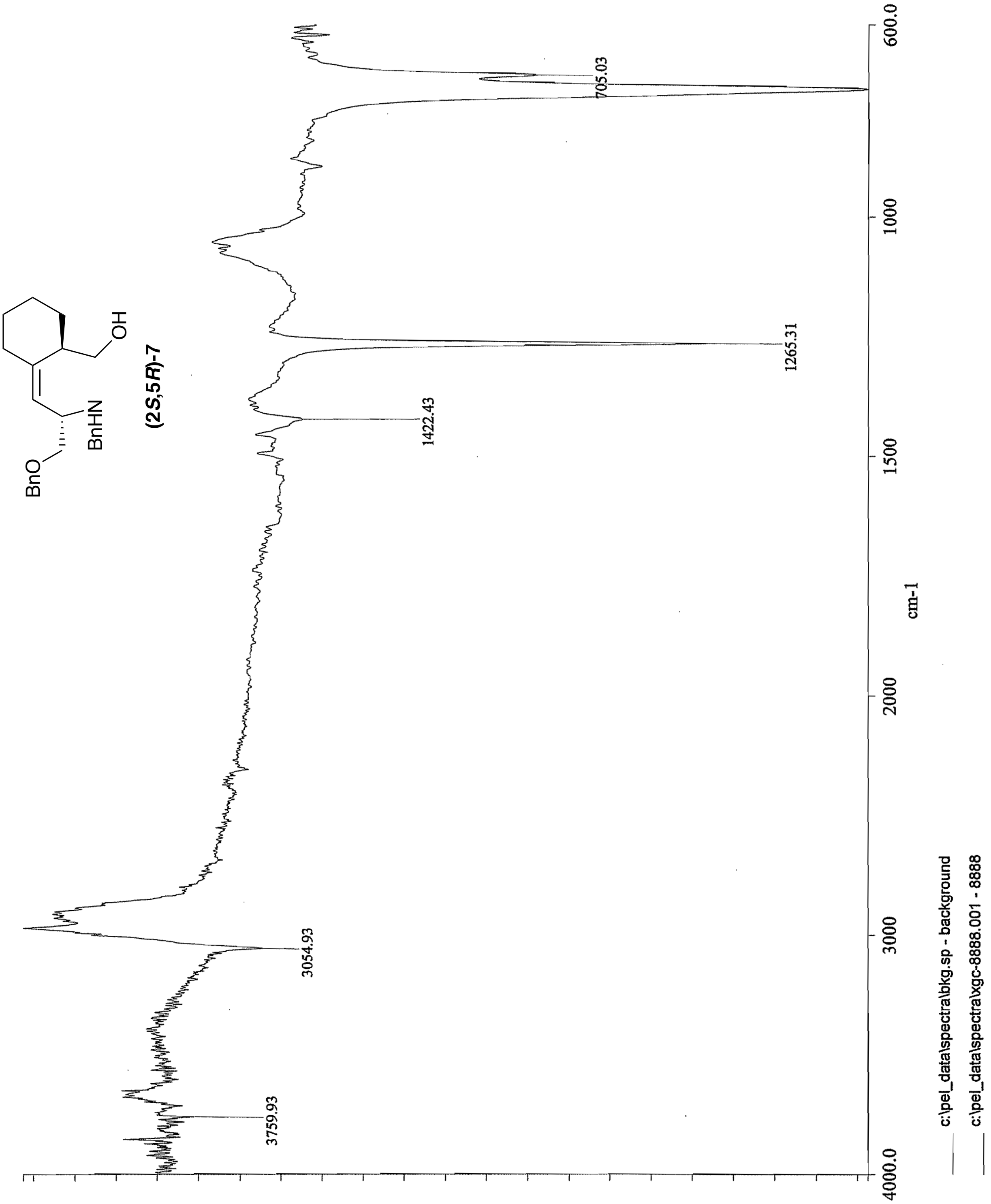

FTIR of **(2*S*,5*R*)-7** neat

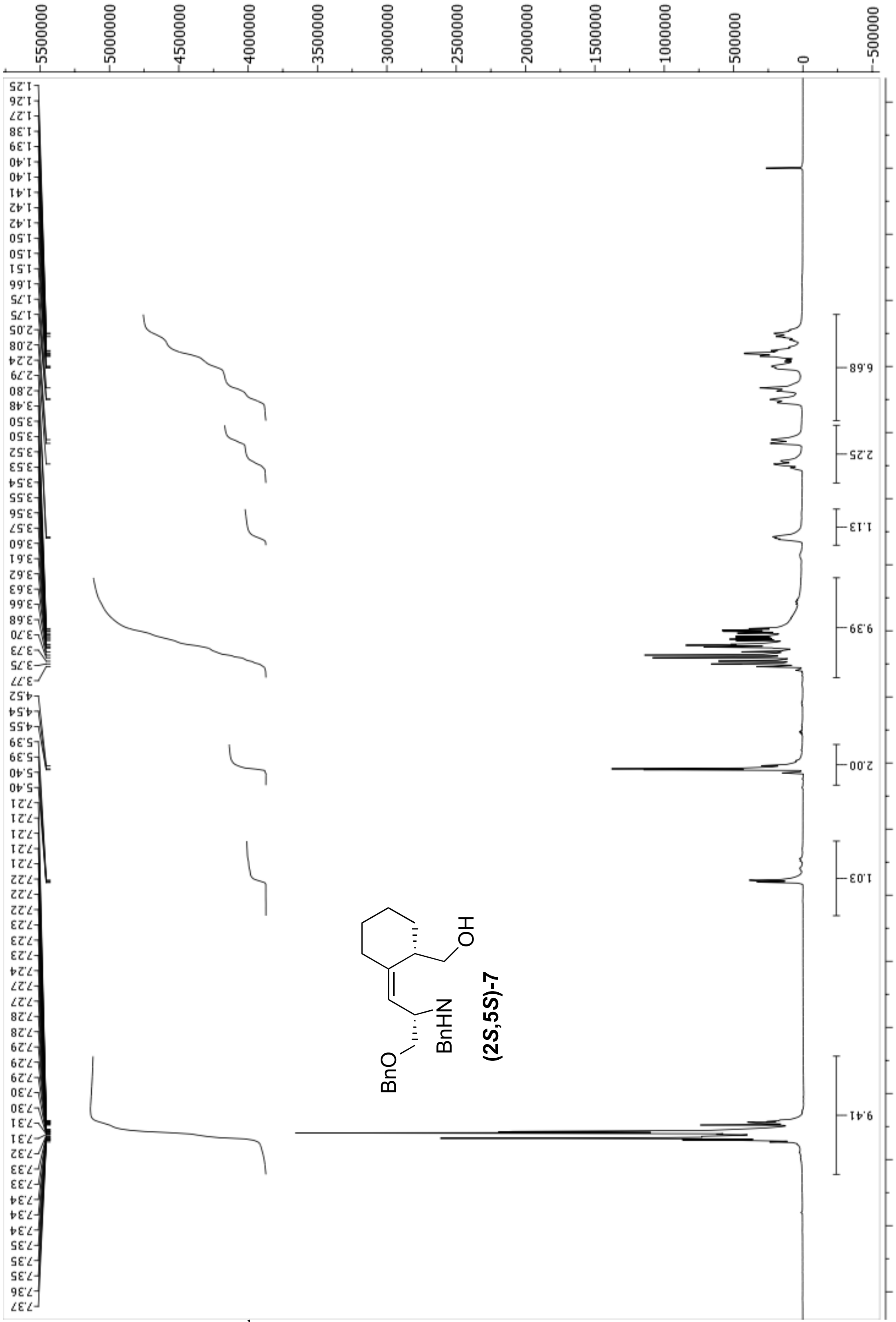

^{1}H NMR of **(2*S*,5*S*)-7** in $CDCl_3$ (500 MHz)

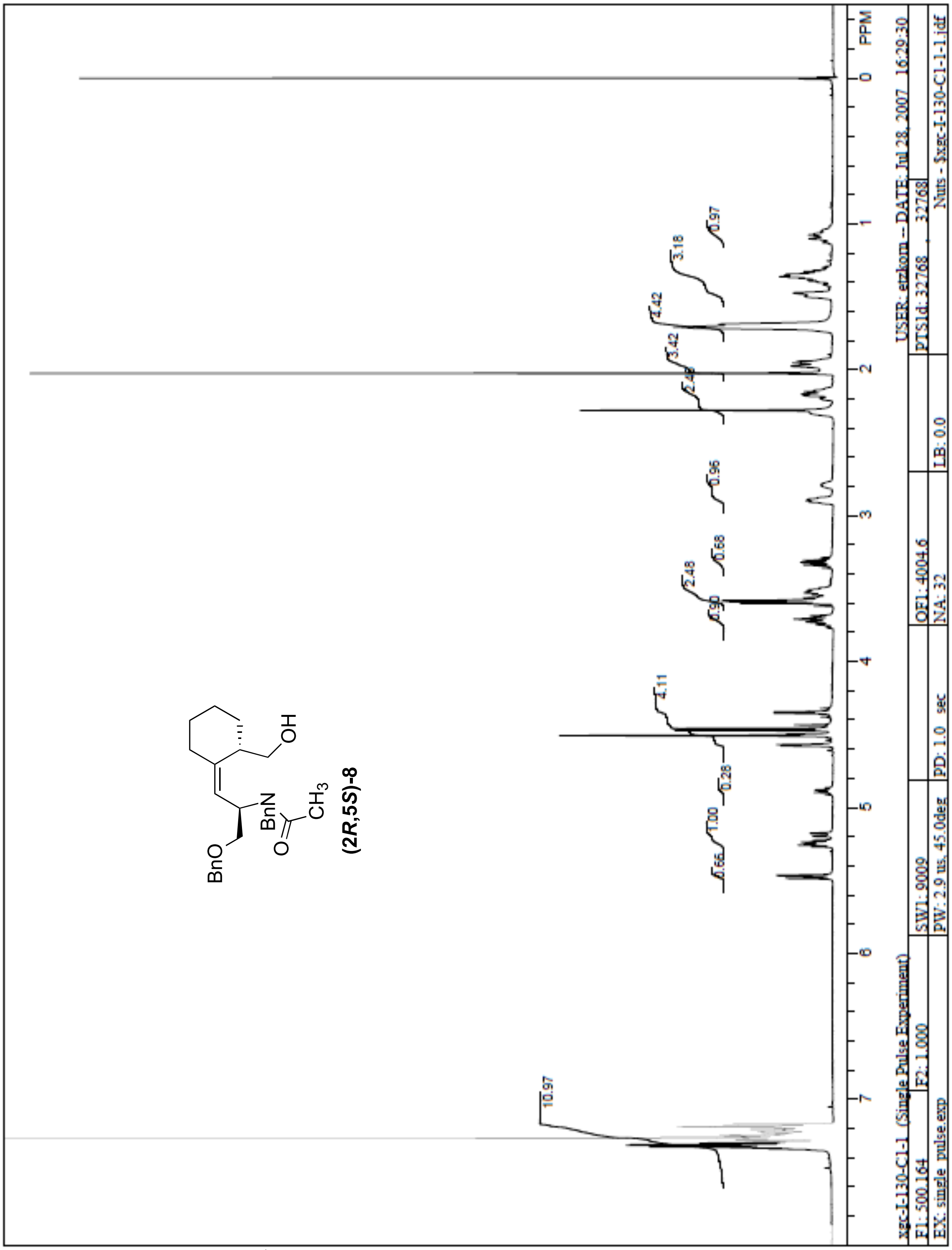

^{1}H NMR of **(2*R*,5*S*)-8** in $CDCl_3$ (500 MHz)

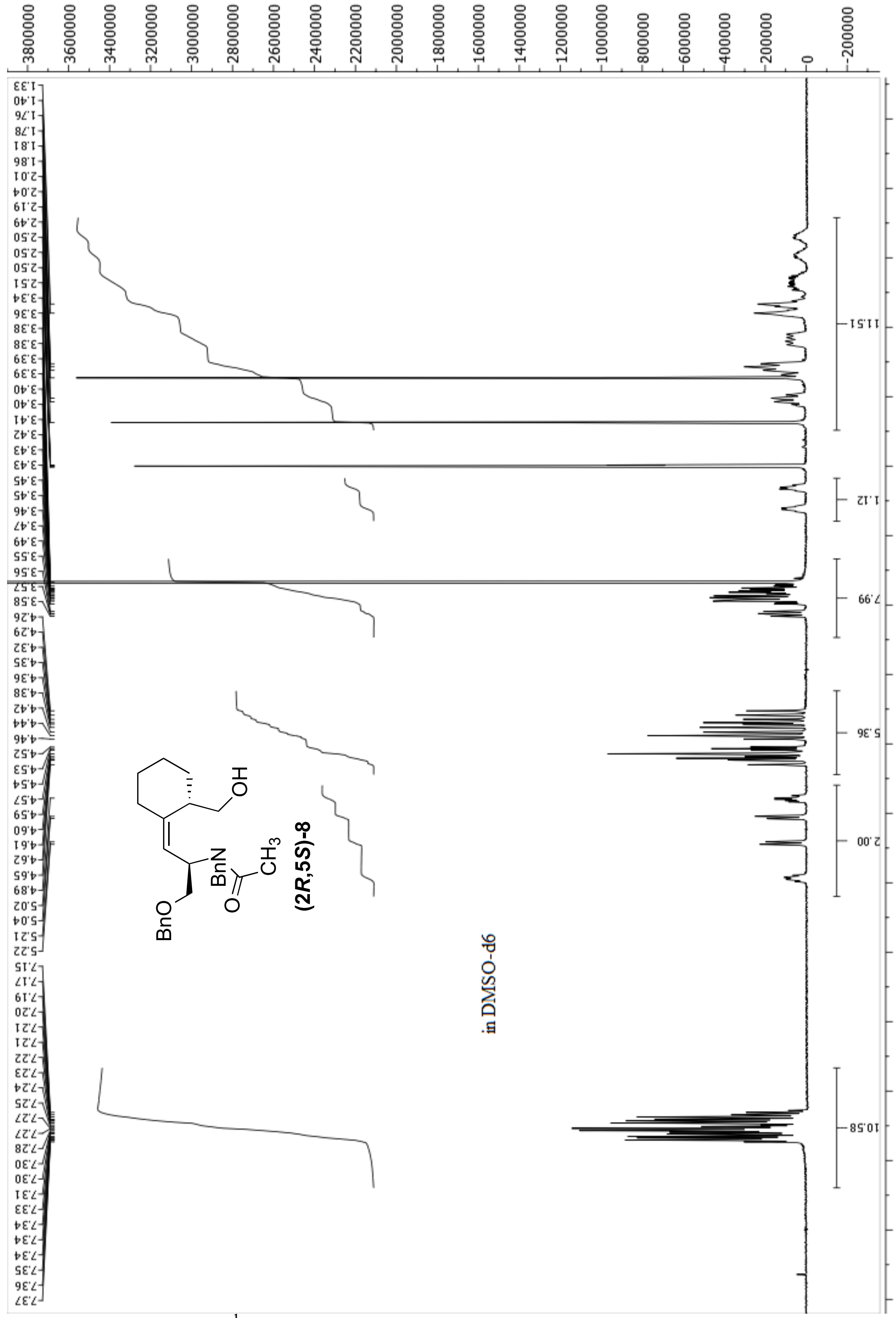

^{1}H NMR of **(2*R*,5*S*)-8** in DMSO-d_6 (500 MHz)

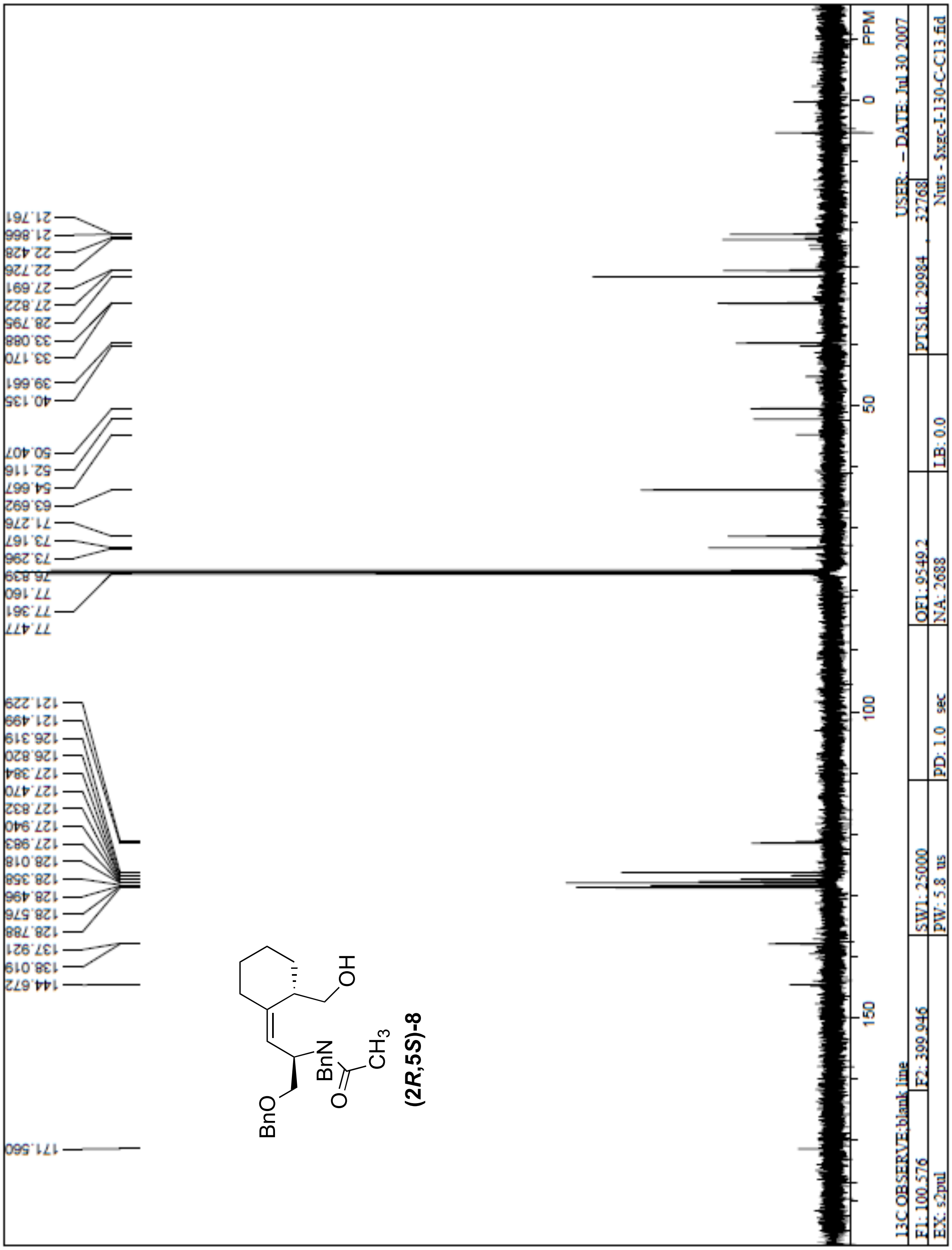

^{13}C NMR of **(2*R*,5*S*)-8** in $CDCl_3$ (125 MHz)

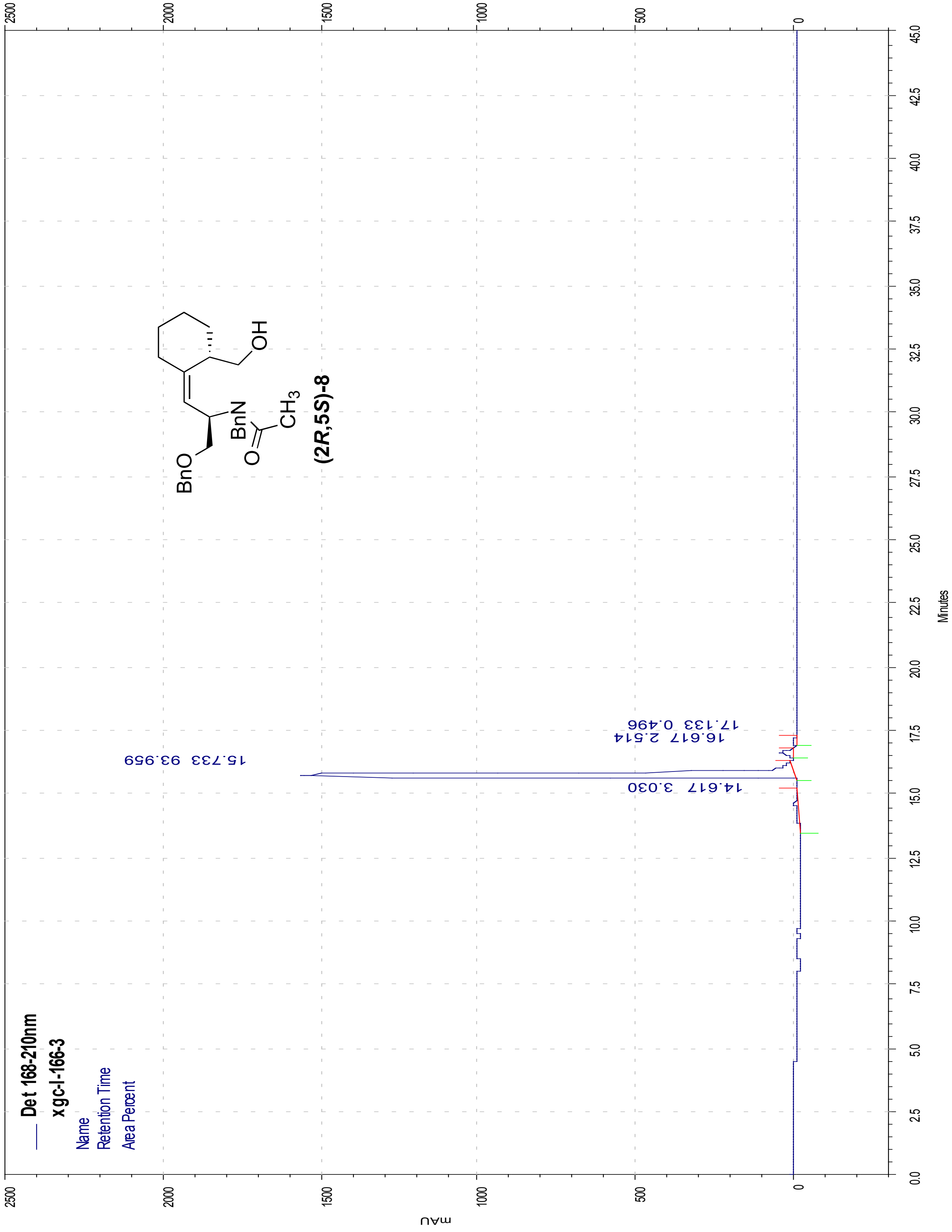

Analytical HPLC of **(2*R*,5*S*)-8**

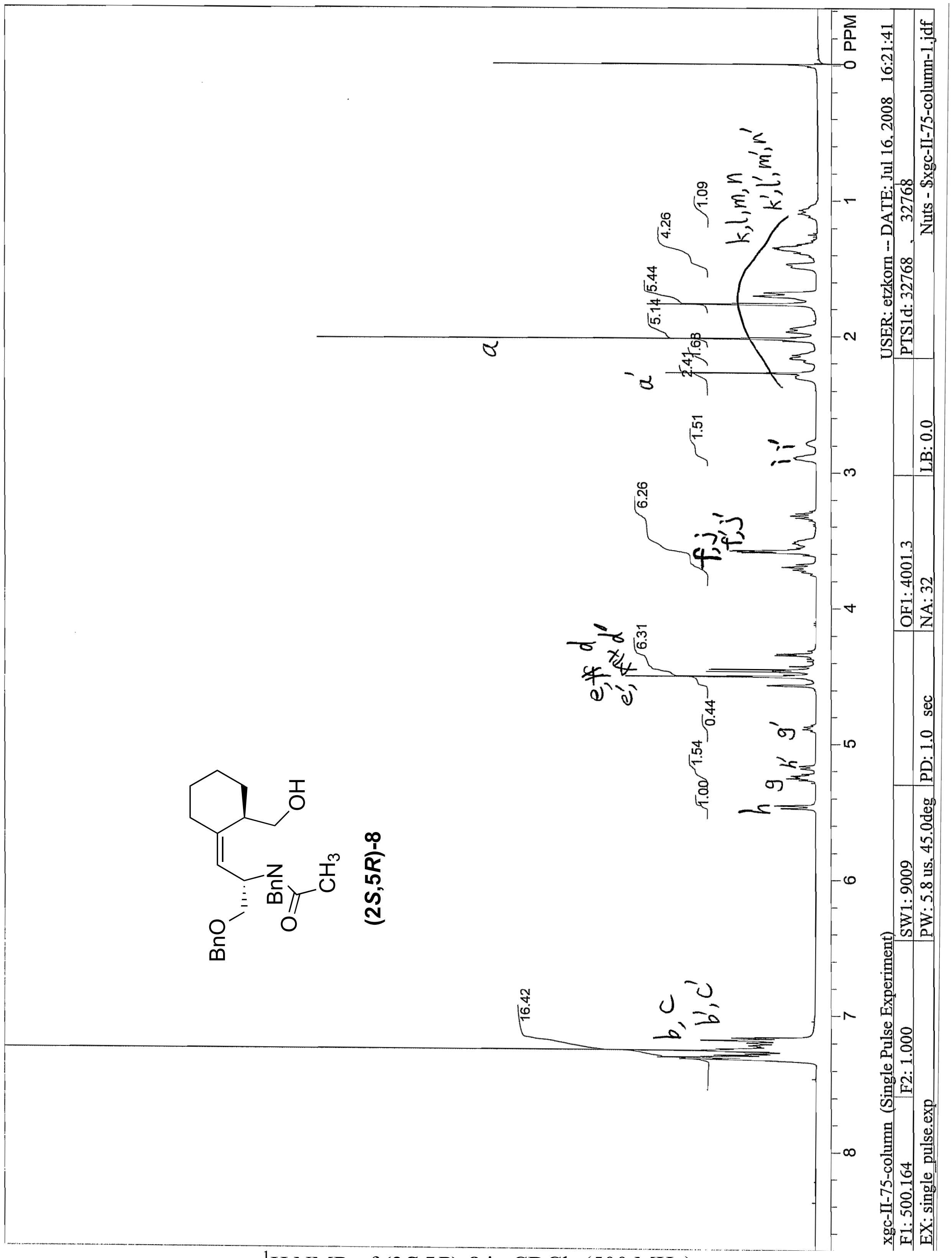

^{1}H NMR of **(2*S*,5*R*)-8** in $CDCl_3$ (500 MHz)

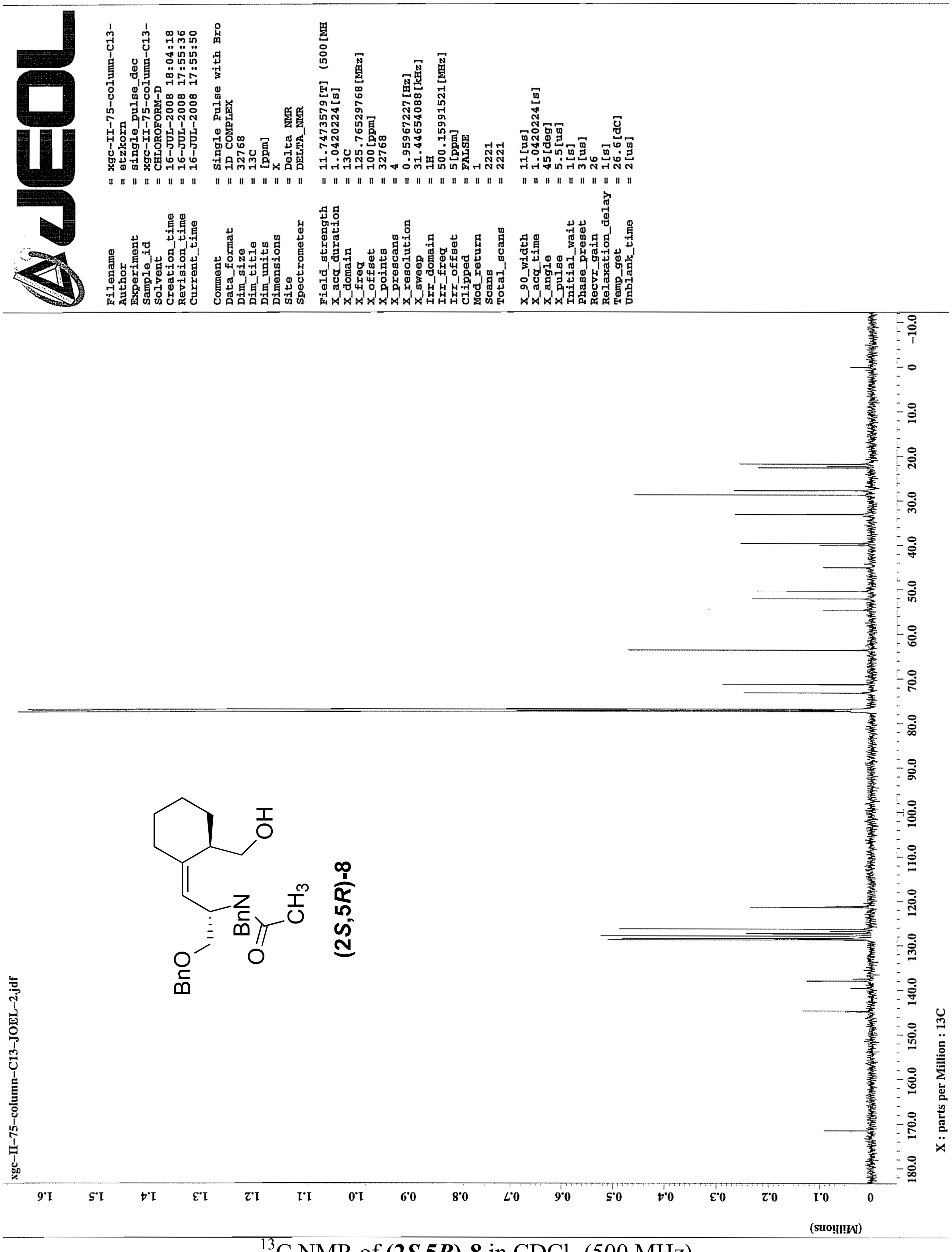

^{13}C NMR of **(2*S*,5*R*)-8** in $CDCl_3$ (500 MHz)

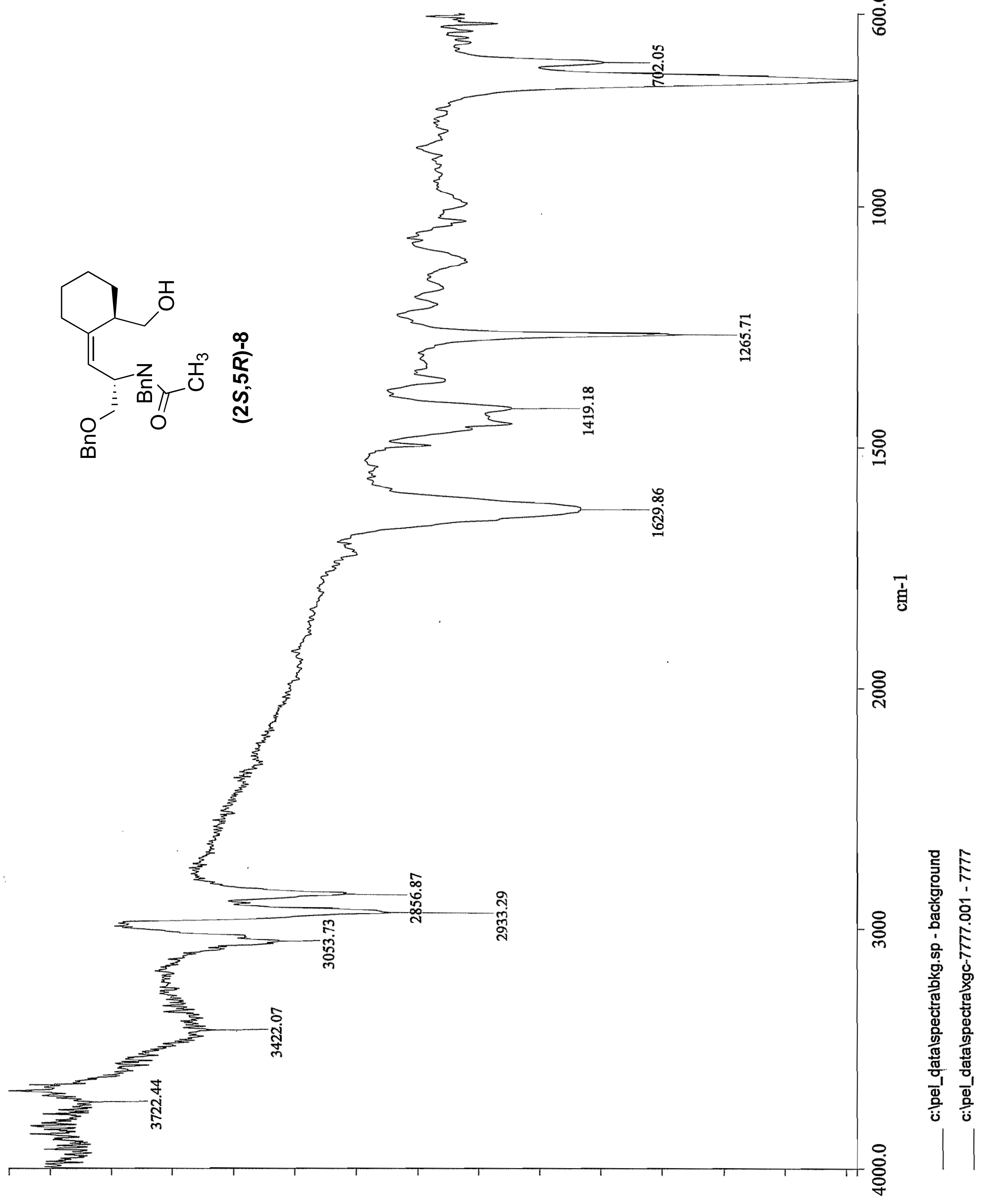

FTIR of **(2*S*,5R)-8** (neat)

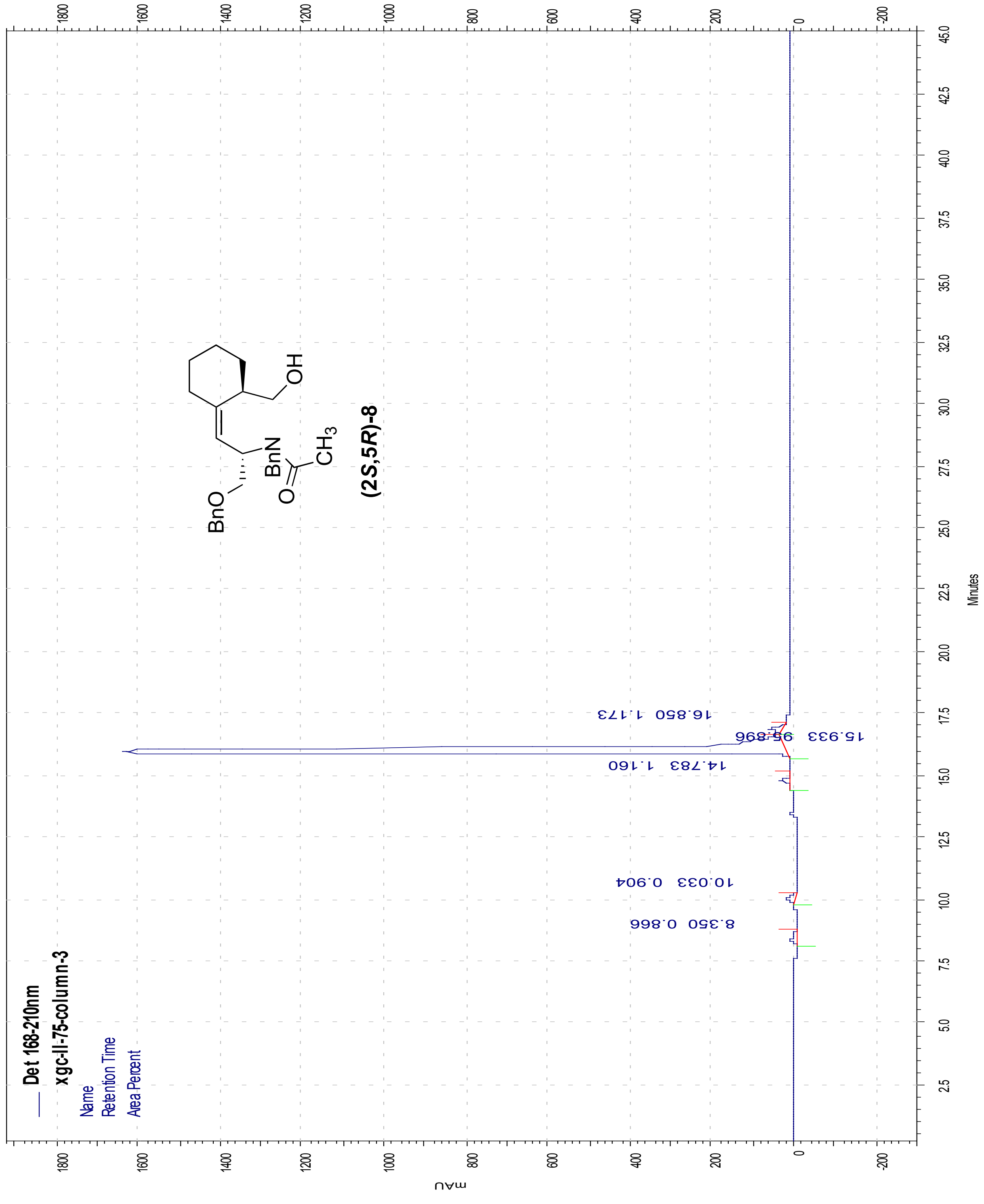

Analytical HPLC of **(2*S*,5*R*)-8**

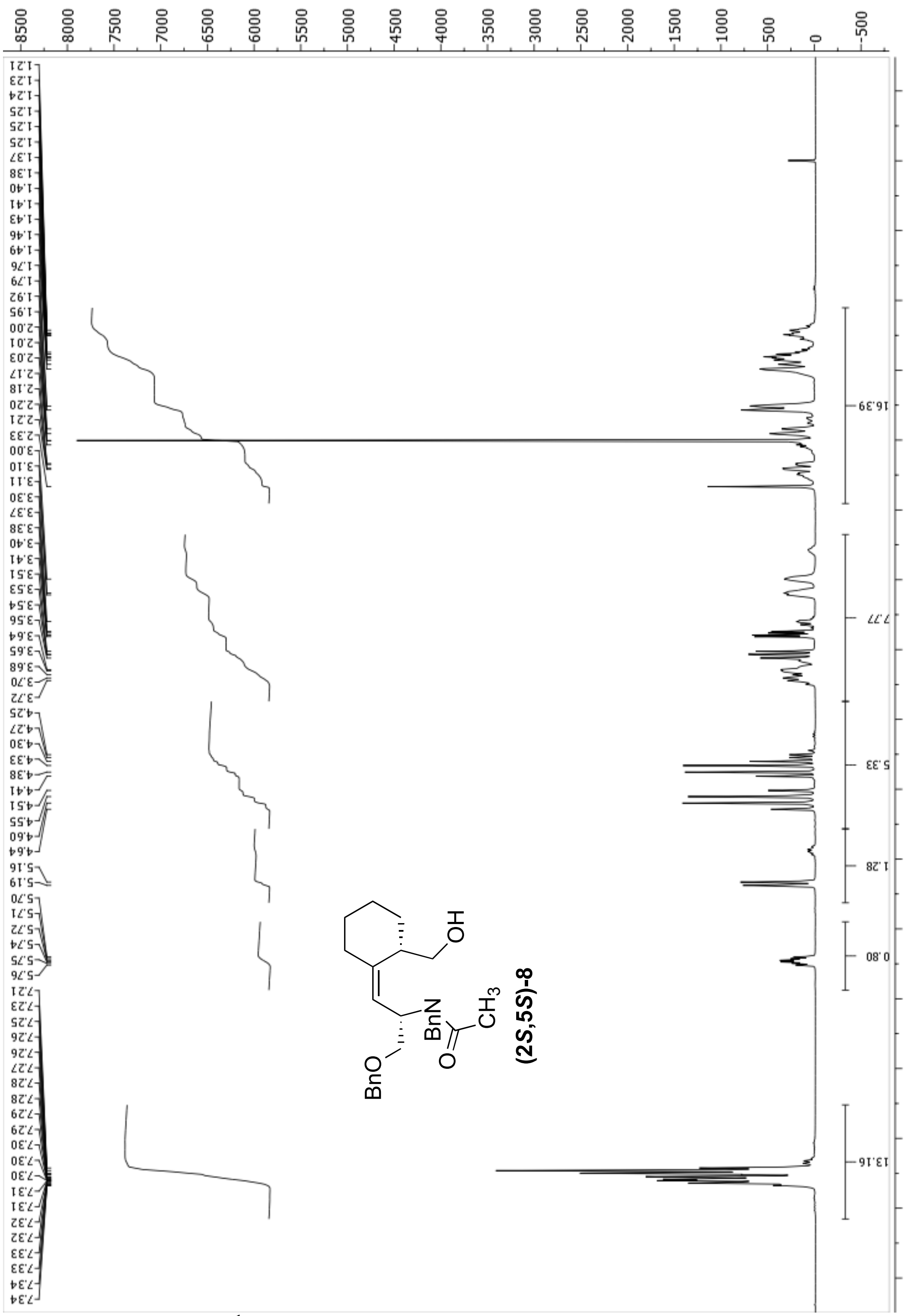

^{1}H NMR of **(2*S*,5*S*)-8** in $CDCl_3$ (400 MHz)

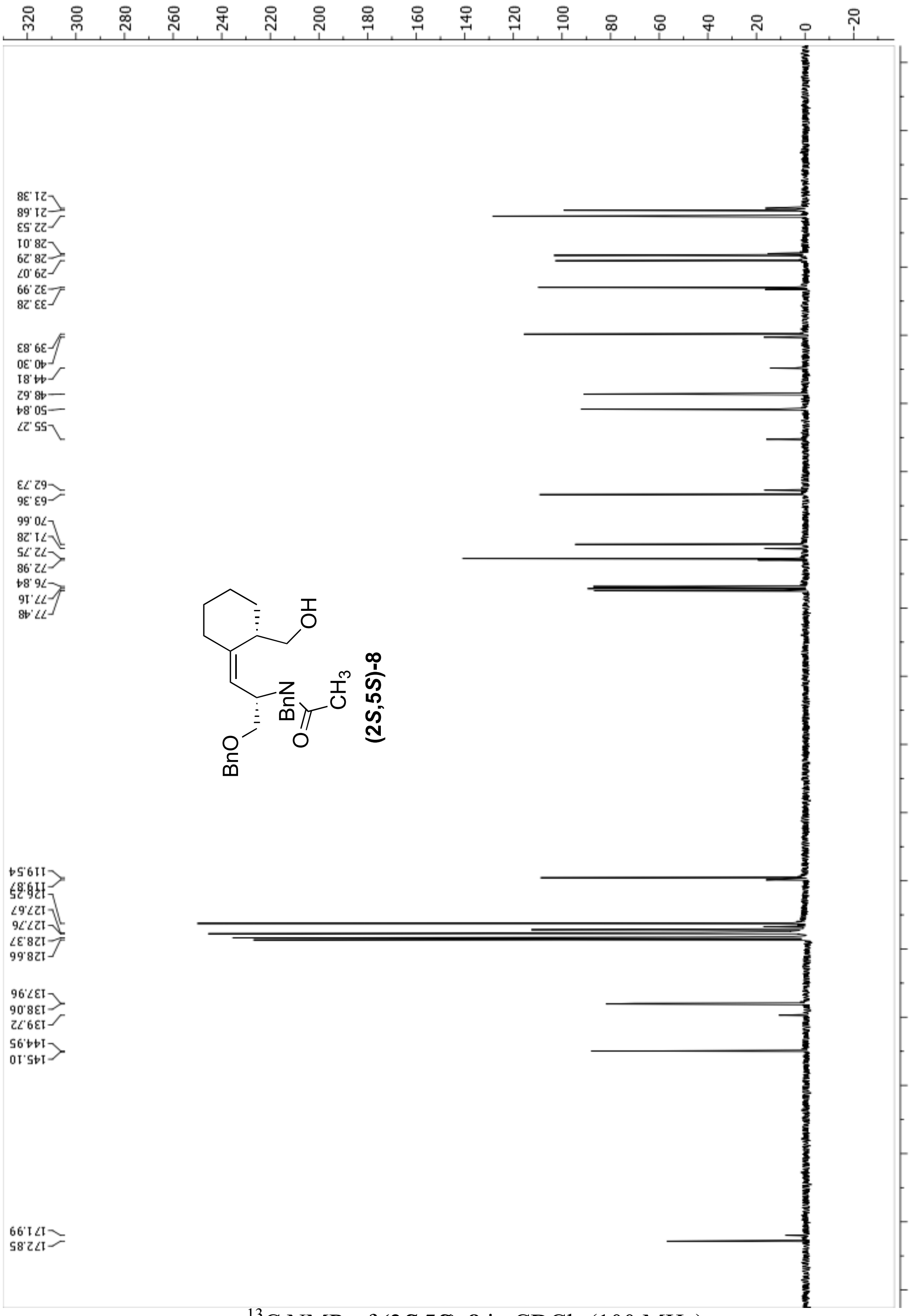

^{13}C NMR of **(2*S*,5*S*)-8** in $CDCl_3$ (100 MHz)

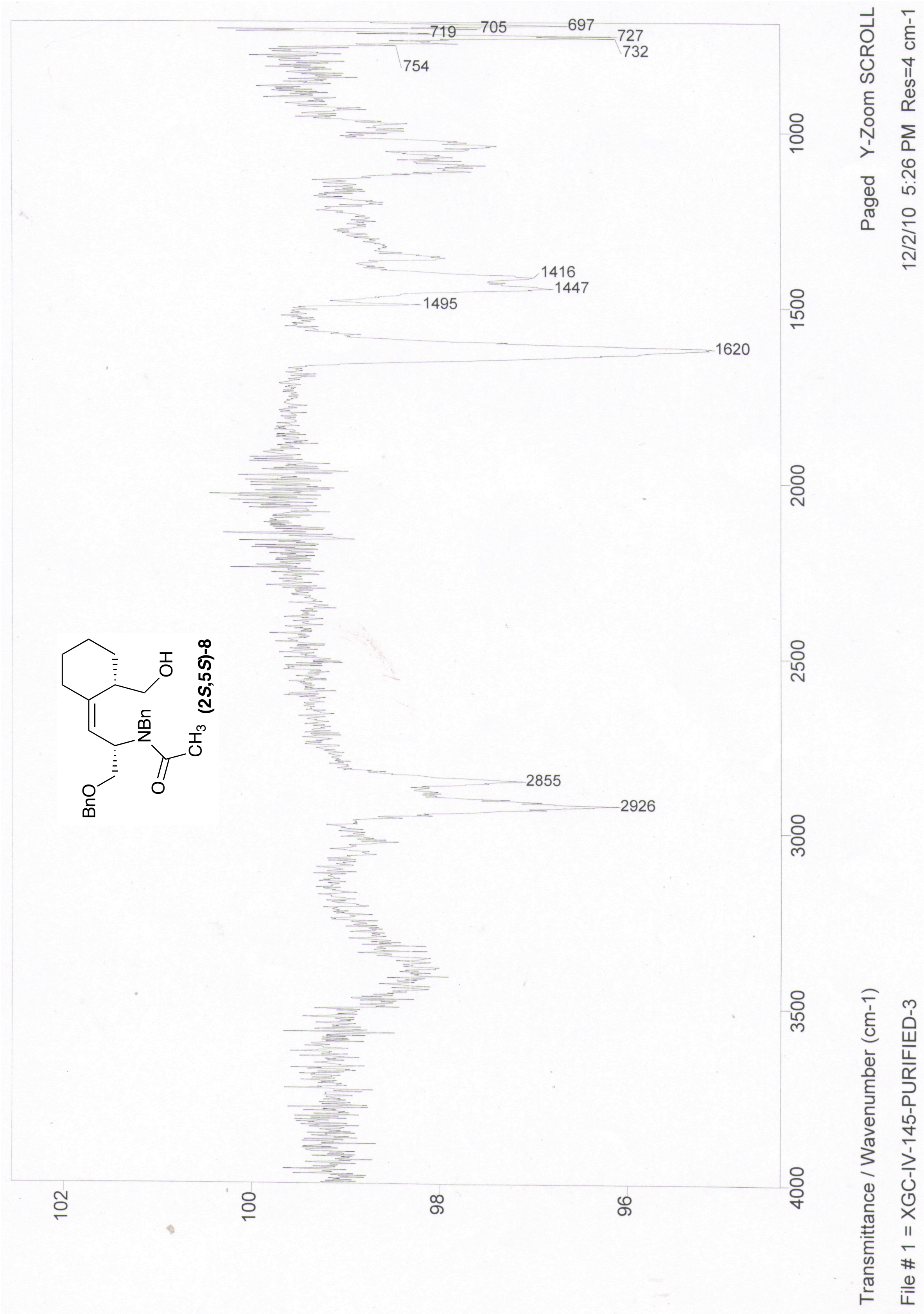

FTIR of **(2*S*,5*S*)-8** (neat)

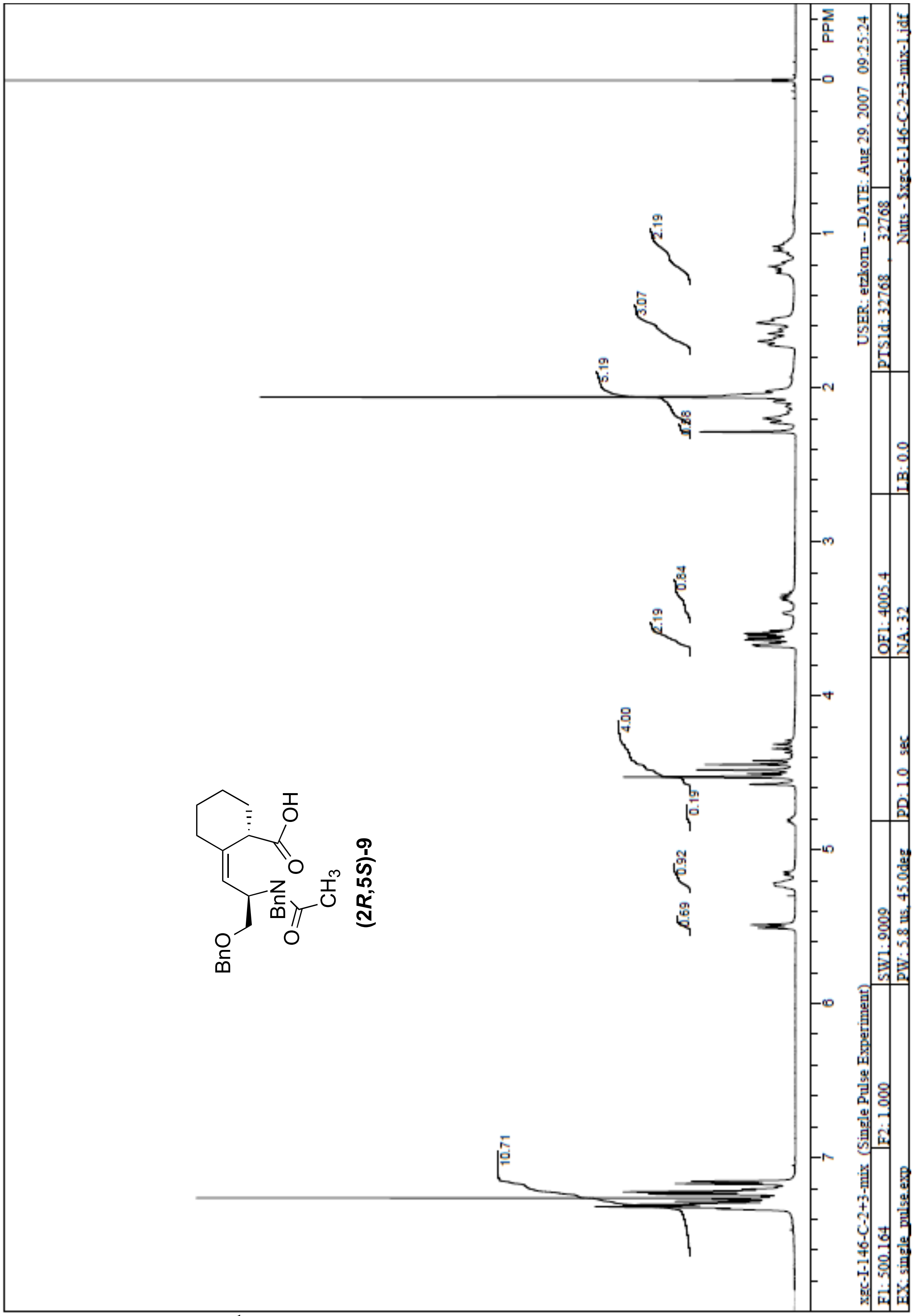

^{1}H NMR of **(2*R*,5*S*)-9** in $CDCl_3$ (500 MHz)

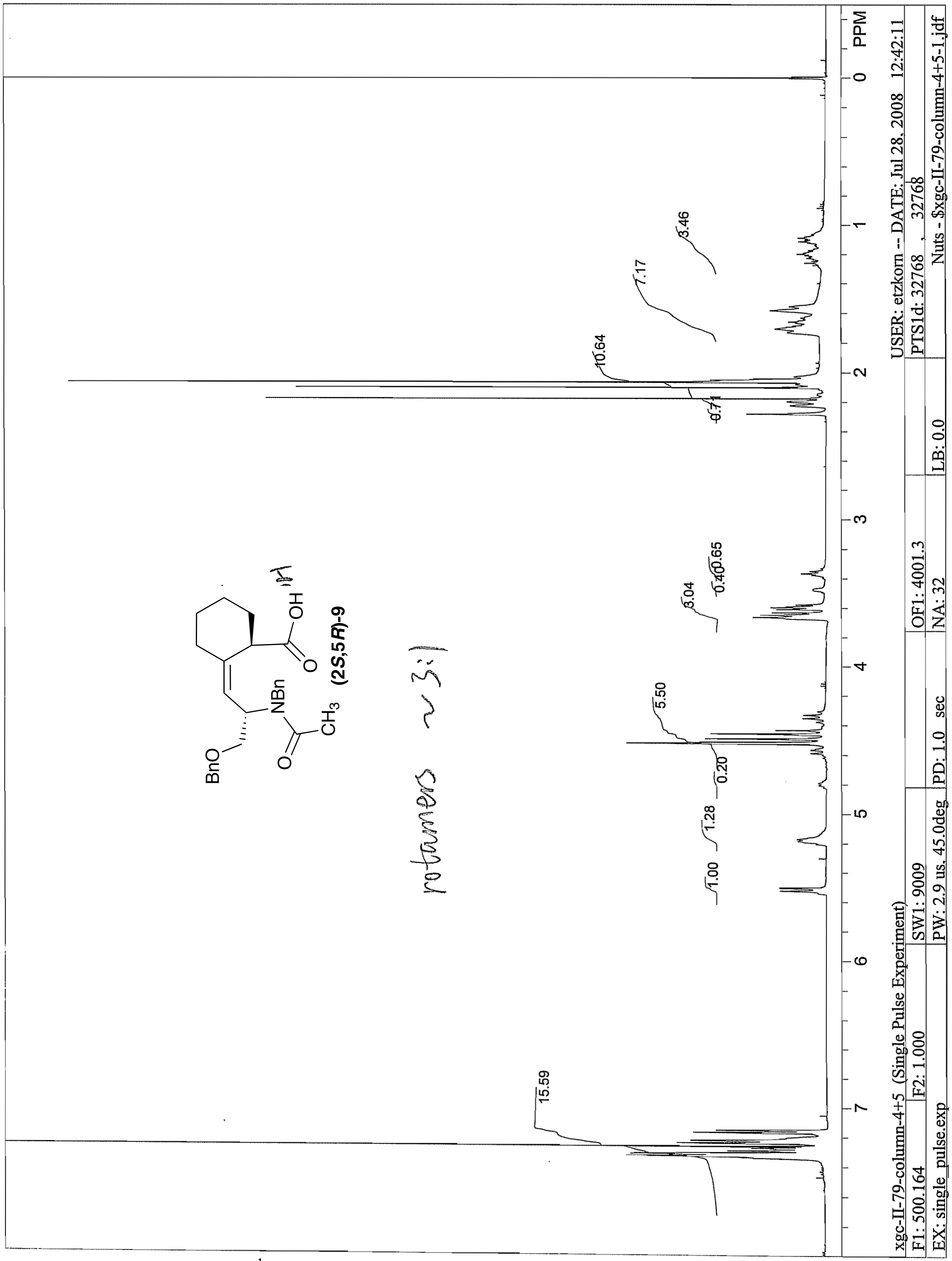

^{1}H NMR of **(2*S*,5*R*)-9** in $CDCl_3$ (500 MHz)

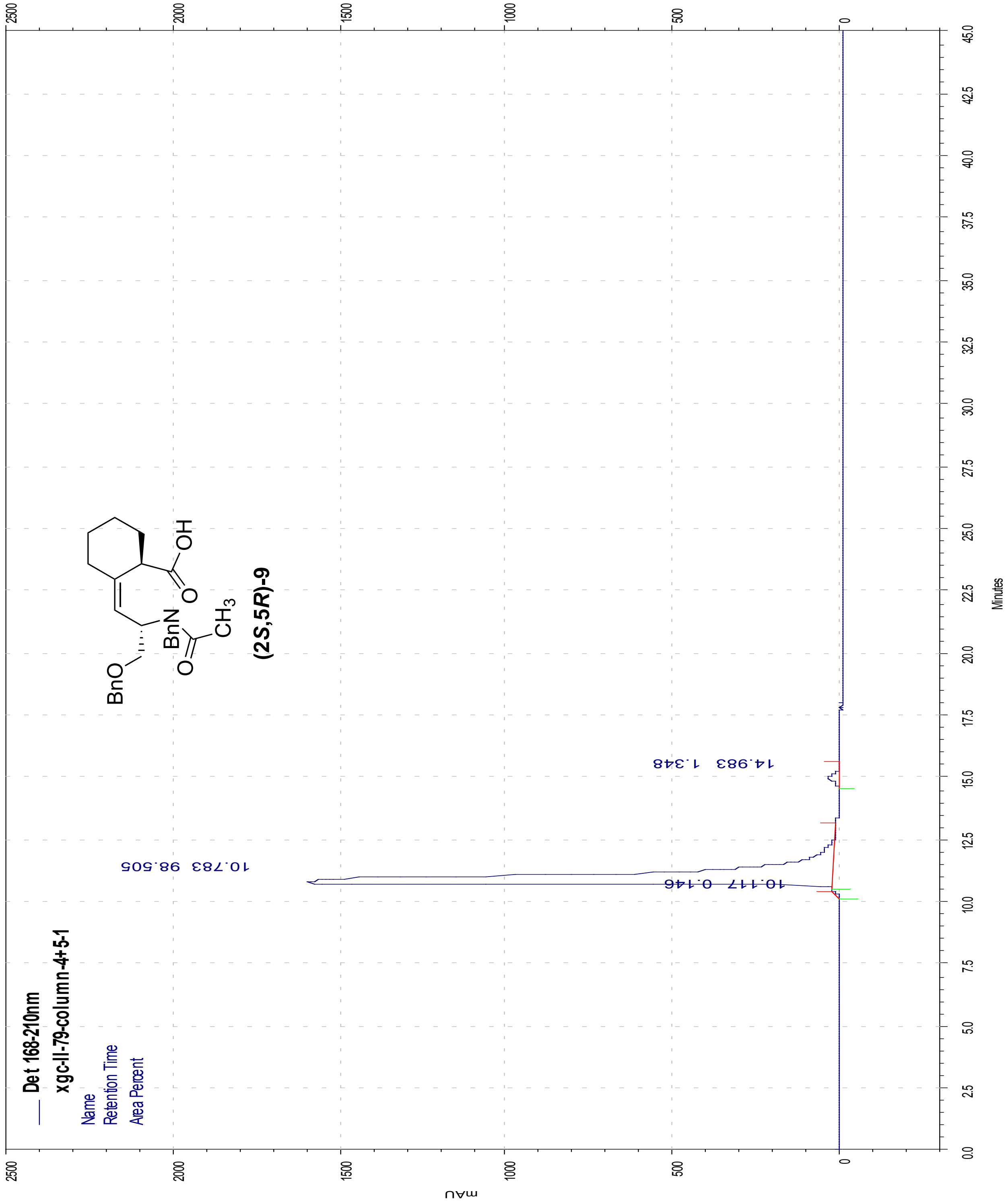

Analytical HPLC of **(2*S*,5*R*)-9**

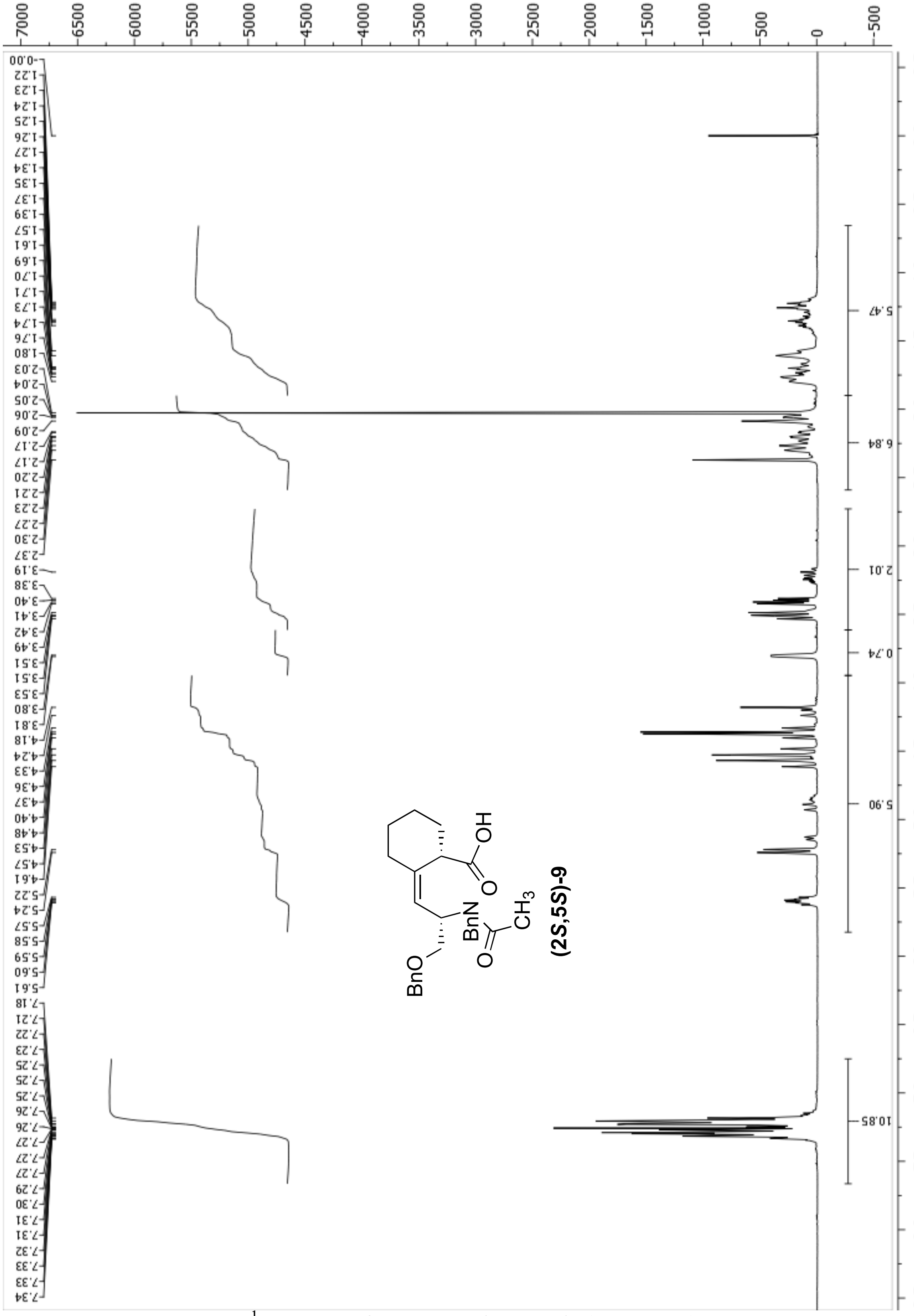

^{1}H NMR of **(2*S*,5*S*)-9** in $CDCl_3$ (400 MHz)

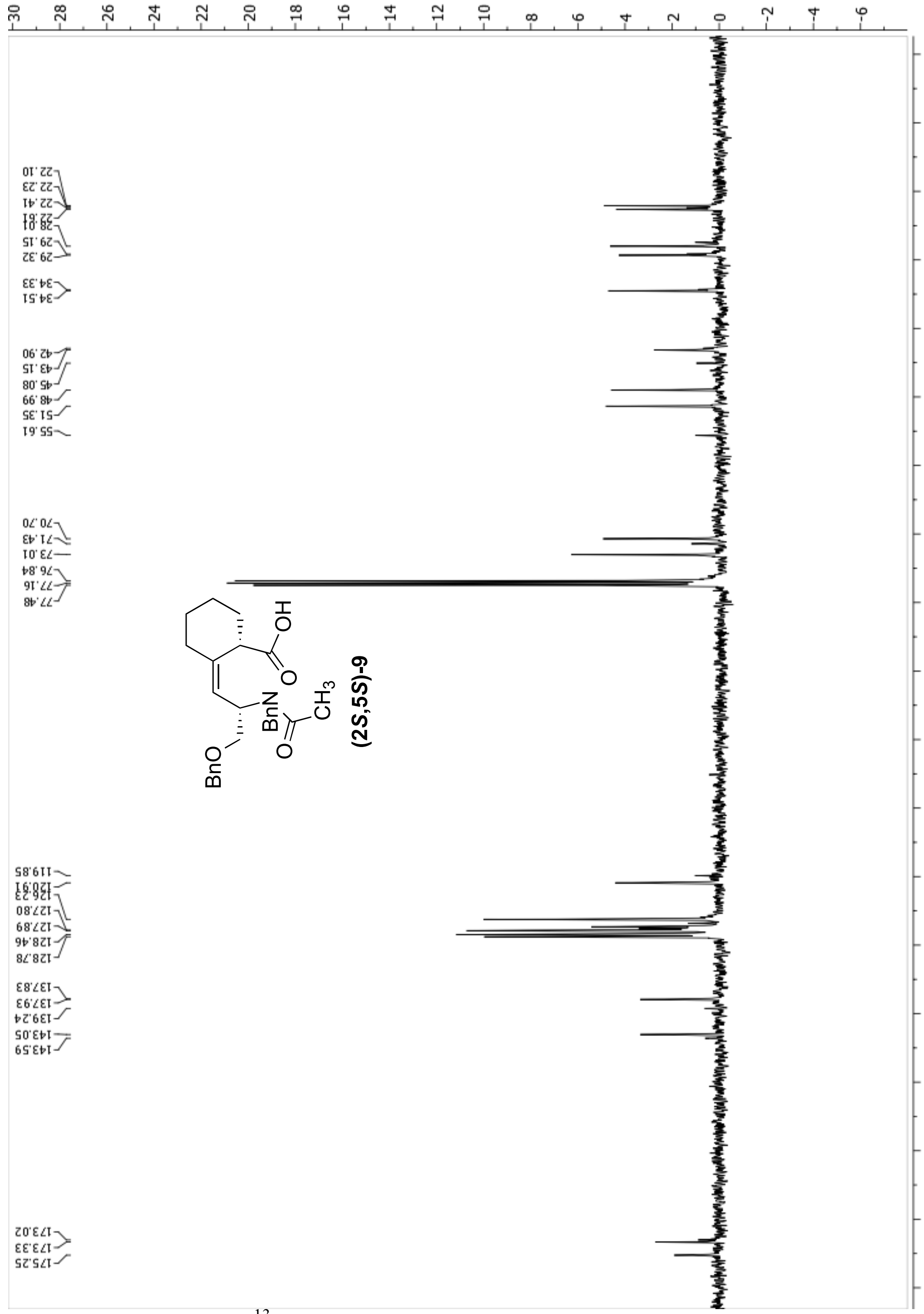

^{13}C NMR of **(2*S*,5*S*)-9** in $CDCl_3$ (100 MHz)

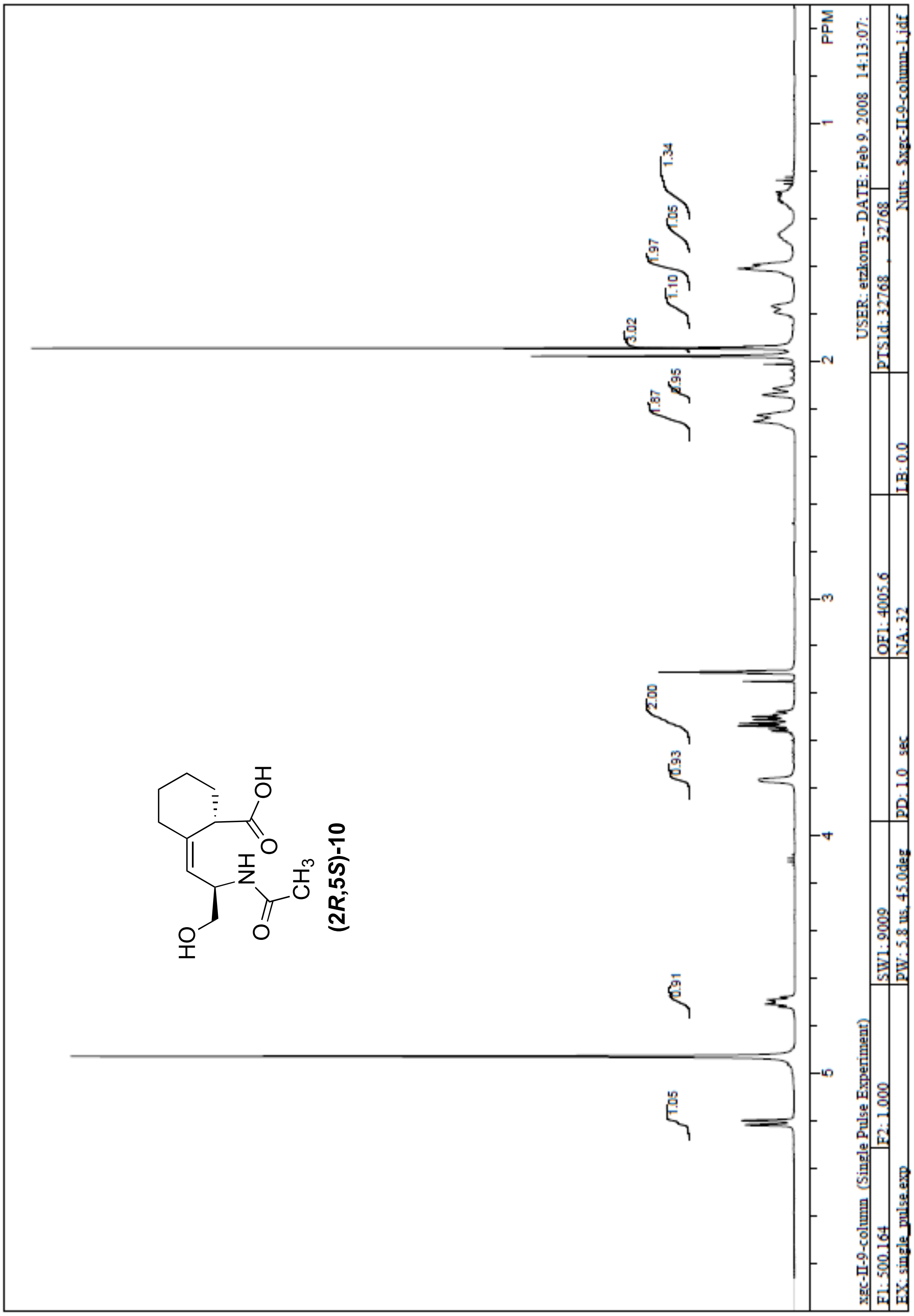

^{1}H NMR of **(2*R*,5*S*)-10** in CD_3OD (500 MHz)

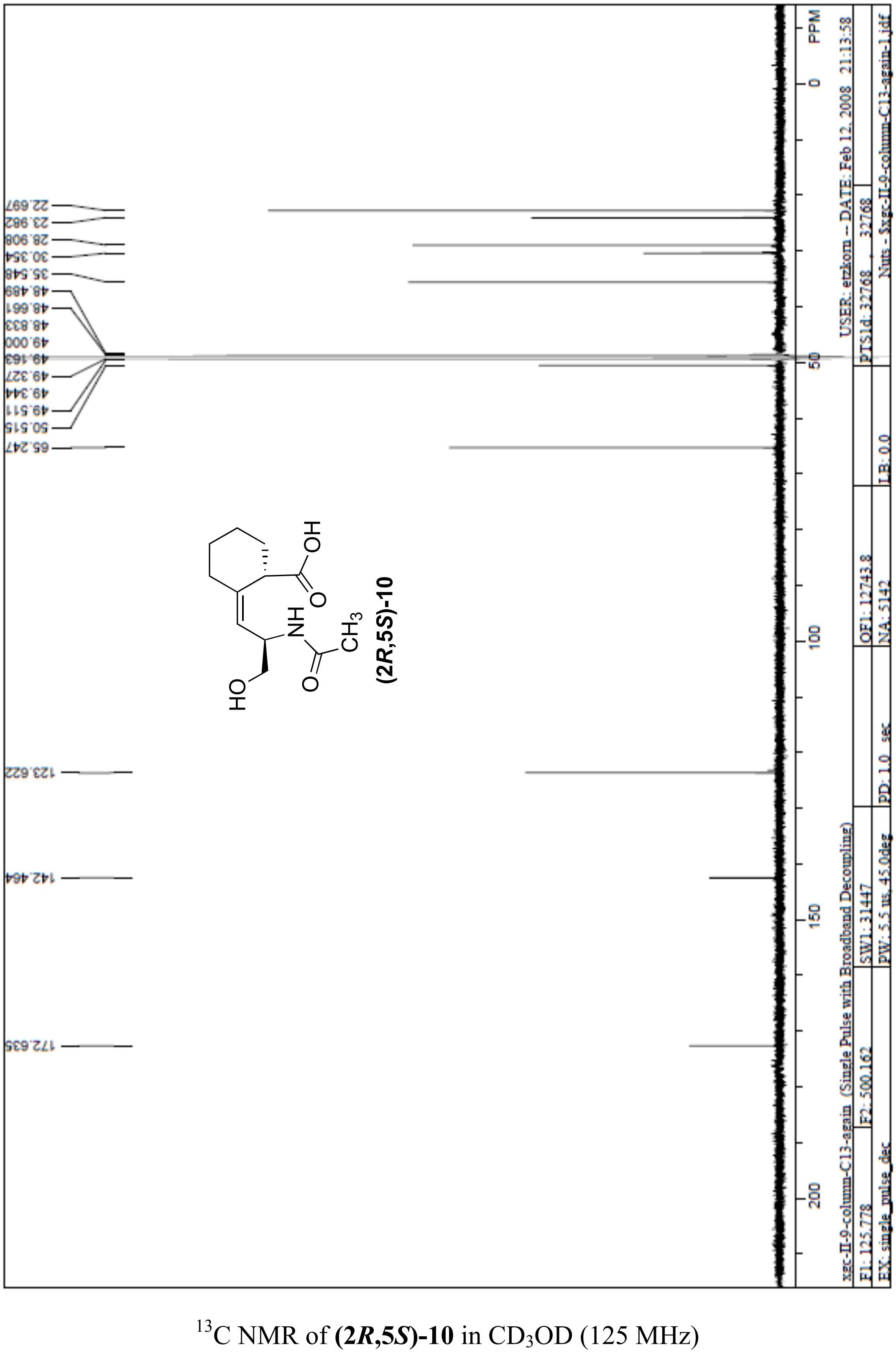

^{13}C NMR of **(2*R*,5*S*)-10** in CD_3OD (125 MHz)

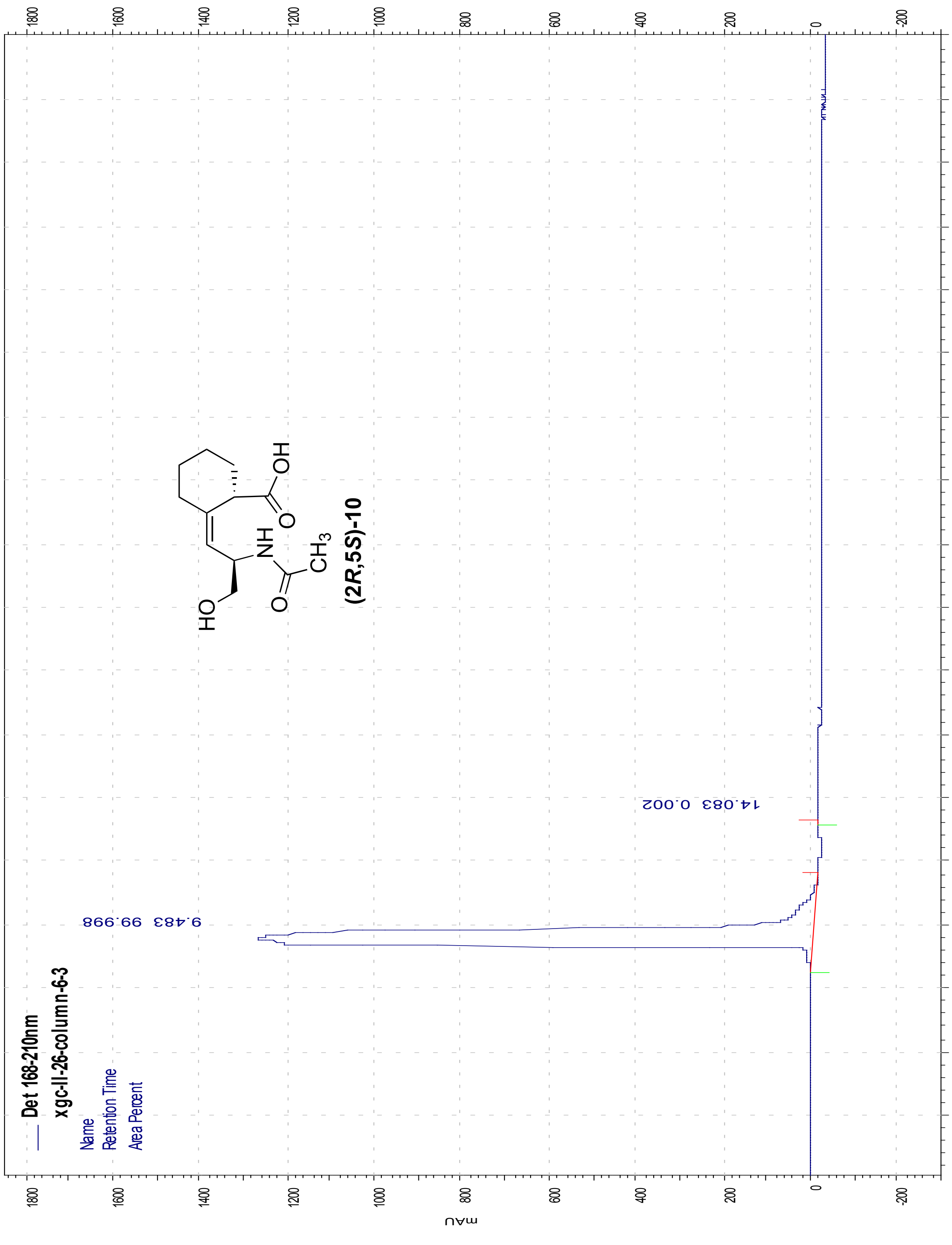

Analytical HPLC of **(2*R*,5*S*)-10**

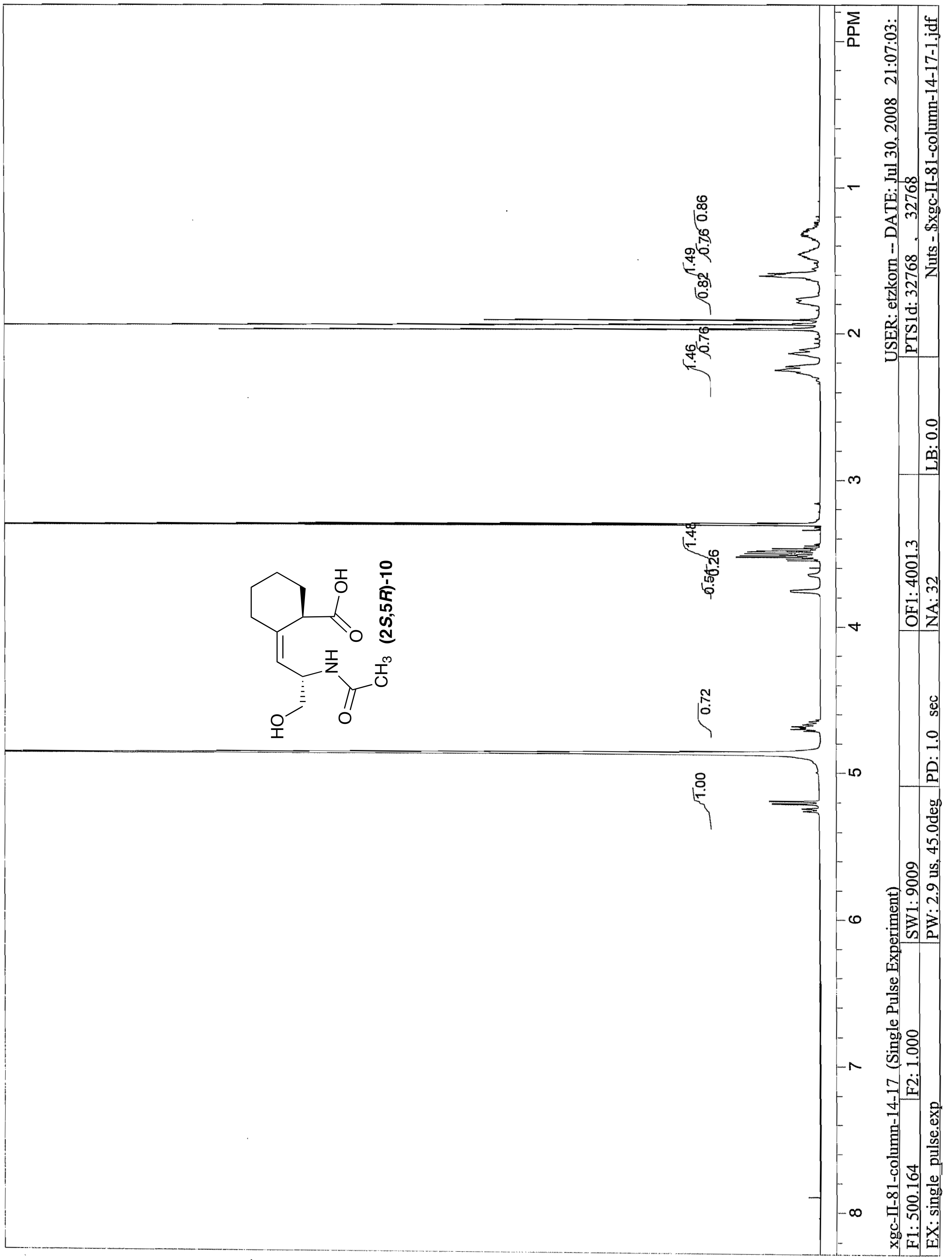

^{1}H NMR of **(2*S*,5*R*)-10** in CD_3OD (500 MHz)

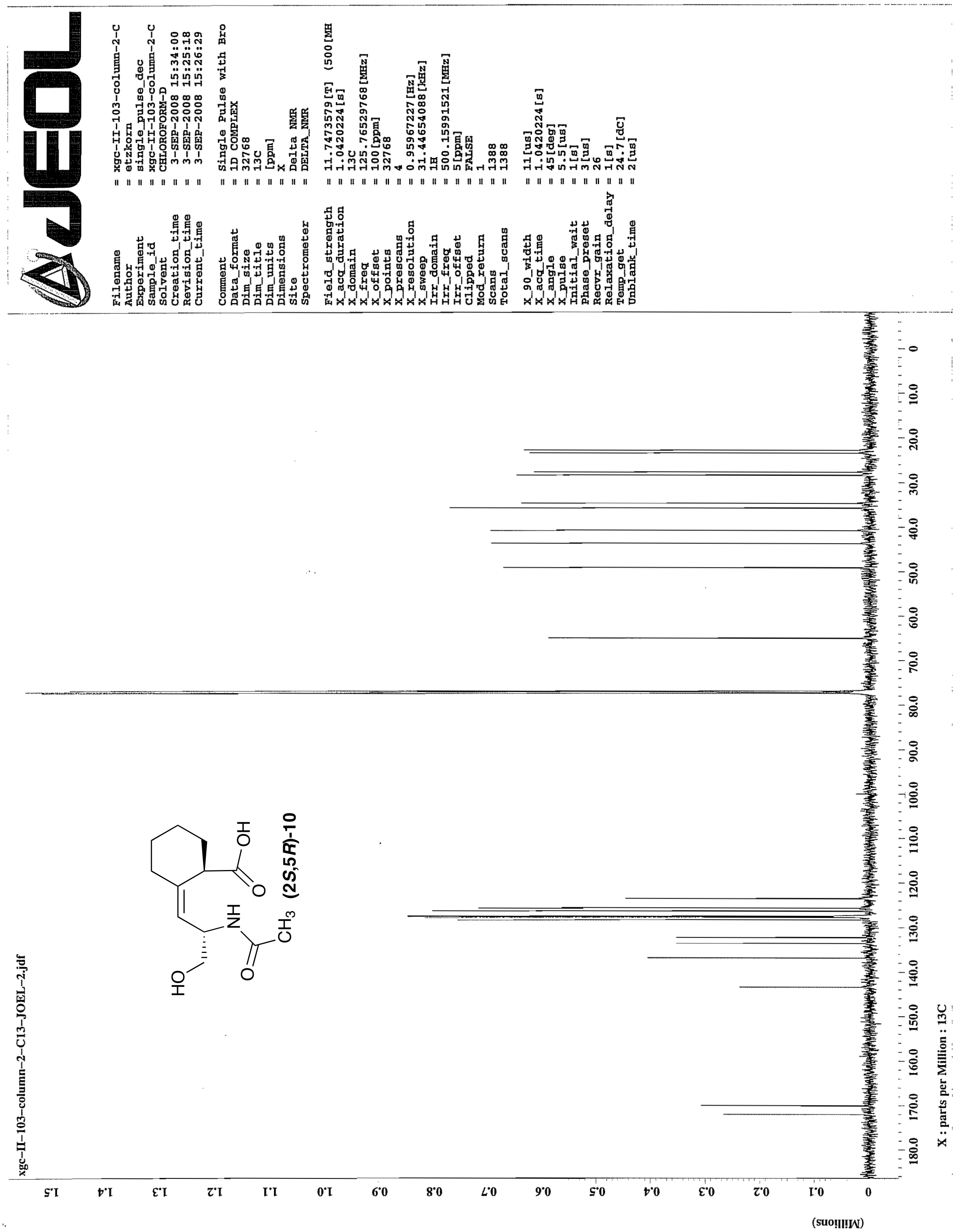

^{13}C NMR of **(2*S*,5*R*)-10** in $CDCl_3$ (125 MHz)

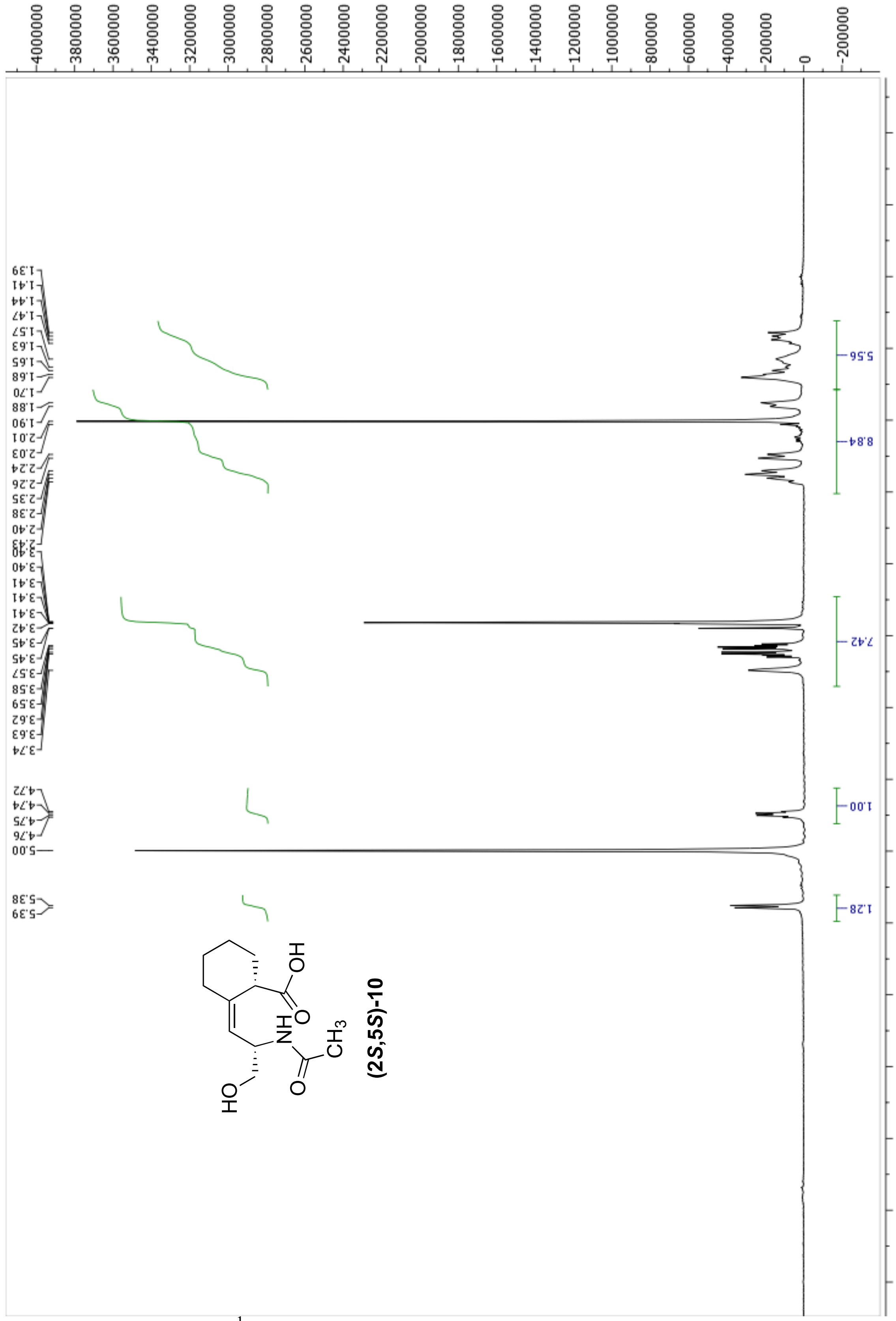

^{1}H NMR of **(2*S*,5*S*)-10** in CD_3OD (500 MHz)

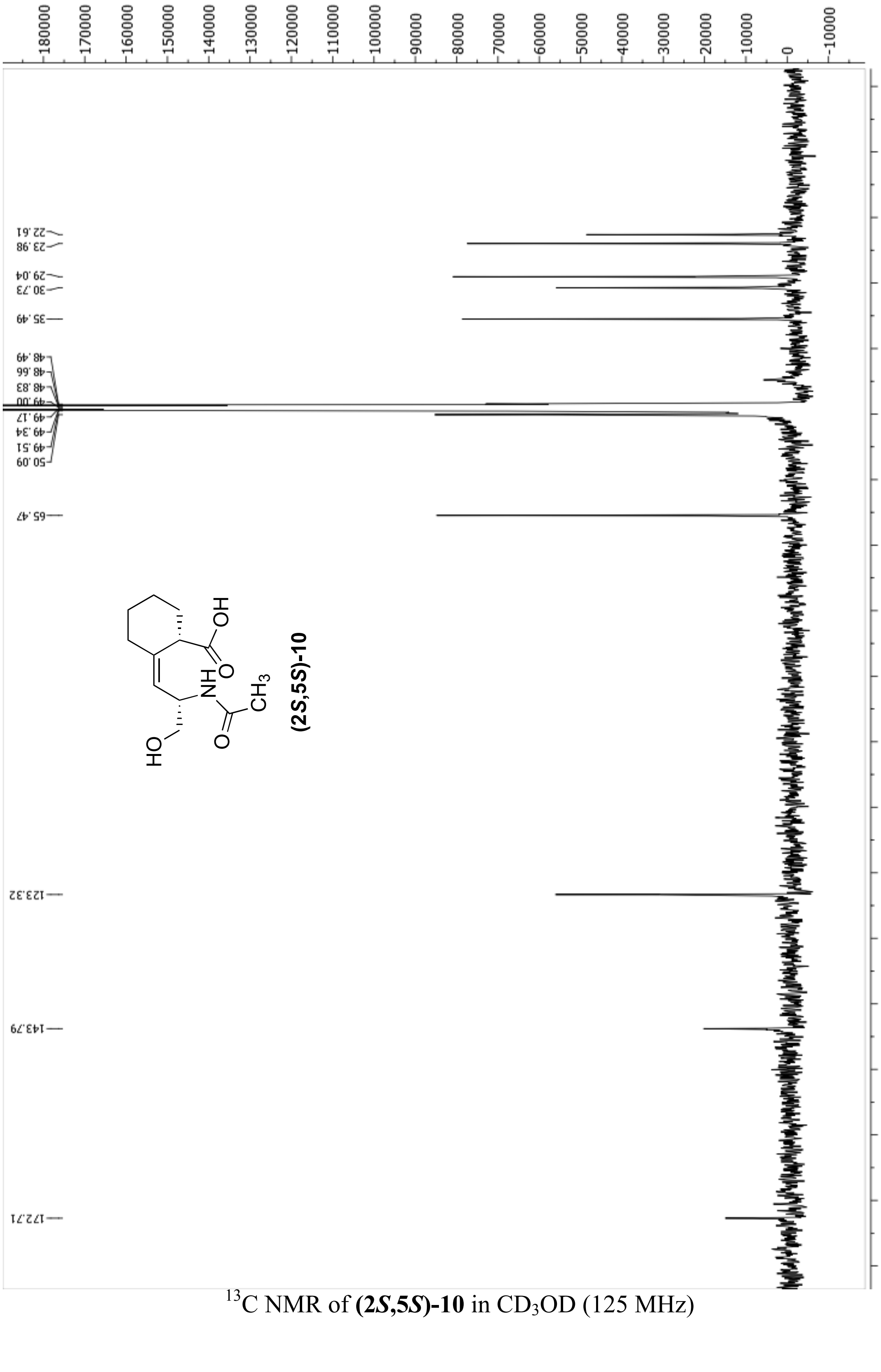

^{13}C NMR of **(2*S*,5*S*)-10** in CD_3OD (125 MHz)

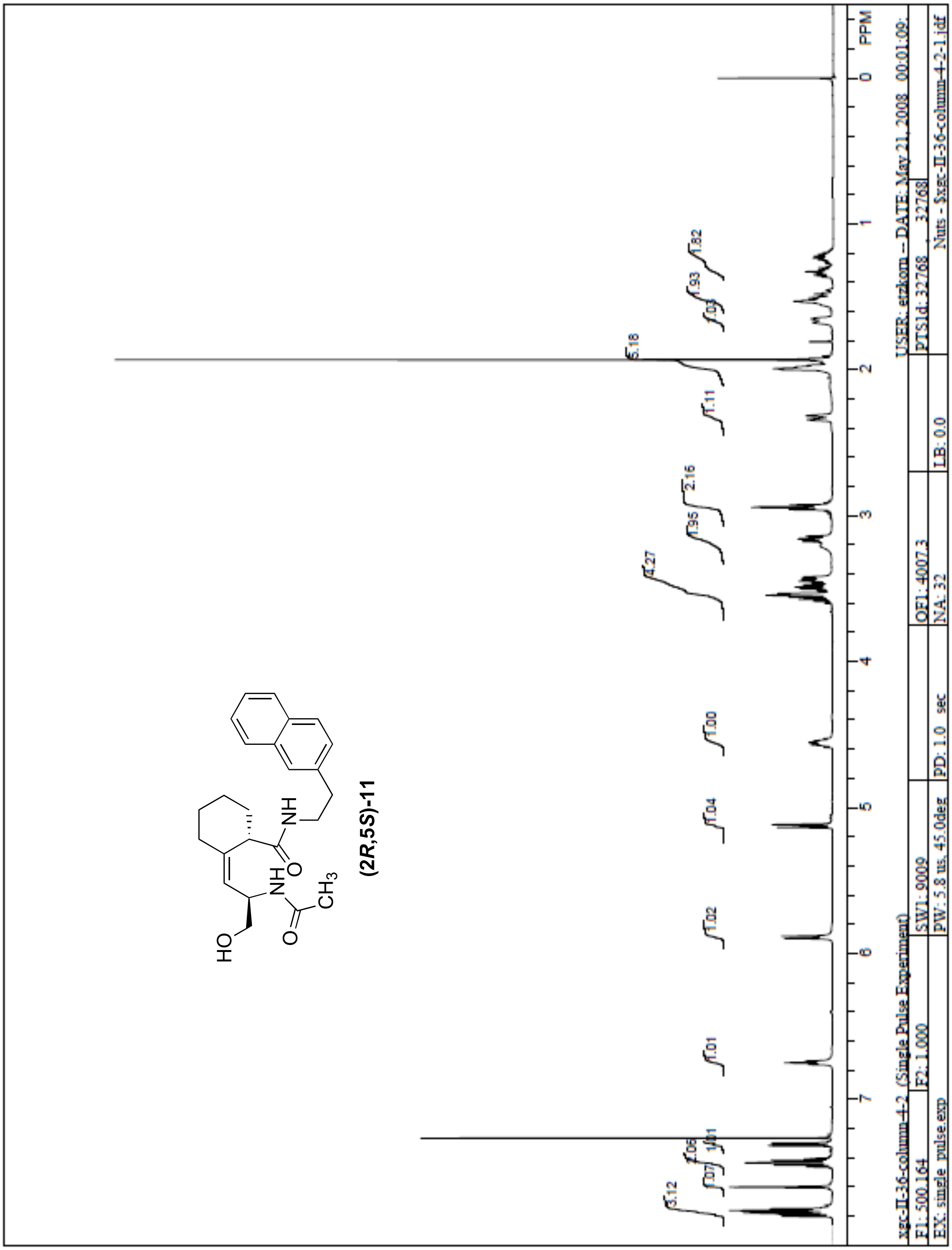

^{1}H NMR of **(2*R*,5*S*)-11** in $CDCl_3$ (500 MHz)

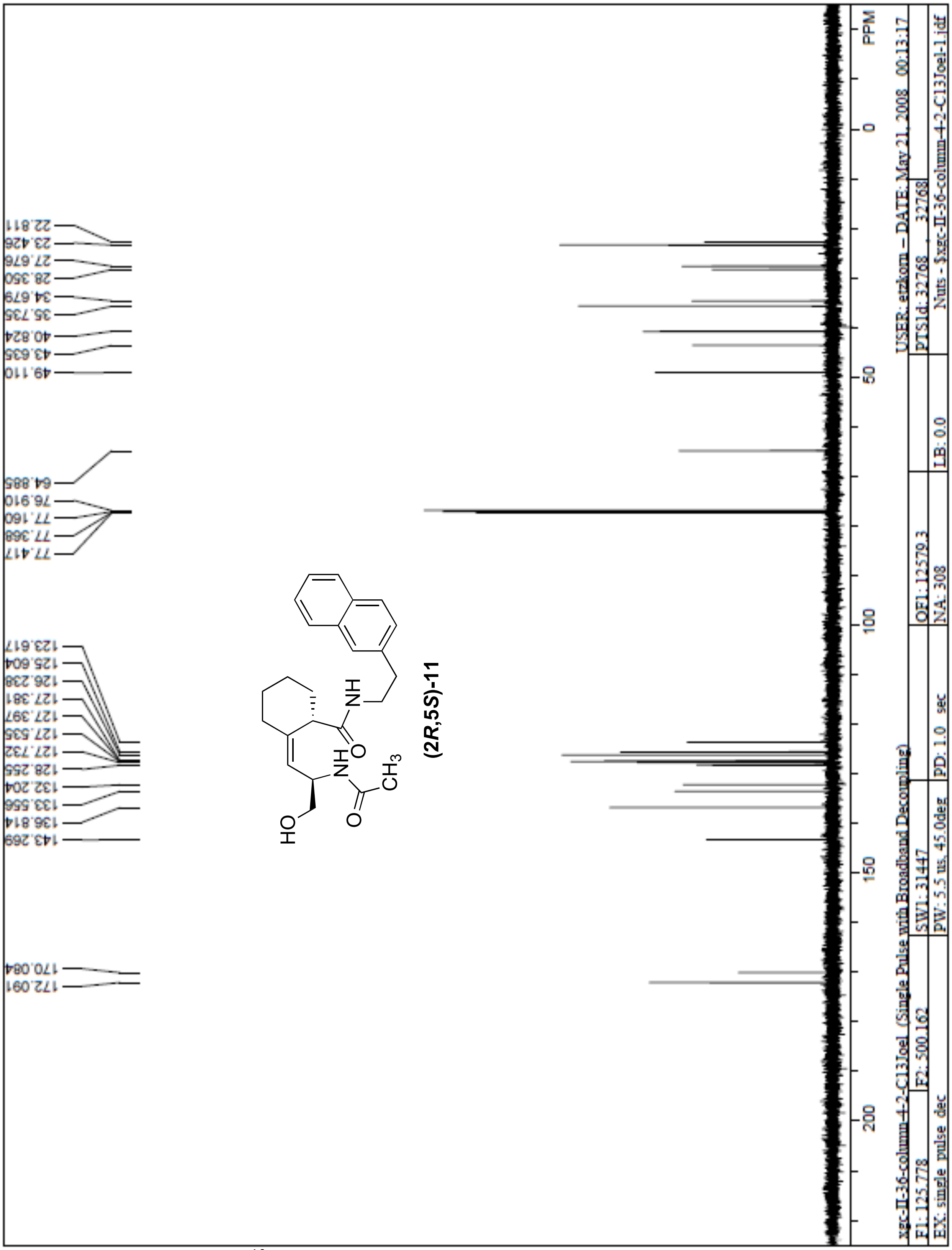

^{13}C NMR of **(2*R*,5*S*)-11** in $CDCl_3$ (125 MHz)

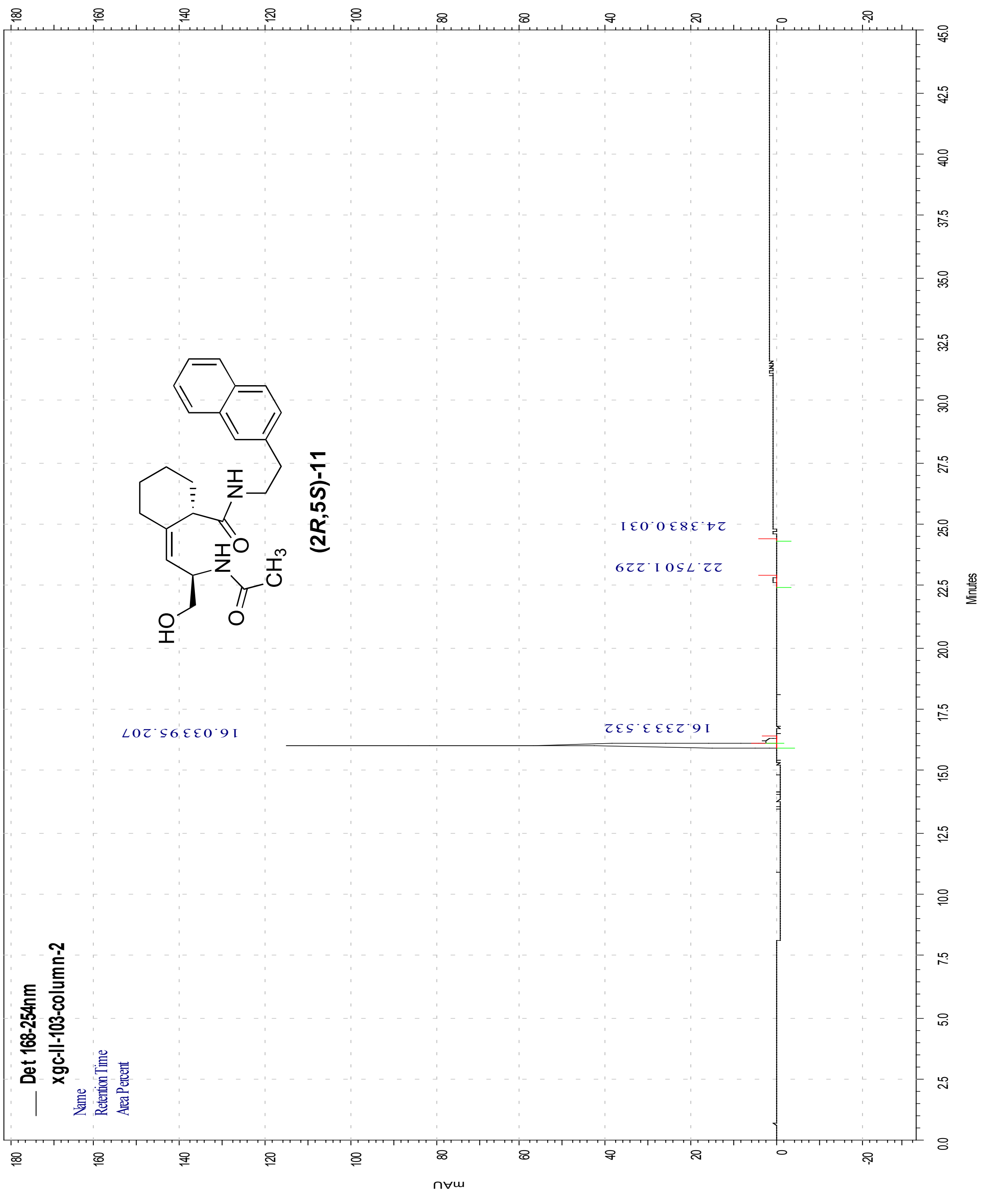

Analytical HPLC of **(2*S*,5*R*)-11**

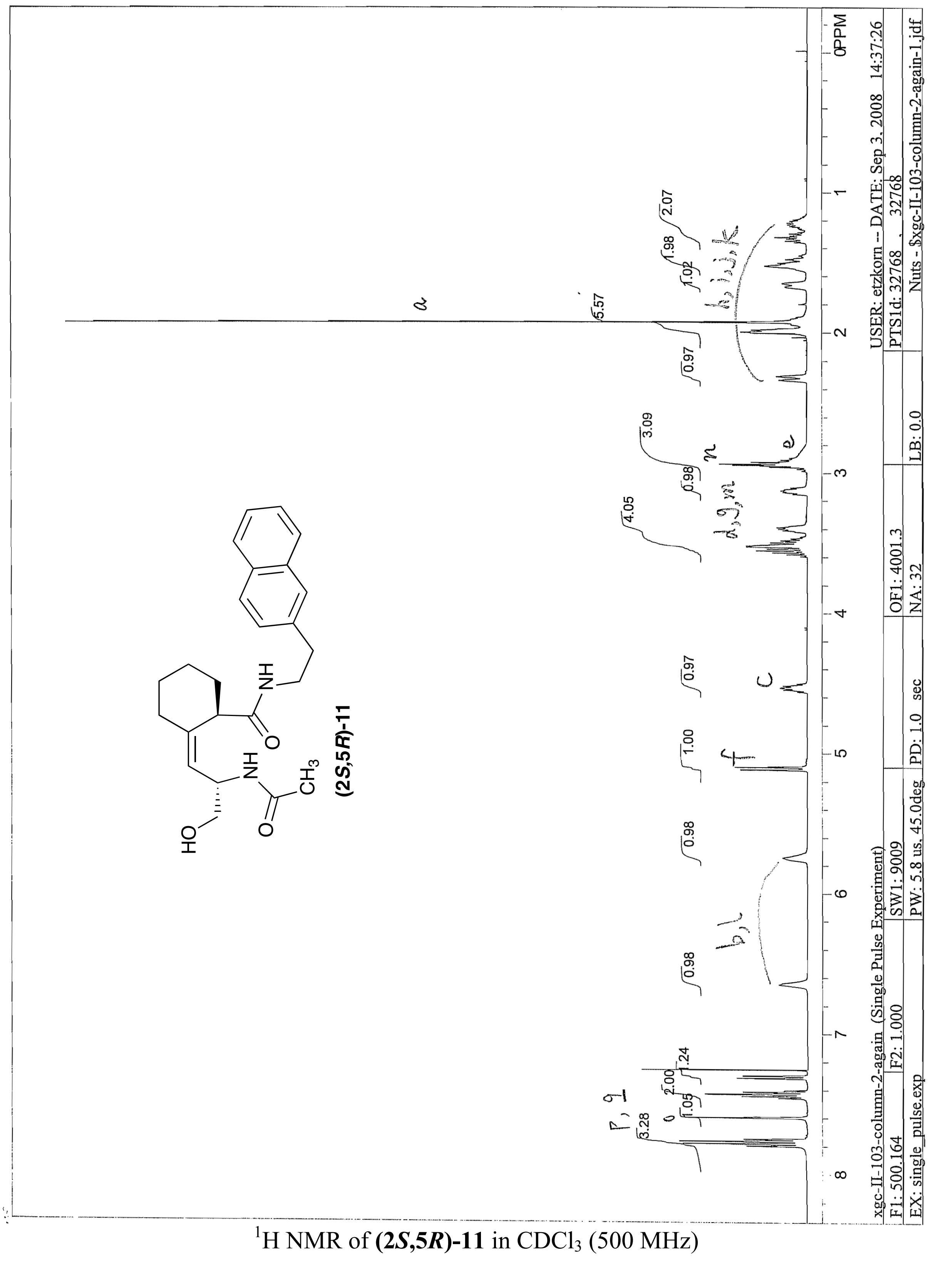

^{1}H NMR of **(2*S*,5*R*)-11** in $CDCl_3$ (500 MHz)

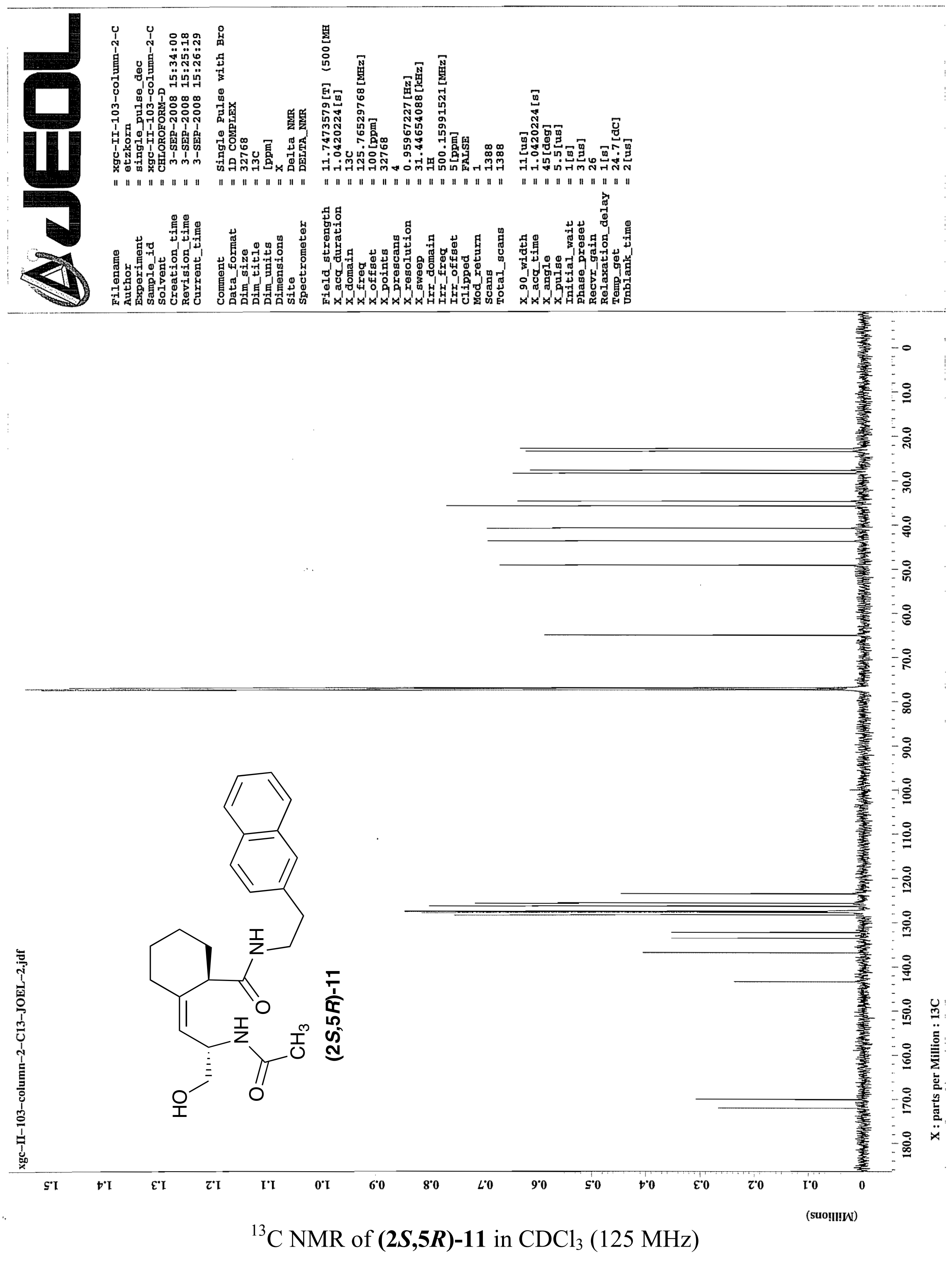

^{13}C NMR of **(2*S*,5*R*)-11** in $CDCl_3$ (125 MHz)

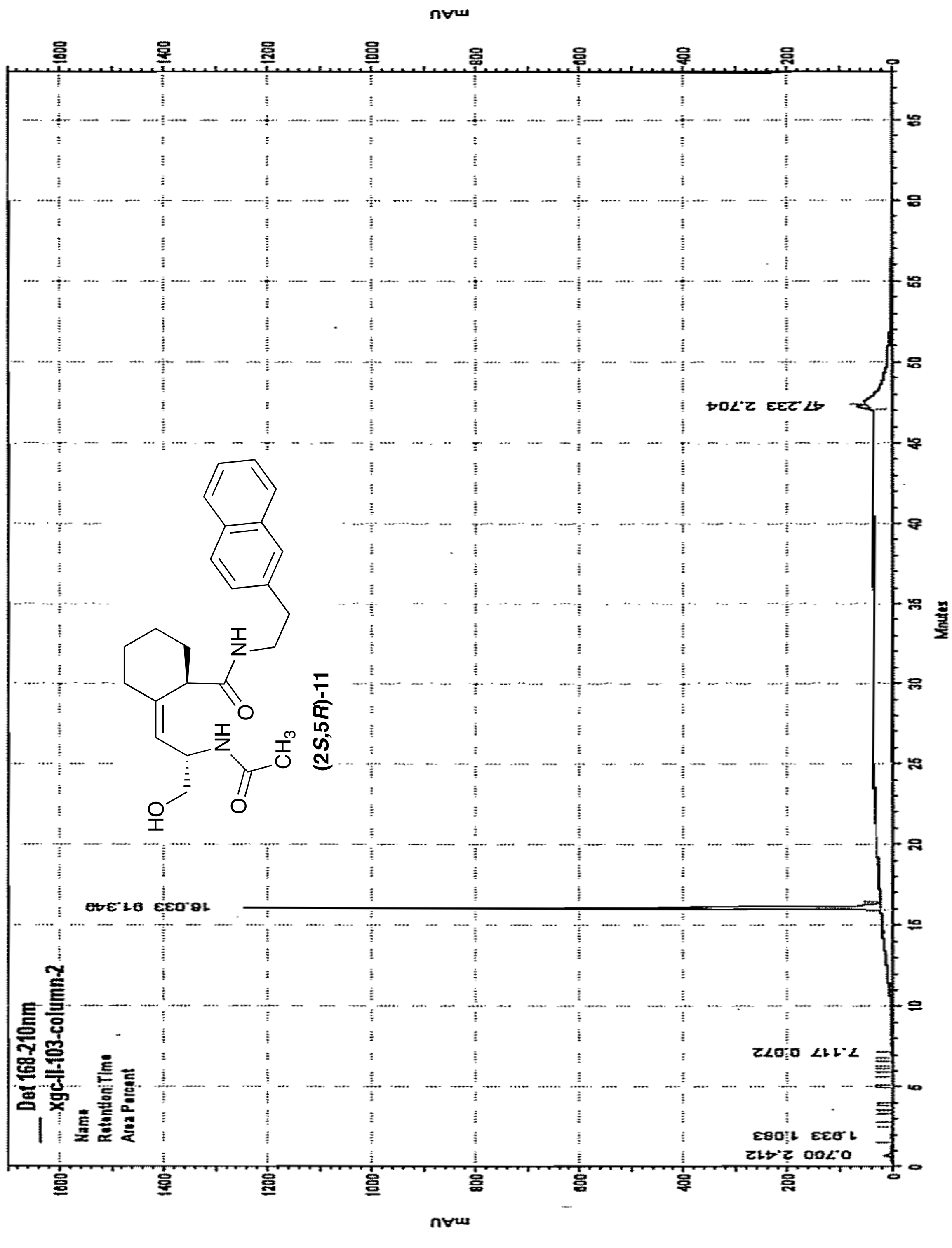

Analytical HPLC of **(2*S*,5*R*)-11**

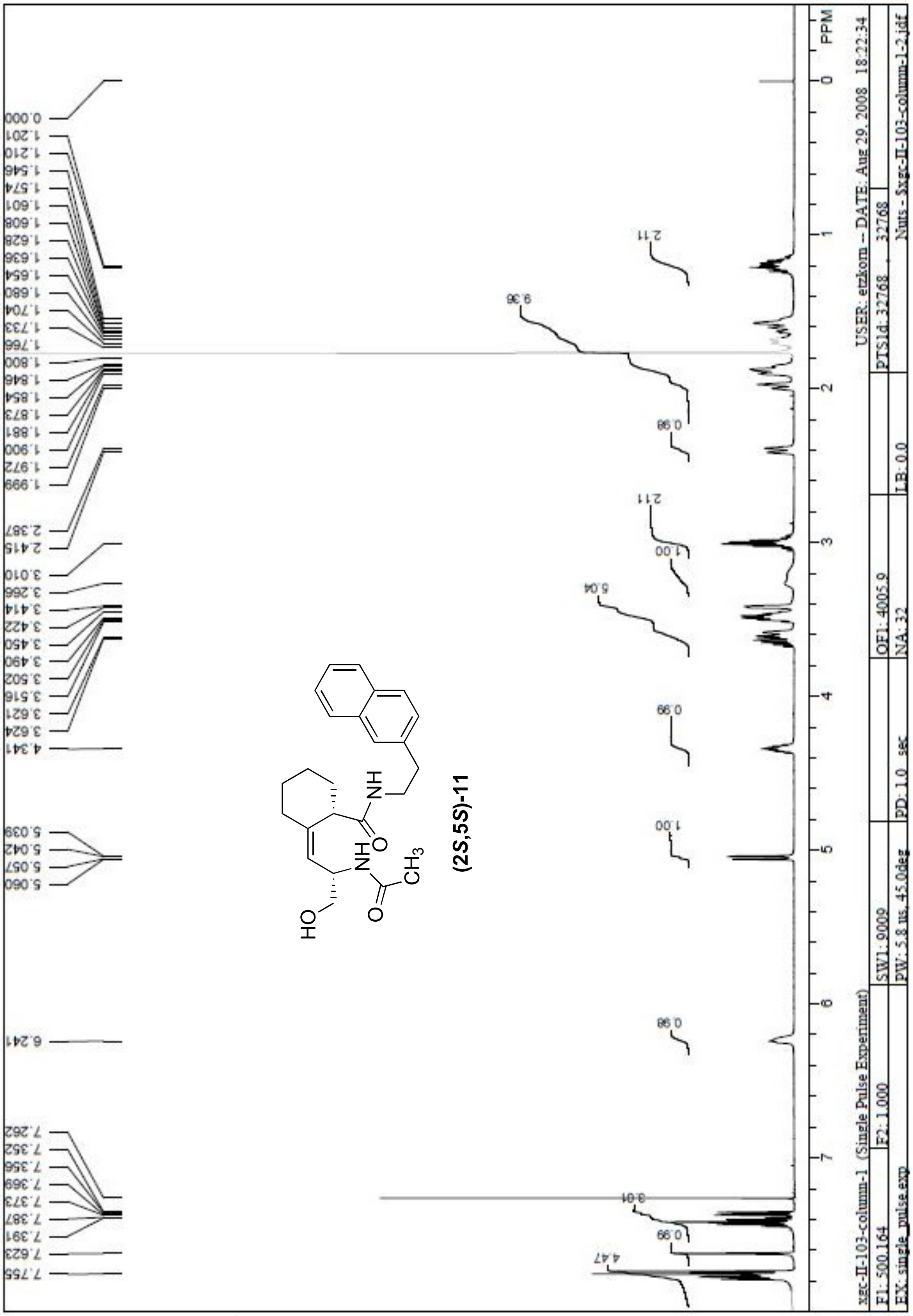

^{1}H NMR of **(2*S*,5*S*)-11** in $CDCl_3$ (500 MHz)

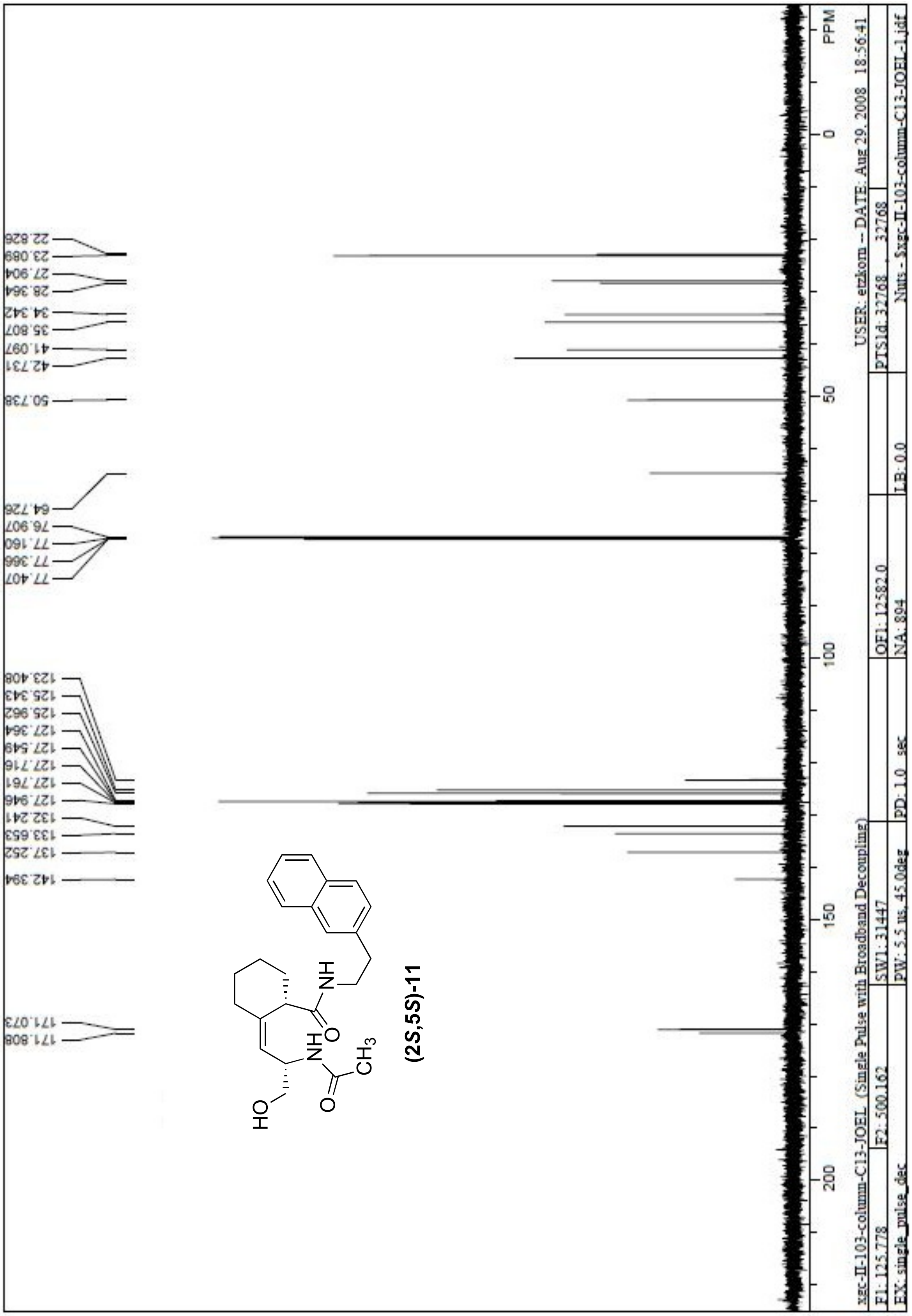

^{13}C NMR of **(2*S*,5*S*)-11** in $CDCl_3$ (125 MHz)

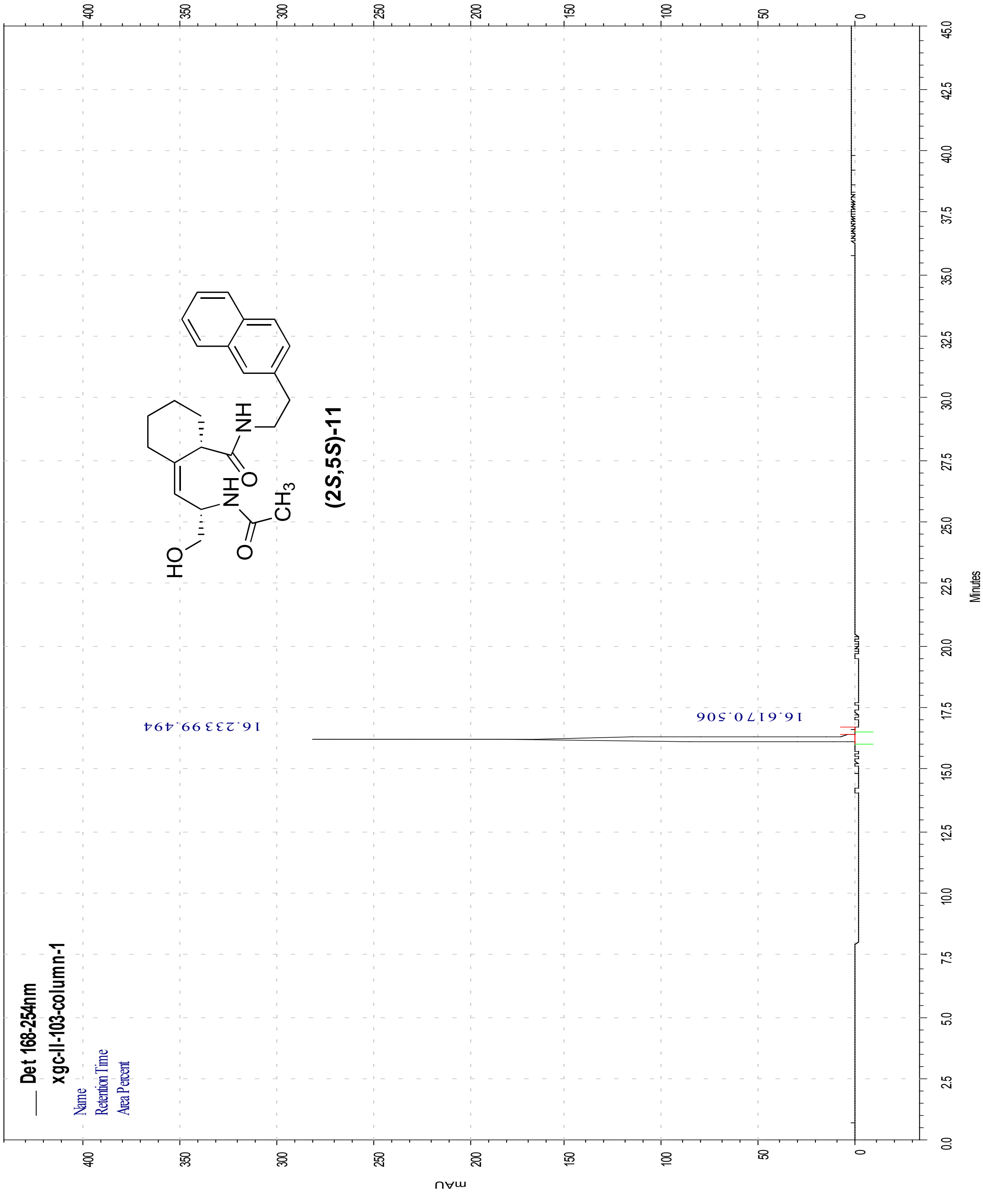

Analytical HPLC of **(2*S*,5*S*)-11**

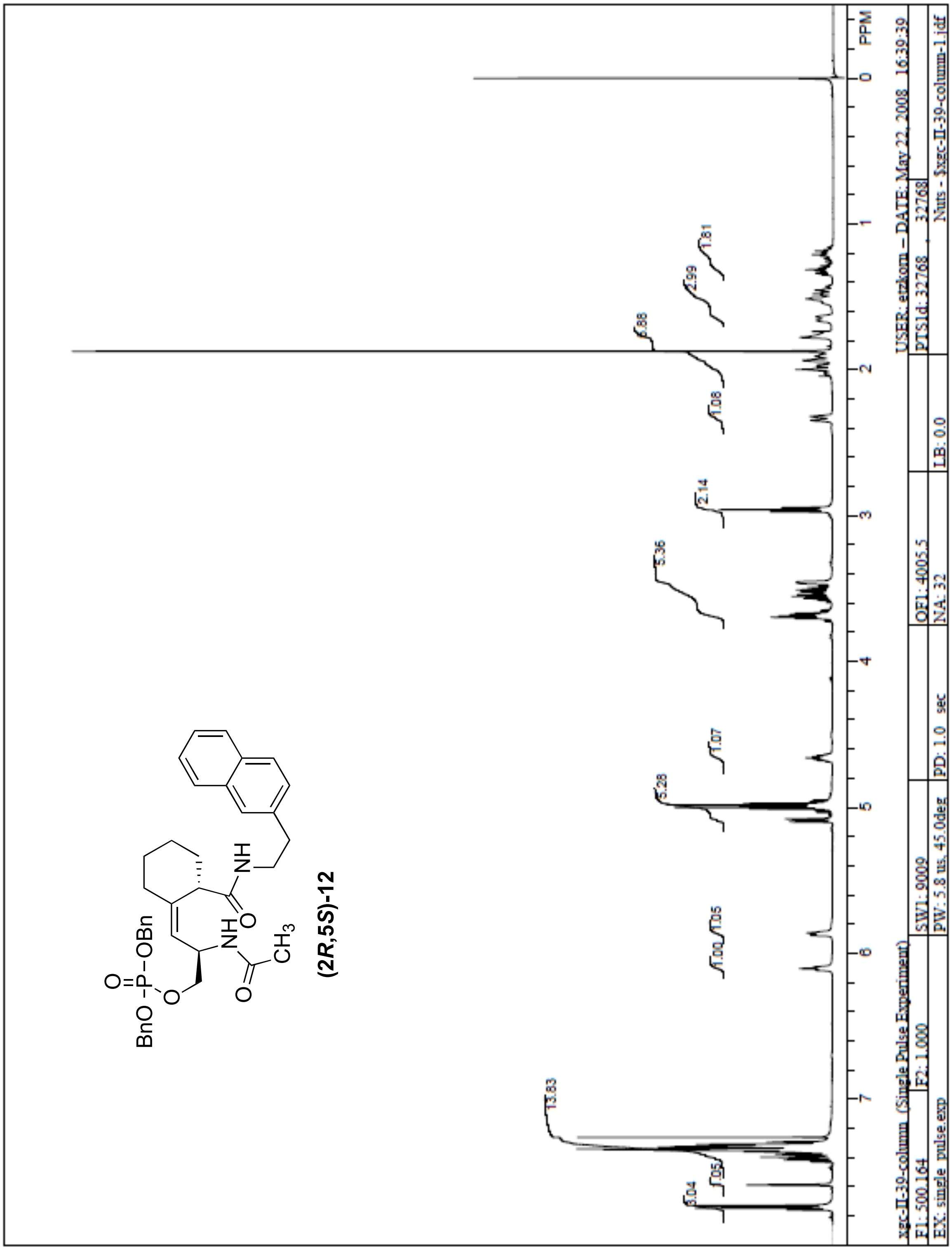

^{1}H NMR of **(2*R*,5*S*)-12** in $CDCl_3$ (500 MHz)

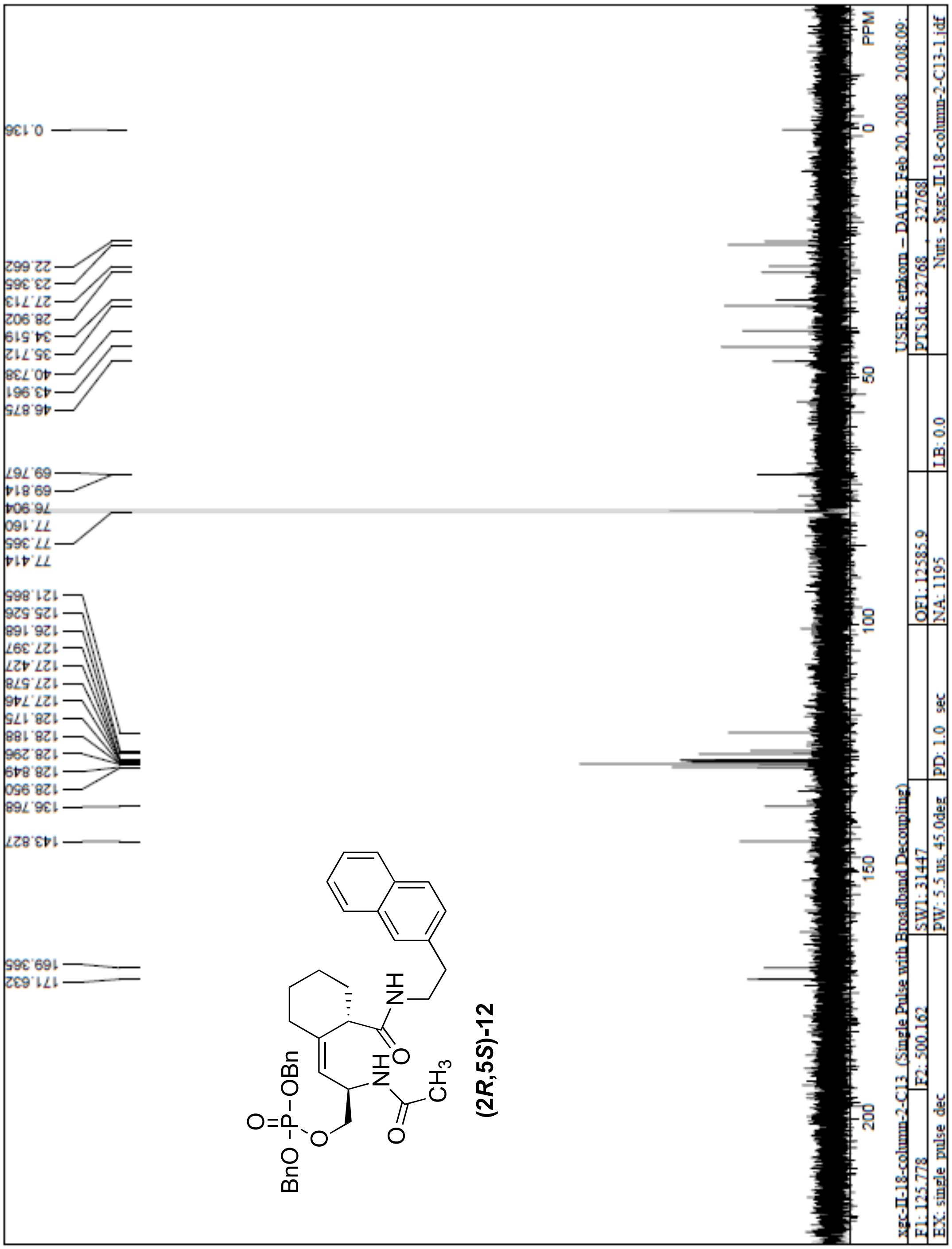

^{13}C NMR of **(2*R*,5*S*)-12** in $CDCl_3$ (125 MHz)

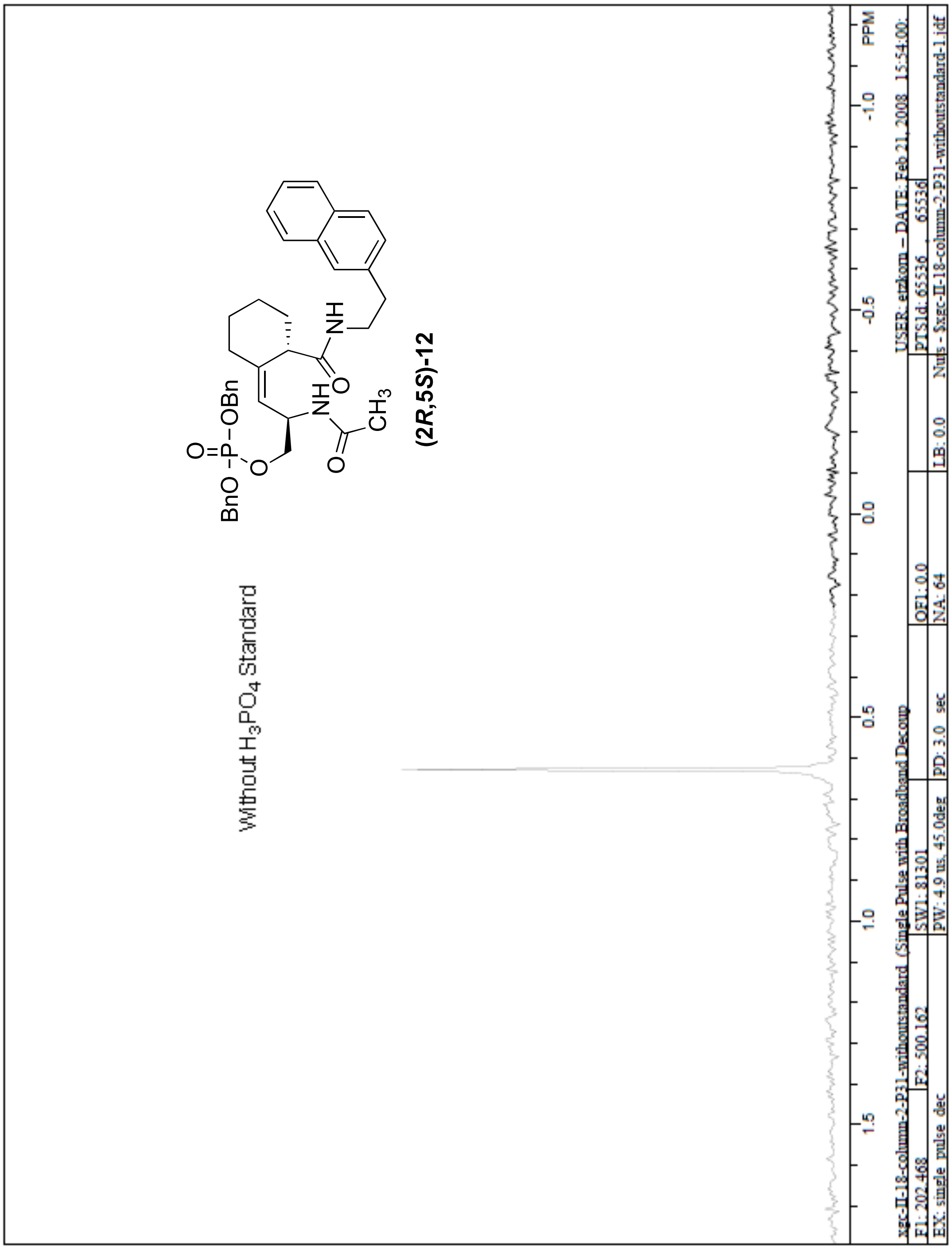

^{31}P NMR of **(2*R*,5*S*)-12** in $CDCl_3$ (202 MHz)

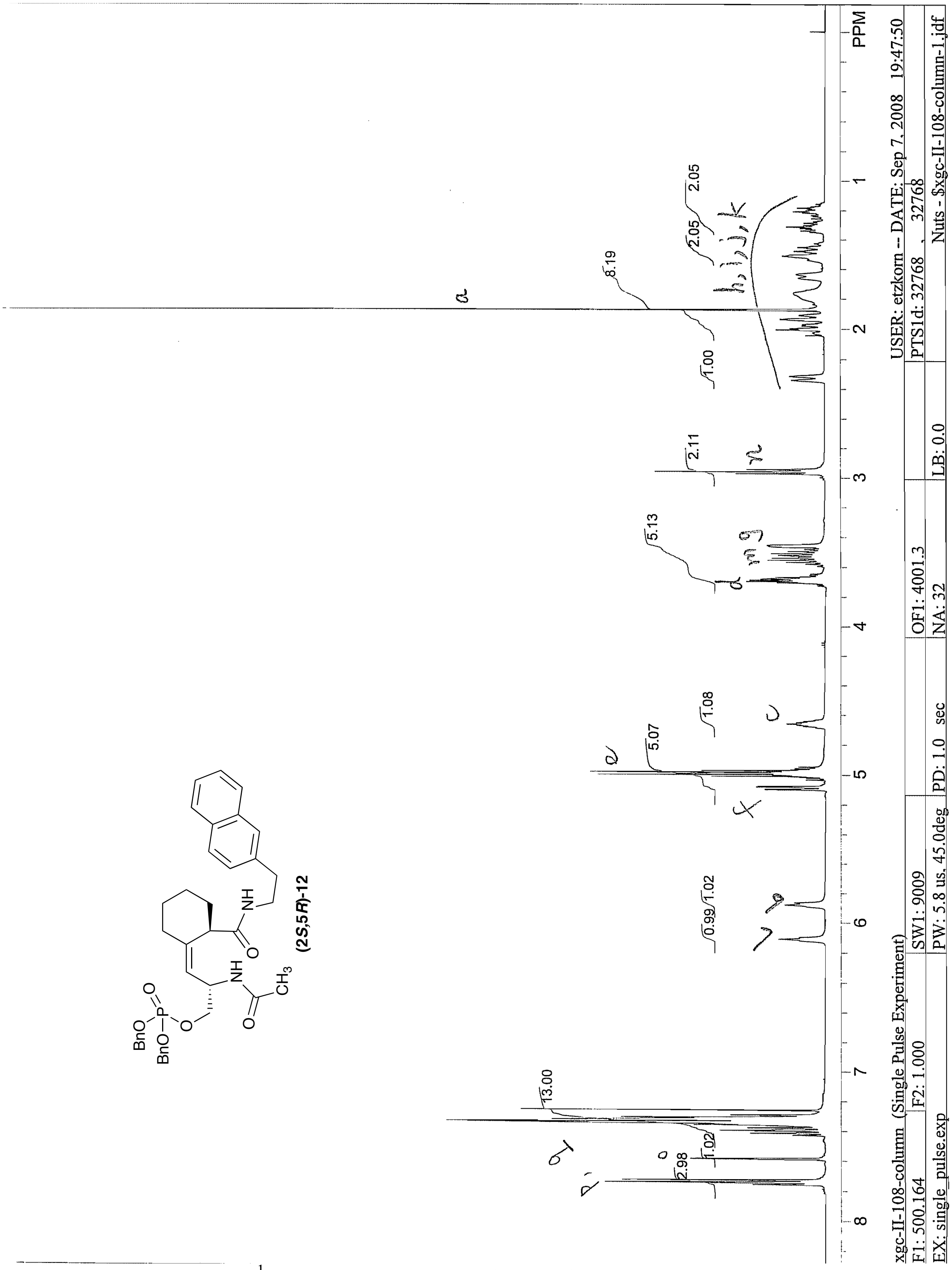

^{1}H NMR of **(2*S*,5*R*)-12** in $CDCl_3$ (500 MHz)

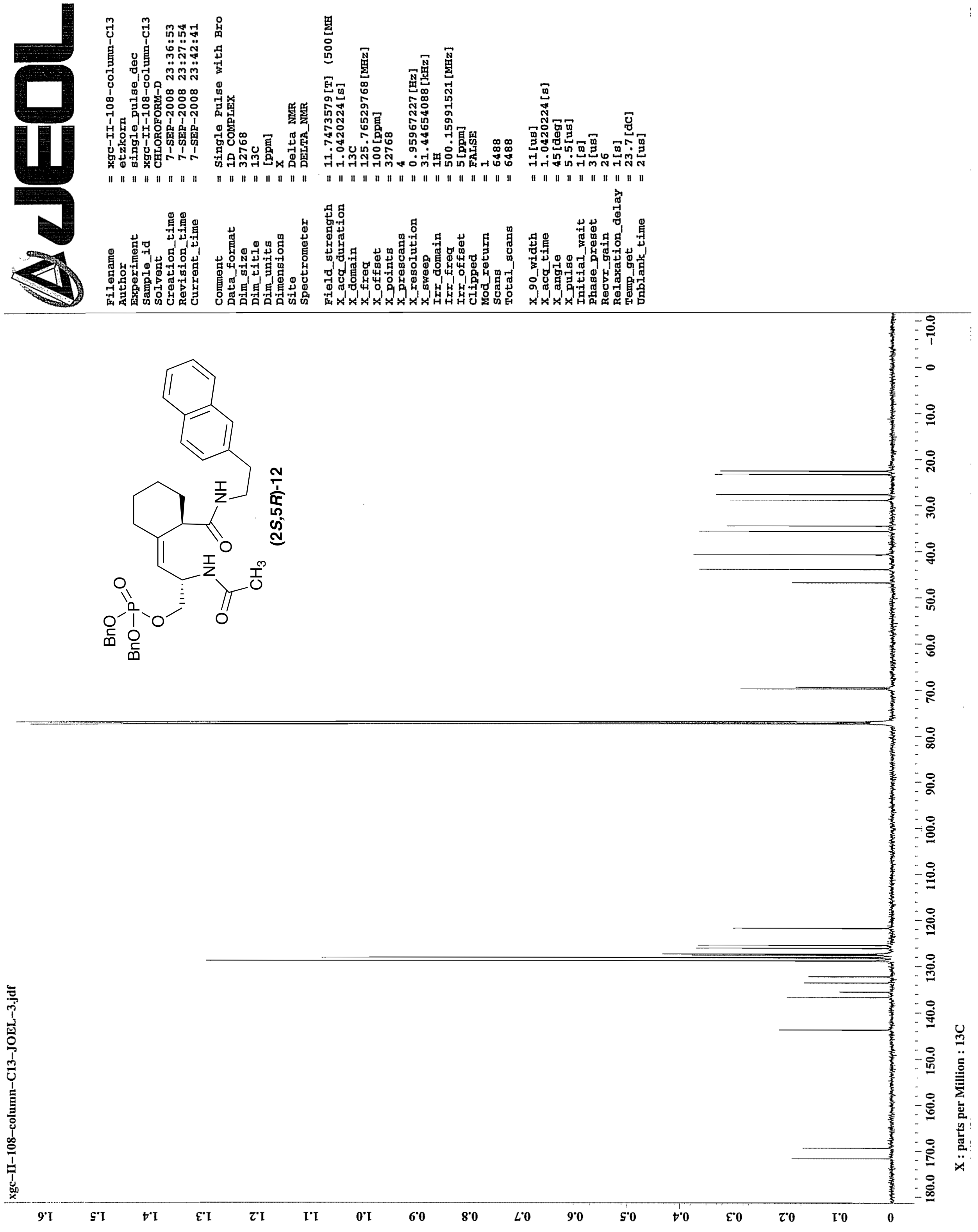

^{13}C NMR of **(2*S*,5*R*)-12** in $CDCl_3$ (125 MHz)

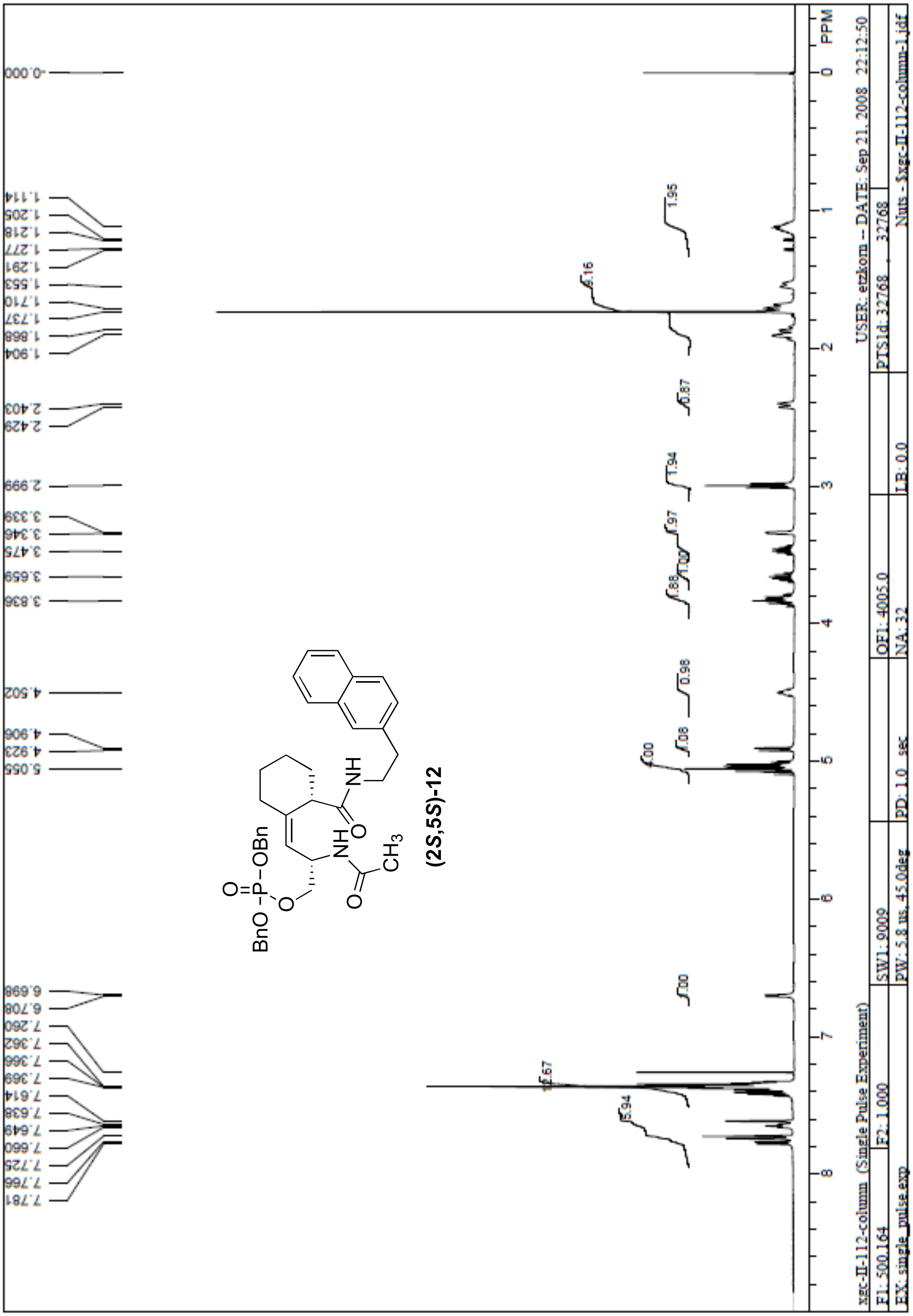

^{1}H NMR of **(2*S*,5*S*)-12** in $CDCl_3$ (500 MHz)

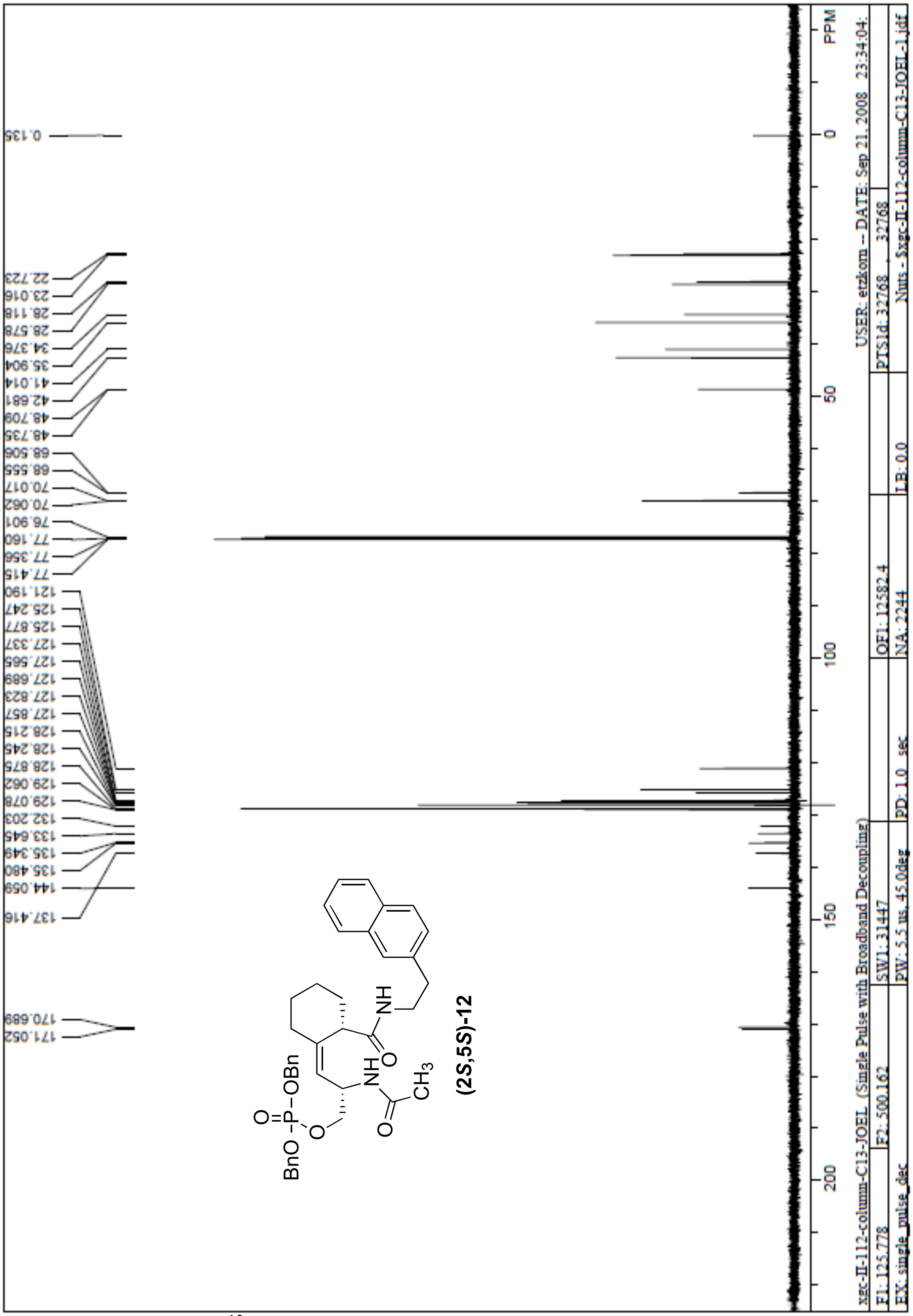

^{13}C NMR of **(2*S*,5*S*)-12** in $CDCl_3$ (125 MHz)

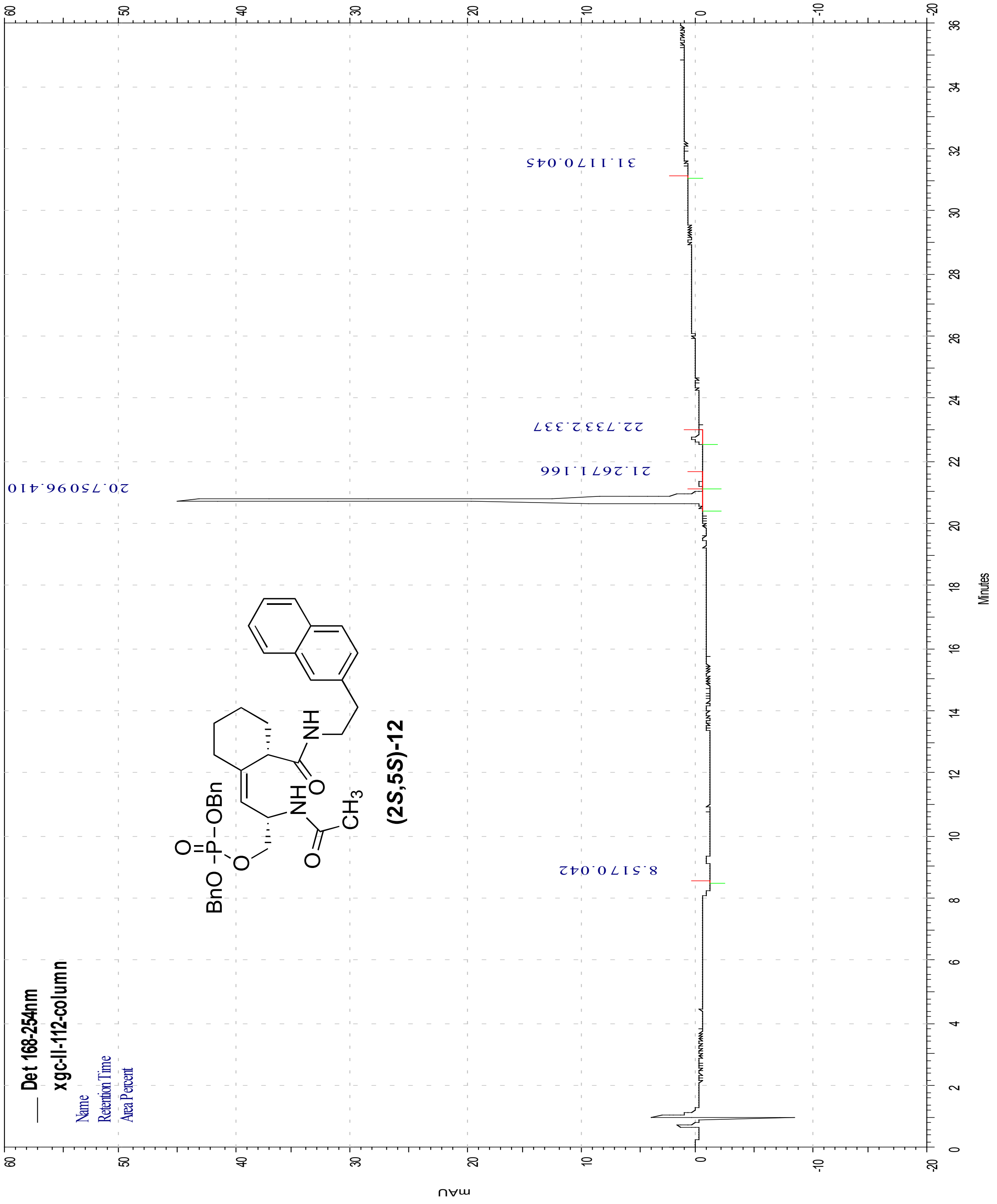

Analytical HPLC of **(2*S*,5*S*)-12**

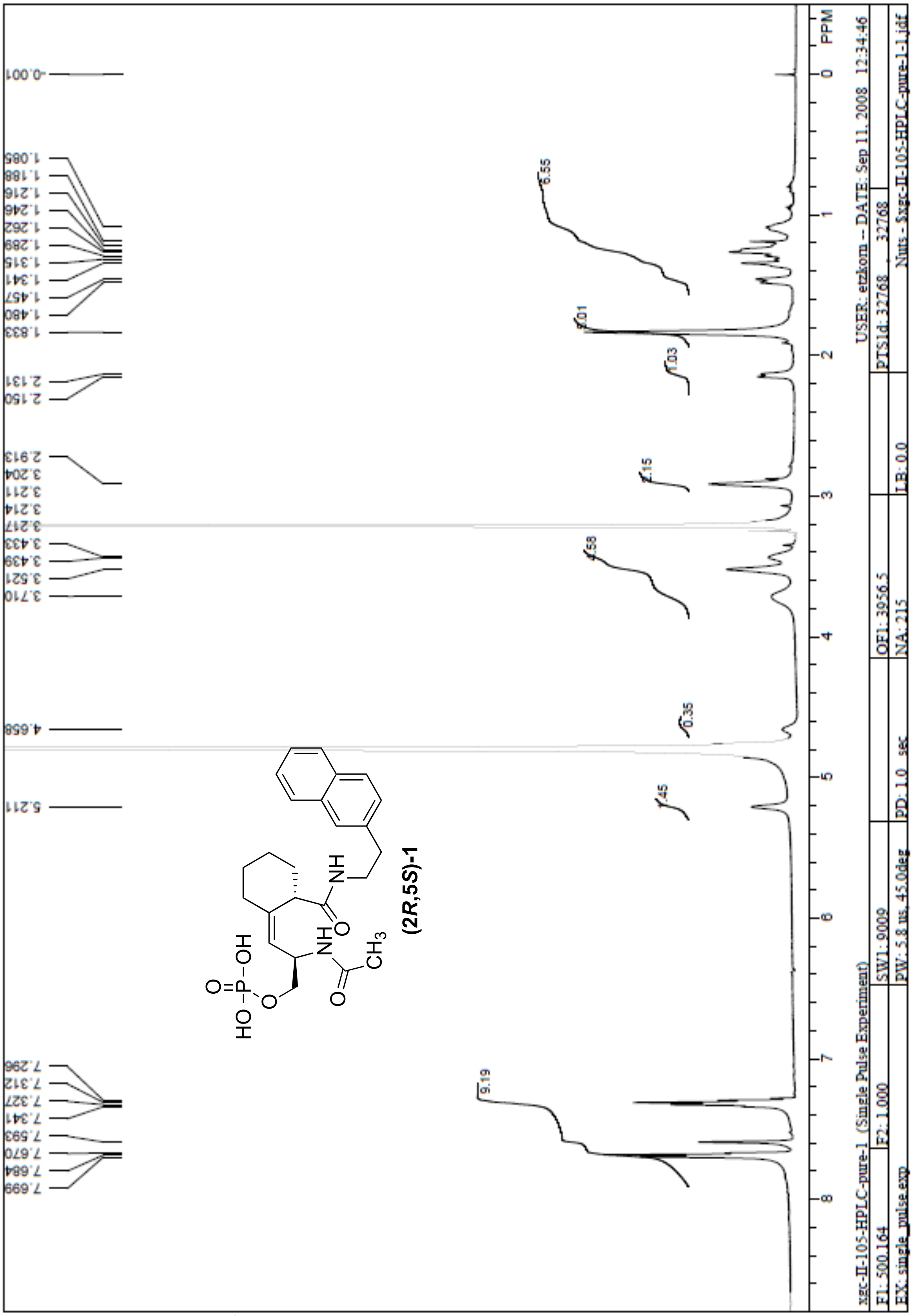

^{1}H NMR of **(2*R*,5*S*)-1** in CD_3OD (500 MHz)

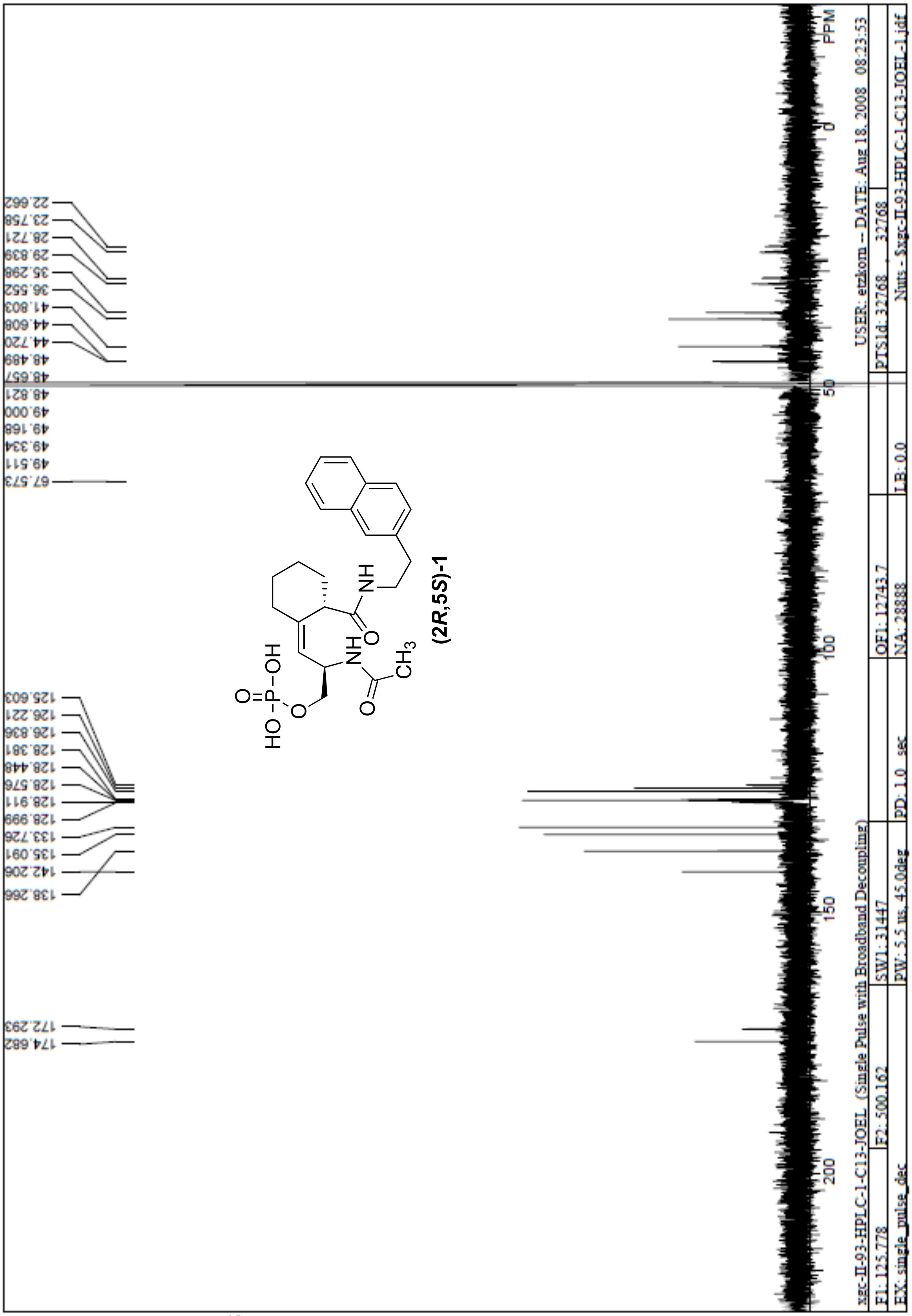

^{13}C NMR of **(2*R*,5*S*)-1** in CD_3OD (125 MHz)

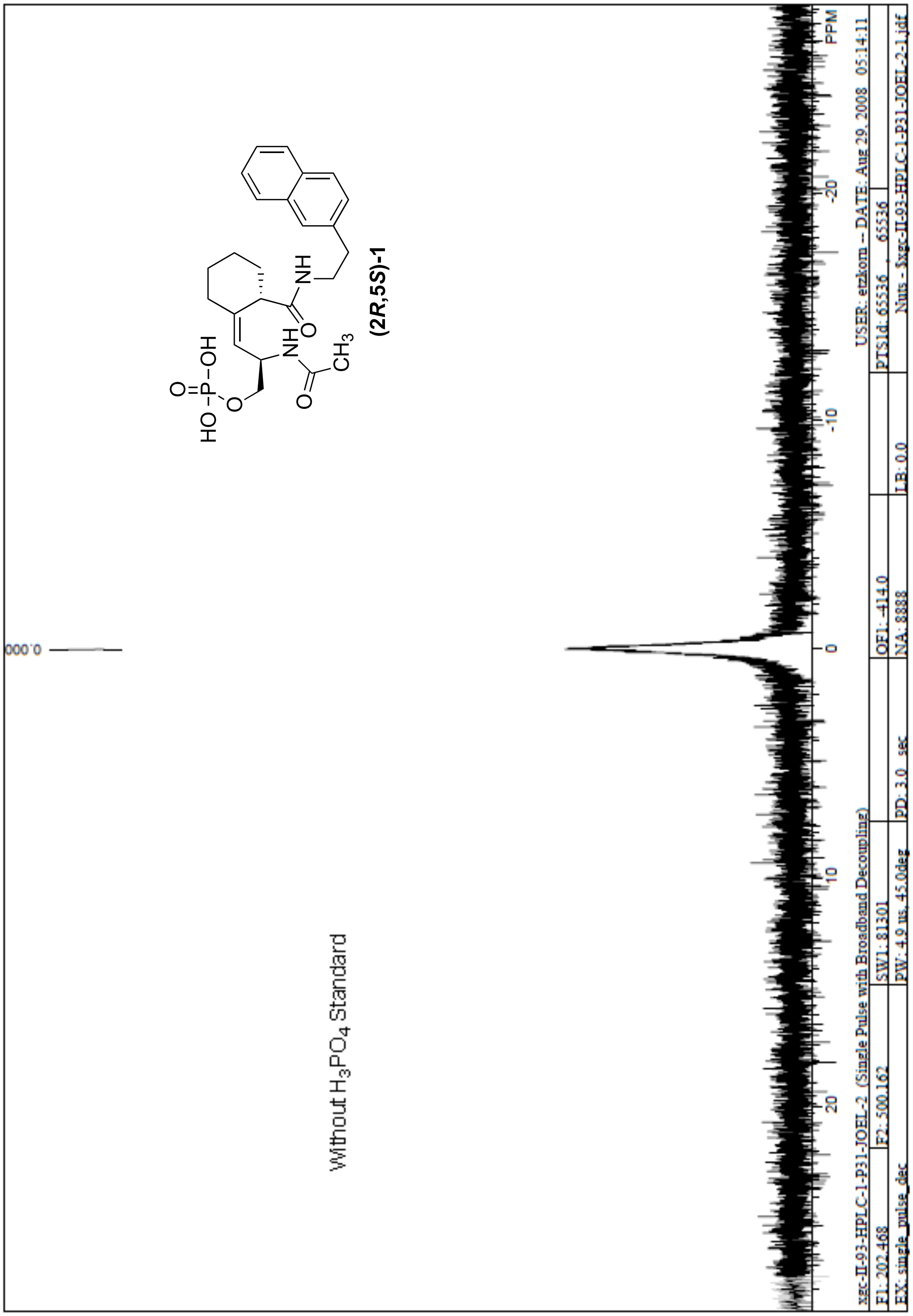

^{31}P NMR of **(2*R*,5*S*)-1** in CD_3OD (202 MHz)

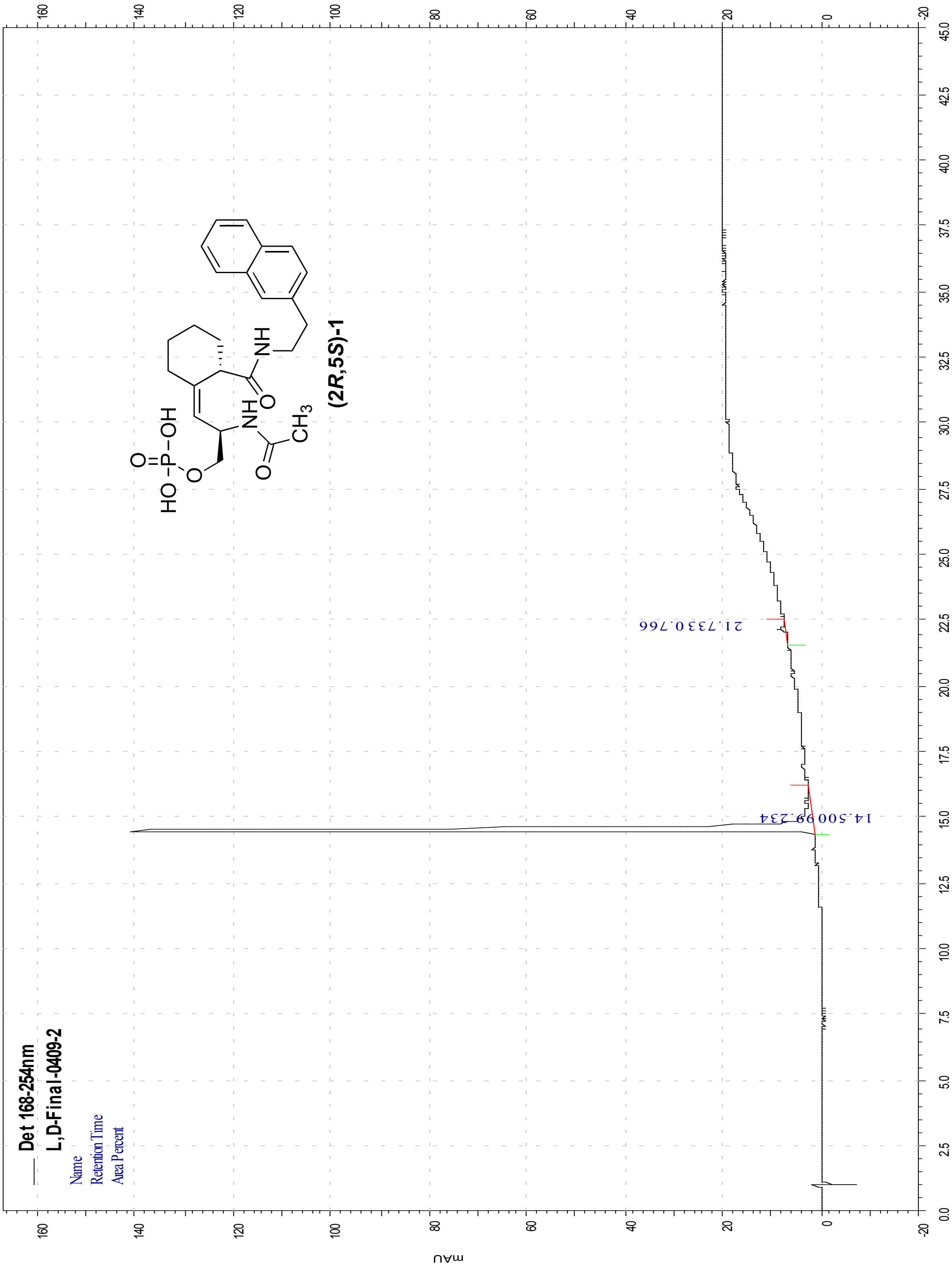

Analytic HPLC of **(2*R*,5*S*)-1**

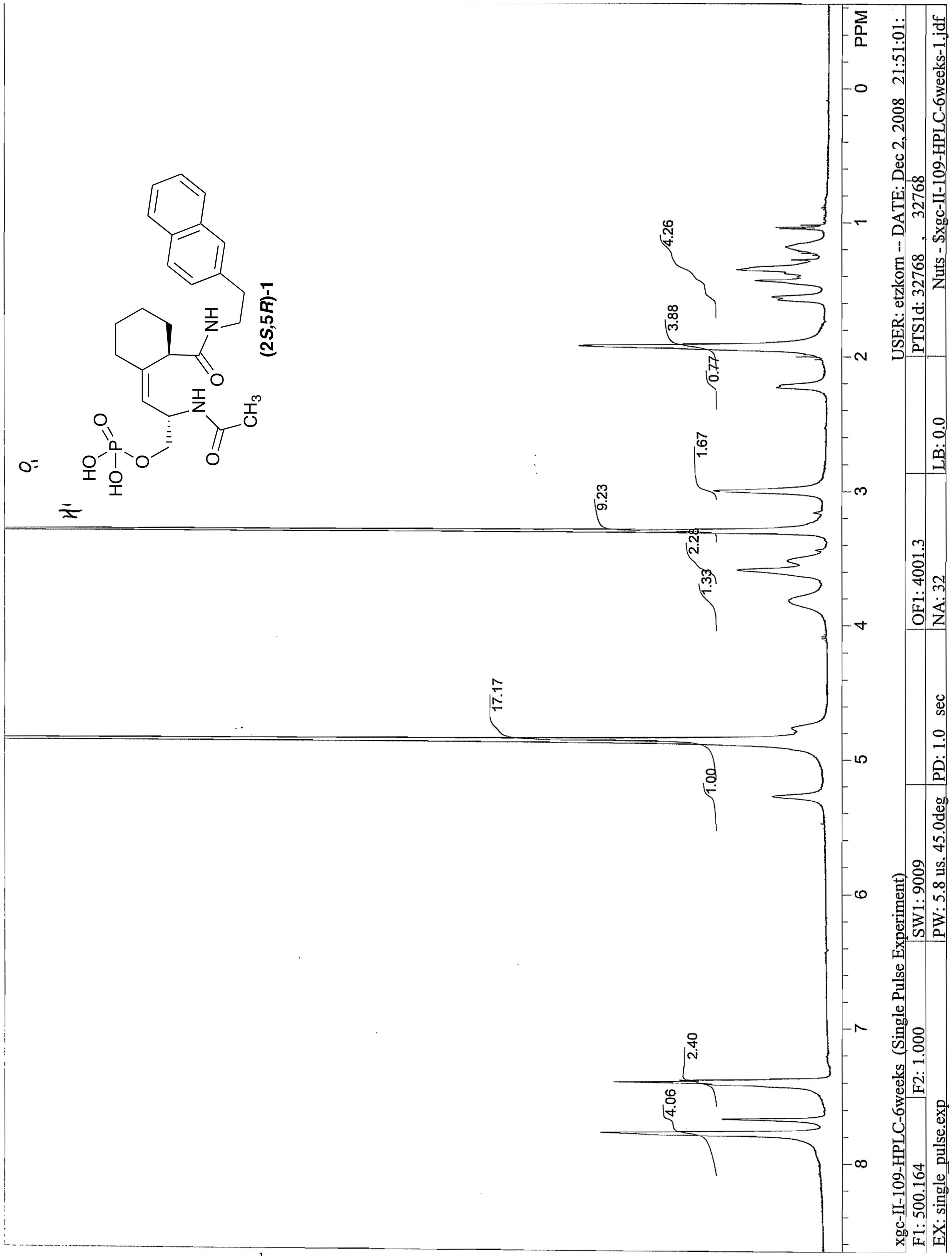

^{1}H NMR of **(2*S*,5*R*)-1** in CD_3OD (500 MHz)

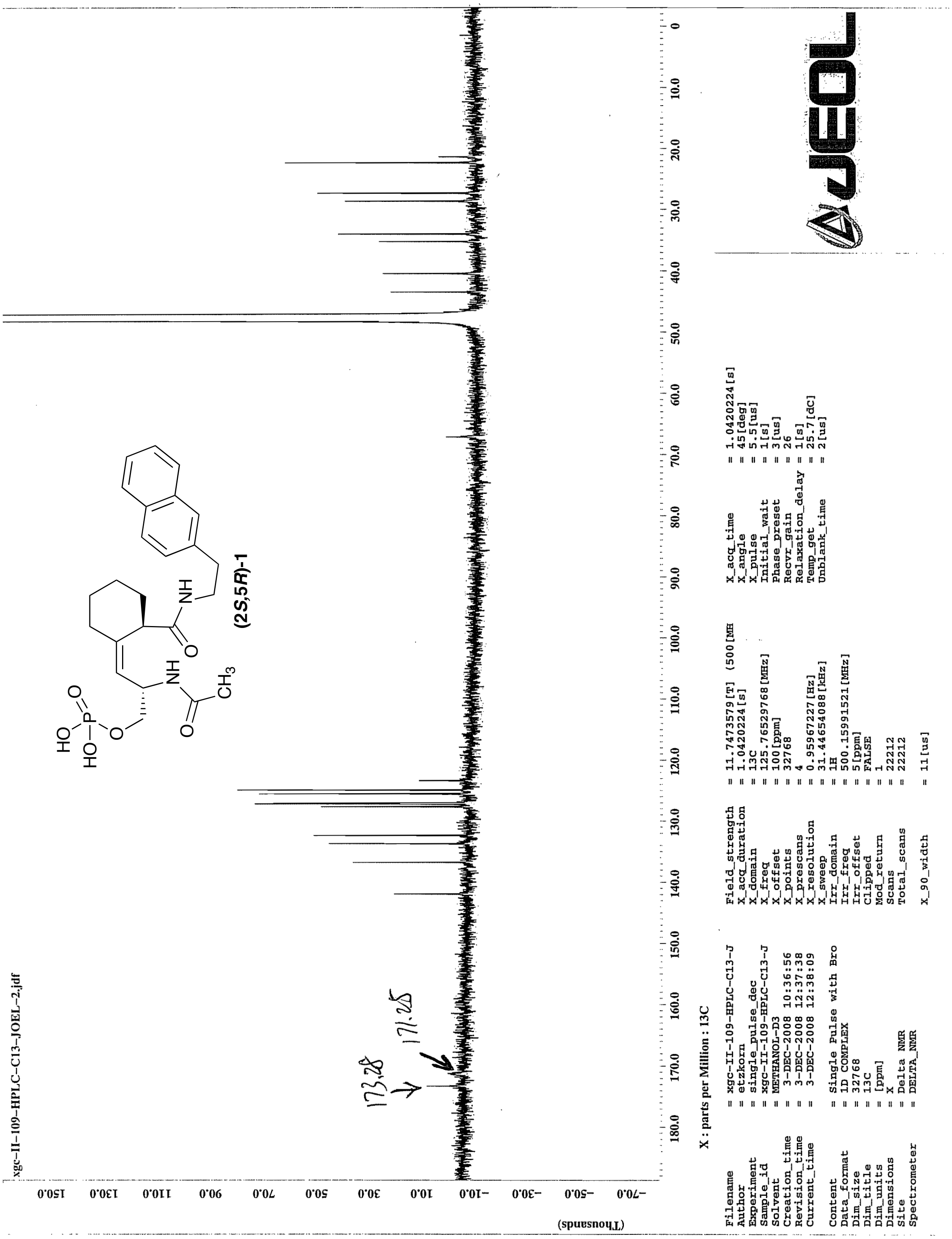

^{13}C NMR of **(2*S*,5*S*)-1** in CD_3OD (125 MHz)

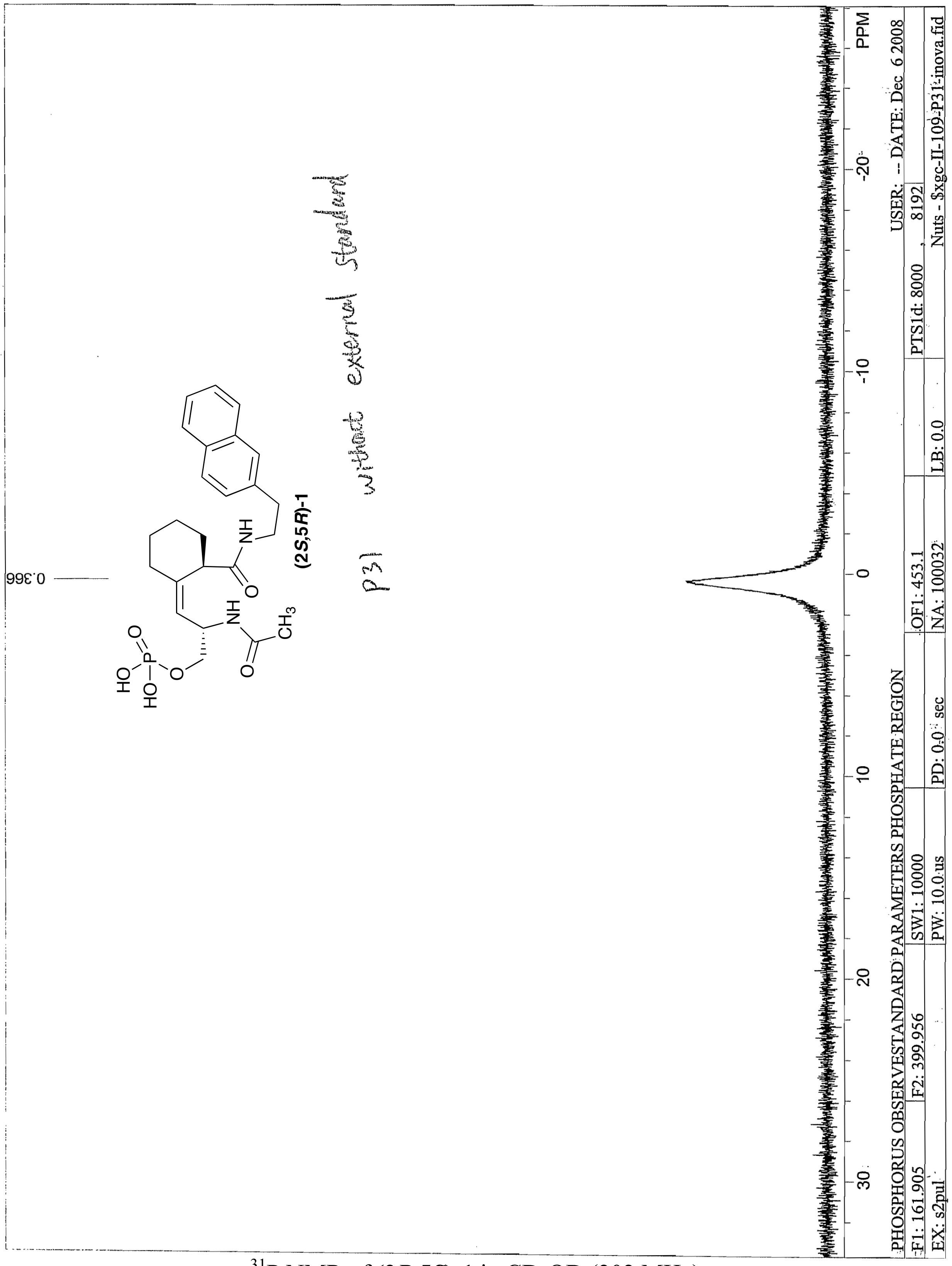

^{31}P NMR of **(2*R*,5*S*)-1** in CD_3OD (202 MHz)

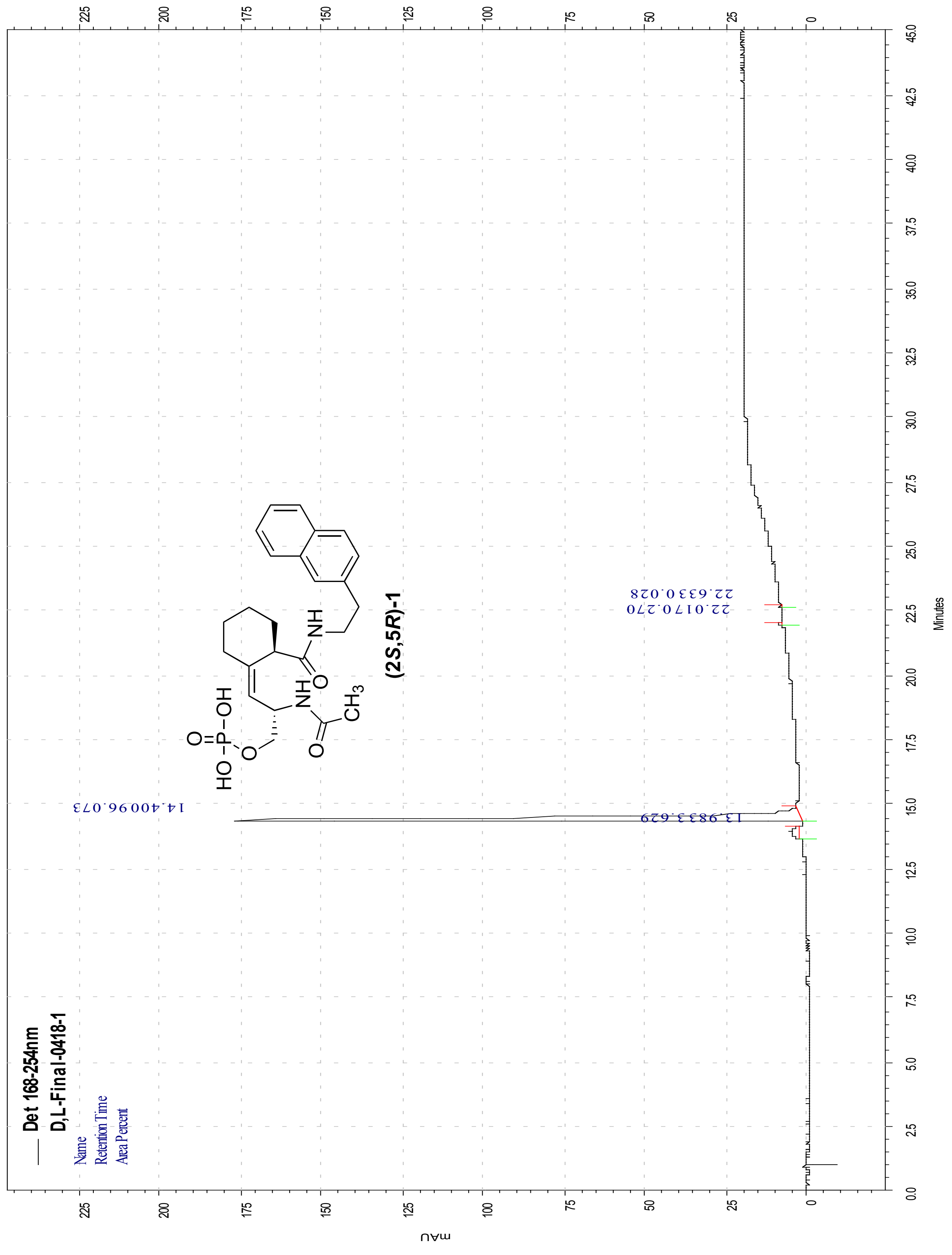

Analytic HPLC of **(2*S*,5*R*)-1**

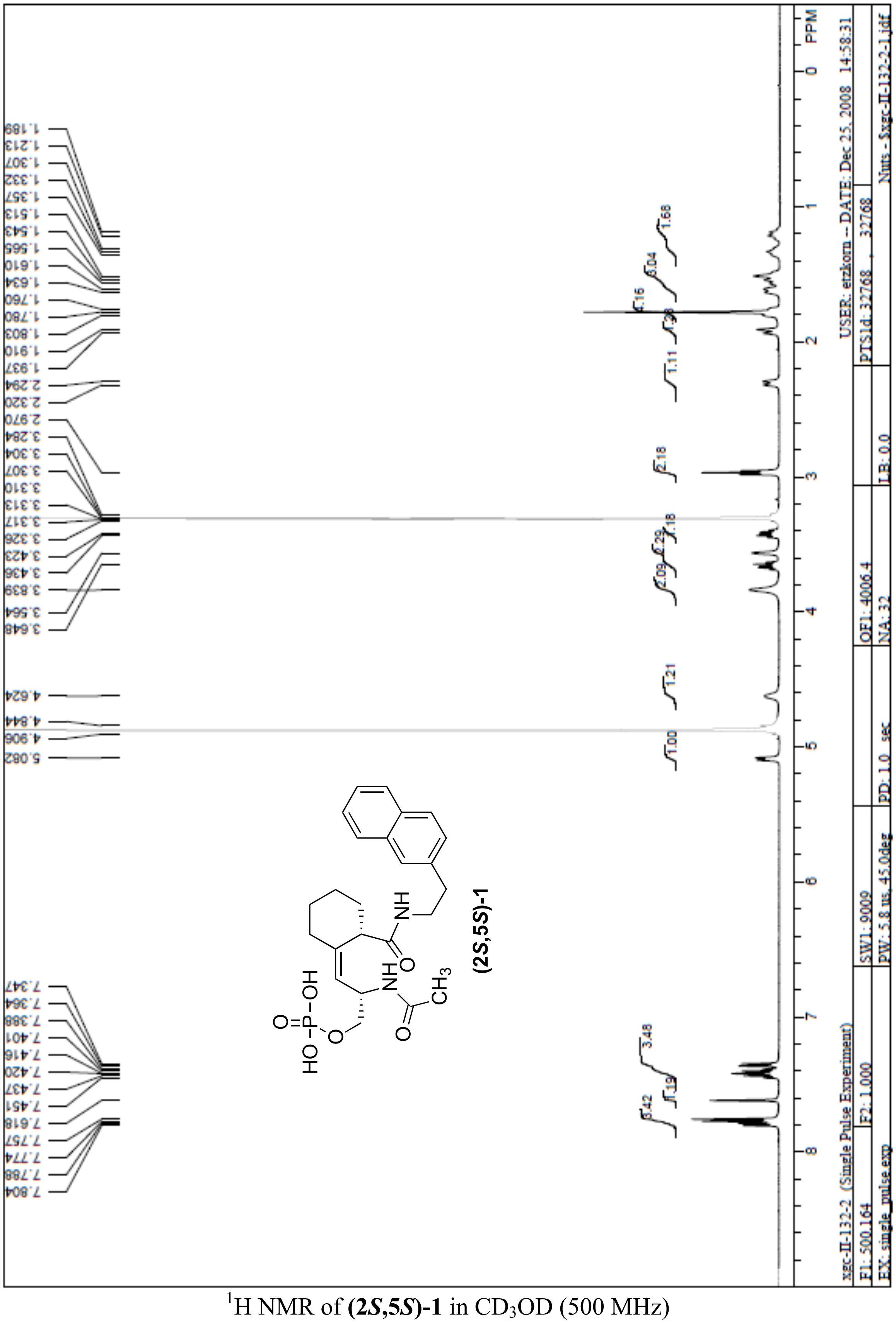

^{1}H NMR of **(2*S*,5*S*)-1** in CD_3OD (500 MHz)

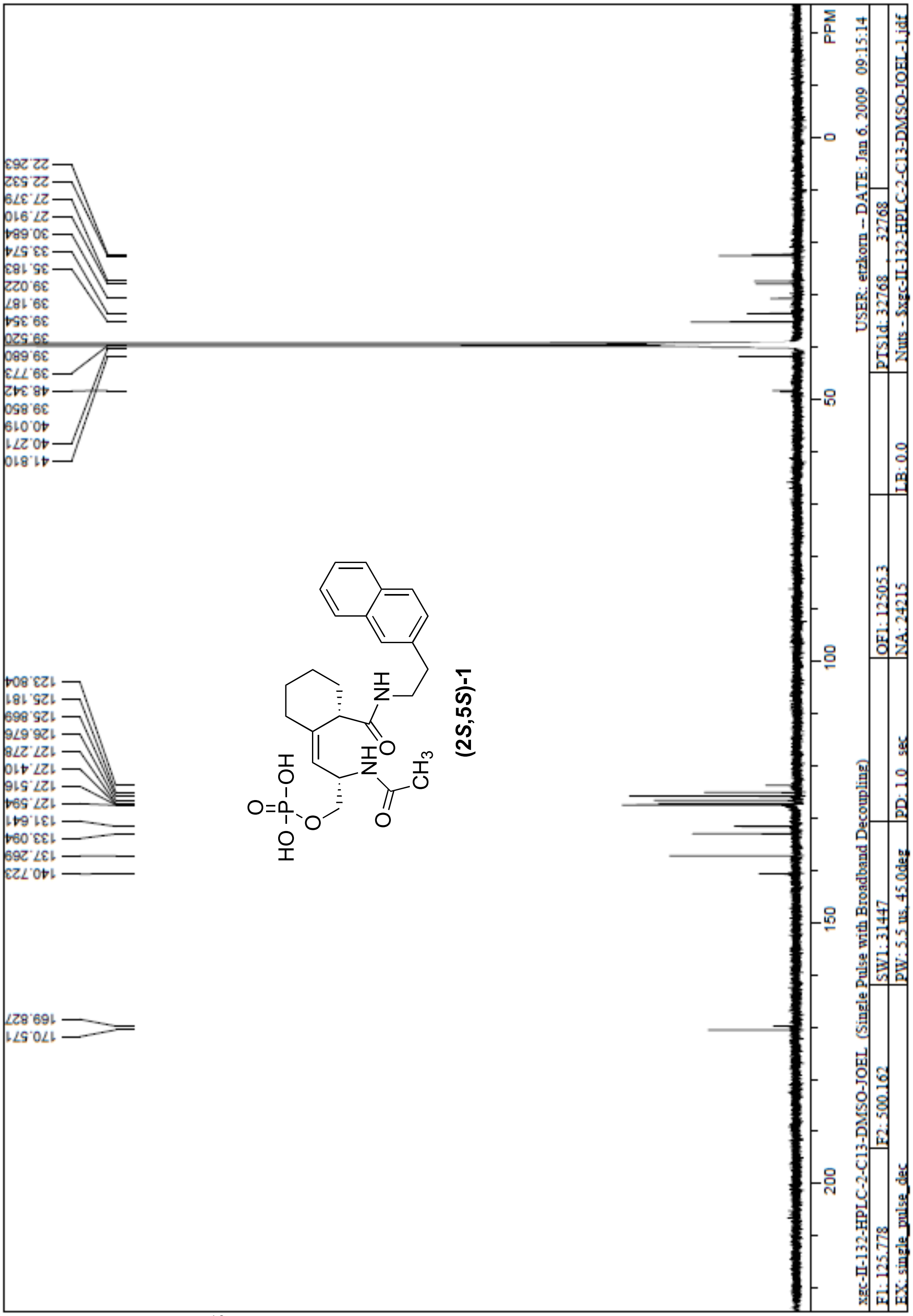

^{13}C NMR of **(2*S*,5*S*)-1** in DMSO-d$_6$ (125 MHz)

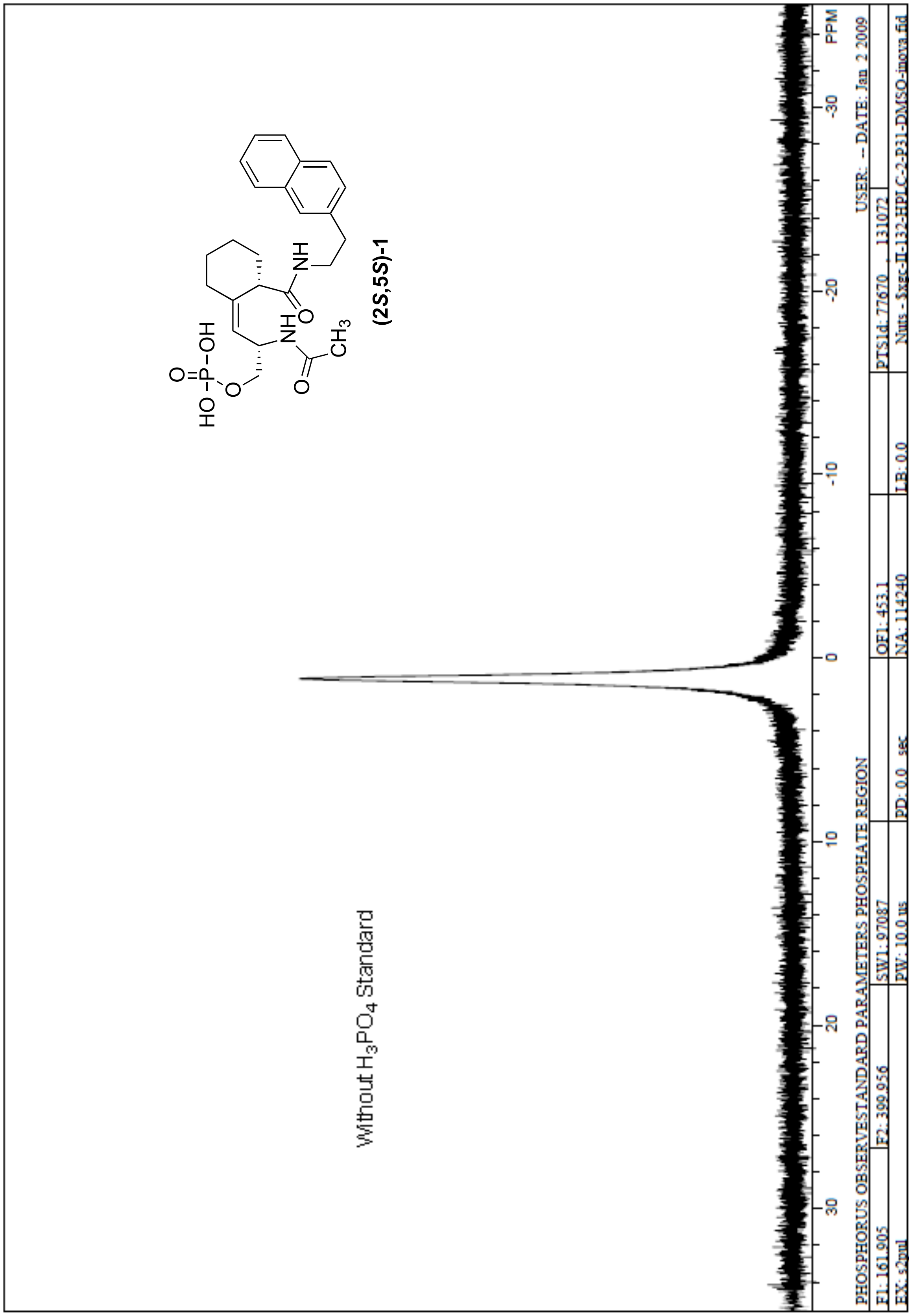

[31] P NMR of **(2*S*,5*S*)-1** in DMSO-d_6 (162 MHz)

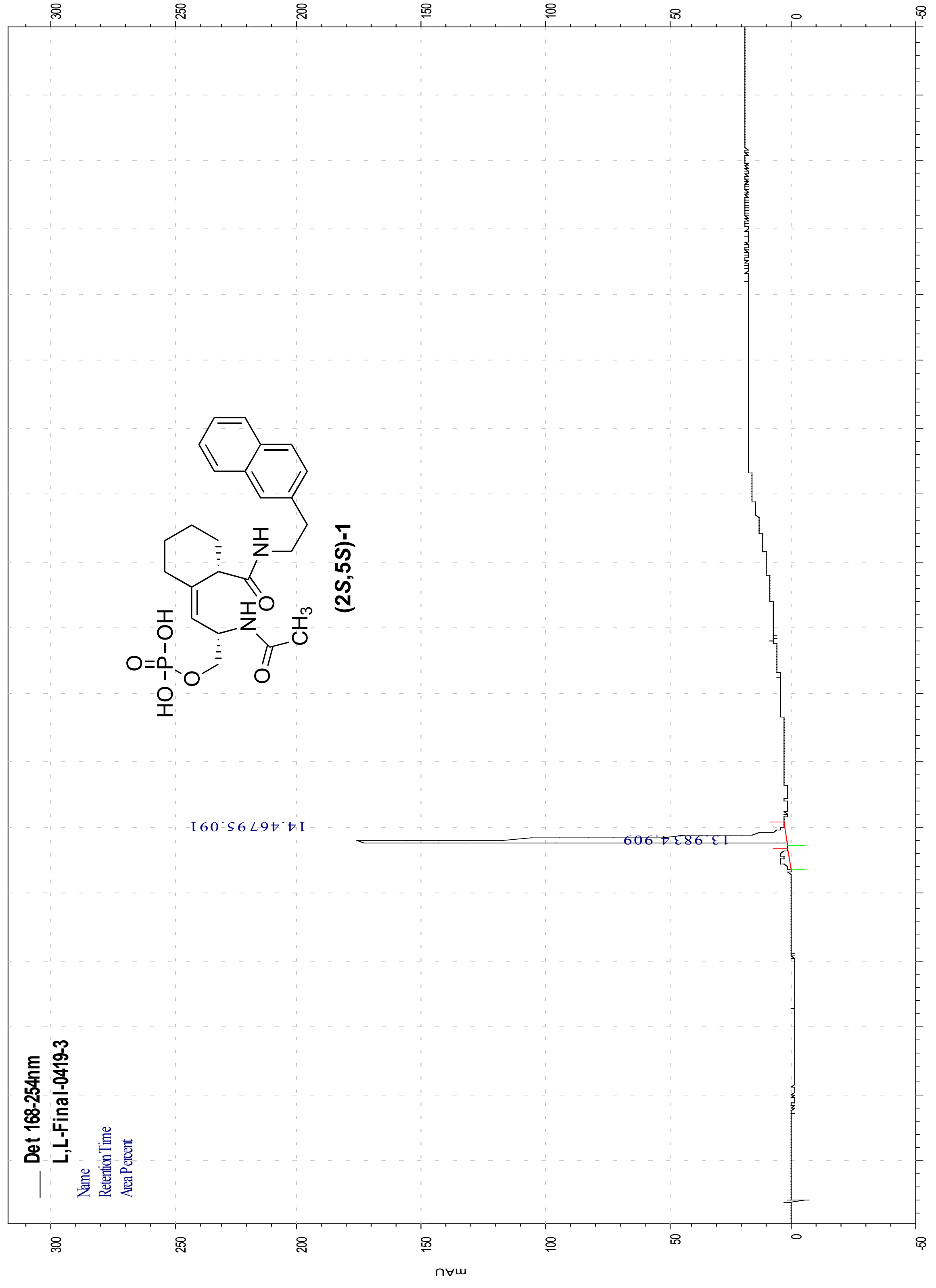

Analytical HPLC of **(2*S*,5*S*)-1**

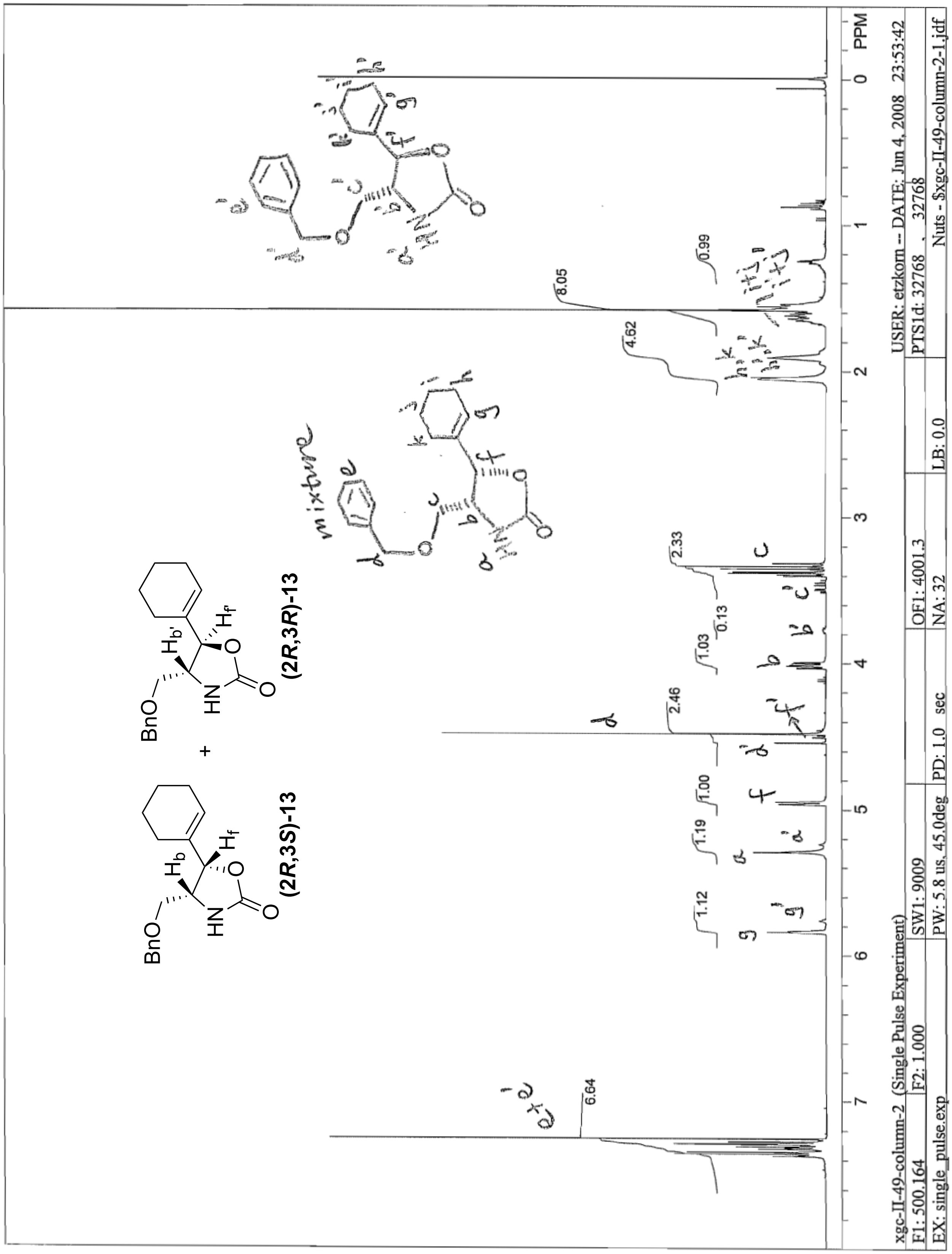

^{1}H NMR of the mixture of **(2*R*,3*S*)-13** and **(2*R*,3*R*)-13** in $CDCl_3$ (500 MHz)

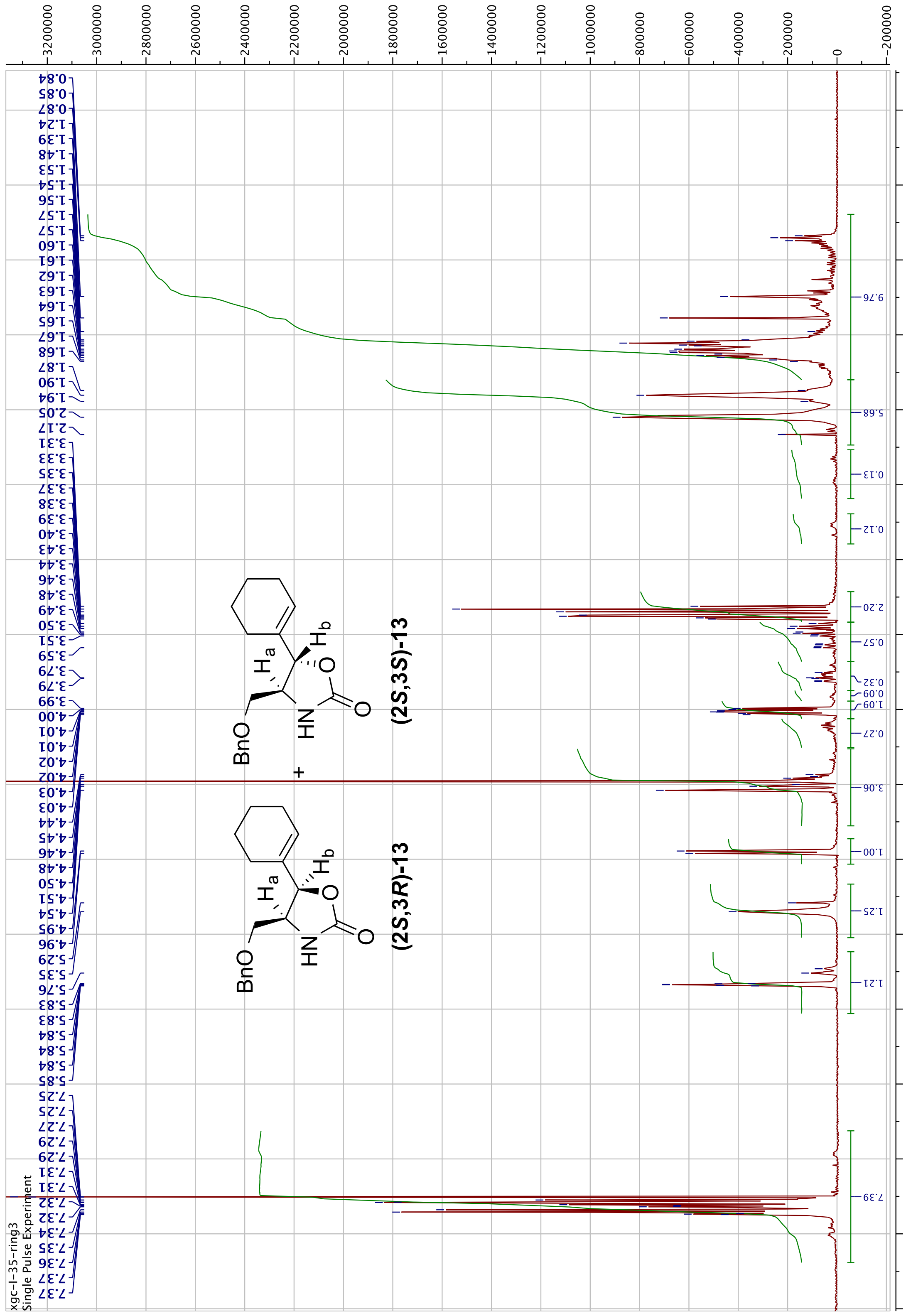

^{1}H NMR of the mixture of **(2*S*,3*R*)-13** and **(2*S*,3*S*)-13** in $CDCl_3$ (500 MHz)

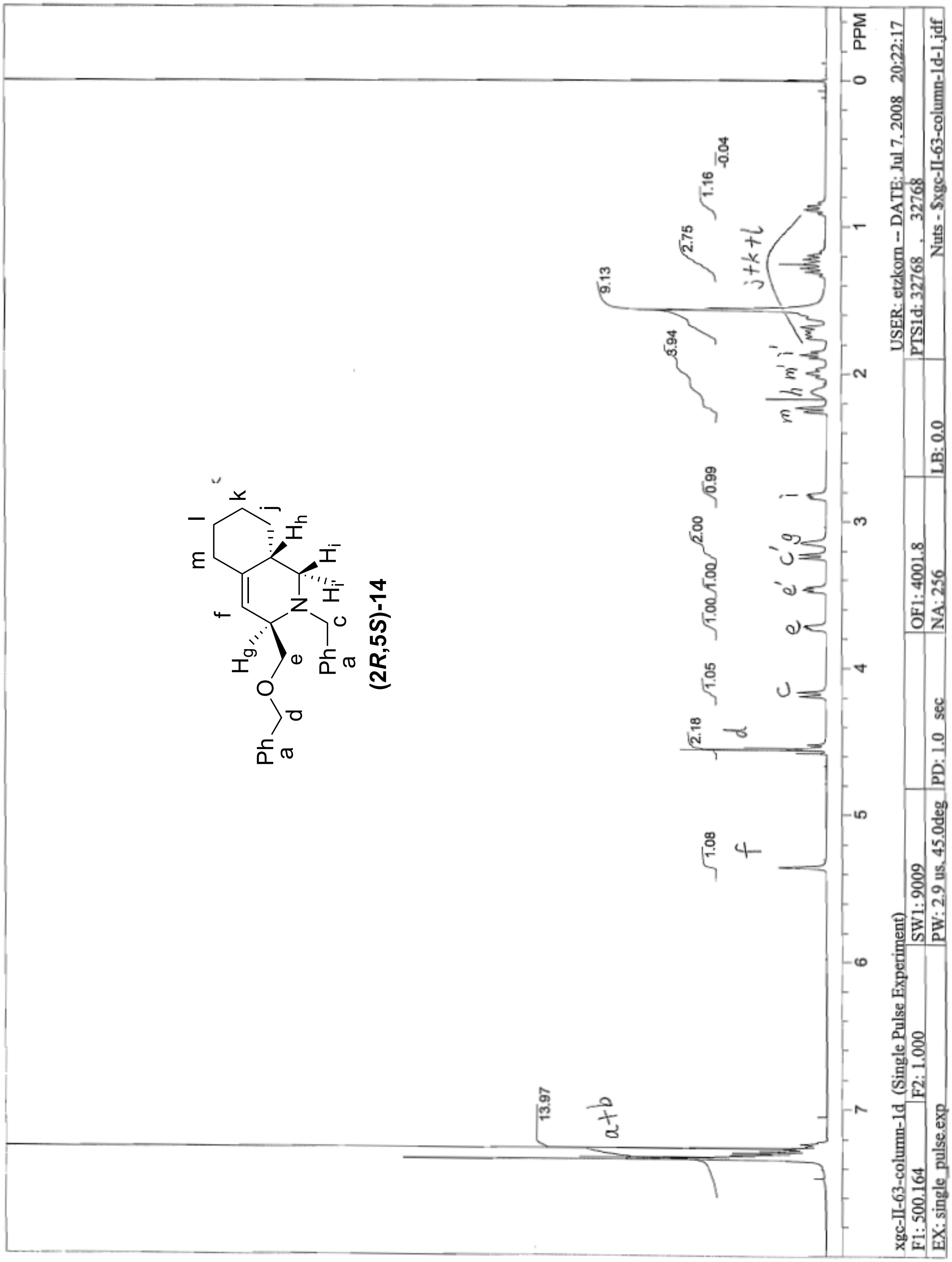

^{1}H NMR of **(2*R*,5*S*)-14** in $CDCl_3$ (500 MHz)

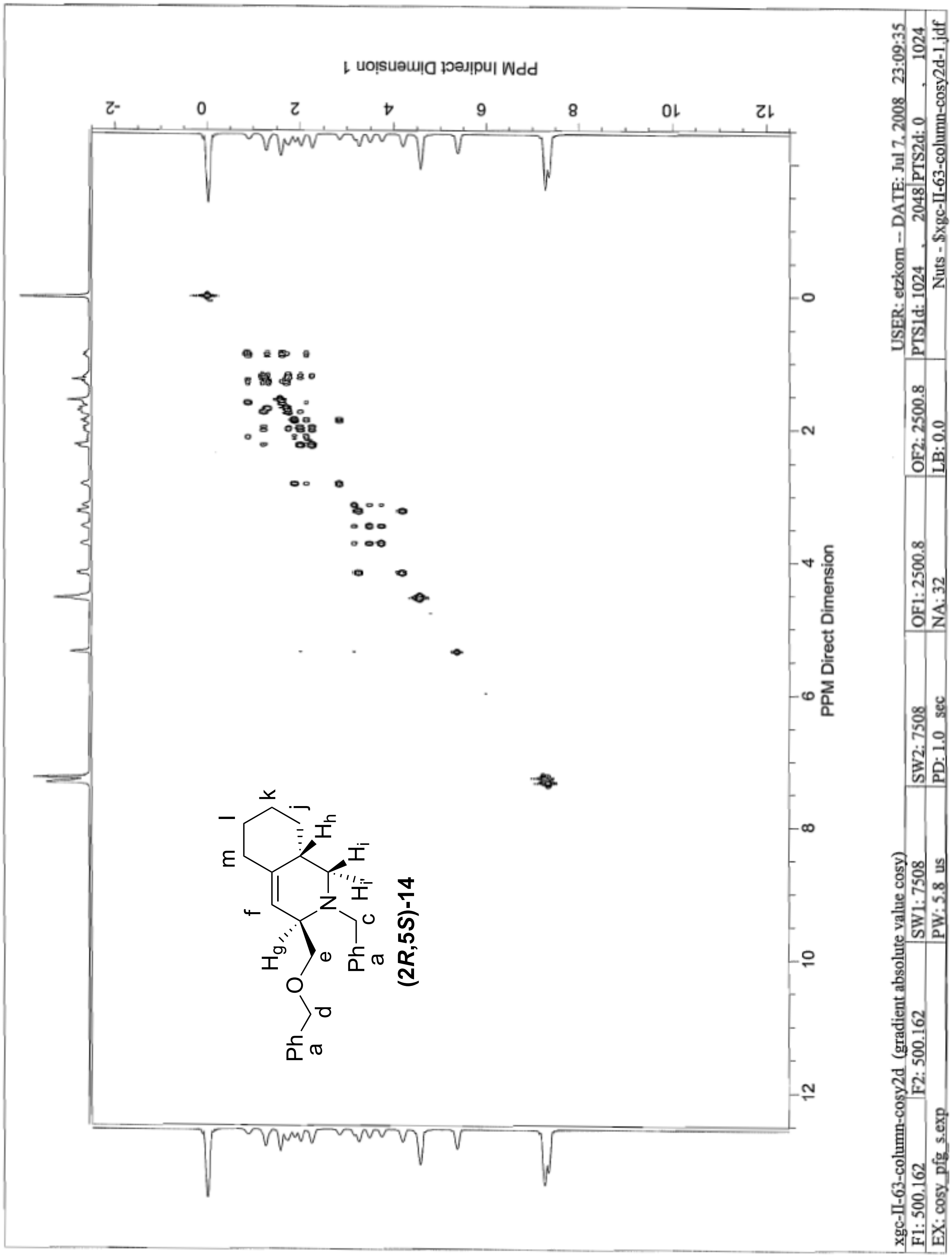

COSY of **(2*R*,5*S*)-14** in $CDCl_3$ (500 MHz)

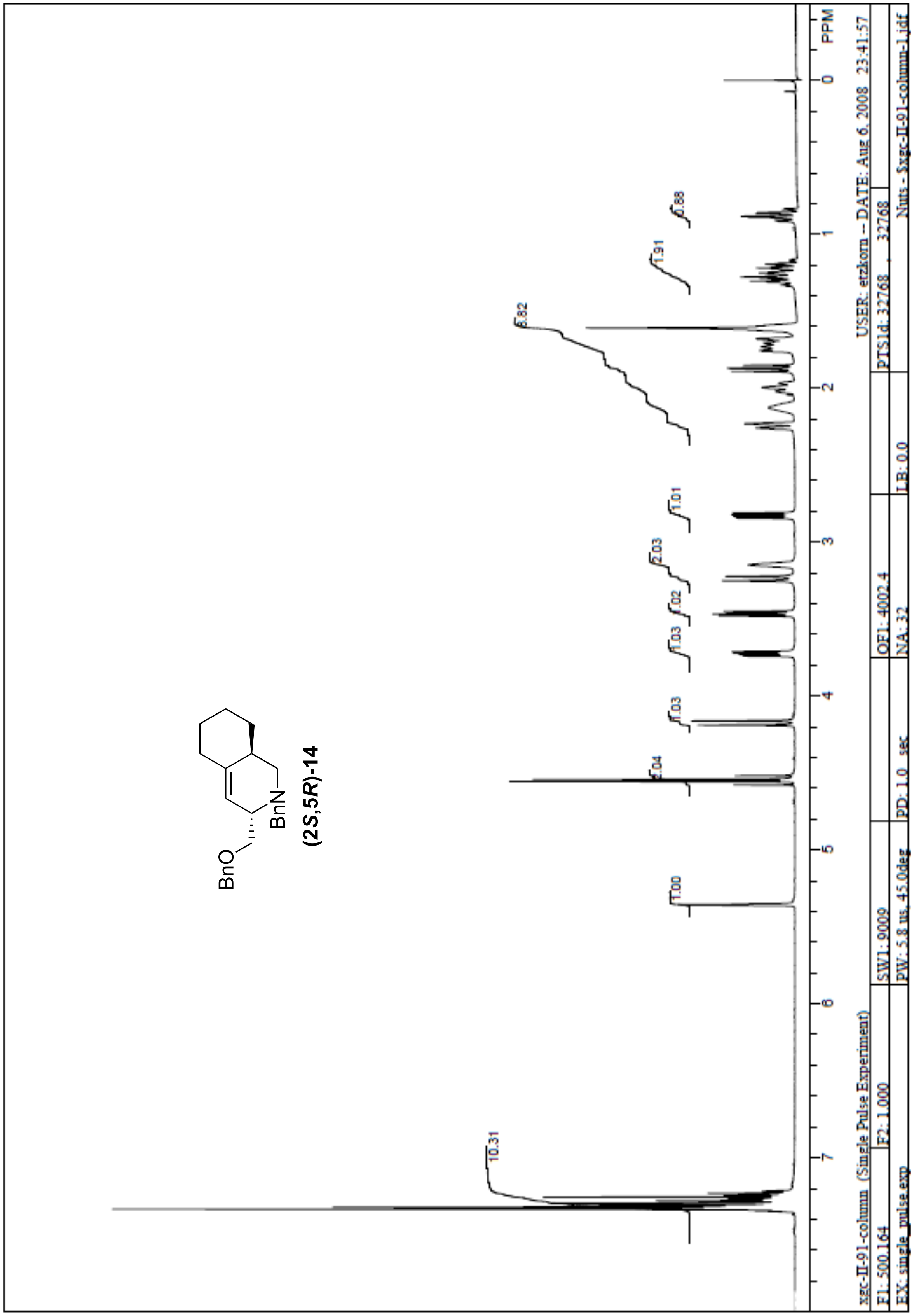

^{1}H NMR of **(2*S*,5*R*)-14** in $CDCl_3$ (500 MHz)

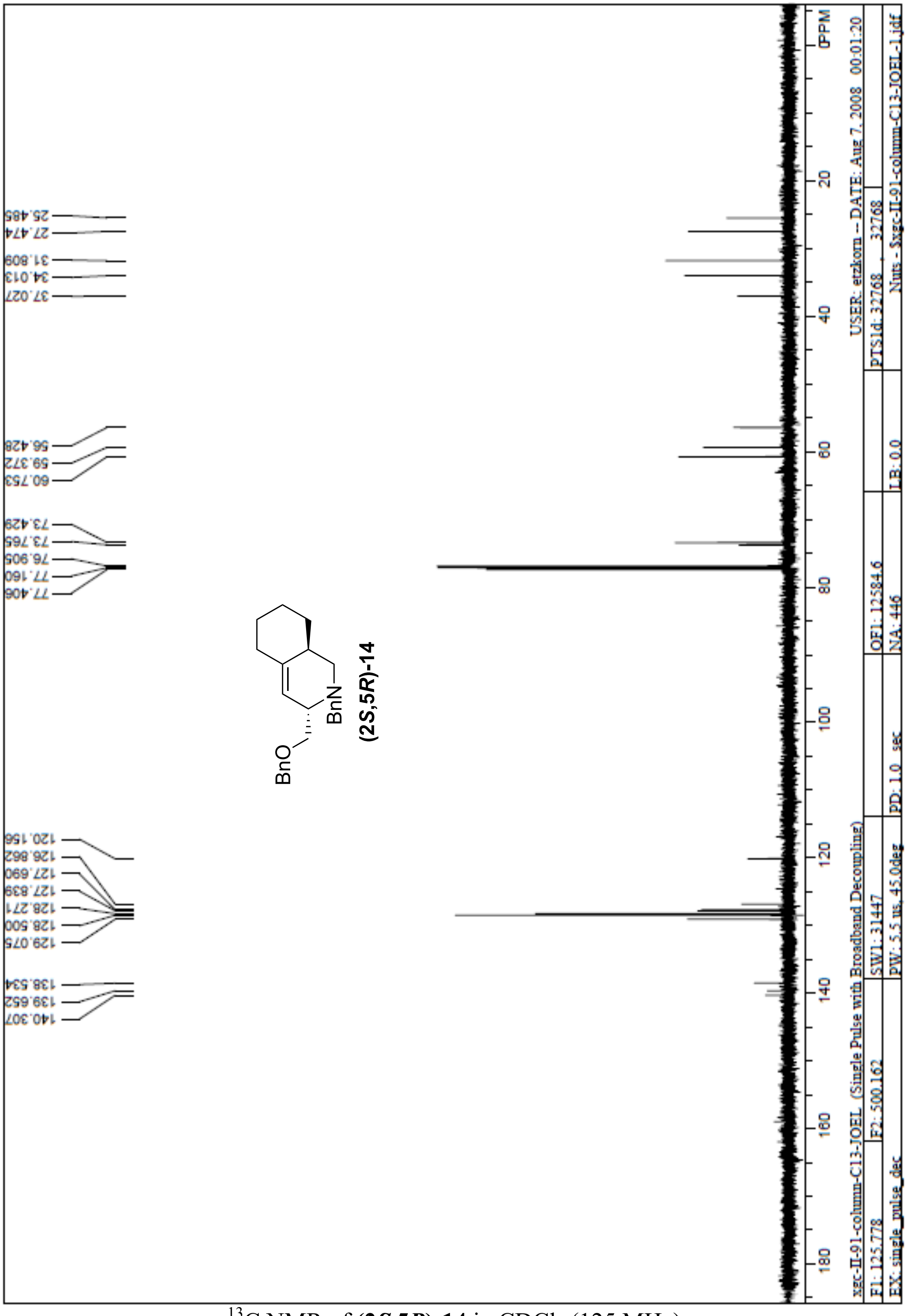

^{13}C NMR of **(2*S*,5*R*)-14** in $CDCl_3$ (125 MHz)

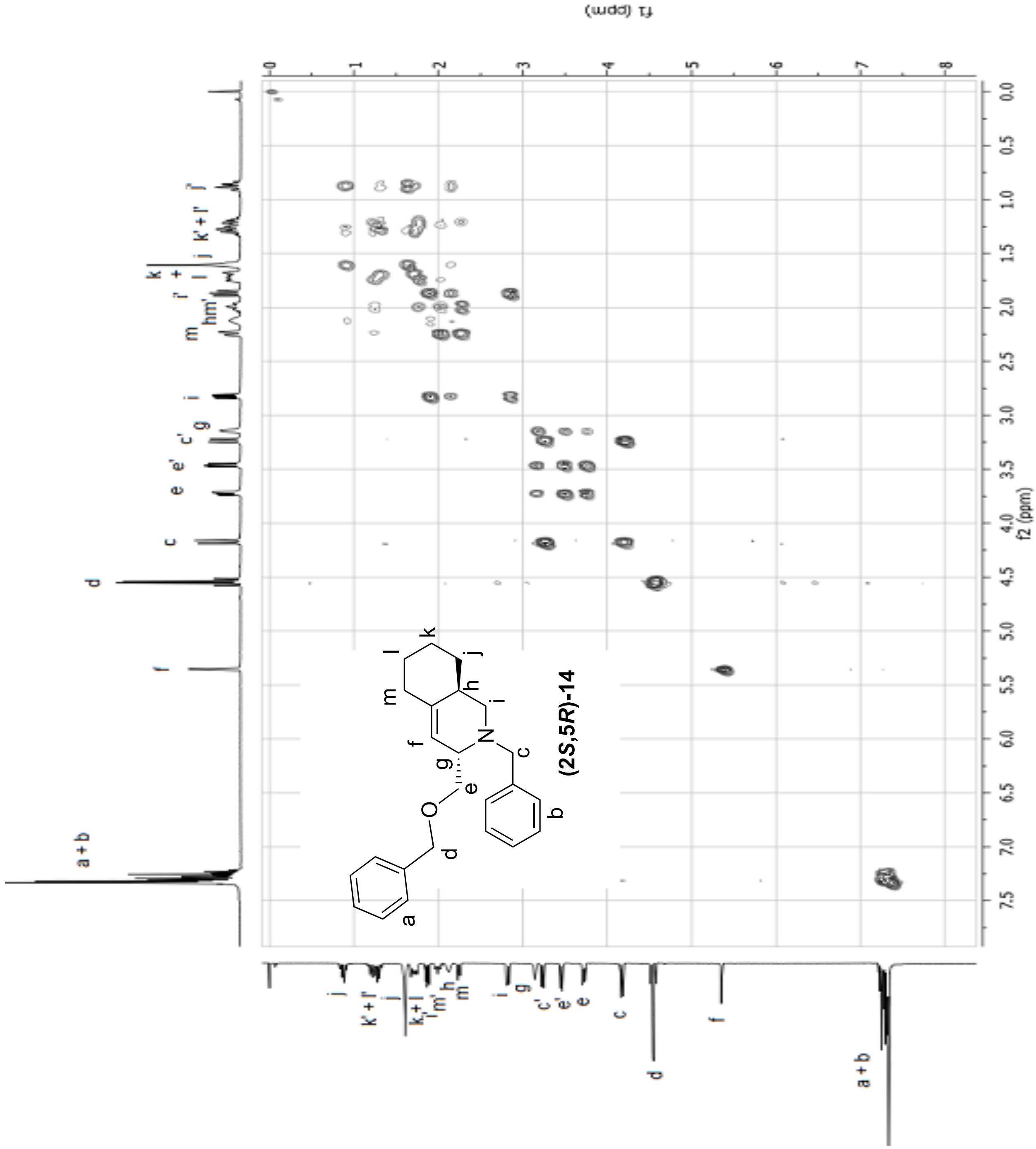

COSY of **(2*S*,5*R*)-14** in $CDCl_3$ (500 MHz)

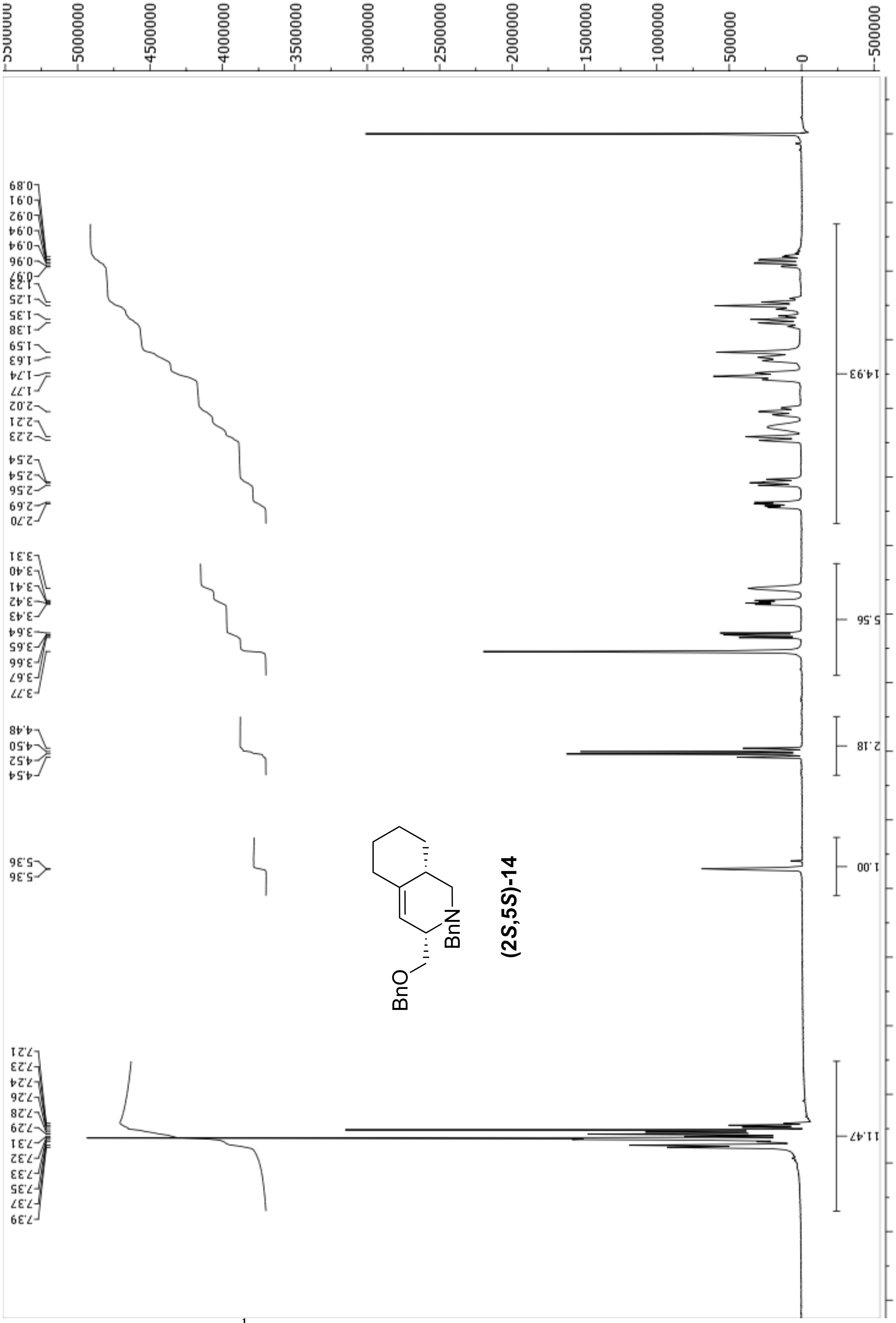

^{1}H NMR of **(2*S*,5*S*)-14** in $CDCl_3$ (500 MHz)

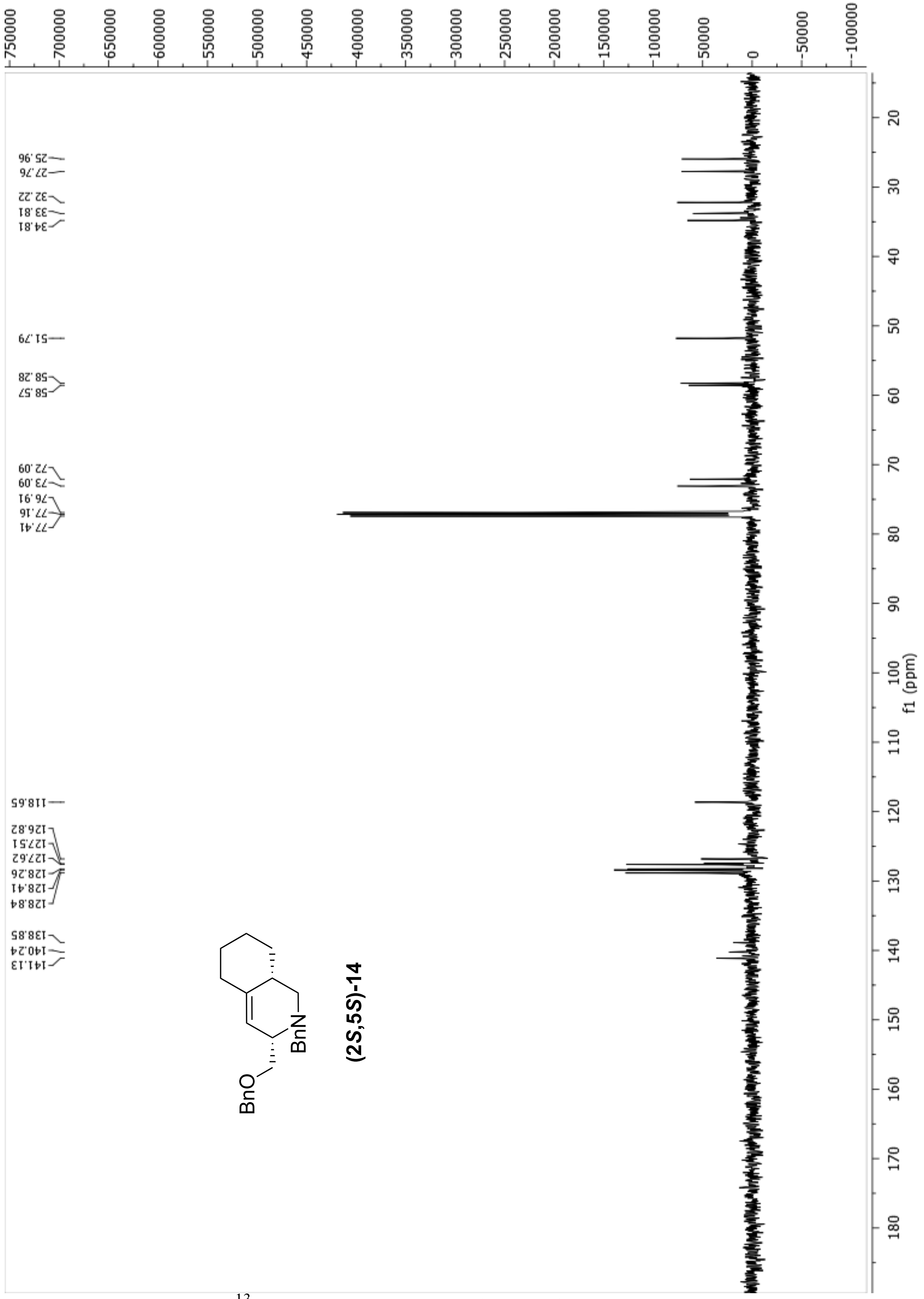

^{13}C NMR of **(2*S*,5*S*)-14** in $CDCl_3$ (125 MHz)

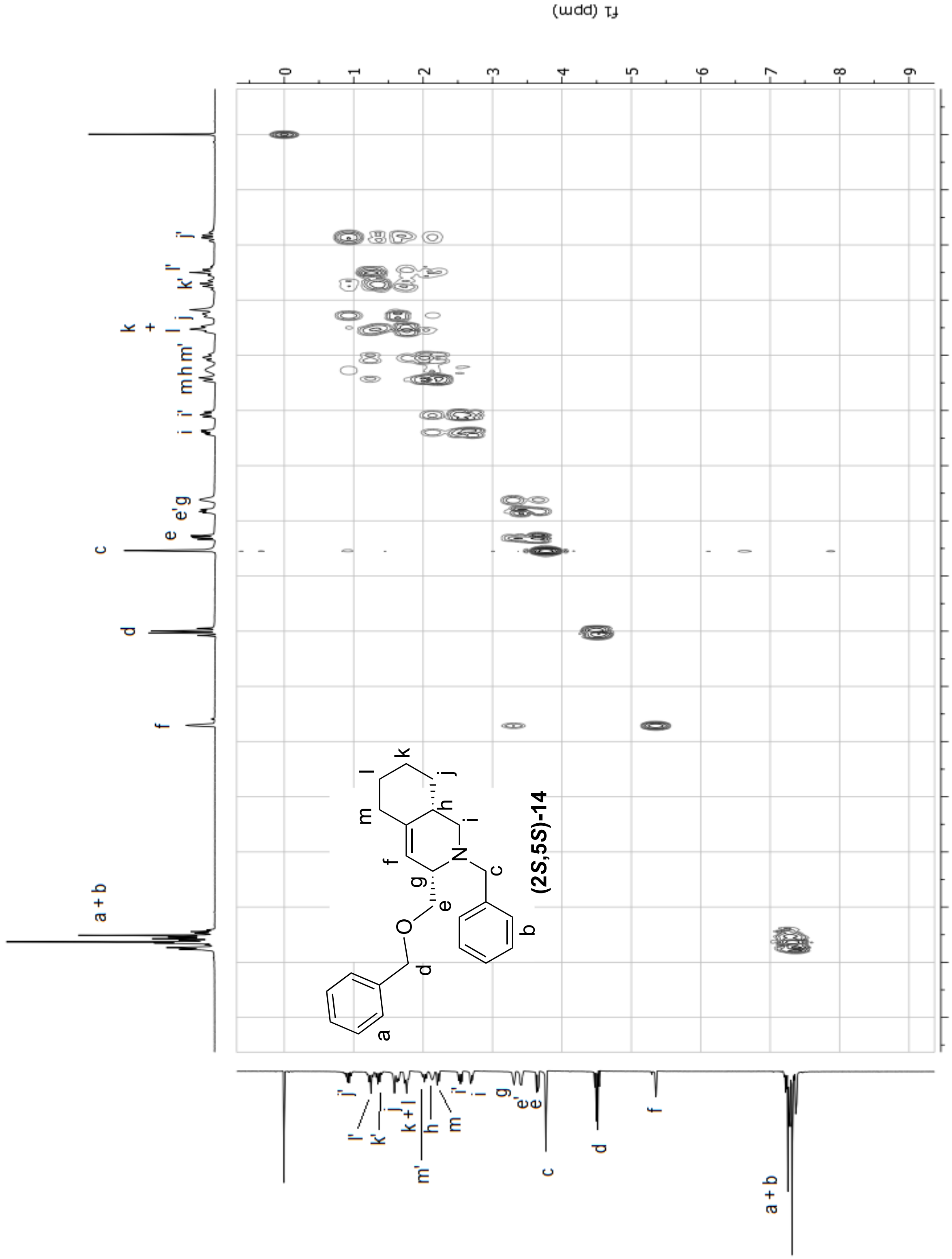

COSY of **(2*S*,5*S*)-14** in $CDCl_3$ (500 MHz)
