# Supplementary material for: Stereochemical Control in the Still-Wittig Rearrangement Synthesis of Cyclohexyl (Z)-Alkene Inhibitors of Pin1
Source: PLoS One. 2015 Oct 7;10(10):e0139543. doi: 10.1371/journal.pone.0139543 (PMC4596862; doi:10.1371/journal.pone.0139543)
Supplement: S2 Dataset — Fig A. Inhibition of Pin1 by (2R,5S)-1, IC50 = 52 ± 4 μM. Fig B. Inhibition of Pin1 by (2S,5R)-1, IC50 = 85 ± 10 μM. Fig C. Inhibition of Pin1 by (2S,5S)-1, IC50 = 140 ± 20 μM. (PDF) [file pone.0139543.s002.pdf]

Electronic Supporting Information: S2 Dataset

Stereochemical control in the Still-Wittig rearrangement synthesis of cyclohexyl (*Z*)-alkene inhibitors of Pin1

Xingguo R. Chen, Shuang A. Fan, Rachel I. Ware, and Felicia A. Etzkorn*

Department of Chemistry, Virginia Tech, Blacksburg, Virginia 24061
*Corresponding author
E-mail: fetzkorn@vt.edu

Pin1 inhibition plots for (2*R*,5*S*)-1, (2*S*,5*R*)-1and (2*S*, 5*S*)-1.

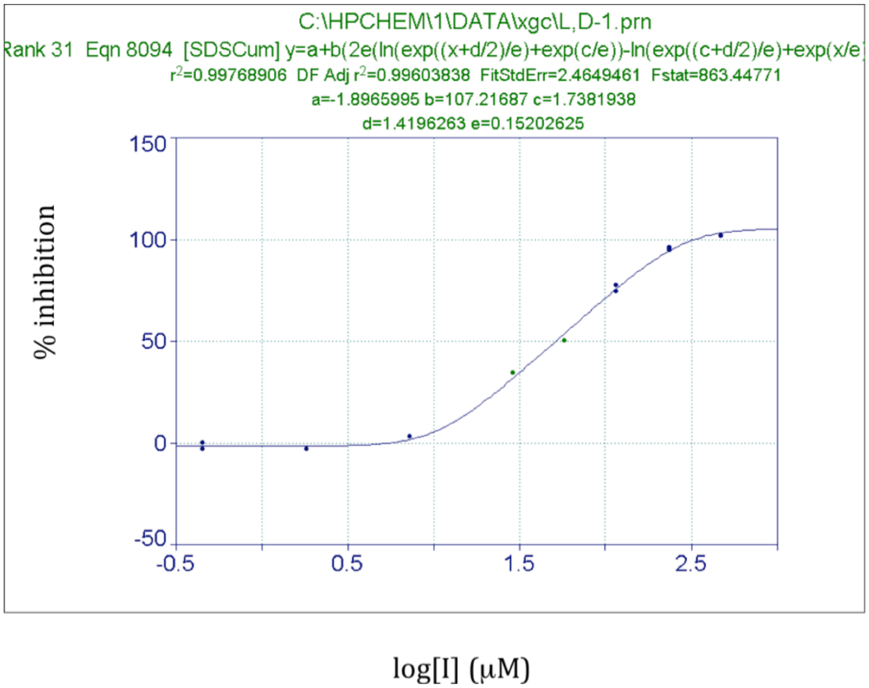

Fig A in S2 Dataset. Inhibition of Pin1 by (2*R*,5*S*)-1, IC_{50} = 52 ± 4 μM.

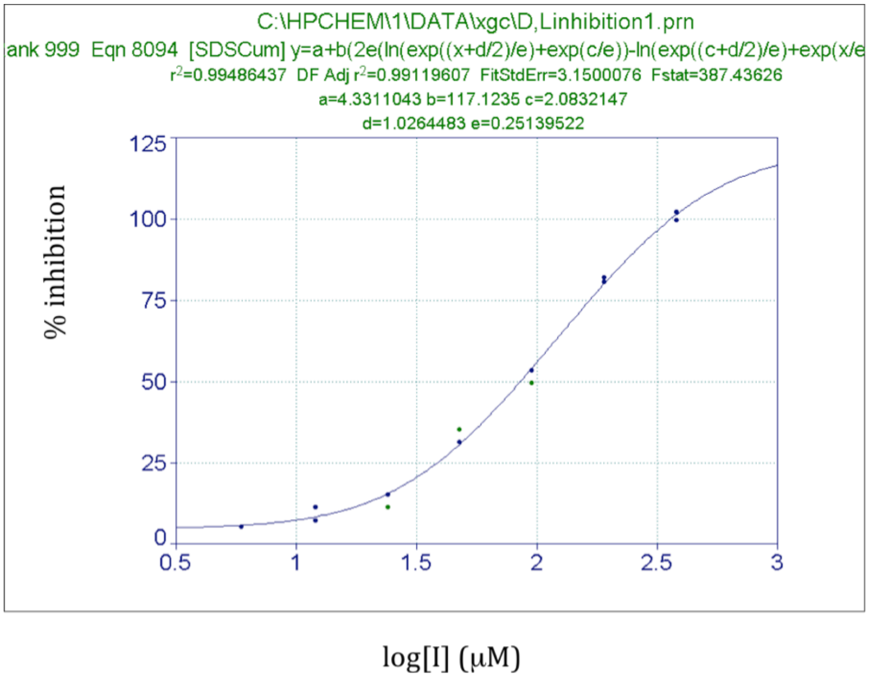

Fig B in S2 Dataset. Inhibition of Pin1 by (2*S*,5*R*)-1, IC_{50} = 85 ± 10 μM.

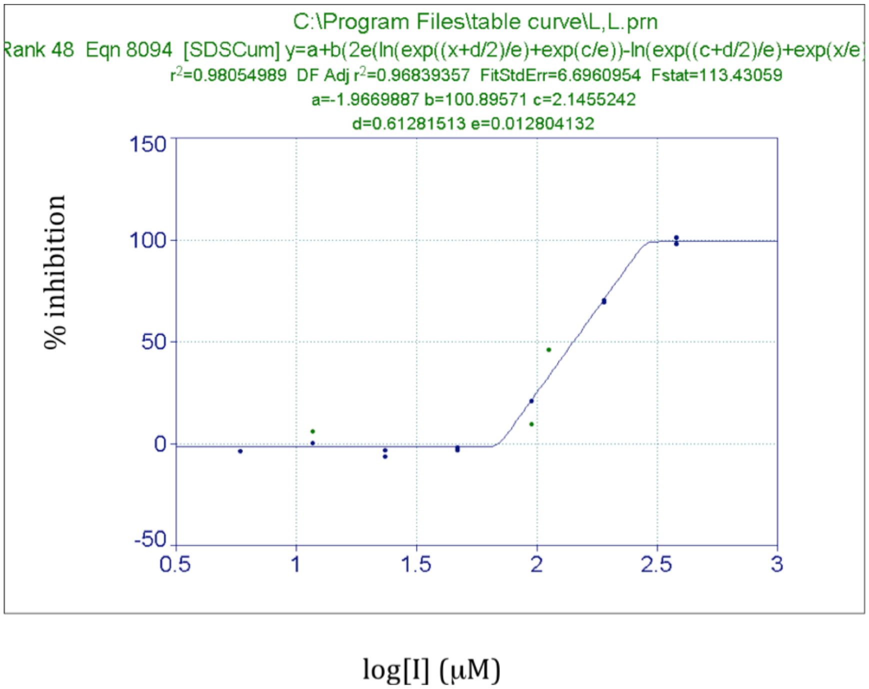

Fig C in S2 Dataset. Inhibition of Pin1 by (2*S*,5*S*)-1, IC_{50} = 140 ± 20 μM.
